# SWATH‐based proteomics reveals processes associated with immune evasion and metastasis in poor prognosis colorectal tumours

**DOI:** 10.1111/jcmm.14693

**Published:** 2019-09-27

**Authors:** Laura M. López‐Sánchez, Rafael Jiménez‐Izquierdo, Jon Peñarando, Rafael Mena, Silvia Guil‐Luna, Marta Toledano, Francisco Conde, Carlos Villar, César Díaz, Ignacio Ortea, Juan R. De la Haba‐Rodríguez, Enrique Aranda, Antonio Rodríguez‐Ariza

**Affiliations:** ^1^ Instituto Maimónides de Investigación Biomédica de Córdoba (IMIBIC) Córdoba Spain; ^2^ Centro de Investigación Biomédica en Red de Cáncer (CIBERONC) Madrid Spain; ^3^ Unidad de Gestión Clínica de Anatomía Patológica Hospital Universitario Reina Sofía Córdoba Spain; ^4^ Unidad de Gestión Clínica de Cirugía General y del Aparato Digestivo Hospital Universitario Reina Sofía Córdoba Spain; ^5^ Unidad de Gestión Clínica de Oncología Médica Hospital Universitario Reina Sofía Universidad de Córdoba Córdoba Spain

**Keywords:** colorectal cancer, immune evasion, mesenchymal, metastasis, proteomics, SWATH

## Abstract

Newly emerged proteomic methodologies, particularly data‐independent acquisition (DIA) analysis–related approaches, would improve current gene expression–based classifications of colorectal cancer (CRC). Therefore, this study was aimed to identify protein expression signatures using SWATH‐MS DIA and targeted data extraction, to aid in the classification of molecular subtypes of CRC and advance in the diagnosis and development of new drugs. For this purpose, 40 human CRC samples and 7 samples of healthy tissue were subjected to proteomic and bioinformatic analysis. The proteomic analysis identified three different molecular CRC subtypes: P1, P2 and P3. Significantly, P3 subtype showed high agreement with the mesenchymal/stem‐like subtype defined by gene expression signatures and characterized by poor prognosis and survival. The P3 subtype was characterized by decreased expression of ribosomal proteins, the spliceosome, and histone deacetylase 2, as well as increased expression of osteopontin, SERPINA 1 and SERPINA 3, and proteins involved in wound healing, acute inflammation and complement pathway. This was also confirmed by immunodetection and gene expression analyses. Our results show that these tumours are characterized by altered expression of proteins involved in biological processes associated with immune evasion and metastasis, suggesting new therapeutic options in the treatment of this aggressive type of CRC.

## 1 INTRODUCTION

Globally, colorectal cancer (CRC) is the third most commonly diagnosed cancer in men and the second in women, with two million new cases and around 800 000 deaths in 2018.^1^ Although CRC mortality has declined slightly over the past two decades, and despite advances in detection and surgical management, metastatic CRC (mCRC) is associated with poor prognosis, with 5‐year survival rates with a range from 5% to 8%.^2^

CRC is often classified into different phenotypes according to genetic alterations, including chromosomal instability (CIN) and microsatellite instability (MSI), and epigenetic changes such as silencing of genes due to methylation of the CpG islands (CIM) located in their promoters.^3^ In addition, recent gene expression–based studies have been reported to classify CRC patients into distinct molecular subtypes.^4^, ^5^, ^6^ More recently, the analysis of the interrelation of the proposed CRC subtypes has provided evidence for the existence of consensus molecular subtypes.^7^ Interestingly, a stem‐like/mesenchymal subtype associated with poor patient outcome was identified in all these gene‐based classifications.^8^ These molecular classifications can therefore identify different CRC phenotypes, which confer a different behaviour from the point of view of prognosis or response to specific treatments. This complexity is highly relevant for the development of more effective clinical treatments, through the identification of novel, more specific therapeutic targets and biomarkers that determine different phenotypes, which will lead to an individualized treatment for patients with CRC.

Proteomics could expand our current knowledge on abnormal processes among different CRC subtypes identifying new proteins or protein profiles to improve current classifications. In addition, proteomics could provide biomarkers that define differences in resistance to therapy, prognosis and metastatic spread in a specific subtype. Mass spectrometry (MS)–based technologies are powerful tools for the investigation of biomarkers in clinical samples,^9^ and SWATH‐MS (Sequential Window Acquisition of all THeoretical Mass Spectra‐MS) is an innovative MS approach that allows the quantification of almost all peptides and proteins present in a single sample being useful in large sample cohort studies.^10^, ^11^

Therefore, the aim of our study was to identify and quantify differential expression of proteins in clinical samples of CRC using the SWATH‐MS approach of proteomic analysis and to establish potential subtypes of CRC based on differential expression of proteins.

## 2 MATERIALS AND METHODS

### 2.1 Subjects

Forty patients over 18 years of age with resectable colorectal cancer submitted to surgery in Reina Sofía University Hospital (Córdoba, Spain) were included in this study (Table S1). In addition, 7 samples of healthy tissue from these patients (5 men and 2 women; mean age 67 ± 5 year) were used as controls. An independent cohort of 45 FFPE tumour samples was retrospectively analysed to confirm the association between CMS subtype and the SRSF3 and SERPINA1 expression (Table S2). The study protocol was approved by the Ethics Committee of the Reina Sofia University Hospital, according to the Code of Ethics of the World Medical Association (Declaration of Helsinki), and signed informed written consent was obtained from each patient.

### 2.2 Sample preparation and protein extraction

Fresh tissue samples were washed with phosphate‐buffered saline (PBS) at pH 7.4 (Sigma‐Aldrich), being directly frozen in liquid nitrogen and stored at −80°C until further use. Protein lysates from fresh‐frozen colorectal tissues were obtained by mechanical disruption of 100 mg of tissue by homogenizer pestle using a sample grinding kit (GE Healthcare) in 300 μL of lysis buffer (20 mmol/L Tris‐HCl, pH 7.6; 0.5 M sucrose; 0.15 M KCl; dithiothreitol; PMSF; and 1 x anti‐protease cocktail from Sigma‐Aldrich), incubating on ice for 10 minutes and centrifugation at 25 000 × *g* for 15 minutes at 4°C. Supernatants were collected, and total protein was quantified using the Qubit Protein Assay Kit (Thermo Fisher Scientific).

### 2.3 Sample preparation for LC‐MS analysis

Protein precipitation with trichloroacetic acid (TCA)/acetone was carried out to remove contaminants from the samples. Thus, TCA was added to the samples to a final concentration of 10% and was incubated on ice for 30 minutes. Then, precipitated proteins were collected by centrifugation for 30 minutes at 8000 × *g* and 4°C. Pellets were resuspended with ice‐cold acetone and were incubated at −20°C overnight. After performing a second centrifugation step, protein was solubilized in 50 μL of 0.2% RapiGest SF (Waters) with 50 mmol/L ammonium bicarbonate. Qubit Protein Assay Kit allowed to measure total protein, and 50 μg of protein was trypsin‐digested.^12^ Proteomic analysis was carried out in the Proteomics Unit of the Maimonides Institute of Biomedical Research of Cordoba (IMIBIC). MS data have been deposited to the ProteomeXchange Consortium via the PRIDE 13 partner repository with the data set identifier PXD007810 (Username: reviewer78273@ebi.ac.uk; Password: lvwvsWOi).

### 2.4 Creation of the spectral library

In SWATH pipeline, chromatogram traces for the peptide fragment ions are matched to the peptides and proteins contained in a peptide spectral library, and then, the fragment chromatogram traces are used for peptide and protein quantitation. SWATH method heavily relies on the peptide spectral library, which is previously established by shotgun proteomic (data‐dependent acquisition, DDA, runs) analysis of the same pooled samples. Therefore, the 47 samples were mixed in 9 pools, to maximize the number of peptides and proteins contained in the spectral library, which were analysed by LC‐MS/MS for DDA massive protein identification (shotgun proteomics). In this way, all samples are represented in the DDA runs used in the database search used for building the spectral library.

Identification of peptides and proteins was carried out using the Protein Pilot software (version 5.0.1; Sciex) with a human Swiss‐Prot database (March 2016). False discovery rate (FDR), calculated by Protein Pilot using the target‐decoy database approach, was set at 0.01 for both peptides and proteins. MS/MS spectra of peptides were next used to create the spectral library for SWATH peak extraction using the PeakView software (version 2.1; Sciex) using MS/MSALL with SWATH Acquisition MicroApp (version 2.0, Sciex). Peptides with greater than 99% confidence interval were added to the spectral library.

### 2.5 Relative quantification by SWATH acquisition

Forty‐seven samples of colonic tissue (40 tumours and 7 healthy tissue) were evaluated using an independent data acquisition (DIA) method. Samples were analysed by LC‐MS as described above to construct the spectral library but using a SWATH‐MS acquisition method. SWATH method comprised a TOF MS (350‐1250 m/z, acquisition time 50 ms) followed by 50 windows of variable size (230‐1500 m/z, with acquisition time of 90 ms) with a minimum size of 5 m/z. SWATH variable window calculator from Sciex was used to adjust the window width of these variables to ion density.

### 2.6 SWATH‐MS data analysis

Data extraction from SWATH runs was carried out by PeakView using MS/MSALL with SWATH Acquisition MicroApp, resulting in a library containing 2915 proteins. Peptide retention times for each protein were realigned in each run according to indexed retention time (iRT) peptides (Biognosys AG, Schlieren/Zürich, Switzerland). Chromatograms of the extracted ions were created for each selected ionic fragment. PeakView calculated a score and FDR for each assigned peptide using chromatographic and spectral components. MarkerView (version 1.2.1; Sciex) allowed signal normalization, and a *t* test was applied for testing differential abundance.

### 2.7 Gene expression–based classification into colon cancer subtypes

Total RNA was extracted from samples using RNeasy Mini Kit (Quiagen) following the manufacturer's recommendations. We performed nCounter Element system by NanoString to analyse the RNA expression of a set of genes and classify CRC samples according to Sadanandam et al 2013 4 (5 subtypes) and De Sousa E Melo et al 2013 5 (3 subtypes). For these classifications, we used the expression of those genes included in qPCR mini‐classifiers described by Sadanandam et al 4 (7 genes) and by De Sousa E Melo et al 5 (8 genes). ZEB1 gene is included in both mini‐classifiers (Table S2). The nSolver software (NanoString Technologies) was used for data analysis. Complement and immune‐related gene expression in tumour subtypes was analysed by using the nCounter^®^ PanCancer Immune Profiling Panel (NanoString Technologies, Inc).

### 2.8 Western blot analysis

Proteins (20 μg) were separated on a Criterion^™^ TGX Stain‐Free 4%‐20% acrylamide gel in the Bio‐Rad Criterion System and transferred to PVDF membranes (Bio‐Rad Laboratories). After blocking with 5% non‐fat milk, membranes were incubated with the specific primary antibodies followed by incubation with secondary antibody conjugated with horseradish peroxidase and detection was performed with chemiluminescent reaction with the Clarity Western ECL Substrate (Bio‐Rad). Images were captured on a ChemiDoc XRS Imaging System (Bio‐Rad). Stain‐free technology was used as protein loading control, and densitometric analysis was performed with Image Lab software (Bio‐Rad). Antibodies used were as follows: SPP1 (OPN), HDAC2, SERPINA1 and RPS27L from Sigma‐Aldrich, and SFRS3 from Abcam. Secondary antibodies conjugated with HRP were from Santa Cruz Biotechnology.

#### 2.8.1 Immunohistochemical analysis

Immunohistochemical (IHC) staining was performed incubating 4 µm FFPE sections in 10 mmol/L citrate buffer (pH 6.0) at 120°C for 5 minutes for antigen retrieval. Endogenous peroxidase was neutralized using EnVision FLEX Peroxidase‐Blocking Reagent (Dako) for 10 minutes. Tissue sections were blocked with 3% bovine serum albumin and incubated with the primary antibodies overnight at 4°C. Primary antibodies used were FRMD6 (Sigma‐Aldrich), ZEB1 (Sigma‐Aldrich), HTR2B (Sigma‐Aldrich), AE1AE3 (Thermo Scientific), CDX2 (Novus Biologicals), SRSF3 (Abcam) and SERPINA1 (Sigma‐Aldrich). After incubation with the EnVision FLEX + mouse or rabbit linker (Dako), EnVision FLEX/HRP (Dako) was used as the secondary antibody for 1 hour at room temperature, followed by 3,3'‐diaminobenzidine (DAB) staining (Dako). CMS molecular classification was performed according to Thrin et al,^14^ analysing the intensity and content of FRMD6, ZEB1, HTR2B, AE1AE3 and CDX2. SRSF3 and SERPINA1 tumoral epithelial expression was categorized as high (intense staining) and low expression (moderate and negative staining). Individual cores were scored by trained pathologists (CVP and SGL).

### 2.9 Bioinformatic analysis

MetaboAnalyst 3.0 software was used for the generation of heatmaps and sample classification according to an unsupervised analysis. The generation of the hierarchical clustering of proteins was performed to establish differences between protein profiles expressed in healthy tissue compared to tumours and to discern those patterns of protein expression that establish a classification in different molecular subtypes of CRC alternative based on gene signatures. We performed an integrated molecular pathway analysis using Gene ontology (GO) terms based on the Kyoto Encyclopedia of Genes and Genomes (KEGG) database (http://www.genome.jp/kegg/) in order to classify the proteins identified. Furthermore, iPathway Guide (Advaita Bioinformatics) was consulted for individual protein analyses, their ontology (biological processes, molecular functions and cellular components) and molecular pathways. The statistical values provided by Student's *t* test were corrected through the Benjamini and Hochberg procedure, when comparing the GO terms of Ontology Genes proteins involved. Then, a cut‐off (*P*‐value < .05) was used to select the significant biological/processes pathways genes/proteins. STRING database (http://string-db.org) was also used to assess protein‐protein interactions.

### 2.10 Statistical analysis

First, relative abundance values of proteins from each sample were subjected to Student's *t* test (*P*‐value < .05), assuming equal variances. Then, the fold change of differential expression values was calculated for each of the comparisons. In order to be more strict, due to the large number of proteins obtained by MS, a Volcano diagram was used to select those proteins that were significant (*P*‐value < .05) and no <2 fold change. Fisher´s exact test was used to compare the association between proteomic subtypes and CCS classification. Protein expression by immunoblot and gene expression statistical tests was carried out using GraphPad Prism 5. Statistical differences were assessed by a parametric approach (Student's *t* test, in the case of equal variances) or nonparametric method (Mann‐Whitney test) according to normality, assessed by Shapiro‐Wilk test. Differences were considered statistically significant at *P* < .05.

## 3 RESULTS

### 3.1 SWATH‐based proteomic analysis

A total of 40 human adenocarcinoma samples (see Table S1 for Clinical and pathological characteristics) and 7 samples of healthy tissue were analysed. Samples of colonic tissue were grouped in 9 pools and subjected to the shotgun proteomic analysis to construct the spectral library, as described in the Materials and Methods section. As a result, after integrating all nine data sets, 3080 proteins were identified (Table S4), with a FDR < 1% for both protein and peptide levels. We quantified 2752 proteins across all 47 samples in the SWATH‐MS analysis, with a FDR threshold of 5%. Table S5 lists the quantification values for these 2752 proteins.

### 3.2 Differentially expressed proteins in SWATH‐MS completely discriminate between CRC tissues and the normal tissues

**Figure 1:**
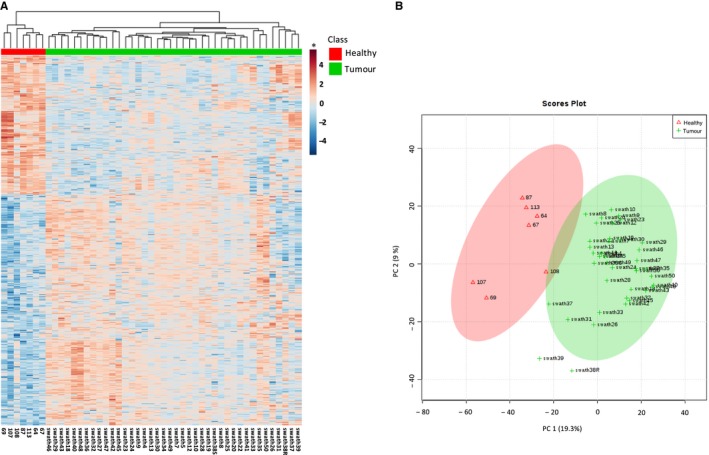
Analysis of differentially expressed proteins in SWATH‐MS. (A) Heatmap showing hierarchical clustering between controls and CRC using differentially expressed proteins (n = 2752). The relative protein abundance values for each sample were transformed on a logarithmic scale (log_2_), normalized and grouped using a strategy based on the Euclidean distance with the criterion of minimum variance or Ward method. (B) Principal component analysis showing separation of control and CRC samples. * In the heatmap, red and blue colours represent higher and lower relative abundance of proteins, respectively

As shown in Figure 1, protein expression profiles revealed by SWATH‐MS completely separate CRC tissues from the normal tissues. The unsupervised hierarchical clustering analysis demonstrated a clear discrimination between these two groups of samples with different protein expression patterns (Figure 1A). Moreover, principal component analysis (PCA), which is another unsupervised method, confirmed that tumour and healthy tissues were clearly distinguished using the quantitative protein expression data obtained by SWATH‐MS (Figure 1B).

### 3.3 SWATH‐MS analysis identifies three molecular subgroups of CRC

**Figure 2:**
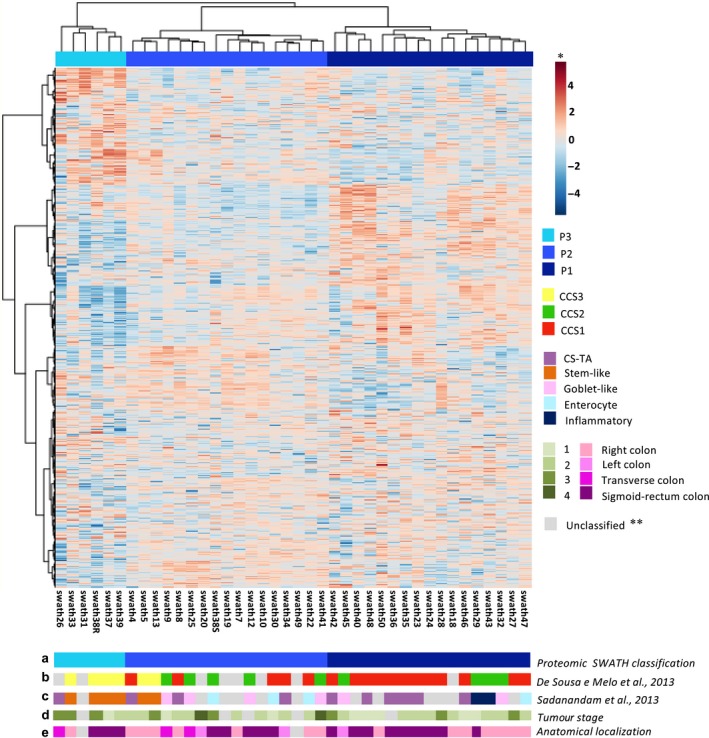
Unsupervised clustering analysis of SWATH‐MS data from tumoral samples. Heatmap showing hierarchical clustering of CRC samples (n = 40). Relative protein abundance values for each sample were transformed on a logarithmic scale (log_2_), normalized and grouped using a strategy based on the Euclidean distance with the criterion of minimum variance or the Ward method. At the bottom of the heatmap are shown: (a) the proteomic classification of samples, (b) the gene expression–based classification of samples according to De Sousa e Melo et al (2013) and (c) Sadanandam et al (2013), as well as clinical data of CRC samples, including tumour stage (d) and anatomical localization of tumour (e). * In the heatmap, red and blue colours represent higher and lower relative abundance of proteins, respectively. **Grey colour indicates those samples that could not be classified in any of the subgroups or categories

The unsupervised clustering analysis of SWATH‐MS data from tumour samples also revealed that 3 subgroups of CRC, denominated P1, P2 and P3, could be differentiated (Figure 2). These proteomic subtypes were then compared with diverse classifications based on gene expression signatures to characterize different molecular subtypes of CRC. For this purpose, we first performed an RNA expression analysis in the 40 tumour samples of different gene classifiers (Table S3). This allowed the supervised classification of tumours into five distinct subtypes: transit‐amplifying (TA), enterocyte, goblet‐like, inflammatory and stem cell‐like tumours.^4^ Using the same approach, tumours were also classified into the CCS1, CCS2 and CCS3 molecular subtypes described by De Sousa e Melo et al.^5^

Notably, there was a high concordance between the subgroups identified by the proteomic analysis and classifications based on gene expression signatures, particularity according to De Sousa e Melo et al (*P* < .001) (Figure 2). Thus, P1 subgroup included a majority of tumour samples classified as CCS1 subtype,^5^ characterized by KRAS and/or TP53 mutations and corresponding to the group of chromosomal‐instable (CIN) tumours. This proteomic subgroup also included a significant number of transit‐amplifying (TA) tumours,^4^ which is a subgroup that has been reported as a subset of the CCS1 subtype.^6^ The P2 subgroup showed a more heterogeneous pattern but contained a majority of samples from the CCS2 subgroup, which is related to microsatellite instability (MSI) 5 and also most of goblet‐like tumours,^4^ that has been reported as a subset of the CCS2 subtype.^6^ Finally, a third proteomic subtype (P3) was clearly differentiated from both P1 and P2 subtypes. Importantly, this subtype included a majority of CCS3 tumours 5 that also were classified as stem‐like tumours according to the classification of Sadanandam et al^4^ This stem‐like/mesenchymal CRC subtype is a distinct set of highly aggressive CRC tumours associated with poor patient outcome. Remarkably, this P3/mesenchymal/stem‐like subgroup was associated with a significant lower 3‐year overall survival rate when compared with P1 or P2 subgroups (Figure S1A). However, this association was not observed comparing gene expression classifiers (Figure S1B and S1C) adding value for risk stratification.

### 3.4 The P3/mesenchymal/stem‐like subgroup shows a distinct protein expression pattern compared to the other proteomic CRC subtypes

**Figure 3:**
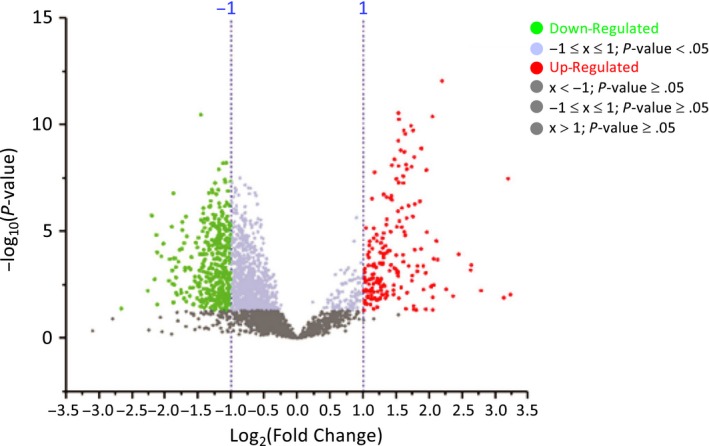
Volcano diagram of proteins with significant differential expression comparing P3 with the rest of proteomic subtypes in CRC. Volcano diagram resulted from comparison of subtypes P3 vs P1 and P2. Proteins are separated according to the log_2_ of the fold change (x‐axis) and the ‐log_10_ of the *P*‐values based on a two‐tailed *t* test (y‐axis). A total of 186 proteins (green dots) were found with increased expression in the P3 subtype, compared to P1 and P2, and 379 proteins (red dots) with decreased expression (*P*‐value <.05; FC ≥ 2 or FC ≤ 0.5)

Proteomic expression profile of the mesenchymal/stem‐like (P3) subgroup was analysed and compared with the rest of subgroups (P1 and P2). Due to the large number of proteins obtained by MS, a Volcano diagram was first made to select those proteins with statistically significant differential expression and no less than 2 fold change (Figure 3). As a result, 186 proteins were found in the P3 subtype with increased expression, compared to P1 and P2, and 379 proteins with decreased expression.

**Figure 4:**
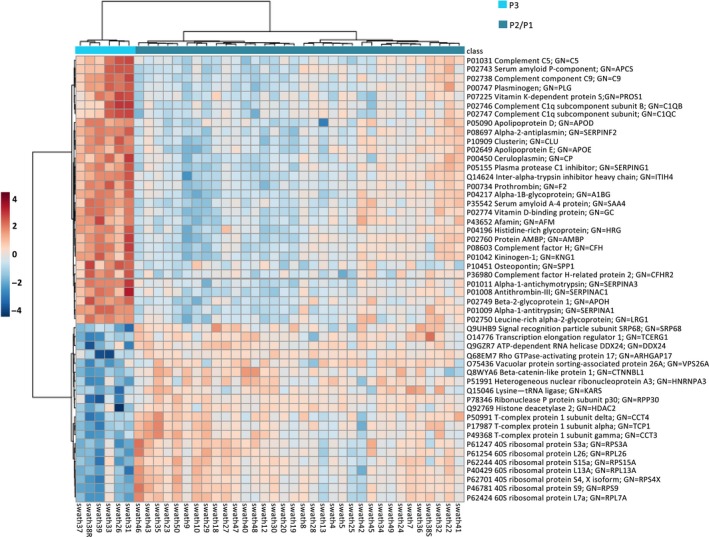
Top 50 proteins showing the greatest expression differences between P3 and both P1 and P2 subtypes in CRC. Heatmap of ‘top 50’ proteins with greatest magnitude of change (fold change) when comparing P3 with the rest of subtypes. Of these top 50 proteins, 30 were up‐regulated in the P3 subtype, whereas 20 were down‐regulated, compared with both P1 and P2 subtypes. In the heatmap, red and blue colours represent higher and lower relative abundance of proteins, respectively

In order to analyse most significant proteins in reference to their expression, we decided to consider the top 50 proteins showing the largest expression differences between P3 and both P1 and P2 subtypes (Figure 4). As it can be observed, there was a clearly different protein expression pattern comparing the P3 subtype with the rest of subtypes. Of these top 50 proteins, 30 were clearly up‐regulated in the P3 subtype, whereas 20 were markedly down‐regulated, compared with both P1 and P2 subtypes (Table 1).

**Table 1:** Top 50 proteins showing the greatest expression differences between P3 and the rest of subtypes in colorectal cancer

UniProt accession code	Protein name	*P*‐value	Fold Change
P10451	Osteopontin GN = SPP1	3.35E‐08	9.17
P01009	Alpha‐1‐antitrypsin GN = SERPINA1	8.57E‐13	4.57
P01011	Alpha‐1‐antichymotrypsin GN = SERPINA3	4.09E‐11	4.13
P00747	Plasminogen GN = PLG	1.33E‐08	3.88
P02748	Complement component C9 GN = C9	1.27E‐09	3.68
P02743	Serum amyloid P‐component GN = APCS	3.81E‐07	3.62
P07225	Vitamin K‐dependent protein S GN = PROS1	5.22E‐07	3.44
P02750	Leucine‐rich alpha‐2‐glycoprotein GN = LRG1	3.31E‐09	3.42
P02749	Beta‐2‐glycoprotein 1 GN = APOH	1.79E‐10	3.38
P02747	Complement C1q subcomponent subunit C GN = C1QC	1.96E‐06	3.37
P35542	Serum amyloid A‐4 protein GN = SAA4	1.11E‐10	3.30
P10909	Clusterin GN = CLU	1.22E‐08	3.16
P04217	Alpha‐1B‐glycoprotein GN = A1BG	2.62E‐10	3.10
P01042	Kininogen‐1 GN = KNG1	1.86E‐09	3.08
P05090	Apolipoprotein D GN = APOD	1.83E‐10	3.06
P36980	Complement factor H‐related protein 2 GN = CFHR2	2.52E‐08	3.04
P02746	Complement C1q subcomponent subunit B GN = C1QB	6.19E‐06	2.99
P02774	Vitamin D‐binding protein GN = GC	5.54E‐11	2.90
P02649	Apolipoprotein E GN = APOE	5.05E‐08	2.89
P08697	Alpha‐2‐antiplasmin GN = SERPINF2	2.83E‐11	2.89
P00734	Prothrombin GN = F2	5.50E‐10	2.89
P00450	Ceruloplasmin GN = CP	1.12E‐06	2.88
P43652	Afamin GN = AFM	3.40E‐08	2.83
P08603	Complement factor H GN = CFH	4.15E‐09	2.78
Q14624	Inter‐alpha‐trypsin inhibitor heavy chain H4 GN = ITIH4	7.84E‐09	2.70
P02760	Protein AMBP GN = AMBP	2.12E‐07	2.68
P05155	Plasma protease C1 inhibitor GN = SERPING1	2.87E‐07	2.66
P01008	Antithrombin‐III GN = SERPINC1	2.48E‐07	2.56
P01031	Complement C5 GN = C5	2.64E‐06	2.56
P04196	Histidine‐rich glycoprotein GN = HRG	1.68E‐08	2.25
Q9UHB9	Signal recognition particle subunit SRP68 GN = SRP68	5.93E‐09	0.47
P50991	T‐complex protein 1 subunit delta GN = CCT4	3.64E‐08	0.47
O75436	Vacuolar protein sorting‐associated protein 26A GN = VPS26A	6.15E‐09	0.46
O14776	Transcription elongation regulator 1 GN = TCERG1	1.23E‐08	0.44
P49368	T‐complex protein 1 subunit gamma GN = CCT3	1.13E‐07	0.44
Q15046	Lysine‐tRNA ligase GN = KARS	1.75E‐07	0.43
P17987	T‐complex protein 1 subunit alpha GN = TCP1	5.29E‐08	0.42
P46781	40S ribosomal protein S9 GN = RPS9	1.53E‐07	0.41
P51991	Heterogeneous nuclear ribonucleoprotein A3 GN = HNRNPA3	1.12E‐07	0.41
Q8WYA6	Beta‐catenin‐like protein 1 GN = CTNNBL1	3.67E‐07	0.40
P61247	40S ribosomal protein S3a GN = RPS3A	6.90E‐07	0.40
P62701	40S ribosomal protein S4. X isoform GN = RPS4X	1.55E‐07	0.40
P61254	60S ribosomal protein L26 GN = RPL26	2.81E‐06	0.39
P62424	60S ribosomal protein L7a GN = RPL7A	2.42E‐06	0.39
P62244	40S ribosomal protein S15a GN = RPS15A	2.53E‐06	0.38
P40429	60S ribosomal protein L13a GN = RPL13A	8.05E‐07	0.37
Q68EM7	Rho GTPase‐activating protein 17 GN = ARHGAP17	3.39E‐11	0.36
Q9GZR7	ATP‐dependent RNA helicase DDX24 GN = DDX24	1.99E‐06	0.31
P78346	Ribonuclease P protein subunit p30 GN = RPP30	1.61E‐07	0.27
Q92769	Histone deacetylase 2 GN = HDAC2	1.78E‐06	0.22

### 3.5 Biological processes and pathways altered in the P3/mesenchymal/stem‐like subtype of CRC

Gene Ontology enrichment analysis was carried out (Figure S2). As a result of this comparison of P3 subtype with both P1 and P2 subtypes, we found key biological processes, molecular functions, cellular components such as ribosome and molecular pathways related to spliceosome among others (Figure S2).

**Figure 5:**
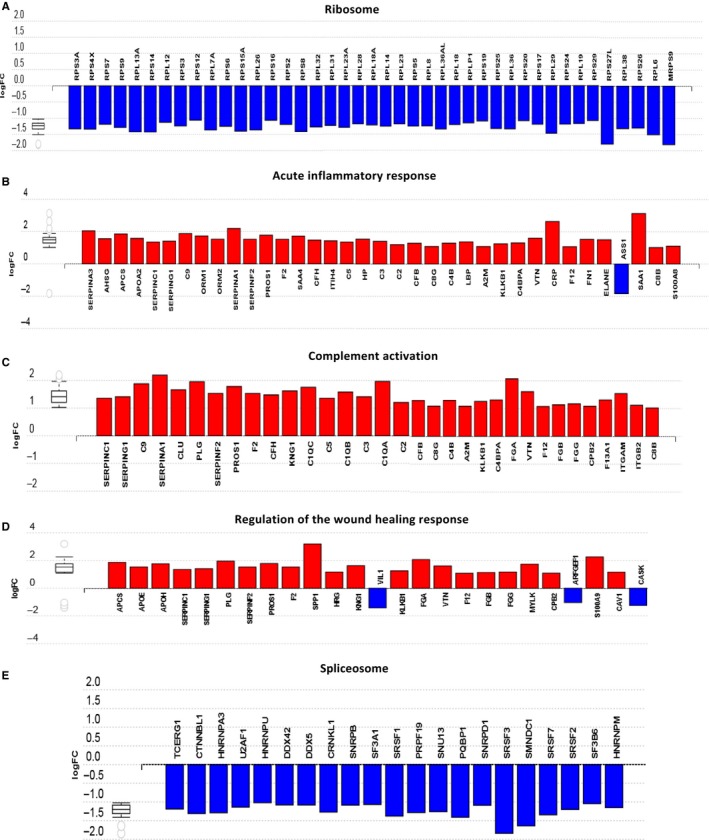
Biological processes and pathways altered in P3 tumours. Bar charts showing the expression, based on log_2_FC (Fold Change), of proteins related to: (A) ribosomal structure and biogenesis, (B) acute inflammatory response, (C) complement activation, (D) regulation of the response to wound healing and (E) spliceosome. The increase and decrease in the expression of the proteins in the P3 subtype, compared to the subtypes P1 and P2, are indicated in red and blue, respectively. Note: Because of the large number of ribosome‐associated proteins, not all of them have been included in the above diagram, but they exhibited a similar downtrend in expression in the P3 subtype

Using the iPathwayGuide software (Advaita Bioinformatics) and applying. the correction of Benjamini and Hochberg, the proteins were grouped according to the process, pathway or function exerted. First, comparing the P3/mesenchymal/stem‐like subtype with the rest of subgroups, a decrease in the proteins involved in the structure and ribosomal biogenesis, such as RPL7A, RPS9, RPS4X, RPL13A, RPS15A, RPL26 and RPS3A, was observed (Figure 5A). P3 tumours also showed an increase in the expression of proteins related to the acute inflammatory response, such as C‐reactive protein (CRP) or protein serum amyloid A‐1 (SAA1) (Figure 5B), as well as increased expression of proteins related to the complement activation pathway, such as the complement (C9), plasminogen (PLG), subunit A, B and C of component C1q of complement (C1QA, C1QB and C1QC), and fibrinogen α chain (FGA) (Figure 5C). Notably, the P3 subtype also showed higher levels of proteins involved in the regulation of wound healing response (Figure 5D), mainly osteopontin (SPP1; OPN), S100A9 protein, plasminogen (PLG) and vitronectin (VTN). Finally, as shown in Figure 5E, P3 tumours showed a marked decrease in the expression of proteins related to the spliceosome, such as the serine/arginine‐rich splicing factor 3 (SRSF3), survival of motor neuron‐related‐splicing factor 30 (SMNDC1), serine/arginine‐rich splicing factor 1 (SRSF1) and crooked neck‐like protein 1 (CRNKL1). Protein interaction networks of the proteins of interest were also analysed. As shown in Figure S3, different clusters of biological processes and pathways were identified, which specifically included ribosome and spliceosome proteins (Figure S3A), regulation of wound response, acute inflammatory response, wound healing and coagulation and complement cascade (Figure S3B).

**Figure 6:**
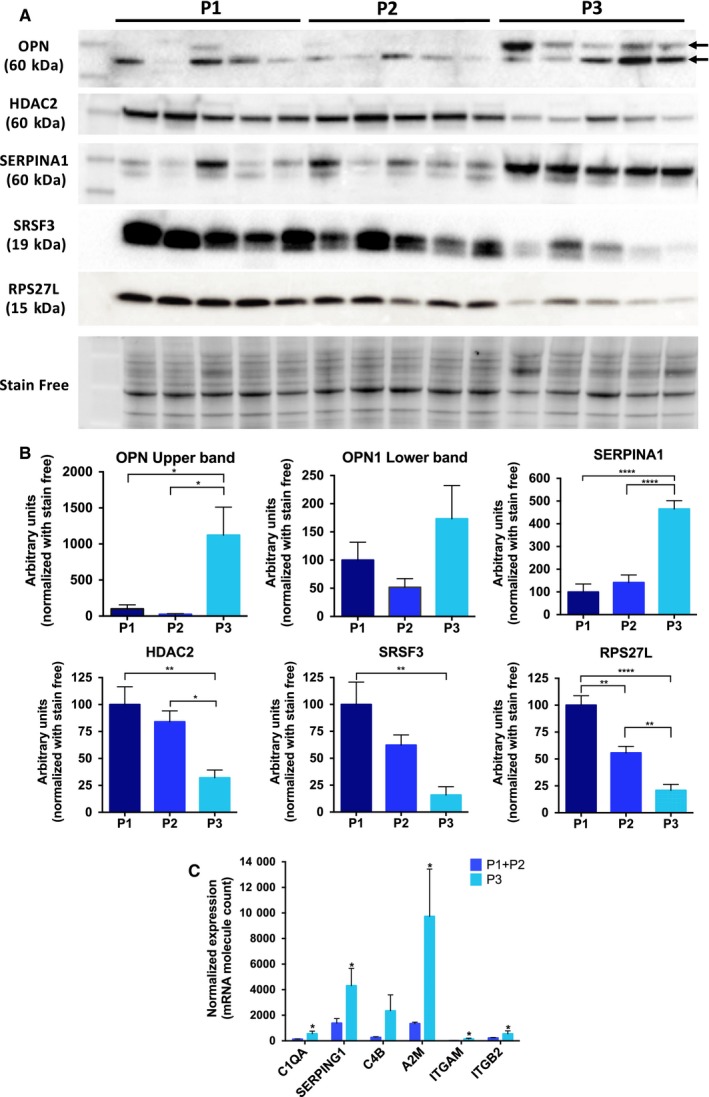
Western blot and gene expression analyses of several proteins of interest. (A) Immunoblot analyses confirmed in P3 tumours the up‐regulation of OPN and SERPINA 1, whereas the expression of HDAC2, SRSF 3 (spliceosome) and RPS27L (ribosome) was down‐regulated in this type of tumours. (B) Corresponding densitometric analyses of protein bands detected in the immunoblots and normalized to stain‐free signal as loading control. Error bars for each group indicate means ± SEM (n = 5), *P ≤ .05, **P ≤ .01, ***P ≤ .001, ****P ≤ .0001. (C) mRNA expression of genes related to complement pathway activation (C1QA, SERPING1, C4B, A2M, ITGAM and ITGB2) comparing P3 with the rest of subtypes. mRNA molecule counts were performed with the nCounter^®^ PanCancer Immune Profiling Panel. Values represent mean ± SEM (n = 5 of each subtype) of the normalized expression of each mRNA. Statistical comparisons were performed with the Mann‐Whitney test. *P ≤ .05

Western blot and gene expression analyses of several proteins of interest were then performed to validate the above results. As shown in Figure 6, the immunoblot analyses confirmed in P3 tumours the up‐regulation of OPN and SERPINA 1, whereas the expression of HDAC2, SRSF3 (spliceosome) and RPS27L (ribosome) was down‐regulated in this type of tumours. Human osteopontin is subject to alternative splicing, and the molecular size of this protein is known to be variable ranging between 41 and 75 kD.^15^ As illustrated in Figure 6, there was 10‐fold increase in the detection of the upper osteopontin protein band at around 65 kD in the P3 tumours compared with P1 and P2 subtypes. These results are consistent with the marked decrease in the expression of spliceosome proteins in P3 tumours. On the other hand, gene expression analyses confirmed that proteins related to the complement activation, such as C1QA, SERPING1, A2M, ITGAM and ITGB2, were significantly overexpressed in P3 tumours, compared to P1 and P2 subtypes (Figure 6C). Finally, to explore whether there is also an association between our proteomic subtypes and CMS subtyping,^16^ we made a retrospective analysis of 45 FFPE CRC tumour samples (Table S2) and, after their classification into CMS subtypes by IHC as described by Trinh et al,^14^ the expression of SERPINA1 and SRSF3 was evaluated by IHC in each of CMS1, CMS2/3 and CMS4 subtypes (Figure S4). As expected, these analyses confirmed in CMS4 subtype the higher expression of SERPINA 1, whereas the expression of SRSF3 (spliceosome) was down‐regulated in this type of tumours. Therefore, the analysis of this additional cohort of tumour samples also indicated the association between proteomic subtyping and CMS classification. The above results validate the proteomic analysis and suggest novel biomarkers that can be useful tools for the molecular classification of CRC and also for the development of new therapeutic strategies in the mesenchymal CRC subtype of worse prognosis. Furthermore, our analyses demonstrate the added value of our proteomic data set, relative to published gene expression data sets generated for CRC.

## 4 DISCUSSION

In this study, the proteomic analysis of 47 samples of colorectal tissue allowed, first, to distinguish tumours from samples of healthy tissue used as a control and, second, to establish a classification of tumours based on their protein expression profiles. Our SWATH‐MS data from tumour samples revealed 3 subgroups of CRC, denominated P1, P2 and P3. The P1 subtype was highly similar to CCS1 and TA subtypes that have been associated with an epithelial gene signature and better prognosis.^6^ On the other hand, the P2 subgroup was more heterogeneous in term of molecular characteristics, and included tumours from the CCS2 subgroup and also most of goblet‐like tumours, suggesting that this proteomic subgroup may comprise MSI CRC tumours.^6^ Importantly, the unsupervised classification based on the proteomic profiles confirmed in our cohort a subgroup of mesenchymal tumours (P3)/stem‐like, equivalent to that characterized by the expression of mesenchymal and stem genes, which is also associated with a poor prognosis and low patient survival.^16^ Importantly, a retrospective analysis of an additional cohort of FFPE CRC tumours also indicated the association between our proteomic subtyping and CMS classification, and validated the differential expression of our proteomic biomarkers in CMS subtypes, with CMS4 tumours showing a higher expression of SERPINA 1 and a down‐regulation of SRSF3 (spliceosome). This subtype represents a particularly aggressive type of CRC,^8^ so it is important to identify in these tumours molecules and signalling pathways associated with processes of metastasis and relapse that can be approached therapeutically, as well as biomarkers that facilitate an early diagnosis of the disease.

Different proteomic technologies have been used to find new cancer biomarkers for CRC employed for risk prediction, diagnosis, prognosis, staging and monitoring treatment response of this disease. First proteomic studies focused on two‐dimensional electrophoresis (2‐DE) to characterize diseased vs control proteomes in combination with MALDI‐TOF MS 17, 18 or LC‐MS/MS.^19^, ^20^, ^21^ Nevertheless, these proteomic approaches involve hydrophobicity, size and solubility limitations of protein samples,^22^ and label‐free LC‐MS technologies have been used as an alternative approach in the search of CRC biomarkers.^21^ Nowadays, SWATH technique provides comprehensive and permanent records of all detectable molecular species and this proteomic technology is emerging as a powerful tool for CRC research. Thus, SWATH‐MS–based quantitative proteomic analysis has been used to analyse glycoproteins secreted by a colon adenocarcinoma cell line that were confirmed by immunodetection 23 or to determinate the molecular effects induced by DDHD1 silencing in colorectal cancer cells.^24^ Therefore, SWATH‐MS studies are particularly useful for better proteome characterization and may accelerate future discovery and validation of CRC biomarkers.

Tumours classified as P3/mesenchymal/stem‐like were found to have a very different pattern of protein expression compared to the other CRC tumours. Thus, P3 tumours were characterized by a decrease in proteins involved in ribosomal structure and biogenesis, in addition to a decrease in proteins involved in the spliceosome. Recent studies indicate that ribosome‐independent functions may be involved in various physiological and pathological processes, including tumorigenesis or tumour suppression.^25^, ^26^, ^27^ In this study, we observed in P3 tumours a decreased expression of ribosomal proteins involved in tumour suppression, such as RPL6,^27^ RPL23,^28^, ^29^ RPL26,^30^ RPS3,^31^ RPS14,^32^ RPS15 and RPS20,^33^ RPS25,^34^ RPS26 35 and RPS27L.^36^ In addition, elevated RPS27L expression in tumour has been related to a better prognosis in CRC patients.^37^ Interestingly, RPL13A has been identified as a negative regulator of inflammatory proteins, suggesting that this ribosomal protein could be a repressor of inflammatory signalling.^38^ Inflammatory response plays an essential role during tumorigenesis, and prolonged expression of inflammatory genes promotes tumour progression. Therefore, and in agreement with the tumour suppressive function of ribosomal proteins, RPL13A not only protects host tissues from inflammatory injury, but also prevents cancerous growth of the inflamed cells.^38^ Accordingly, in our study P3 tumours showed a decreased expression of RPL13A and an increased expression of proteins related to the acute inflammatory response.

Splicing process is commonly deregulated in cancer, resulting in non‐functional end products.^39^ The results of the present study indicate that the P3/mesenchymal/stem‐like subtype of CRC is characterized by an overall decrease in the expression of spliceosomal proteins. Notably, studies on differential splicing events among tumours support transcriptome instability as a molecular characteristic of CRC.^40^, ^41^ Furthermore, a strong inverse correlation was found between transcriptome instability and the expression of splicing factor genes, which was also associated with poor patient survival.^21^, ^40^, ^41^

Our results also indicated in P3 tumours an increased expression of proteins related to acute inflammatory response, wound healing response and complement pathway activation. Zhang et al^42^ identified five proteomic subtypes in genomically annotated TCGA colon tumours and reported that the proteomic subtype with stem‐like characteristics was enriched in genes involved in wounding response. On the other hand, it is known that most non‐tumour cells in the tumour microenvironment are immune cells. Therefore, the distinct expressions of proteins related to the immune system detected in our study in the tumour are very likely due to the interaction of tumour and the immune system, including immune infiltration in the tumour microenvironment.

Wound healing and cancer relationship have long been studied, and inflammatory processes that occur during normal wound healing have been linked to tumorigenesis, tumour progression and metastasis in many different cancers.^43^, ^44^ On the other hand, proinflammatory factors, which are known regulators of normal adult stem cells during tissue repair, also promote survival and proliferation of cancer stem cells.^44^

Wound healing and inflammation in tumour biology are also associated with complement activation.^45^ The complement system is a central part of the innate and adaptive immune response and has traditionally been considered part of the body's immunosurveillance against cancer. However, accumulating evidence supports a tumour‐promoting role of complement activation within the tumour microenvironment by perpetuating local T‐cell immunosuppression and chronic inflammation facilitating tumour immune escape, outgrowth and metastasis.^46^ Significantly, the mesenchymal/stem‐like subtype defined by gene expression signatures is also characterized by a marked overexpression of genes involved in complement‐related signalling.^16^, ^47^, ^48^ Our results also support that novel immunotherapeutic approaches, including inhibition of complement regulators, must be explored in inflamed P3/mesenchymal/stem‐like tumours.

Osteopontin (OPN; SPP1) and two proteins of the serpin family, such as SERPINA1 and SERPINA3, were those with higher expression in the P3 subtype compared to the rest of tumours. OPN is a versatile phosphoprotein, secreted by different types of cells, including lymphocytes, macrophages and osteoclasts.^49^ Numerous studies have revealed the roles that OPN plays in tumour biology, participating in inflammation, tumour progression and metastasis.^50^ Recent studies have demonstrated overexpression of OPN in many human carcinomas, including lung, breast and gastric cancer, hepatocellular carcinoma and colorectal cancer.^51^ Notably, OPN appears to play a main role in the mechanisms deployed by tumours to evade immune recognition by participating in the crosstalk between cancer cells and the host microenvironment. Furthermore, the human OPN transcript is subject to alternative splicing, and the expression patterns of splicing factors dictate the major OPN splicing isoform in a specific pathological condition.^15^ In this regard, the altered expression of immunodetected OPN protein bands in P3 tumours may be related to the altered expression of spliceosome proteins in these tumours. Therefore, the results of the present study support the hypothesis that an altered OPN expression in P3/mesenchymal/stem‐like subtype CRC could promote invasion and metastasis, being responsible for the poor prognosis and low survival in these patients.

On the other hand, serpins play a key role in the maintenance of cellular homeostasis. They are known to be irreversible suicide inhibitors of proteases, but they can also participate in critical proteolytic pathways such as blood coagulation (SERPINA1, SERPINA5, SERPINA8, SERPINA10), tissue remodelling (SERPINA1, SERPINA3), angiogenesis (SERPINAC1), inflammation, apoptosis and tumour metastasis (SERPINA1, SERPINA3, SERPINA4, SERPINAC1).^52^, ^53^ High levels of SERPINA1 are associated with inflammatory bowel disease and CRC progression.^54^ These studies reinforce the notion that SERPINA1 is associated with tumour invasion and could be a useful protein marker for CRC diagnosis. In addition, this protein is related to tumour aggressiveness, local spread and capacity to produce metastases.^55^

P3 subtype was further characterized by a marked decrease in the expression of the histone deacetylase 2 protein (HDAC2), compared to P1 and P2 subtypes. HDACs play an important role in epigenetic regulation of transcription by removing the acetyl group from histones and promoting chromatin compaction.^56^ Significantly, recent research indicates that HDAC inhibitors are capable of inducing EMT in colon carcinoma cells.^57^ Furthermore, HDAC inhibitors exert immune suppressive effects.^58^ Although HDACs repress gene transcription by deacetylating lysine residues of histone proteins, they also remove acetyl groups from nonhistone proteins and modulate their activity.^59^ The down‐regulated levels HDAC2 expression in P3 CRC subtype may therefore contribute to the immunosuppressive mechanisms deployed by these tumours.

In summary, SWATH technology allows distinguishing different molecular subtypes of CRC. Significantly, differential protein expression has allowed to identify a subgroup of tumours similar to the mesenchymal/stem‐like subtype defined by gene expression signatures. Our results show that these tumours are characterized by alterations in the expression of proteins involved in processes and signalling pathways that are key determinants in the crosstalk between cancer cells and tumour microenvironment, modulating immune evasion and the metastasis process. This proteomic analysis hence suggests new therapeutic targets for the treatment of this particularly aggressive type of CRC.

## CONFLICT OF INTERESTS

The authors declare no conflict of interests.

## AUTHOR CONTRIBUTIONS

EA and A.R‐A. conceived the study. LM. L‐S. and RJ‐I. performed experiments, performed bioinformatic analyses and prepared figures and tables. JP, R.M and SG performed gene expression analyses. MT and FC processed clinical samples. CV and CD contributed to the pathological analysis of samples. IO performed proteomic analysis. JR. H‐R. and EA contributed to the clinical information of samples. LM. L‐S., RJ‐I, EA and A.R‐A wrote the manuscript text. All authors reviewed the manuscript.

## Data Availability

MS data have been deposited to the ProteomeXchange Consortium via the PRIDE partner repository (https://www.ebi.ac.uk/pride/archive/) with the data set identifier PXD007810. Other data that support the findings of this study are available from the corresponding author upon reasonable request.
