# Supplementary figures and images for: SWATH‐based proteomics reveals processes associated with immune evasion and metastasis in poor prognosis colorectal tumours
Source: J Cell Mol Med. 2019 Sep 27;23(12):8219–32. doi: 10.1111/jcmm.14693 (PMC6850959; doi:10.1111/jcmm.14693)

Figure S1

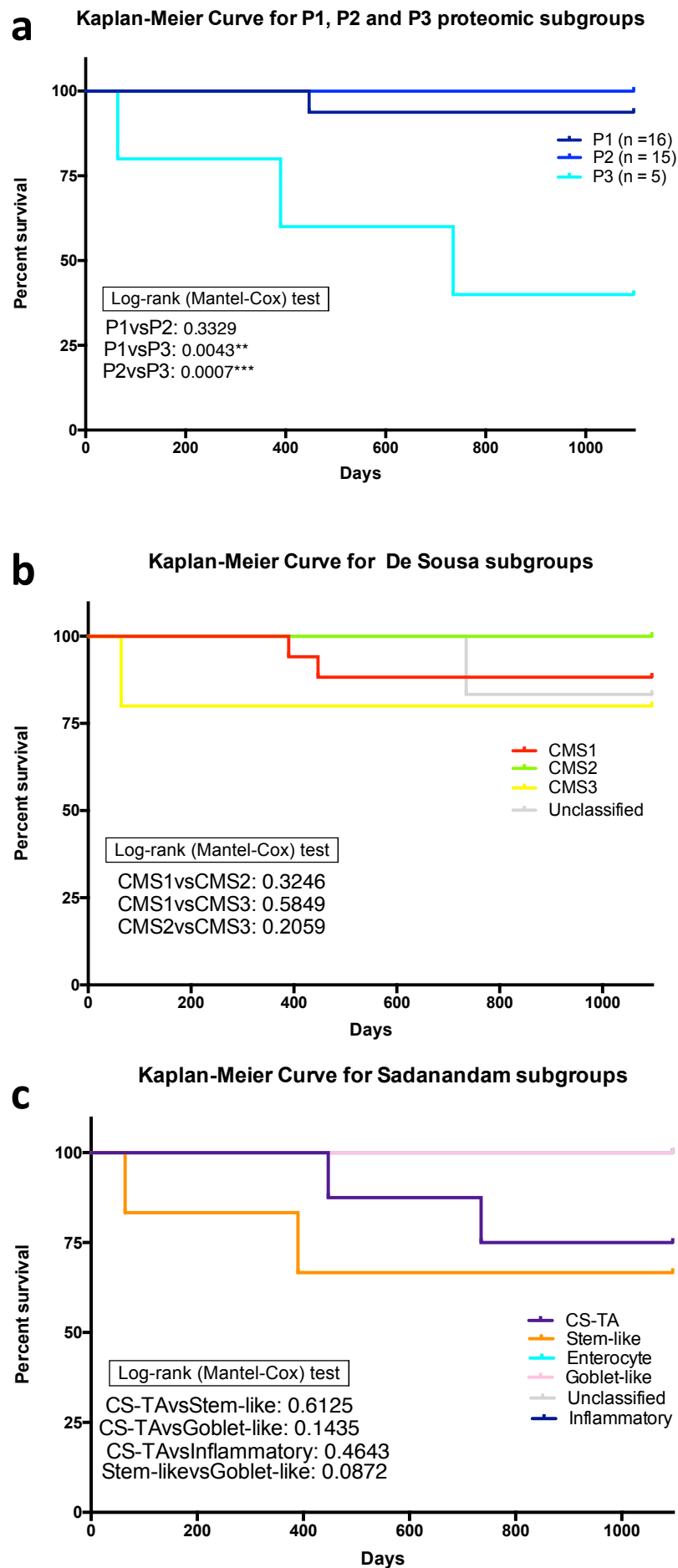

Supplement: Supplementary file 1 [file JCMM-23-8219-s001.pdf]

**Figure S2**

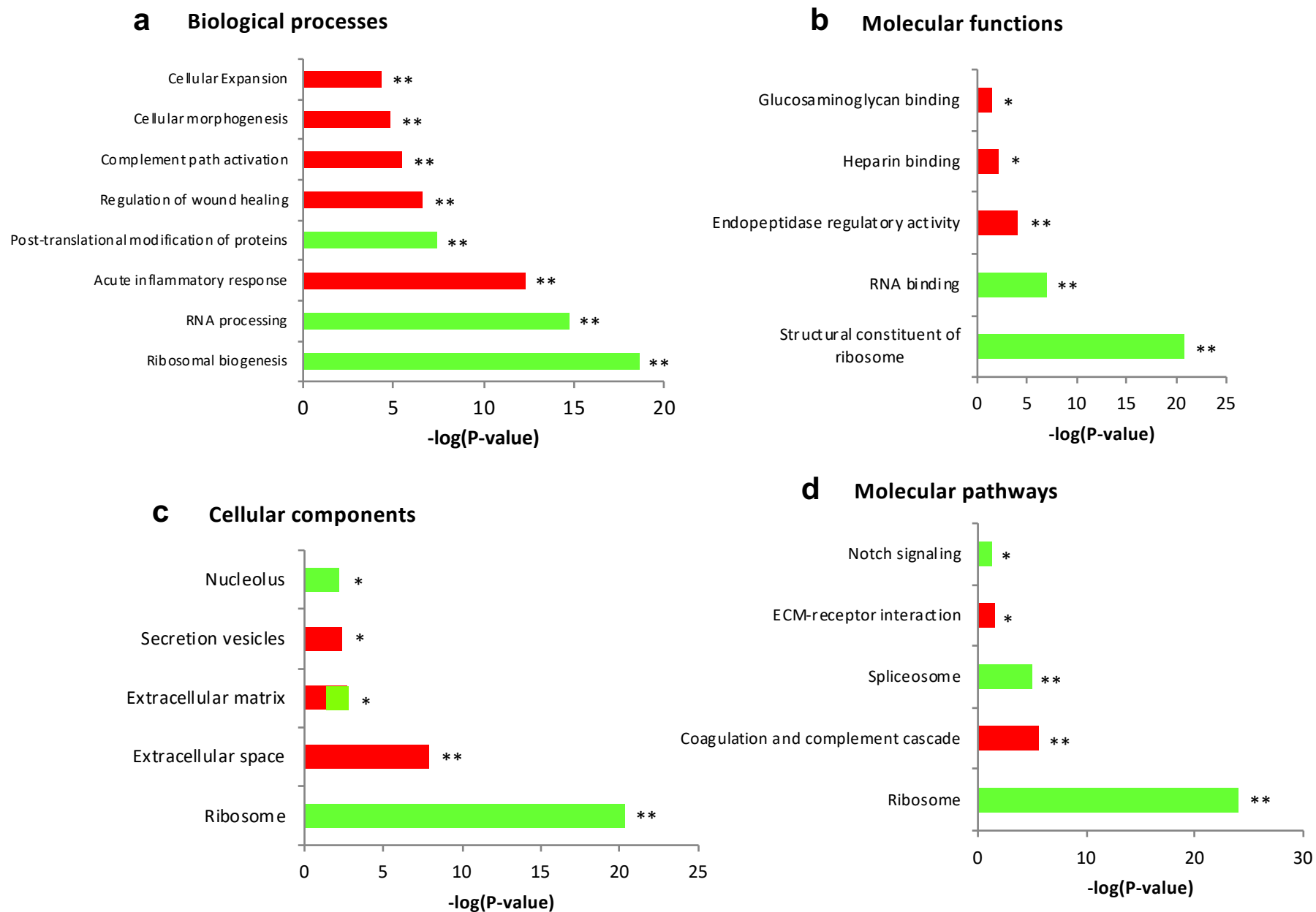

Supplement: Supplementary file 2 [file JCMM-23-8219-s002.pdf]

**a**

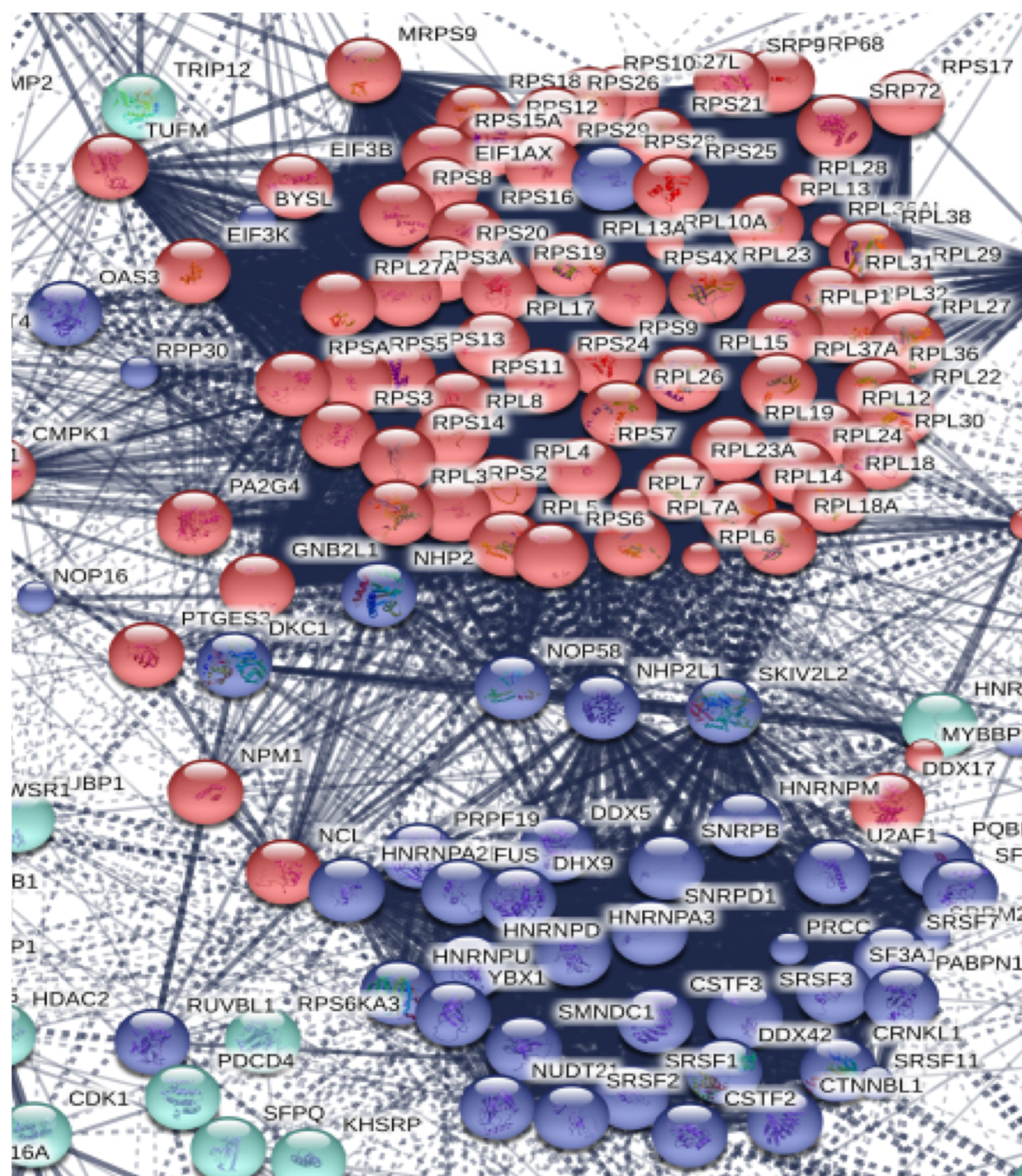

Supplement: Supplementary file 3 [file JCMM-23-8219-s003.pdf]

## Figure S3

**b**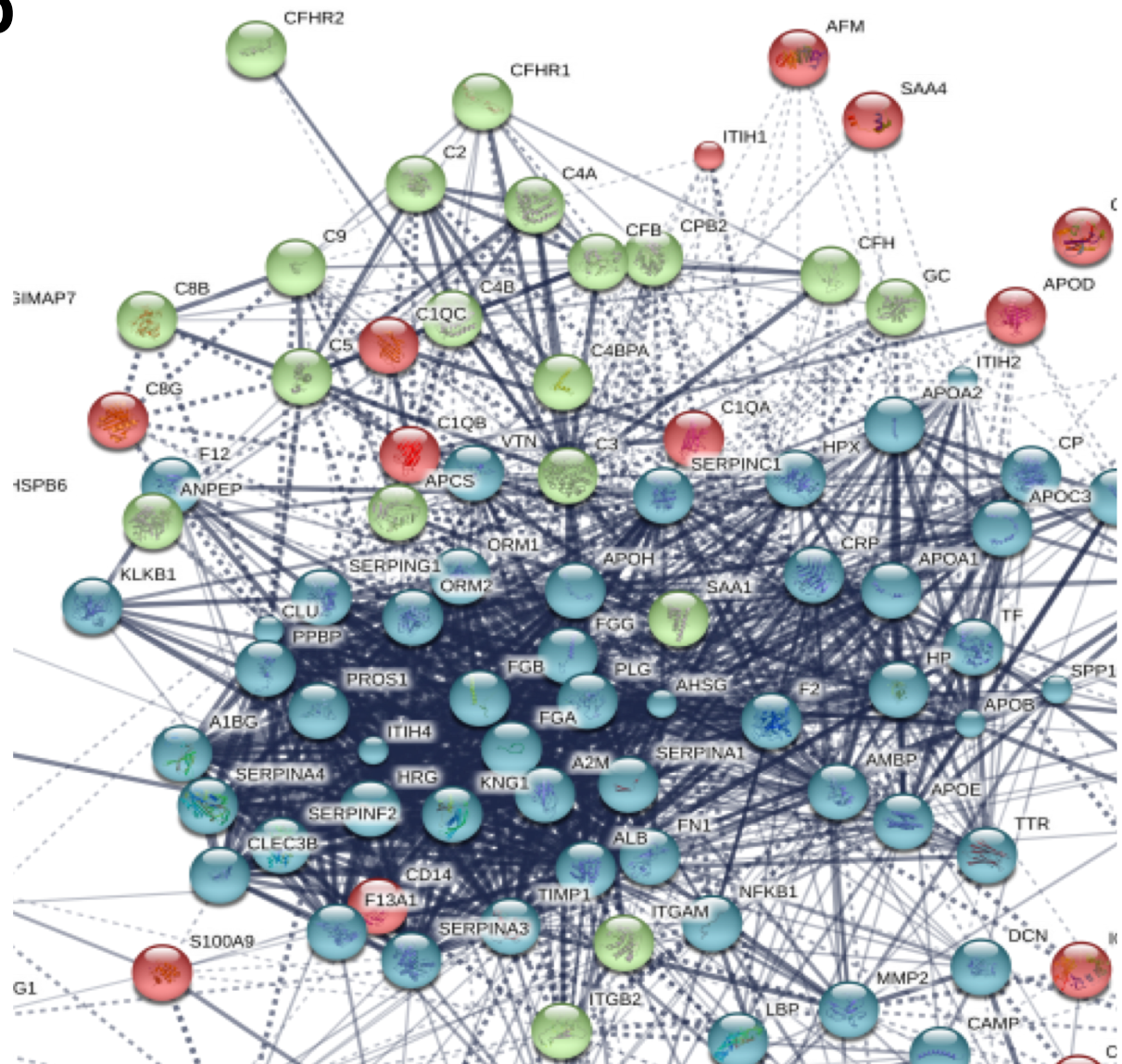

Supplement: Supplementary file 4 [file JCMM-23-8219-s004.pdf]

**Figure S4**

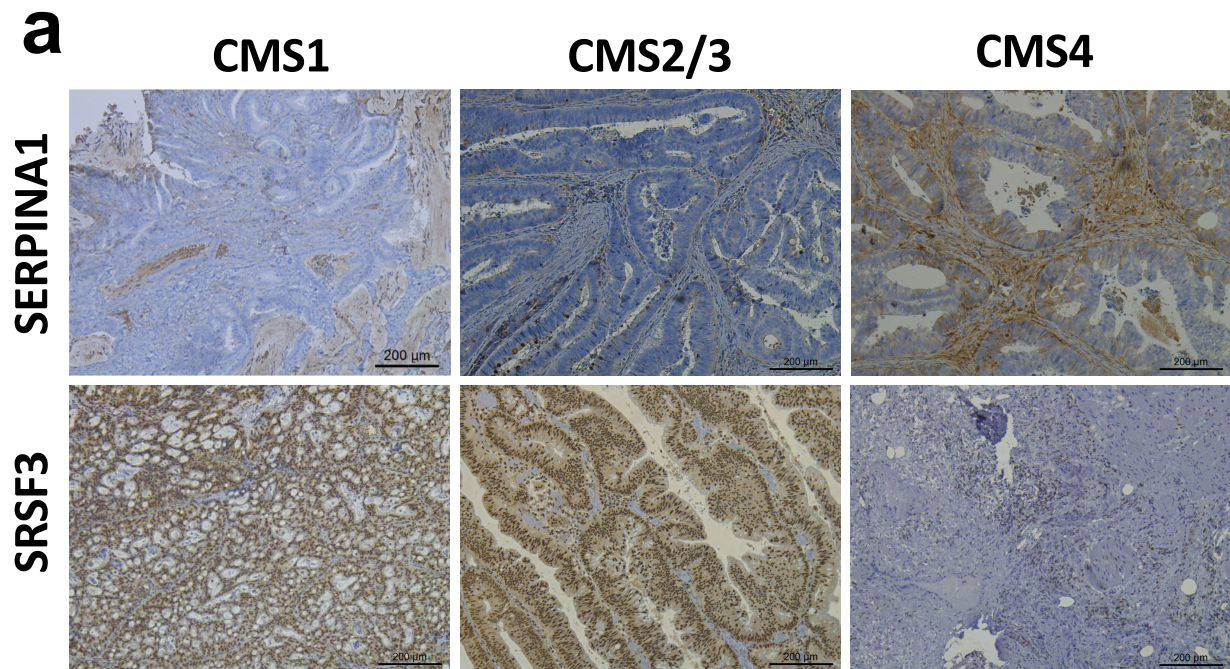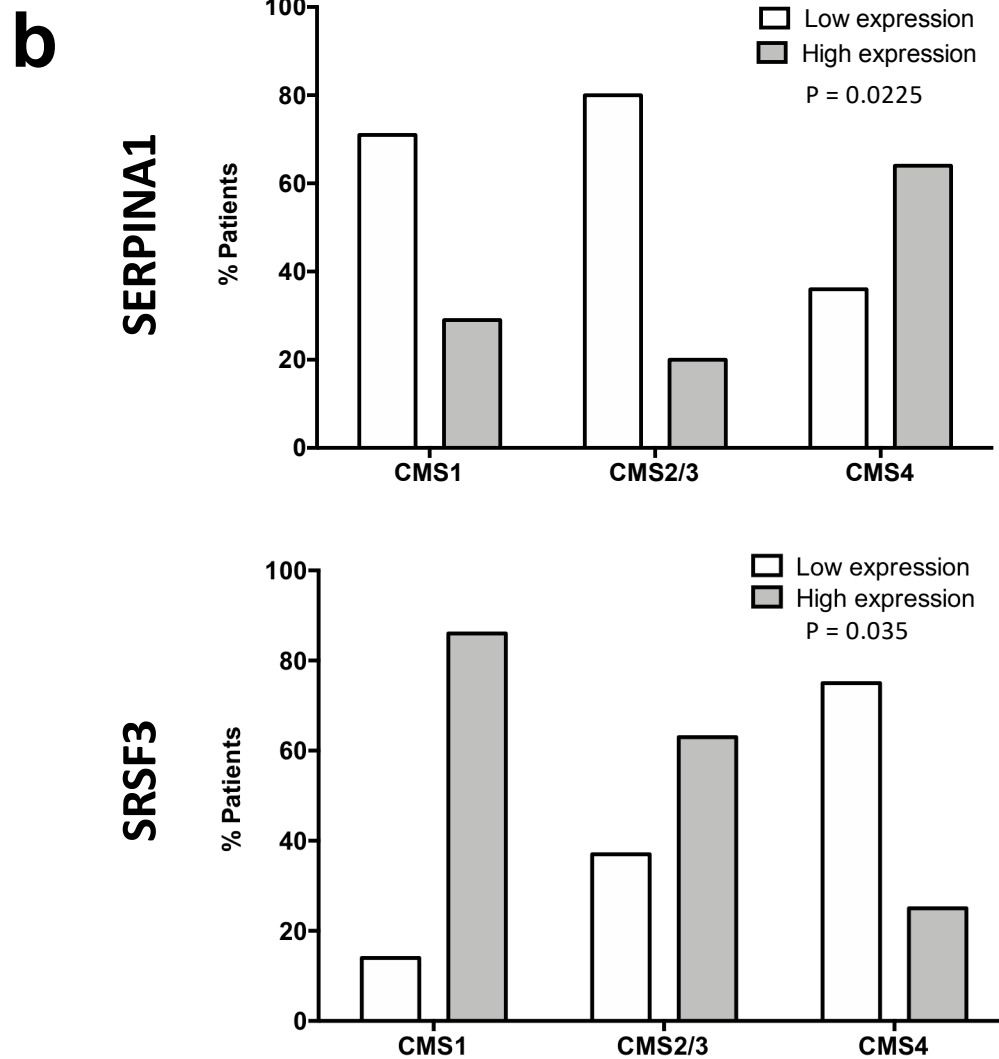

Supplement: Supplementary file 5 [file JCMM-23-8219-s005.pdf]
