# Supplementary material for: SWATH‐based proteomics reveals processes associated with immune evasion and metastasis in poor prognosis colorectal tumours
Source: J Cell Mol Med. 2019 Sep 27;23(12):8219–32. doi: 10.1111/jcmm.14693 (PMC6850959; doi:10.1111/jcmm.14693)
Supplement: Supplementary file 6 [file JCMM-23-8219-s006.docx]

**Table S1. Clinical and pathological characteristics of patients**

| **Characteristics** |  | **Patients** |
| --- | --- | --- |
| **Number** |  | 40 |
| **Age** |  | 69 ± 9 |
| **Gender** |  |  |
| Men |  | 26 (65%) |
| Women |  | 14 (35%) |
| **Anatomical location** |  |  |
| Right colon |  | 16 (40%) |
| Left colon |  | 3 (7,5%) |
| Transverse colon |  | 3 (7.5 %) |
| Sigmoid-rectum colon |  | 18 (45 %) |
| **Tumor differentiation grade** |  |  |
| Low | | 35 (87.5%) |
| High | | 4 (10%) |
| Undetermined | | 1 (2.5%) |
| **Tumor stage** |  |  |
| I |  | 8 (20%) |
| II |  | 17 (42.5%) |
| III |  | 9 (22.5%) |
| IV |  | 3 (7.5%) |
| Undetermined |  | 3 (7.5%) |
|  |  |  |
|  |  |  |
