# Supplementary material for: SWATH‐based proteomics reveals processes associated with immune evasion and metastasis in poor prognosis colorectal tumours
Source: J Cell Mol Med. 2019 Sep 27;23(12):8219–32. doi: 10.1111/jcmm.14693 (PMC6850959; doi:10.1111/jcmm.14693)
Supplement: Supplementary file 7 [file JCMM-23-8219-s007.docx]

**Table S2. Clinical and pathological characteristics of paraffin-embedded tumor tissues for CMS classification**

| **Characteristics** |  | **Patients** |
| --- | --- | --- |
| **Number** |  | 45 |
| **Age** |  | 73.8 ± 10.1 |
| **Gender** |  |  |
| Men |  | 30 (67%) |
| Women |  | 15 (33%) |
| **Anatomical location** |  |  |
| Right colon |  | 25 (55,6%) |
| Left colon |  | 15 (33,3%) |
| Transverse colon |  | 0 (0,0 %) |
| Sigmoid-rectum colon |  | 5 (11,1 %) |
| **Tumor differentiation grade** |  |  |
| Low | | 39 (87%) |
| High | | 6 (13%) |
| **Tumor stage** |  |  |
| 0  I |  | 1 (2,2%)  1 (2,2%) |
| II |  | 21 (46,7%) |
| III |  | 16 (35,6%) |
| IV |  | 5 (11,1%) |
| Undetermined |  | 1 (2,2%) |
|  |  |  |
|  |  |  |
