# Supplementary material for: SWATH‐based proteomics reveals processes associated with immune evasion and metastasis in poor prognosis colorectal tumours
Source: J Cell Mol Med. 2019 Sep 27;23(12):8219–32. doi: 10.1111/jcmm.14693 (PMC6850959; doi:10.1111/jcmm.14693)
Supplement: Supplementary file 8 [file JCMM-23-8219-s008.doc]

**Table S3. Expression values of classifier genes used for the gene expression analyzes to characterize different molecular subtypes of CRC ^(a)^**

| **Gene Name** | **CDX2** | **CFTR** | **CYP1B1** | **FLNA** | **FRMD6** | **GAS1** | **HTR2B** | **INHBA** | **MUC2** | **RARRES3** | **RGS4** | **SFRP2** | **TFF3** | **ZEB1** |
| --- | --- | --- | --- | --- | --- | --- | --- | --- | --- | --- | --- | --- | --- | --- |
| Accession # | NM_001265.2 | NM_000492.3 | NM_000104.3 | NM_001456.3 | NM_152330.3 | NM_002048.2 | NM_000867.4 | NM_002192.2 | NM_002457.2 | NM_004585.3 | NM_005613.3 | NM_003013.2 | NM_003226.3 | NM_001128128.1 |
| Class Name | Endogenous | Endogenous | Endogenous | Endogenous | Endogenous | Endogenous | Endogenous | Endogenous | Endogenous | Endogenous | Endogenous | Endogenous | Endogenous | Endogenous |
| **Sample10** | 599.13 | 1805.19 | 9.11 | 388.66 | 89.51 | 19.75 | 1.18 | 115.52 | 3940.64 | 97.79 | 26.84 | 1.18 | 8606.45 | 71.77 |
| **Sample12** | 1160.92 | 6506.78 | 42.87 | 1087.60 | 85.64 | 67.31 | 6.11 | 91.75 | 132956.28 | 427.77 | 30.65 | 24.54 | 11315.00 | 165.06 |
| **Sample13** | 14.66 | 130.24 | 758.39 | 46259.92 | 513.83 | 964.42 | 44.81 | 138.61 | 100.09 | 1232.43 | 178.81 | 7356.48 | 306.12 | 1592.57 |
| **Sample18** | 73.46 | 321.00 | 14.15 | 779.10 | 62.58 | 108.28 | 1.00 | 384.12 | 1730.12 | 190.97 | 26.67 | 103.92 | 649.62 | 50.06 |
| **Sample19** | 599.89 | 580.44 | 6.16 | 287.67 | 151.52 | 17.42 | 1.02 | 204.75 | 283.58 | 71.67 | 43.01 | 8.21 | 3818.35 | 74.75 |
| **Sample20.1** | 1362.55 | 1896.02 | 13.02 | 426.98 | 30.43 | 12.63 | 1.00 | 320.92 | 569.45 | 30.04 | 38.35 | 213.27 | 7158.78 | 42.31 |
| **Sample20** | 1185.26 | 1295.55 | 28.23 | 554.41 | 13.27 | 17.94 | 1.00 | 208.60 | 3713.36 | 78.69 | 9.53 | 102.06 | 5687.24 | 46.92 |
| **Sample4** | 1744.58 | 3829.08 | 59.99 | 2414.42 | 74.98 | 44.99 | 5.00 | 269.93 | 4693.87 | 479.88 | 49.99 | 279.93 | 2964.28 | 89.98 |
| **Sample5** | 409.21 | 207.17 | 3232.69 | 3894.36 | 570.84 | 2323.52 | 85.95 | 2788.21 | 15.24 | 707.22 | 121.31 | 11834.44 | 858.75 | 570.84 |
| **Sample7** | 940.97 | 1272.61 | 16.38 | 240.71 | 15.17 | 15.17 | 1.00 | 75.19 | 4619.33 | 317.10 | 10.32 | 6.07 | 2635.55 | 18.81 |
| **Sample8** | 1111.08 | 4616.15 | 34.36 | 744.54 | 45.82 | 45.82 | 11.45 | 34.36 | 10045.56 | 492.54 | 103.09 | 68.73 | 801.81 | 137.45 |
| **Sample9** | 1090.06 | 4812.70 | 30.77 | 136.70 | 15.64 | 15.64 | 15.13 | 15.64 | 89888.53 | 484.75 | 30.77 | 121.57 | 7324.72 | 15.64 |
| **Sample22** | 2129.86 | 2178.33 | 171.07 | 1987.30 | 128.30 | 333.59 | 2.85 | 85.54 | 12964.46 | 533.18 | 34.21 | 886.73 | 3022.29 | 191.03 |
| **Sample23** | 924.60 | 3369.42 | 10.53 | 631.35 | 24.56 | 4.29 | 1.00 | 156.63 | 430.65 | 187.31 | 8.45 | 1.17 | 898.60 | 28.20 |
| **Sample24** | 1012.83 | 459.90 | 87.18 | 931.56 | 58.09 | 18.51 | 9.82 | 83.88 | 11.61 | 235.01 | 6.82 | 100.97 | 4650.95 | 41.30 |
| **Sample25** | 1089.08 | 2448.94 | 8.56 | 314.85 | 29.83 | 1.42 | 1.42 | 80.87 | 9334.78 | 59.60 | 21.32 | 1.42 | 5138.90 | 59.60 |
| **Sample26** | 730.65 | 1756.75 | 2.19 | 755.04 | 7.20 | 6.57 | 1.00 | 164.14 | 619.35 | 39.71 | 2.82 | 4.07 | 2637.15 | 12.20 |
| **Sample27** | 1298.05 | 1738.93 | 3.47 | 263.53 | 10.11 | 3.47 | 3.47 | 121.20 | 1061.98 | 51.76 | 23.99 | 3.47 | 9112.48 | 3.47 |
| **Sample28** | 1509.62 | 1212.61 | 78.59 | 616.15 | 36.86 | 27.04 | 22.13 | 481.14 | 903.33 | 189.05 | 78.59 | 51.59 | 1305.89 | 157.14 |
| **Sample29** | 616.02 | 1245.11 | 17.99 | 518.94 | 17.99 | 52.94 | 6.34 | 282.06 | 1839.25 | 666.50 | 10.23 | 17.99 | 546.12 | 91.78 |
| **Sample30** | 1956.82 | 3270.15 | 11.54 | 399.89 | 15.07 | 8.01 | 3.53 | 11.54 | 34126.36 | 1000.07 | 4.48 | 3.53 | 3040.67 | 43.31 |
| **Sample32** | 445.44 | 524.70 | 7.16 | 510.20 | 34.10 | 26.85 | 1.00 | 185.89 | 23158.09 | 223.19 | 11.83 | 33.58 | 8683.06 | 55.34 |
| **Sample33** | 539.60 | 32.24 | 449.53 | 3434.59 | 267.97 | 1298.88 | 41.38 | 1690.14 | 11784.61 | 203.23 | 66.01 | 5110.09 | 5809.56 | 234.20 |
| **Sample34** | 2102.32 | 4515.59 | 35.36 | 1001.40 | 24.43 | 82.75 | 3.65 | 203.05 | 8357.87 | 173.89 | 3.65 | 254.09 | 994.11 | 42.65 |
| **Sample35** | 1831.68 | 2399.24 | 1.00 | 588.76 | 24.89 | 4.62 | 1.00 | 232.20 | 1134.21 | 145.59 | 47.00 | 1.00 | 1053.13 | 75.57 |
| **Sample36** | 1246.00 | 4073.73 | 8.11 | 303.43 | 39.13 | 5.42 | 1.35 | 56.66 | 631.10 | 58.01 | 9.46 | 1.37 | 6225.87 | 95.76 |
| **Sample37** | 1348.18 | 3308.42 | 223.32 | 3575.85 | 173.69 | 88.22 | 2.76 | 1080.75 | 9227.72 | 507.29 | 33.08 | 1414.35 | 1932.67 | 482.48 |
| **Sample38.1** | 482.90 | 2623.54 | 151.26 | 211.56 | 30.66 | 30.66 | 30.66 | 392.46 | 39557.07 | 151.26 | 151.26 | 241.71 | 3467.74 | 211.56 |
| **Sample38.2** | 51.52 | 21.47 | 210.37 | 62090.39 | 442.21 | 1206.43 | 188.91 | 553.84 | 25.76 | 1038.99 | 42.93 | 1223.60 | 34.35 | 2017.87 |
| **Sample39** | 3.15 | 3.15 | 506.65 | 59353.62 | 610.50 | 239.16 | 163.64 | 714.35 | 12.59 | 793.02 | 103.85 | 4015.44 | 18.88 | 1866.11 |
| **Sample40** | 1931.92 | 2114.34 | 2.70 | 275.90 | 22.88 | 1.00 | 1.00 | 158.22 | 1333.40 | 199.41 | 1.02 | 1.02 | 2996.99 | 45.57 |
| **Sample41** | 1093.09 | 1951.83 | 10.49 | 212.09 | 10.49 | 9.19 | 1.31 | 130.93 | 19607.12 | 163.65 | 2.64 | 9.19 | 6918.40 | 56.31 |
| **Sample42** | 2022.72 | 4183.48 | 69.02 | 663.62 | 26.54 | 15.93 | 5.31 | 621.15 | 10.62 | 47.78 | 37.16 | 21.24 | 1948.40 | 153.96 |
| **Sample43** | 185.79 | 428.26 | 20.65 | 371.94 | 27.33 | 31.15 | 1.00 | 86.52 | 3104.90 | 1384.75 | 11.11 | 8.24 | 5319.53 | 61.70 |
| **Sample44** | 2190.35 | 4971.86 | 104.86 | 854.81 | 58.63 | 35.51 | 2.57 | 171.63 | 4563.49 | 636.50 | 12.40 | 40.65 | 1779.41 | 163.93 |
| **Sample45** | 720.88 | 1032.84 | 8.45 | 294.26 | 37.11 | 12.66 | 1.00 | 161.05 | 8105.76 | 75.05 | 4.23 | 28.68 | 4922.14 | 39.64 |
| **Sample46** | 503.24 | 1448.58 | 2.16 | 176.17 | 13.79 | 2.99 | 1.00 | 44.87 | 235.00 | 223.20 | 73.95 | 2.66 | 5446.13 | 8.80 |
| **Sample47** | 1317.85 | 3351.52 | 36.71 | 414.81 | 40.38 | 150.51 | 3.67 | 444.18 | 7194.95 | 407.47 | 18.35 | 33.04 | 2268.61 | 40.38 |
| **Sample48** | 2611.70 | 4031.43 | 7.91 | 362.60 | 23.26 | 8.39 | 1.00 | 67.90 | 16.54 | 225.33 | 2.63 | 8.86 | 4779.21 | 26.62 |
| **Sample50** | 755.09 | 745.68 | 10.73 | 564.30 | 19.24 | 12.52 | 1.00 | 123.15 | 13.42 | 82.84 | 12.08 | 21.03 | 1420.17 | 30.89 |

^(a)^ nCounter Element system by NanoString was used to analyse the RNA expression of a set of genes and classify CRC. For these classifications the expression of those genes included in the qPCR mini-classifiers described by Sadanandam et al. (7 genes), and by De Sousa E Melo et al (8 genes), was used. ZEB1 gene is included in both mini-classifiers
