# Supplementary material for: SWATH‐based proteomics reveals processes associated with immune evasion and metastasis in poor prognosis colorectal tumours
Source: J Cell Mol Med. 2019 Sep 27;23(12):8219–32. doi: 10.1111/jcmm.14693 (PMC6850959; doi:10.1111/jcmm.14693)
Supplement: Supplementary file 9 [file JCMM-23-8219-s009.doc]

**Table S4. List of identified proteins in the SWATH-based proteomic analysis. (See explanatory notes at the end of the Table)**

| **Protein** | **Representative Accession** | **Species** | **Name** | **Unused ProtScore** | **Total ProtScore** | **Confident Peptides** | **%Seq Cov - peptides >95% conf** |
| --- | --- | --- | --- | --- | --- | --- | --- |
| 1 | sp|Q09666|AHNK_HUMAN | HUMAN | Neuroblast differentiation-associated protein AHNAK OS=Homo sapiens GN=AHNAK PE=1 SV=2 | 413,59 | 413,59 | 247 | 65,98 |
| 2 | sp|P21333|FLNA_HUMAN | HUMAN | Filamin-A OS=Homo sapiens GN=FLNA PE=1 SV=4 | 322,29 | 322,28 | 357 | 77,52 |
| 3 | sp|P35749|MYH11_HUMAN | HUMAN | Myosin-11 OS=Homo sapiens GN=MYH11 PE=1 SV=3 | 258,92 | 260,12 | 218 | 60,45 |
| 4 | sp|Q13813|SPTN1_HUMAN | HUMAN | Spectrin alpha chain, non-erythrocytic 1 OS=Homo sapiens GN=SPTAN1 PE=1 SV=3 | 253,54 | 253,51 | 147 | 63,83 |
| 5 | sp|Q14204|DYHC1_HUMAN | HUMAN | Cytoplasmic dynein 1 heavy chain 1 OS=Homo sapiens GN=DYNC1H1 PE=1 SV=5 | 250,54 | 250,51 | 130 | 33,79 |
| 6 | sp|Q9Y6R7|FCGBP_HUMAN | HUMAN | IgGFc-binding protein OS=Homo sapiens GN=FCGBP PE=1 SV=3 | 240,52 | 240,51 | 223 | 54,47 |
| 7 | sp|Q9Y490|TLN1_HUMAN | HUMAN | Talin-1 OS=Homo sapiens GN=TLN1 PE=1 SV=3 | 232,97 | 232,96 | 158 | 64,31 |
| 8 | sp|P02768|ALBU_HUMAN | HUMAN | Serum albumin OS=Homo sapiens GN=ALB PE=1 SV=2 | 227,11 | 227,1 | 865 | 89,82 |
| 9 | sp|O75369|FLNB_HUMAN | HUMAN | Filamin-B OS=Homo sapiens GN=FLNB PE=1 SV=2 | 222,92 | 246,35 | 143 | 63,68 |
| 10 | sp|P78527|PRKDC_HUMAN | HUMAN | DNA-dependent protein kinase catalytic subunit OS=Homo sapiens GN=PRKDC PE=1 SV=3 | 213,09 | 213,07 | 110 | 30,35 |
| 11 | sp|Q15149|PLEC_HUMAN | HUMAN | Plectin OS=Homo sapiens GN=PLEC PE=1 SV=3 | 206,85 | 206,97 | 104 | 26,39 |
| 12 | sp|P35579|MYH9_HUMAN | HUMAN | Myosin-9 OS=Homo sapiens GN=MYH9 PE=1 SV=4 | 191,76 | 229,03 | 164 | 58,16 |
| 13 | sp|P01024|CO3_HUMAN | HUMAN | Complement C3 OS=Homo sapiens GN=C3 PE=1 SV=2 | 188,06 | 188,06 | 150 | 66,99 |
| 14 | sp|Q01082|SPTB2_HUMAN | HUMAN | Spectrin beta chain, non-erythrocytic 1 OS=Homo sapiens GN=SPTBN1 PE=1 SV=2 | 175,04 | 175,03 | 92 | 44,71 |
| 15 | sp|P49327|FAS_HUMAN | HUMAN | Fatty acid synthase OS=Homo sapiens GN=FASN PE=1 SV=3 | 173,55 | 173,54 | 106 | 48,71 |
| 16 | sp|P46940|IQGA1_HUMAN | HUMAN | Ras GTPase-activating-like protein IQGAP1 OS=Homo sapiens GN=IQGAP1 PE=1 SV=1 | 164,3 | 164,28 | 106 | 60,05 |
| 17 | sp|Q00610|CLH1_HUMAN | HUMAN | Clathrin heavy chain 1 OS=Homo sapiens GN=CLTC PE=1 SV=5 | 159,76 | 159,68 | 120 | 57,73 |
| 18 | sp|P18206|VINC_HUMAN | HUMAN | Vinculin OS=Homo sapiens GN=VCL PE=1 SV=4 | 140,73 | 140,73 | 114 | 63,84 |
| 19 | sp|P04114|APOB_HUMAN | HUMAN | Apolipoprotein B-100 OS=Homo sapiens GN=APOB PE=1 SV=2 | 136,12 | 136,95 | 65 | 16,63 |
| 20 | sp|O43707|ACTN4_HUMAN | HUMAN | Alpha-actinin-4 OS=Homo sapiens GN=ACTN4 PE=1 SV=2 | 132,39 | 132,39 | 121 | 76,73 |
| 21 | sp|Q9P2E9|RRBP1_HUMAN | HUMAN | Ribosome-binding protein 1 OS=Homo sapiens GN=RRBP1 PE=1 SV=4 | 130,33 | 130,32 | 85 | 48,87 |
| 22 | sp|P01023|A2MG_HUMAN | HUMAN | Alpha-2-macroglobulin OS=Homo sapiens GN=A2M PE=1 SV=3 | 130,13 | 130,33 | 98 | 60,38 |
| 23 | sp|P0C0L4|CO4A_HUMAN | HUMAN | Complement C4-A OS=Homo sapiens GN=C4A PE=1 SV=2 | 120,27 | 120,27 | 68 | 43,18 |
| 24 | sp|Q05707|COEA1_HUMAN | HUMAN | Collagen alpha-1(XIV) chain OS=Homo sapiens GN=COL14A1 PE=1 SV=3 | 113,31 | 113,31 | 68 | 46,38 |
| 25 | sp|P08238|HS90B_HUMAN | HUMAN | Heat shock protein HSP 90-beta OS=Homo sapiens GN=HSP90AB1 PE=1 SV=4 | 112,24 | 112,27 | 132 | 70,03 |
| 26 | sp|P13639|EF2_HUMAN | HUMAN | Elongation factor 2 OS=Homo sapiens GN=EEF2 PE=1 SV=4 | 110,93 | 110,93 | 100 | 72,61 |
| 27 | sp|P02787|TRFE_HUMAN | HUMAN | Serotransferrin OS=Homo sapiens GN=TF PE=1 SV=3 | 107,59 | 107,72 | 158 | 82,38 |
| 28 | sp|Q05682|CALD1_HUMAN | HUMAN | Caldesmon OS=Homo sapiens GN=CALD1 PE=1 SV=3 | 103,48 | 103,62 | 92 | 50,69 |
| 29 | sp|P05787|K2C8_HUMAN | HUMAN | Keratin, type II cytoskeletal 8 OS=Homo sapiens GN=KRT8 PE=1 SV=7 | 101,95 | 101,95 | 119 | 81,57 |
| 30 | sp|P53621|COPA_HUMAN | HUMAN | Coatomer subunit alpha OS=Homo sapiens GN=COPA PE=1 SV=2 | 99,22 | 99,24 | 60 | 51,88 |
| 31 | sp|P02751|FINC_HUMAN | HUMAN | Fibronectin OS=Homo sapiens GN=FN1 PE=1 SV=4 | 99,1 | 99,1 | 59 | 33,49 |
| 32 | sp|P07814|SYEP_HUMAN | HUMAN | Bifunctional glutamate/proline--tRNA ligase OS=Homo sapiens GN=EPRS PE=1 SV=5 | 97,6 | 97,6 | 54 | 42,2 |
| 33 | sp|P14625|ENPL_HUMAN | HUMAN | Endoplasmin OS=Homo sapiens GN=HSP90B1 PE=1 SV=1 | 97,55 | 101,67 | 107 | 67,62 |
| 34 | sp|P55072|TERA_HUMAN | HUMAN | Transitional endoplasmic reticulum ATPase OS=Homo sapiens GN=VCP PE=1 SV=4 | 94,82 | 94,82 | 68 | 71,34 |
| 35 | sp|P11216|PYGB_HUMAN | HUMAN | Glycogen phosphorylase, brain form OS=Homo sapiens GN=PYGB PE=1 SV=5 | 94,79 | 94,8 | 74 | 63,46 |
| 36 | sp|P22314|UBA1_HUMAN | HUMAN | Ubiquitin-like modifier-activating enzyme 1 OS=Homo sapiens GN=UBA1 PE=1 SV=3 | 91,3 | 91,28 | 105 | 54,73 |
| 37 | sp|Q15746|MYLK_HUMAN | HUMAN | Myosin light chain kinase, smooth muscle OS=Homo sapiens GN=MYLK PE=1 SV=4 | 89,78 | 89,78 | 70 | 32,86 |
| 38 | sp|P14618|KPYM_HUMAN | HUMAN | Pyruvate kinase PKM OS=Homo sapiens GN=PKM PE=1 SV=4 | 88,07 | 88,47 | 114 | 81,92 |
| 39 | sp|P42704|LPPRC_HUMAN | HUMAN | Leucine-rich PPR motif-containing protein, mitochondrial OS=Homo sapiens GN=LRPPRC PE=1 SV=3 | 87,57 | 87,6 | 46 | 37,88 |
| 40 | sp|Q14764|MVP_HUMAN | HUMAN | Major vault protein OS=Homo sapiens GN=MVP PE=1 SV=4 | 87,05 | 87,07 | 61 | 59,46 |
| 41 | sp|P27816|MAP4_HUMAN | HUMAN | Microtubule-associated protein 4 OS=Homo sapiens GN=MAP4 PE=1 SV=3 | 86,77 | 86,93 | 59 | 62,5 |
| 42 | sp|P08603|CFAH_HUMAN | HUMAN | Complement factor H OS=Homo sapiens GN=CFH PE=1 SV=4 | 86,62 | 86,66 | 53 | 52,32 |
| 43 | sp|Q14315|FLNC_HUMAN | HUMAN | Filamin-C OS=Homo sapiens GN=FLNC PE=1 SV=3 | 86,48 | 117,13 | 73 | 31,27 |
| 44 | sp|P08670|VIME_HUMAN | HUMAN | Vimentin OS=Homo sapiens GN=VIM PE=1 SV=4 | 86,4 | 91,17 | 118 | 81,76 |
| 45 | sp|P08727|K1C19_HUMAN | HUMAN | Keratin, type I cytoskeletal 19 OS=Homo sapiens GN=KRT19 PE=1 SV=4 | 85,98 | 86,01 | 79 | 91 |
| 46 | sp|P11021|GRP78_HUMAN | HUMAN | 78 kDa glucose-regulated protein OS=Homo sapiens GN=HSPA5 PE=1 SV=2 | 85,24 | 85,29 | 68 | 67,74 |
| 47 | sp|Q86VP6|CAND1_HUMAN | HUMAN | Cullin-associated NEDD8-dissociated protein 1 OS=Homo sapiens GN=CAND1 PE=1 SV=2 | 84,67 | 84,71 | 49 | 43,98 |
| 48 | sp|P02788|TRFL_HUMAN | HUMAN | Lactotransferrin OS=Homo sapiens GN=LTF PE=1 SV=6 | 84,27 | 88,21 | 56 | 76,48 |
| 49 | sp|P13796|PLSL_HUMAN | HUMAN | Plastin-2 OS=Homo sapiens GN=LCP1 PE=1 SV=6 | 84,2 | 84,23 | 76 | 78,47 |
| 50 | sp|Q14697|GANAB_HUMAN | HUMAN | Neutral alpha-glucosidase AB OS=Homo sapiens GN=GANAB PE=1 SV=3 | 83,29 | 83,9 | 49 | 55,4 |
| 51 | sp|P31948|STIP1_HUMAN | HUMAN | Stress-induced-phosphoprotein 1 OS=Homo sapiens GN=STIP1 PE=1 SV=1 | 79,74 | 79,74 | 50 | 60,41 |
| 52 | sp|Q92616|GCN1_HUMAN | HUMAN | eIF-2-alpha kinase activator GCN1 OS=Homo sapiens GN=GCN1 PE=1 SV=6 | 78,81 | 78,82 | 39 | 19,47 |
| 53 | sp|P63261|ACTG_HUMAN | HUMAN | Actin, cytoplasmic 2 OS=Homo sapiens GN=ACTG1 PE=1 SV=1 | 78,61 | 78,62 | 346 | 80,27 |
| 54 | sp|P12814|ACTN1_HUMAN | HUMAN | Alpha-actinin-1 OS=Homo sapiens GN=ACTN1 PE=1 SV=2 | 77,97 | 123,57 | 105 | 76,57 |
| 55 | sp|P07237|PDIA1_HUMAN | HUMAN | Protein disulfide-isomerase OS=Homo sapiens GN=P4HB PE=1 SV=3 | 77,52 | 77,61 | 81 | 80,51 |
| 56 | sp|Q13228|SBP1_HUMAN | HUMAN | Selenium-binding protein 1 OS=Homo sapiens GN=SELENBP1 PE=1 SV=2 | 77,28 | 77,34 | 63 | 82,2 |
| 57 | sp|A8K7I4|CLCA1_HUMAN | HUMAN | Calcium-activated chloride channel regulator 1 OS=Homo sapiens GN=CLCA1 PE=1 SV=3 | 77,19 | 77,36 | 64 | 53,17 |
| 58 | sp|P02671|FIBA_HUMAN | HUMAN | Fibrinogen alpha chain OS=Homo sapiens GN=FGA PE=1 SV=2 | 76,76 | 76,86 | 100 | 55,66 |
| 59 | sp|P11142|HSP7C_HUMAN | HUMAN | Heat shock cognate 71 kDa protein OS=Homo sapiens GN=HSPA8 PE=1 SV=1 | 76,33 | 80,54 | 99 | 71,21 |
| 60 | sp|P26038|MOES_HUMAN | HUMAN | Moesin OS=Homo sapiens GN=MSN PE=1 SV=3 | 76,19 | 76,53 | 59 | 64,12 |
| 61 | sp|P04075|ALDOA_HUMAN | HUMAN | Fructose-bisphosphate aldolase A OS=Homo sapiens GN=ALDOA PE=1 SV=2 | 75,87 | 75,92 | 118 | 90,66 |
| 62 | sp|P12111|CO6A3_HUMAN | HUMAN | Collagen alpha-3(VI) chain OS=Homo sapiens GN=COL6A3 PE=1 SV=5 | 75,67 | 76,01 | 40 | 16,53 |
| 63 | sp|P68871|HBB_HUMAN | HUMAN | Hemoglobin subunit beta OS=Homo sapiens GN=HBB PE=1 SV=2 | 75,06 | 75,16 | 357 | 99,32 |
| 64 | sp|P33176|KINH_HUMAN | HUMAN | Kinesin-1 heavy chain OS=Homo sapiens GN=KIF5B PE=1 SV=1 | 74,85 | 74,98 | 42 | 49,33 |
| 65 | sp|P10809|CH60_HUMAN | HUMAN | 60 kDa heat shock protein, mitochondrial OS=Homo sapiens GN=HSPD1 PE=1 SV=2 | 74,16 | 74,21 | 81 | 74,17 |
| 66 | sp|P29401|TKT_HUMAN | HUMAN | Transketolase OS=Homo sapiens GN=TKT PE=1 SV=3 | 74,02 | 74,19 | 59 | 75,92 |
| 67 | sp|P30101|PDIA3_HUMAN | HUMAN | Protein disulfide-isomerase A3 OS=Homo sapiens GN=PDIA3 PE=1 SV=4 | 73,64 | 75,16 | 77 | 76,04 |
| 68 | sp|P55786|PSA_HUMAN | HUMAN | Puromycin-sensitive aminopeptidase OS=Homo sapiens GN=NPEPPS PE=1 SV=2 | 73,52 | 73,64 | 41 | 52,88 |
| 69 | sp|P26640|SYVC_HUMAN | HUMAN | Valine--tRNA ligase OS=Homo sapiens GN=VARS PE=1 SV=4 | 72,88 | 72,92 | 39 | 36,23 |
| 70 | sp|P06396|GELS_HUMAN | HUMAN | Gelsolin OS=Homo sapiens GN=GSN PE=1 SV=1 | 71,93 | 71,93 | 76 | 59,21 |
| 71 | sp|Q8WUM4|PDC6I_HUMAN | HUMAN | Programmed cell death 6-interacting protein OS=Homo sapiens GN=PDCD6IP PE=1 SV=1 | 71,61 | 71,74 | 43 | 53,46 |
| 72 | sp|P05783|K1C18_HUMAN | HUMAN | Keratin, type I cytoskeletal 18 OS=Homo sapiens GN=KRT18 PE=1 SV=2 | 70,94 | 75,31 | 74 | 82,56 |
| 73 | sp|P07900|HS90A_HUMAN | HUMAN | Heat shock protein HSP 90-alpha OS=Homo sapiens GN=HSP90AA1 PE=1 SV=5 | 70,89 | 111,7 | 122 | 69,67 |
| 74 | sp|P08133|ANXA6_HUMAN | HUMAN | Annexin A6 OS=Homo sapiens GN=ANXA6 PE=1 SV=3 | 70,8 | 70,88 | 38 | 55,72 |
| 75 | sp|Q7KZF4|SND1_HUMAN | HUMAN | Staphylococcal nuclease domain-containing protein 1 OS=Homo sapiens GN=SND1 PE=1 SV=1 | 70,67 | 70,74 | 45 | 53,63 |
| 76 | sp|P31939|PUR9_HUMAN | HUMAN | Bifunctional purine biosynthesis protein PURH OS=Homo sapiens GN=ATIC PE=1 SV=3 | 69,94 | 70,19 | 43 | 77,2 |
| 77 | sp|Q00839|HNRPU_HUMAN | HUMAN | Heterogeneous nuclear ribonucleoprotein U OS=Homo sapiens GN=HNRNPU PE=1 SV=6 | 69,8 | 69,89 | 66 | 43,76 |
| 78 | sp|Q99715|COCA1_HUMAN | HUMAN | Collagen alpha-1(XII) chain OS=Homo sapiens GN=COL12A1 PE=1 SV=2 | 69,69 | 70,28 | 37 | 16,85 |
| 79 | sp|P68104|EF1A1_HUMAN | HUMAN | Elongation factor 1-alpha 1 OS=Homo sapiens GN=EEF1A1 PE=1 SV=1 | 69,22 | 69,27 | 154 | 73,38 |
| 80 | sp|Q92598|HS105_HUMAN | HUMAN | Heat shock protein 105 kDa OS=Homo sapiens GN=HSPH1 PE=1 SV=1 | 69,14 | 69,17 | 38 | 49,42 |
| 81 | sp|P13667|PDIA4_HUMAN | HUMAN | Protein disulfide-isomerase A4 OS=Homo sapiens GN=PDIA4 PE=1 SV=2 | 69,09 | 72,54 | 50 | 62,17 |
| 82 | sp|P69905|HBA_HUMAN | HUMAN | Hemoglobin subunit alpha OS=Homo sapiens GN=HBA1 PE=1 SV=2 | 68,96 | 69,08 | 324 | 96,48 |
| 83 | sp|P53396|ACLY_HUMAN | HUMAN | ATP-citrate synthase OS=Homo sapiens GN=ACLY PE=1 SV=3 | 68,72 | 68,92 | 35 | 35,06 |
| 84 | sp|Q08211|DHX9_HUMAN | HUMAN | ATP-dependent RNA helicase A OS=Homo sapiens GN=DHX9 PE=1 SV=4 | 68,64 | 68,86 | 38 | 33,39 |
| 85 | sp|P00738|HPT_HUMAN | HUMAN | Haptoglobin OS=Homo sapiens GN=HP PE=1 SV=1 | 68,57 | 68,67 | 96 | 82,76 |
| 86 | sp|P02545|LMNA_HUMAN | HUMAN | Prelamin-A/C OS=Homo sapiens GN=LMNA PE=1 SV=1 | 68,53 | 68,72 | 35 | 56,93 |
| 87 | sp|Q14152|EIF3A_HUMAN | HUMAN | Eukaryotic translation initiation factor 3 subunit A OS=Homo sapiens GN=EIF3A PE=1 SV=1 | 68,38 | 68,94 | 44 | 29,88 |
| 88 | sp|P53618|COPB_HUMAN | HUMAN | Coatomer subunit beta OS=Homo sapiens GN=COPB1 PE=1 SV=3 | 68,31 | 68,42 | 39 | 47,43 |
| 89 | sp|Q99798|ACON_HUMAN | HUMAN | Aconitate hydratase, mitochondrial OS=Homo sapiens GN=ACO2 PE=1 SV=2 | 67,3 | 67,32 | 37 | 53,72 |
| 90 | sp|P01009|A1AT_HUMAN | HUMAN | Alpha-1-antitrypsin OS=Homo sapiens GN=SERPINA1 PE=1 SV=3 | 66,81 | 66,81 | 109 | 76,08 |
| 91 | sp|P50990|TCPQ_HUMAN | HUMAN | T-complex protein 1 subunit theta OS=Homo sapiens GN=CCT8 PE=1 SV=4 | 66,67 | 66,68 | 40 | 69,34 |
| 92 | sp|P09327|VILI_HUMAN | HUMAN | Villin-1 OS=Homo sapiens GN=VIL1 PE=1 SV=4 | 65,87 | 65,91 | 43 | 50,54 |
| 93 | sp|Q71U36|TBA1A_HUMAN | HUMAN | Tubulin alpha-1A chain OS=Homo sapiens GN=TUBA1A PE=1 SV=1 | 65,35 | 65,37 | 98 | 90,91 |
| 94 | sp|P50395|GDIB_HUMAN | HUMAN | Rab GDP dissociation inhibitor beta OS=Homo sapiens GN=GDI2 PE=1 SV=2 | 65,26 | 65,27 | 49 | 77,53 |
| 95 | sp|P11586|C1TC_HUMAN | HUMAN | C-1-tetrahydrofolate synthase, cytoplasmic OS=Homo sapiens GN=MTHFD1 PE=1 SV=3 | 65,13 | 65,15 | 37 | 49,95 |
| 96 | sp|P34932|HSP74_HUMAN | HUMAN | Heat shock 70 kDa protein 4 OS=Homo sapiens GN=HSPA4 PE=1 SV=4 | 64,91 | 69,38 | 38 | 52,98 |
| 97 | sp|P01833|PIGR_HUMAN | HUMAN | Polymeric immunoglobulin receptor OS=Homo sapiens GN=PIGR PE=1 SV=4 | 64,9 | 64,95 | 55 | 57,59 |
| 98 | sp|P07437|TBB5_HUMAN | HUMAN | Tubulin beta chain OS=Homo sapiens GN=TUBB PE=1 SV=2 | 64,77 | 64,8 | 108 | 84,46 |
| 99 | sp|P48643|TCPE_HUMAN | HUMAN | T-complex protein 1 subunit epsilon OS=Homo sapiens GN=CCT5 PE=1 SV=1 | 64,49 | 65,06 | 38 | 69,13 |
| 100 | sp|P12956|XRCC6_HUMAN | HUMAN | X-ray repair cross-complementing protein 6 OS=Homo sapiens GN=XRCC6 PE=1 SV=2 | 63,91 | 63,96 | 40 | 52,05 |
| 101 | sp|Q9NZN4|EHD2_HUMAN | HUMAN | EH domain-containing protein 2 OS=Homo sapiens GN=EHD2 PE=1 SV=2 | 63,28 | 63,35 | 38 | 77,35 |
| 102 | sp|O75083|WDR1_HUMAN | HUMAN | WD repeat-containing protein 1 OS=Homo sapiens GN=WDR1 PE=1 SV=4 | 63,09 | 63,13 | 43 | 70,13 |
| 103 | sp|P38646|GRP75_HUMAN | HUMAN | Stress-70 protein, mitochondrial OS=Homo sapiens GN=HSPA9 PE=1 SV=2 | 63,04 | 64,05 | 46 | 55,23 |
| 104 | sp|P27797|CALR_HUMAN | HUMAN | Calreticulin OS=Homo sapiens GN=CALR PE=1 SV=1 | 62,89 | 62,9 | 84 | 78,18 |
| 105 | sp|P48147|PPCE_HUMAN | HUMAN | Prolyl endopeptidase OS=Homo sapiens GN=PREP PE=1 SV=2 | 62,81 | 62,84 | 33 | 59,44 |
| 106 | sp|Q04637|IF4G1_HUMAN | HUMAN | Eukaryotic translation initiation factor 4 gamma 1 OS=Homo sapiens GN=EIF4G1 PE=1 SV=4 | 62,73 | 62,87 | 33 | 21,76 |
| 107 | sp|P02675|FIBB_HUMAN | HUMAN | Fibrinogen beta chain OS=Homo sapiens GN=FGB PE=1 SV=2 | 62,69 | 62,72 | 57 | 73,73 |
| 108 | sp|Q00341|VIGLN_HUMAN | HUMAN | Vigilin OS=Homo sapiens GN=HDLBP PE=1 SV=2 | 62,28 | 62,54 | 33 | 32,73 |
| 109 | sp|P17987|TCPA_HUMAN | HUMAN | T-complex protein 1 subunit alpha OS=Homo sapiens GN=TCP1 PE=1 SV=1 | 62,11 | 62,15 | 39 | 63,13 |
| 110 | sp|Q06210|GFPT1_HUMAN | HUMAN | Glutamine--fructose-6-phosphate aminotransferase [isomerizing] 1 OS=Homo sapiens GN=GFPT1 PE=1 SV=3 | 61,98 | 62,14 | 37 | 55,65 |
| 111 | sp|P55060|XPO2_HUMAN | HUMAN | Exportin-2 OS=Homo sapiens GN=CSE1L PE=1 SV=3 | 61,36 | 61,39 | 33 | 36,05 |
| 112 | sp|P49588|SYAC_HUMAN | HUMAN | Alanine--tRNA ligase, cytoplasmic OS=Homo sapiens GN=AARS PE=1 SV=2 | 61,1 | 61,13 | 35 | 46,18 |
| 113 | sp|O43491|E41L2_HUMAN | HUMAN | Band 4.1-like protein 2 OS=Homo sapiens GN=EPB41L2 PE=1 SV=1 | 60,62 | 60,67 | 30 | 38,21 |
| 114 | sp|P13010|XRCC5_HUMAN | HUMAN | X-ray repair cross-complementing protein 5 OS=Homo sapiens GN=XRCC5 PE=1 SV=3 | 60,55 | 60,65 | 42 | 54,37 |
| 115 | sp|Q9Y4L1|HYOU1_HUMAN | HUMAN | Hypoxia up-regulated protein 1 OS=Homo sapiens GN=HYOU1 PE=1 SV=1 | 60,52 | 60,59 | 31 | 38,24 |
| 116 | sp|P35900|K1C20_HUMAN | HUMAN | Keratin, type I cytoskeletal 20 OS=Homo sapiens GN=KRT20 PE=1 SV=1 | 60,51 | 62,59 | 44 | 66,98 |
| 117 | sp|O75643|U520_HUMAN | HUMAN | U5 small nuclear ribonucleoprotein 200 kDa helicase OS=Homo sapiens GN=SNRNP200 PE=1 SV=2 | 60,44 | 60,59 | 31 | 19,01 |
| 118 | sp|Q9Y678|COPG1_HUMAN | HUMAN | Coatomer subunit gamma-1 OS=Homo sapiens GN=COPG1 PE=1 SV=1 | 60,39 | 61,63 | 35 | 50,23 |
| 119 | sp|P12277|KCRB_HUMAN | HUMAN | Creatine kinase B-type OS=Homo sapiens GN=CKB PE=1 SV=1 | 60,26 | 60,28 | 78 | 82,68 |
| 120 | sp|P78371|TCPB_HUMAN | HUMAN | T-complex protein 1 subunit beta OS=Homo sapiens GN=CCT2 PE=1 SV=4 | 59,96 | 60,6 | 39 | 72,34 |
| 121 | sp|P09493|TPM1_HUMAN | HUMAN | Tropomyosin alpha-1 chain OS=Homo sapiens GN=TPM1 PE=1 SV=2 | 59,82 | 59,88 | 64 | 60,92 |
| 122 | sp|P30153|2AAA_HUMAN | HUMAN | Serine/threonine-protein phosphatase 2A 65 kDa regulatory subunit A alpha isoform OS=Homo sapiens GN=PPP2R1A PE=1 SV=4 | 59,22 | 59,3 | 38 | 59,25 |
| 123 | sp|Q7Z406|MYH14_HUMAN | HUMAN | Myosin-14 OS=Homo sapiens GN=MYH14 PE=1 SV=2 | 59,01 | 85,9 | 53 | 24,21 |
| 124 | sp|Q86UP2|KTN1_HUMAN | HUMAN | Kinectin OS=Homo sapiens GN=KTN1 PE=1 SV=1 | 58,74 | 58,83 | 29 | 26,16 |
| 125 | sp|Q12864|CAD17_HUMAN | HUMAN | Cadherin-17 OS=Homo sapiens GN=CDH17 PE=2 SV=3 | 58,65 | 58,7 | 35 | 46,51 |
| 126 | sp|P35221|CTNA1_HUMAN | HUMAN | Catenin alpha-1 OS=Homo sapiens GN=CTNNA1 PE=1 SV=1 | 58,57 | 58,6 | 37 | 45,47 |
| 127 | sp|P00558|PGK1_HUMAN | HUMAN | Phosphoglycerate kinase 1 OS=Homo sapiens GN=PGK1 PE=1 SV=3 | 58,37 | 58,37 | 74 | 71,94 |
| 128 | sp|P20810|ICAL_HUMAN | HUMAN | Calpastatin OS=Homo sapiens GN=CAST PE=1 SV=4 | 58,26 | 58,27 | 56 | 65,4 |
| 129 | sp|P49368|TCPG_HUMAN | HUMAN | T-complex protein 1 subunit gamma OS=Homo sapiens GN=CCT3 PE=1 SV=4 | 58,2 | 58,34 | 35 | 57,61 |
| 130 | sp|P43490|NAMPT_HUMAN | HUMAN | Nicotinamide phosphoribosyltransferase OS=Homo sapiens GN=NAMPT PE=1 SV=1 | 57,94 | 57,96 | 32 | 65,58 |
| 131 | sp|P22626|ROA2_HUMAN | HUMAN | Heterogeneous nuclear ribonucleoproteins A2/B1 OS=Homo sapiens GN=HNRNPA2B1 PE=1 SV=2 | 57,86 | 57,89 | 51 | 73,94 |
| 132 | sp|O00410|IPO5_HUMAN | HUMAN | Importin-5 OS=Homo sapiens GN=IPO5 PE=1 SV=4 | 57,8 | 57,82 | 40 | 37,37 |
| 133 | sp|P06744|G6PI_HUMAN | HUMAN | Glucose-6-phosphate isomerase OS=Homo sapiens GN=GPI PE=1 SV=4 | 57,78 | 58,89 | 59 | 63,98 |
| 134 | sp|Q96AC1|FERM2_HUMAN | HUMAN | Fermitin family homolog 2 OS=Homo sapiens GN=FERMT2 PE=1 SV=1 | 57,73 | 57,78 | 35 | 58,82 |
| 135 | sp|P0DMV9|HS71B_HUMAN | HUMAN | Heat shock 70 kDa protein 1B OS=Homo sapiens GN=HSPA1B PE=1 SV=1 | 57,71 | 67,32 | 90 | 63,96 |
| 136 | sp|P00450|CERU_HUMAN | HUMAN | Ceruloplasmin OS=Homo sapiens GN=CP PE=1 SV=1 | 57,14 | 57,21 | 34 | 37,84 |
| 137 | sp|P98160|PGBM_HUMAN | HUMAN | Basement membrane-specific heparan sulfate proteoglycan core protein OS=Homo sapiens GN=HSPG2 PE=1 SV=4 | 56,99 | 57,16 | 30 | 8,631 |
| 138 | sp|P00338|LDHA_HUMAN | HUMAN | L-lactate dehydrogenase A chain OS=Homo sapiens GN=LDHA PE=1 SV=2 | 56,93 | 57,01 | 72 | 81,93 |
| 139 | sp|P02679|FIBG_HUMAN | HUMAN | Fibrinogen gamma chain OS=Homo sapiens GN=FGG PE=1 SV=3 | 56,36 | 56,38 | 50 | 74,17 |
| 140 | sp|Q01518|CAP1_HUMAN | HUMAN | Adenylyl cyclase-associated protein 1 OS=Homo sapiens GN=CAP1 PE=1 SV=5 | 56,17 | 56,18 | 52 | 69,47 |
| 141 | sp|Q7Z6Z7|HUWE1_HUMAN | HUMAN | E3 ubiquitin-protein ligase HUWE1 OS=Homo sapiens GN=HUWE1 PE=1 SV=3 | 55,97 | 56,27 | 25 | 9,465 |
| 142 | sp|P28838|AMPL_HUMAN | HUMAN | Cytosol aminopeptidase OS=Homo sapiens GN=LAP3 PE=1 SV=3 | 55,65 | 55,66 | 34 | 67,05 |
| 143 | sp|P21399|ACOC_HUMAN | HUMAN | Cytoplasmic aconitate hydratase OS=Homo sapiens GN=ACO1 PE=1 SV=3 | 55,52 | 55,7 | 40 | 42,74 |
| 144 | sp|Q01995|TAGL_HUMAN | HUMAN | Transgelin OS=Homo sapiens GN=TAGLN PE=1 SV=4 | 55,34 | 55,4 | 126 | 93,03 |
| 145 | sp|P00751|CFAB_HUMAN | HUMAN | Complement factor B OS=Homo sapiens GN=CFB PE=1 SV=2 | 55,24 | 55,27 | 32 | 39,66 |
| 146 | sp|Q92945|FUBP2_HUMAN | HUMAN | Far upstream element-binding protein 2 OS=Homo sapiens GN=KHSRP PE=1 SV=4 | 55,16 | 56,19 | 39 | 60,06 |
| 147 | sp|P06733|ENOA_HUMAN | HUMAN | Alpha-enolase OS=Homo sapiens GN=ENO1 PE=1 SV=2 | 55 | 56,06 | 100 | 72,35 |
| 148 | sp|P05455|LA_HUMAN | HUMAN | Lupus La protein OS=Homo sapiens GN=SSB PE=1 SV=2 | 54,46 | 54,53 | 35 | 59,8 |
| 149 | sp|P07384|CAN1_HUMAN | HUMAN | Calpain-1 catalytic subunit OS=Homo sapiens GN=CAPN1 PE=1 SV=1 | 54,38 | 54,46 | 29 | 41,6 |
| 150 | sp|Q9H4A4|AMPB_HUMAN | HUMAN | Aminopeptidase B OS=Homo sapiens GN=RNPEP PE=1 SV=2 | 54,22 | 54,3 | 33 | 52,92 |
| 151 | sp|Q9Y3Z3|SAMH1_HUMAN | HUMAN | Deoxynucleoside triphosphate triphosphohydrolase SAMHD1 OS=Homo sapiens GN=SAMHD1 PE=1 SV=2 | 54,11 | 54,18 | 31 | 56,55 |
| 152 | sp|P11940|PABP1_HUMAN | HUMAN | Polyadenylate-binding protein 1 OS=Homo sapiens GN=PABPC1 PE=1 SV=2 | 54,06 | 54,2 | 35 | 52,99 |
| 153 | sp|Q02952|AKA12_HUMAN | HUMAN | A-kinase anchor protein 12 OS=Homo sapiens GN=AKAP12 PE=1 SV=4 | 53,89 | 54,43 | 27 | 24,52 |
| 154 | sp|P19367|HXK1_HUMAN | HUMAN | Hexokinase-1 OS=Homo sapiens GN=HK1 PE=1 SV=3 | 53,89 | 53,97 | 28 | 32,72 |
| 155 | sp|P22102|PUR2_HUMAN | HUMAN | Trifunctional purine biosynthetic protein adenosine-3 OS=Homo sapiens GN=GART PE=1 SV=1 | 53,5 | 53,53 | 27 | 35,94 |
| 156 | sp|P35606|COPB2_HUMAN | HUMAN | Coatomer subunit beta' OS=Homo sapiens GN=COPB2 PE=1 SV=2 | 53,26 | 53,38 | 37 | 43,93 |
| 157 | sp|P04406|G3P_HUMAN | HUMAN | Glyceraldehyde-3-phosphate dehydrogenase OS=Homo sapiens GN=GAPDH PE=1 SV=3 | 53,12 | 53,16 | 147 | 82,69 |
| 158 | sp|Q13263|TIF1B_HUMAN | HUMAN | Transcription intermediary factor 1-beta OS=Homo sapiens GN=TRIM28 PE=1 SV=5 | 53,01 | 52,99 | 33 | 54,61 |
| 159 | sp|Q12906|ILF3_HUMAN | HUMAN | Interleukin enhancer-binding factor 3 OS=Homo sapiens GN=ILF3 PE=1 SV=3 | 52,92 | 52,95 | 34 | 33,45 |
| 160 | sp|Q16555|DPYL2_HUMAN | HUMAN | Dihydropyrimidinase-related protein 2 OS=Homo sapiens GN=DPYSL2 PE=1 SV=1 | 52,65 | 52,7 | 38 | 76,57 |
| 161 | sp|O60506|HNRPQ_HUMAN | HUMAN | Heterogeneous nuclear ribonucleoprotein Q OS=Homo sapiens GN=SYNCRIP PE=1 SV=2 | 52,62 | 52,66 | 33 | 50,88 |
| 162 | sp|P41250|SYG_HUMAN | HUMAN | Glycine--tRNA ligase OS=Homo sapiens GN=GARS PE=1 SV=3 | 52,57 | 52,63 | 32 | 46,68 |
| 163 | sp|O94979|SC31A_HUMAN | HUMAN | Protein transport protein Sec31A OS=Homo sapiens GN=SEC31A PE=1 SV=3 | 51,99 | 52,11 | 30 | 25,66 |
| 164 | sp|P19338|NUCL_HUMAN | HUMAN | Nucleolin OS=Homo sapiens GN=NCL PE=1 SV=3 | 51,81 | 51,88 | 55 | 33,8 |
| 165 | sp|P63010|AP2B1_HUMAN | HUMAN | AP-2 complex subunit beta OS=Homo sapiens GN=AP2B1 PE=1 SV=1 | 51,72 | 51,94 | 31 | 38,42 |
| 166 | sp|P02647|APOA1_HUMAN | HUMAN | Apolipoprotein A-I OS=Homo sapiens GN=APOA1 PE=1 SV=1 | 51,7 | 51,73 | 55 | 80,15 |
| 167 | sp|Q96KP4|CNDP2_HUMAN | HUMAN | Cytosolic non-specific dipeptidase OS=Homo sapiens GN=CNDP2 PE=1 SV=2 | 51,37 | 51,39 | 33 | 70,32 |
| 168 | sp|P50991|TCPD_HUMAN | HUMAN | T-complex protein 1 subunit delta OS=Homo sapiens GN=CCT4 PE=1 SV=4 | 51,35 | 56,14 | 37 | 65,12 |
| 169 | sp|P09960|LKHA4_HUMAN | HUMAN | Leukotriene A-4 hydrolase OS=Homo sapiens GN=LTA4H PE=1 SV=2 | 51,33 | 51,39 | 29 | 53,85 |
| 170 | sp|Q13200|PSMD2_HUMAN | HUMAN | 26S proteasome non-ATPase regulatory subunit 2 OS=Homo sapiens GN=PSMD2 PE=1 SV=3 | 51,31 | 51,4 | 26 | 37,67 |
| 171 | sp|P05164|PERM_HUMAN | HUMAN | Myeloperoxidase OS=Homo sapiens GN=MPO PE=1 SV=1 | 51,21 | 51,34 | 34 | 45,5 |
| 172 | sp|Q9P2J5|SYLC_HUMAN | HUMAN | Leucine--tRNA ligase, cytoplasmic OS=Homo sapiens GN=LARS PE=1 SV=2 | 51,2 | 51,28 | 30 | 30,44 |
| 173 | sp|P00352|AL1A1_HUMAN | HUMAN | Retinal dehydrogenase 1 OS=Homo sapiens GN=ALDH1A1 PE=1 SV=2 | 51,15 | 51,18 | 38 | 63,67 |
| 174 | sp|Q9UHD8|SEPT9_HUMAN | HUMAN | Septin-9 OS=Homo sapiens GN=SEPT9 PE=1 SV=2 | 51,05 | 51,15 | 33 | 54,1 |
| 175 | sp|P01857|IGHG1_HUMAN | HUMAN | Ig gamma-1 chain C region OS=Homo sapiens GN=IGHG1 PE=1 SV=1 | 50,95 | 51,01 | 120 | 84,24 |
| 176 | sp|Q99460|PSMD1_HUMAN | HUMAN | 26S proteasome non-ATPase regulatory subunit 1 OS=Homo sapiens GN=PSMD1 PE=1 SV=2 | 50,78 | 50,93 | 29 | 36,1 |
| 177 | sp|Q13428|TCOF_HUMAN | HUMAN | Treacle protein OS=Homo sapiens GN=TCOF1 PE=1 SV=3 | 50,73 | 50,82 | 27 | 20,5 |
| 178 | sp|O14980|XPO1_HUMAN | HUMAN | Exportin-1 OS=Homo sapiens GN=XPO1 PE=1 SV=1 | 50,72 | 50,97 | 28 | 33,52 |
| 179 | sp|P36871|PGM1_HUMAN | HUMAN | Phosphoglucomutase-1 OS=Homo sapiens GN=PGM1 PE=1 SV=3 | 50,47 | 50,59 | 29 | 51,78 |
| 180 | sp|O75874|IDHC_HUMAN | HUMAN | Isocitrate dehydrogenase [NADP] cytoplasmic OS=Homo sapiens GN=IDH1 PE=1 SV=2 | 50,19 | 50,58 | 38 | 69,57 |
| 181 | sp|Q6P2Q9|PRP8_HUMAN | HUMAN | Pre-mRNA-processing-splicing factor 8 OS=Homo sapiens GN=PRPF8 PE=1 SV=2 | 50,16 | 50,97 | 22 | 10,84 |
| 182 | sp|P04040|CATA_HUMAN | HUMAN | Catalase OS=Homo sapiens GN=CAT PE=1 SV=3 | 50,07 | 50,1 | 33 | 63,38 |
| 183 | sp|Q96QK1|VPS35_HUMAN | HUMAN | Vacuolar protein sorting-associated protein 35 OS=Homo sapiens GN=VPS35 PE=1 SV=2 | 49,79 | 49,91 | 27 | 36,06 |
| 184 | sp|P50570|DYN2_HUMAN | HUMAN | Dynamin-2 OS=Homo sapiens GN=DNM2 PE=1 SV=2 | 49,72 | 49,88 | 27 | 35,52 |
| 185 | sp|O15061|SYNEM_HUMAN | HUMAN | Synemin OS=Homo sapiens GN=SYNM PE=1 SV=2 | 49,68 | 50,38 | 26 | 22,43 |
| 186 | sp|P52272|HNRPM_HUMAN | HUMAN | Heterogeneous nuclear ribonucleoprotein M OS=Homo sapiens GN=HNRNPM PE=1 SV=3 | 49,62 | 49,96 | 26 | 38,36 |
| 187 | sp|P42224|STAT1_HUMAN | HUMAN | Signal transducer and activator of transcription 1-alpha/beta OS=Homo sapiens GN=STAT1 PE=1 SV=2 | 49,42 | 49,49 | 30 | 43,33 |
| 188 | sp|P02774|VTDB_HUMAN | HUMAN | Vitamin D-binding protein OS=Homo sapiens GN=GC PE=1 SV=1 | 49,37 | 49,42 | 36 | 69,83 |
| 189 | sp|P41252|SYIC_HUMAN | HUMAN | Isoleucine--tRNA ligase, cytoplasmic OS=Homo sapiens GN=IARS PE=1 SV=2 | 49,36 | 49,51 | 28 | 28,13 |
| 190 | sp|P61978|HNRPK_HUMAN | HUMAN | Heterogeneous nuclear ribonucleoprotein K OS=Homo sapiens GN=HNRNPK PE=1 SV=1 | 49,19 | 49,22 | 72 | 70,84 |
| 191 | sp|P26641|EF1G_HUMAN | HUMAN | Elongation factor 1-gamma OS=Homo sapiens GN=EEF1G PE=1 SV=3 | 49,15 | 49,18 | 44 | 56,98 |
| 192 | sp|P40939|ECHA_HUMAN | HUMAN | Trifunctional enzyme subunit alpha, mitochondrial OS=Homo sapiens GN=HADHA PE=1 SV=2 | 49,07 | 49,11 | 25 | 42,99 |
| 193 | sp|P14868|SYDC_HUMAN | HUMAN | Aspartate--tRNA ligase, cytoplasmic OS=Homo sapiens GN=DARS PE=1 SV=2 | 49,03 | 49,5 | 29 | 58,08 |
| 194 | sp|Q99832|TCPH_HUMAN | HUMAN | T-complex protein 1 subunit eta OS=Homo sapiens GN=CCT7 PE=1 SV=2 | 48,97 | 49,24 | 29 | 57,83 |
| 195 | sp|Q9HBL0|TENS1_HUMAN | HUMAN | Tensin-1 OS=Homo sapiens GN=TNS1 PE=1 SV=2 | 48,86 | 49,44 | 27 | 22,42 |
| 196 | sp|P47897|SYQ_HUMAN | HUMAN | Glutamine--tRNA ligase OS=Homo sapiens GN=QARS PE=1 SV=1 | 48,78 | 48,87 | 31 | 42,06 |
| 197 | sp|Q92841|DDX17_HUMAN | HUMAN | Probable ATP-dependent RNA helicase DDX17 OS=Homo sapiens GN=DDX17 PE=1 SV=2 | 48,76 | 48,81 | 36 | 42,39 |
| 198 | sp|P23381|SYWC_HUMAN | HUMAN | Tryptophan--tRNA ligase, cytoplasmic OS=Homo sapiens GN=WARS PE=1 SV=2 | 48,35 | 48,44 | 46 | 73,25 |
| 199 | sp|O60701|UGDH_HUMAN | HUMAN | UDP-glucose 6-dehydrogenase OS=Homo sapiens GN=UGDH PE=1 SV=1 | 48,21 | 48,21 | 31 | 65,59 |
| 200 | sp|P54136|SYRC_HUMAN | HUMAN | Arginine--tRNA ligase, cytoplasmic OS=Homo sapiens GN=RARS PE=1 SV=2 | 48,05 | 48,57 | 27 | 46,06 |
| 201 | sp|P52209|6PGD_HUMAN | HUMAN | 6-phosphogluconate dehydrogenase, decarboxylating OS=Homo sapiens GN=PGD PE=1 SV=3 | 47,82 | 47,83 | 35 | 57,35 |
| 202 | sp|Q15124|PGM5_HUMAN | HUMAN | Phosphoglucomutase-like protein 5 OS=Homo sapiens GN=PGM5 PE=1 SV=2 | 47,79 | 47,92 | 29 | 63,14 |
| 203 | sp|Q93052|LPP_HUMAN | HUMAN | Lipoma-preferred partner OS=Homo sapiens GN=LPP PE=1 SV=1 | 47,63 | 47,65 | 45 | 76,8 |
| 204 | sp|Q8WX93|PALLD_HUMAN | HUMAN | Palladin OS=Homo sapiens GN=PALLD PE=1 SV=3 | 47,46 | 47,5 | 28 | 25,23 |
| 205 | sp|O60763|USO1_HUMAN | HUMAN | General vesicular transport factor p115 OS=Homo sapiens GN=USO1 PE=1 SV=2 | 47,43 | 47,5 | 28 | 36,59 |
| 206 | sp|Q16531|DDB1_HUMAN | HUMAN | DNA damage-binding protein 1 OS=Homo sapiens GN=DDB1 PE=1 SV=1 | 47,4 | 47,79 | 25 | 29,21 |
| 207 | sp|P17858|PFKAL_HUMAN | HUMAN | ATP-dependent 6-phosphofructokinase, liver type OS=Homo sapiens GN=PFKL PE=1 SV=6 | 47,3 | 47,36 | 27 | 43,21 |
| 208 | sp|O94832|MYO1D_HUMAN | HUMAN | Unconventional myosin-Id OS=Homo sapiens GN=MYO1D PE=1 SV=2 | 47,05 | 47,08 | 23 | 24,55 |
| 209 | sp|P07355|ANXA2_HUMAN | HUMAN | Annexin A2 OS=Homo sapiens GN=ANXA2 PE=1 SV=2 | 47,01 | 47,17 | 35 | 71,39 |
| 210 | sp|P30740|ILEU_HUMAN | HUMAN | Leukocyte elastase inhibitor OS=Homo sapiens GN=SERPINB1 PE=1 SV=1 | 47 | 47,03 | 36 | 73,09 |
| 211 | sp|P15311|EZRI_HUMAN | HUMAN | Ezrin OS=Homo sapiens GN=EZR PE=1 SV=4 | 46,77 | 71,32 | 51 | 54,1 |
| 212 | sp|O75533|SF3B1_HUMAN | HUMAN | Splicing factor 3B subunit 1 OS=Homo sapiens GN=SF3B1 PE=1 SV=3 | 46,54 | 46,6 | 22 | 22,47 |
| 213 | sp|Q02790|FKBP4_HUMAN | HUMAN | Peptidyl-prolyl cis-trans isomerase FKBP4 OS=Homo sapiens GN=FKBP4 PE=1 SV=3 | 46,47 | 46,57 | 27 | 65,14 |
| 214 | sp|Q06323|PSME1_HUMAN | HUMAN | Proteasome activator complex subunit 1 OS=Homo sapiens GN=PSME1 PE=1 SV=1 | 46,36 | 46,42 | 46 | 71,08 |
| 215 | sp|P26639|SYTC_HUMAN | HUMAN | Threonine--tRNA ligase, cytoplasmic OS=Homo sapiens GN=TARS PE=1 SV=3 | 46,35 | 46,4 | 28 | 40,39 |
| 216 | sp|P25705|ATPA_HUMAN | HUMAN | ATP synthase subunit alpha, mitochondrial OS=Homo sapiens GN=ATP5A1 PE=1 SV=1 | 45,99 | 46,86 | 28 | 47,02 |
| 217 | sp|P54577|SYYC_HUMAN | HUMAN | Tyrosine--tRNA ligase, cytoplasmic OS=Homo sapiens GN=YARS PE=1 SV=4 | 45,73 | 46,34 | 23 | 46,97 |
| 218 | sp|P60174|TPIS_HUMAN | HUMAN | Triosephosphate isomerase OS=Homo sapiens GN=TPI1 PE=1 SV=3 | 45,65 | 45,72 | 85 | 76,92 |
| 219 | sp|Q7L576|CYFP1_HUMAN | HUMAN | Cytoplasmic FMR1-interacting protein 1 OS=Homo sapiens GN=CYFIP1 PE=1 SV=1 | 45,55 | 45,63 | 26 | 22,51 |
| 220 | sp|P62258|1433E_HUMAN | HUMAN | 14-3-3 protein epsilon OS=Homo sapiens GN=YWHAE PE=1 SV=1 | 45,39 | 45,42 | 55 | 80,78 |
| 221 | sp|P31146|COR1A_HUMAN | HUMAN | Coronin-1A OS=Homo sapiens GN=CORO1A PE=1 SV=4 | 45,29 | 45,33 | 36 | 57,48 |
| 222 | sp|Q15063|POSTN_HUMAN | HUMAN | Periostin OS=Homo sapiens GN=POSTN PE=1 SV=2 | 45,14 | 45,24 | 32 | 41,63 |
| 223 | sp|P22234|PUR6_HUMAN | HUMAN | Multifunctional protein ADE2 OS=Homo sapiens GN=PAICS PE=1 SV=3 | 44,92 | 44,94 | 25 | 58,35 |
| 224 | sp|Q14974|IMB1_HUMAN | HUMAN | Importin subunit beta-1 OS=Homo sapiens GN=KPNB1 PE=1 SV=2 | 44,71 | 44,98 | 34 | 40,75 |
| 225 | sp|Q8NBS9|TXND5_HUMAN | HUMAN | Thioredoxin domain-containing protein 5 OS=Homo sapiens GN=TXNDC5 PE=1 SV=2 | 44,65 | 48,88 | 40 | 71,99 |
| 226 | sp|Q15181|IPYR_HUMAN | HUMAN | Inorganic pyrophosphatase OS=Homo sapiens GN=PPA1 PE=1 SV=2 | 44,52 | 44,54 | 44 | 82,7 |
| 227 | sp|P19971|TYPH_HUMAN | HUMAN | Thymidine phosphorylase OS=Homo sapiens GN=TYMP PE=1 SV=2 | 44,49 | 44,54 | 36 | 73,44 |
| 228 | sp|Q14195|DPYL3_HUMAN | HUMAN | Dihydropyrimidinase-related protein 3 OS=Homo sapiens GN=DPYSL3 PE=1 SV=1 | 44,48 | 51,87 | 40 | 67,02 |
| 229 | sp|P08758|ANXA5_HUMAN | HUMAN | Annexin A5 OS=Homo sapiens GN=ANXA5 PE=1 SV=2 | 44,38 | 44,4 | 30 | 70 |
| 230 | sp|Q14651|PLSI_HUMAN | HUMAN | Plastin-1 OS=Homo sapiens GN=PLS1 PE=1 SV=2 | 44,23 | 50,78 | 32 | 52,78 |
| 231 | sp|P05023|AT1A1_HUMAN | HUMAN | Sodium/potassium-transporting ATPase subunit alpha-1 OS=Homo sapiens GN=ATP1A1 PE=1 SV=1 | 44,05 | 44,15 | 25 | 28,64 |
| 232 | sp|P06576|ATPB_HUMAN | HUMAN | ATP synthase subunit beta, mitochondrial OS=Homo sapiens GN=ATP5B PE=1 SV=3 | 43,98 | 44,02 | 33 | 62,57 |
| 233 | sp|P00325|ADH1B_HUMAN | HUMAN | Alcohol dehydrogenase 1B OS=Homo sapiens GN=ADH1B PE=1 SV=2 | 43,82 | 43,84 | 38 | 69,07 |
| 234 | sp|O00429|DNM1L_HUMAN | HUMAN | Dynamin-1-like protein OS=Homo sapiens GN=DNM1L PE=1 SV=2 | 43,77 | 43,81 | 22 | 39,95 |
| 235 | sp|Q96G03|PGM2_HUMAN | HUMAN | Phosphoglucomutase-2 OS=Homo sapiens GN=PGM2 PE=1 SV=4 | 43,74 | 43,83 | 25 | 44,77 |
| 236 | sp|O43143|DHX15_HUMAN | HUMAN | Pre-mRNA-splicing factor ATP-dependent RNA helicase DHX15 OS=Homo sapiens GN=DHX15 PE=1 SV=2 | 43,55 | 43,72 | 22 | 32,2 |
| 237 | sp|P48735|IDHP_HUMAN | HUMAN | Isocitrate dehydrogenase [NADP], mitochondrial OS=Homo sapiens GN=IDH2 PE=1 SV=2 | 43,39 | 46,12 | 29 | 59,73 |
| 238 | sp|Q9NZ08|ERAP1_HUMAN | HUMAN | Endoplasmic reticulum aminopeptidase 1 OS=Homo sapiens GN=ERAP1 PE=1 SV=3 | 43,37 | 43,41 | 21 | 28,48 |
| 239 | sp|P60842|IF4A1_HUMAN | HUMAN | Eukaryotic initiation factor 4A-I OS=Homo sapiens GN=EIF4A1 PE=1 SV=1 | 43,35 | 43,38 | 34 | 51,72 |
| 240 | sp|Q16181|SEPT7_HUMAN | HUMAN | Septin-7 OS=Homo sapiens GN=SEPT7 PE=1 SV=2 | 43,27 | 45,25 | 25 | 54,46 |
| 241 | sp|Q9UHB6|LIMA1_HUMAN | HUMAN | LIM domain and actin-binding protein 1 OS=Homo sapiens GN=LIMA1 PE=1 SV=1 | 43,22 | 43,36 | 21 | 35,18 |
| 242 | sp|P13489|RINI_HUMAN | HUMAN | Ribonuclease inhibitor OS=Homo sapiens GN=RNH1 PE=1 SV=2 | 43,1 | 43,4 | 29 | 69,63 |
| 243 | sp|Q14847|LASP1_HUMAN | HUMAN | LIM and SH3 domain protein 1 OS=Homo sapiens GN=LASP1 PE=1 SV=2 | 42,85 | 42,98 | 41 | 63,22 |
| 244 | sp|P09874|PARP1_HUMAN | HUMAN | Poly [ADP-ribose] polymerase 1 OS=Homo sapiens GN=PARP1 PE=1 SV=4 | 42,77 | 42,98 | 23 | 31,46 |
| 245 | sp|O15144|ARPC2_HUMAN | HUMAN | Actin-related protein 2/3 complex subunit 2 OS=Homo sapiens GN=ARPC2 PE=1 SV=1 | 42,6 | 42,67 | 29 | 73,33 |
| 246 | sp|Q14247|SRC8_HUMAN | HUMAN | Src substrate cortactin OS=Homo sapiens GN=CTTN PE=1 SV=2 | 42,59 | 42,66 | 24 | 45,64 |
| 247 | sp|P62701|RS4X_HUMAN | HUMAN | 40S ribosomal protein S4, X isoform OS=Homo sapiens GN=RPS4X PE=1 SV=2 | 42,59 | 42,62 | 28 | 69,2 |
| 248 | sp|Q13838|DX39B_HUMAN | HUMAN | Spliceosome RNA helicase DDX39B OS=Homo sapiens GN=DDX39B PE=1 SV=1 | 42,47 | 42,51 | 30 | 60,98 |
| 249 | sp|P51911|CNN1_HUMAN | HUMAN | Calponin-1 OS=Homo sapiens GN=CNN1 PE=1 SV=2 | 42,38 | 42,5 | 70 | 82,49 |
| 250 | sp|P49748|ACADV_HUMAN | HUMAN | Very long-chain specific acyl-CoA dehydrogenase, mitochondrial OS=Homo sapiens GN=ACADVL PE=1 SV=1 | 42,32 | 42,39 | 23 | 47,18 |
| 251 | sp|P50851|LRBA_HUMAN | HUMAN | Lipopolysaccharide-responsive and beige-like anchor protein OS=Homo sapiens GN=LRBA PE=1 SV=4 | 42,06 | 42,21 | 22 | 9,675 |
| 252 | sp|P26599|PTBP1_HUMAN | HUMAN | Polypyrimidine tract-binding protein 1 OS=Homo sapiens GN=PTBP1 PE=1 SV=1 | 41,88 | 41,92 | 58 | 69,87 |
| 253 | sp|P14923|PLAK_HUMAN | HUMAN | Junction plakoglobin OS=Homo sapiens GN=JUP PE=1 SV=3 | 41,84 | 41,99 | 21 | 38,52 |
| 254 | sp|P06748|NPM_HUMAN | HUMAN | Nucleophosmin OS=Homo sapiens GN=NPM1 PE=1 SV=2 | 41,73 | 41,75 | 62 | 79,93 |
| 255 | sp|P00747|PLMN_HUMAN | HUMAN | Plasminogen OS=Homo sapiens GN=PLG PE=1 SV=2 | 41,72 | 41,77 | 23 | 39,75 |
| 256 | sp|Q6P996|PDXD1_HUMAN | HUMAN | Pyridoxal-dependent decarboxylase domain-containing protein 1 OS=Homo sapiens GN=PDXDC1 PE=1 SV=2 | 41,61 | 41,66 | 23 | 39,59 |
| 257 | sp|P11413|G6PD_HUMAN | HUMAN | Glucose-6-phosphate 1-dehydrogenase OS=Homo sapiens GN=G6PD PE=1 SV=4 | 41,55 | 41,6 | 26 | 54,56 |
| 258 | sp|P48444|COPD_HUMAN | HUMAN | Coatomer subunit delta OS=Homo sapiens GN=ARCN1 PE=1 SV=1 | 41,46 | 41,5 | 21 | 42,07 |
| 259 | sp|P25786|PSA1_HUMAN | HUMAN | Proteasome subunit alpha type-1 OS=Homo sapiens GN=PSMA1 PE=1 SV=1 | 41,4 | 42,52 | 26 | 76,05 |
| 260 | sp|P14550|AK1A1_HUMAN | HUMAN | Alcohol dehydrogenase [NADP(+)] OS=Homo sapiens GN=AKR1A1 PE=1 SV=3 | 41,35 | 41,7 | 30 | 74,15 |
| 261 | sp|P17655|CAN2_HUMAN | HUMAN | Calpain-2 catalytic subunit OS=Homo sapiens GN=CAPN2 PE=1 SV=6 | 41,25 | 41,33 | 25 | 40,14 |
| 262 | sp|P62195|PRS8_HUMAN | HUMAN | 26S protease regulatory subunit 8 OS=Homo sapiens GN=PSMC5 PE=1 SV=1 | 41,19 | 41,29 | 25 | 60,1 |
| 263 | sp|P62736|ACTA_HUMAN | HUMAN | Actin, aortic smooth muscle OS=Homo sapiens GN=ACTA2 PE=1 SV=1 | 41,17 | 68,96 | 225 | 79,84 |
| 264 | sp|Q9UQ80|PA2G4_HUMAN | HUMAN | Proliferation-associated protein 2G4 OS=Homo sapiens GN=PA2G4 PE=1 SV=3 | 41,15 | 41,2 | 28 | 62,18 |
| 265 | sp|O00151|PDLI1_HUMAN | HUMAN | PDZ and LIM domain protein 1 OS=Homo sapiens GN=PDLIM1 PE=1 SV=4 | 41,1 | 41,18 | 37 | 83,59 |
| 266 | sp|P40227|TCPZ_HUMAN | HUMAN | T-complex protein 1 subunit zeta OS=Homo sapiens GN=CCT6A PE=1 SV=3 | 41,01 | 41,11 | 26 | 45,76 |
| 267 | sp|P00367|DHE3_HUMAN | HUMAN | Glutamate dehydrogenase 1, mitochondrial OS=Homo sapiens GN=GLUD1 PE=1 SV=2 | 40,97 | 41,34 | 26 | 47,49 |
| 268 | sp|P23526|SAHH_HUMAN | HUMAN | Adenosylhomocysteinase OS=Homo sapiens GN=AHCY PE=1 SV=4 | 40,96 | 41,02 | 30 | 49,07 |
| 269 | sp|P02790|HEMO_HUMAN | HUMAN | Hemopexin OS=Homo sapiens GN=HPX PE=1 SV=2 | 40,92 | 40,98 | 47 | 65,58 |
| 270 | sp|P23528|COF1_HUMAN | HUMAN | Cofilin-1 OS=Homo sapiens GN=CFL1 PE=1 SV=3 | 40,84 | 40,96 | 69 | 87,35 |
| 271 | sp|P24821|TENA_HUMAN | HUMAN | Tenascin OS=Homo sapiens GN=TNC PE=1 SV=3 | 40,83 | 40,97 | 20 | 14,86 |
| 272 | sp|P67936|TPM4_HUMAN | HUMAN | Tropomyosin alpha-4 chain OS=Homo sapiens GN=TPM4 PE=1 SV=3 | 40,77 | 54,59 | 82 | 70,97 |
| 273 | sp|P01876|IGHA1_HUMAN | HUMAN | Ig alpha-1 chain C region OS=Homo sapiens GN=IGHA1 PE=1 SV=2 | 40,6 | 40,69 | 81 | 87,25 |
| 274 | sp|P61158|ARP3_HUMAN | HUMAN | Actin-related protein 3 OS=Homo sapiens GN=ACTR3 PE=1 SV=3 | 40,54 | 40,6 | 29 | 62,92 |
| 275 | sp|O60664|PLIN3_HUMAN | HUMAN | Perilipin-3 OS=Homo sapiens GN=PLIN3 PE=1 SV=3 | 40,51 | 40,67 | 34 | 69,59 |
| 276 | sp|P17174|AATC_HUMAN | HUMAN | Aspartate aminotransferase, cytoplasmic OS=Homo sapiens GN=GOT1 PE=1 SV=3 | 40,47 | 40,62 | 28 | 67,8 |
| 277 | sp|P37837|TALDO_HUMAN | HUMAN | Transaldolase OS=Homo sapiens GN=TALDO1 PE=1 SV=2 | 40,43 | 40,62 | 28 | 53,71 |
| 278 | sp|P06727|APOA4_HUMAN | HUMAN | Apolipoprotein A-IV OS=Homo sapiens GN=APOA4 PE=1 SV=3 | 40,38 | 40,57 | 23 | 54,04 |
| 279 | sp|Q15029|U5S1_HUMAN | HUMAN | 116 kDa U5 small nuclear ribonucleoprotein component OS=Homo sapiens GN=EFTUD2 PE=1 SV=1 | 40,35 | 42,7 | 21 | 30,14 |
| 280 | sp|Q13576|IQGA2_HUMAN | HUMAN | Ras GTPase-activating-like protein IQGAP2 OS=Homo sapiens GN=IQGAP2 PE=1 SV=4 | 40,3 | 48,24 | 27 | 19,94 |
| 281 | sp|P39023|RL3_HUMAN | HUMAN | 60S ribosomal protein L3 OS=Homo sapiens GN=RPL3 PE=1 SV=2 | 40,29 | 40,55 | 30 | 47,15 |
| 282 | sp|O00159|MYO1C_HUMAN | HUMAN | Unconventional myosin-Ic OS=Homo sapiens GN=MYO1C PE=1 SV=4 | 40,22 | 40,94 | 22 | 27,66 |
| 283 | sp|P29692|EF1D_HUMAN | HUMAN | Elongation factor 1-delta OS=Homo sapiens GN=EEF1D PE=1 SV=5 | 40,18 | 40,22 | 32 | 79,36 |
| 284 | sp|P49411|EFTU_HUMAN | HUMAN | Elongation factor Tu, mitochondrial OS=Homo sapiens GN=TUFM PE=1 SV=2 | 40,12 | 40,73 | 24 | 56,86 |
| 285 | sp|Q92499|DDX1_HUMAN | HUMAN | ATP-dependent RNA helicase DDX1 OS=Homo sapiens GN=DDX1 PE=1 SV=2 | 40,09 | 40,33 | 22 | 35,81 |
| 286 | sp|P05091|ALDH2_HUMAN | HUMAN | Aldehyde dehydrogenase, mitochondrial OS=Homo sapiens GN=ALDH2 PE=1 SV=2 | 40,05 | 42,08 | 26 | 62,48 |
| 287 | sp|Q96AE4|FUBP1_HUMAN | HUMAN | Far upstream element-binding protein 1 OS=Homo sapiens GN=FUBP1 PE=1 SV=3 | 39,78 | 45,65 | 33 | 48,29 |
| 288 | sp|Q8N1G4|LRC47_HUMAN | HUMAN | Leucine-rich repeat-containing protein 47 OS=Homo sapiens GN=LRRC47 PE=1 SV=1 | 39,76 | 39,84 | 24 | 57,46 |
| 289 | sp|O75116|ROCK2_HUMAN | HUMAN | Rho-associated protein kinase 2 OS=Homo sapiens GN=ROCK2 PE=1 SV=4 | 39,5 | 40,5 | 20 | 14,48 |
| 290 | sp|Q16851|UGPA_HUMAN | HUMAN | UTP--glucose-1-phosphate uridylyltransferase OS=Homo sapiens GN=UGP2 PE=1 SV=5 | 39,47 | 39,64 | 25 | 48,23 |
| 291 | sp|Q15046|SYK_HUMAN | HUMAN | Lysine--tRNA ligase OS=Homo sapiens GN=KARS PE=1 SV=3 | 39,46 | 39,56 | 19 | 35,18 |
| 292 | sp|Q9UGI8|TES_HUMAN | HUMAN | Testin OS=Homo sapiens GN=TES PE=1 SV=1 | 39,44 | 39,58 | 20 | 52,26 |
| 293 | sp|Q12931|TRAP1_HUMAN | HUMAN | Heat shock protein 75 kDa, mitochondrial OS=Homo sapiens GN=TRAP1 PE=1 SV=3 | 39,33 | 39,42 | 22 | 39,06 |
| 294 | sp|P43243|MATR3_HUMAN | HUMAN | Matrin-3 OS=Homo sapiens GN=MATR3 PE=1 SV=2 | 39,22 | 39,56 | 28 | 32,7 |
| 295 | sp|Q9NY33|DPP3_HUMAN | HUMAN | Dipeptidyl peptidase 3 OS=Homo sapiens GN=DPP3 PE=1 SV=2 | 39,21 | 39,37 | 22 | 38,13 |
| 296 | sp|P04083|ANXA1_HUMAN | HUMAN | Annexin A1 OS=Homo sapiens GN=ANXA1 PE=1 SV=2 | 39,16 | 39,23 | 24 | 64,45 |
| 297 | sp|Q8N163|CCAR2_HUMAN | HUMAN | Cell cycle and apoptosis regulator protein 2 OS=Homo sapiens GN=CCAR2 PE=1 SV=2 | 39,1 | 39,3 | 21 | 37,7 |
| 298 | sp|P36578|RL4_HUMAN | HUMAN | 60S ribosomal protein L4 OS=Homo sapiens GN=RPL4 PE=1 SV=5 | 39,03 | 39,08 | 29 | 46,37 |
| 299 | sp|O43776|SYNC_HUMAN | HUMAN | Asparagine--tRNA ligase, cytoplasmic OS=Homo sapiens GN=NARS PE=1 SV=1 | 38,86 | 39,01 | 26 | 43,43 |
| 300 | sp|P50454|SERPH_HUMAN | HUMAN | Serpin H1 OS=Homo sapiens GN=SERPINH1 PE=1 SV=2 | 38,75 | 38,77 | 24 | 52,63 |
| 301 | sp|P00488|F13A_HUMAN | HUMAN | Coagulation factor XIII A chain OS=Homo sapiens GN=F13A1 PE=1 SV=4 | 38,65 | 38,7 | 21 | 36,2 |
| 302 | sp|P07195|LDHB_HUMAN | HUMAN | L-lactate dehydrogenase B chain OS=Homo sapiens GN=LDHB PE=1 SV=2 | 38,59 | 42,25 | 50 | 63,47 |
| 303 | sp|P40926|MDHM_HUMAN | HUMAN | Malate dehydrogenase, mitochondrial OS=Homo sapiens GN=MDH2 PE=1 SV=3 | 38,59 | 38,6 | 33 | 73,67 |
| 304 | sp|P54578|UBP14_HUMAN | HUMAN | Ubiquitin carboxyl-terminal hydrolase 14 OS=Homo sapiens GN=USP14 PE=1 SV=3 | 38,56 | 38,6 | 22 | 43,52 |
| 305 | sp|Q15393|SF3B3_HUMAN | HUMAN | Splicing factor 3B subunit 3 OS=Homo sapiens GN=SF3B3 PE=1 SV=4 | 38,55 | 38,62 | 19 | 17,91 |
| 306 | sp|P00966|ASSY_HUMAN | HUMAN | Argininosuccinate synthase OS=Homo sapiens GN=ASS1 PE=1 SV=2 | 38,53 | 38,57 | 31 | 67,23 |
| 307 | sp|O60841|IF2P_HUMAN | HUMAN | Eukaryotic translation initiation factor 5B OS=Homo sapiens GN=EIF5B PE=1 SV=4 | 38,52 | 39 | 22 | 22,7 |
| 308 | sp|O00231|PSD11_HUMAN | HUMAN | 26S proteasome non-ATPase regulatory subunit 11 OS=Homo sapiens GN=PSMD11 PE=1 SV=3 | 38,5 | 38,54 | 25 | 54,98 |
| 309 | sp|P00734|THRB_HUMAN | HUMAN | Prothrombin OS=Homo sapiens GN=F2 PE=1 SV=2 | 38,49 | 38,53 | 23 | 48,39 |
| 310 | sp|Q14624|ITIH4_HUMAN | HUMAN | Inter-alpha-trypsin inhibitor heavy chain H4 OS=Homo sapiens GN=ITIH4 PE=1 SV=4 | 38,47 | 38,52 | 25 | 30,43 |
| 311 | sp|P11678|PERE_HUMAN | HUMAN | Eosinophil peroxidase OS=Homo sapiens GN=EPX PE=1 SV=2 | 38,43 | 46,64 | 31 | 35,38 |
| 312 | sp|P49591|SYSC_HUMAN | HUMAN | Serine--tRNA ligase, cytoplasmic OS=Homo sapiens GN=SARS PE=1 SV=3 | 38,42 | 38,5 | 23 | 48,64 |
| 313 | sp|P23246|SFPQ_HUMAN | HUMAN | Splicing factor, proline- and glutamine-rich OS=Homo sapiens GN=SFPQ PE=1 SV=2 | 38,38 | 38,53 | 30 | 29,99 |
| 314 | sp|P31943|HNRH1_HUMAN | HUMAN | Heterogeneous nuclear ribonucleoprotein H OS=Homo sapiens GN=HNRNPH1 PE=1 SV=4 | 38,29 | 38,62 | 29 | 49,89 |
| 315 | sp|P00505|AATM_HUMAN | HUMAN | Aspartate aminotransferase, mitochondrial OS=Homo sapiens GN=GOT2 PE=1 SV=3 | 38,27 | 38,38 | 26 | 58,84 |
| 316 | sp|P09429|HMGB1_HUMAN | HUMAN | High mobility group protein B1 OS=Homo sapiens GN=HMGB1 PE=1 SV=3 | 38,1 | 38,2 | 51 | 51,63 |
| 317 | sp|P48637|GSHB_HUMAN | HUMAN | Glutathione synthetase OS=Homo sapiens GN=GSS PE=1 SV=1 | 38,01 | 38,26 | 24 | 53,38 |
| 318 | sp|O43242|PSMD3_HUMAN | HUMAN | 26S proteasome non-ATPase regulatory subunit 3 OS=Homo sapiens GN=PSMD3 PE=1 SV=2 | 38 | 38,14 | 19 | 38,95 |
| 319 | sp|P63244|RACK1_HUMAN | HUMAN | Receptor of activated protein C kinase 1 OS=Homo sapiens GN=RACK1 PE=1 SV=3 | 37,72 | 37,79 | 33 | 79,81 |
| 320 | sp|Q32MZ4|LRRF1_HUMAN | HUMAN | Leucine-rich repeat flightless-interacting protein 1 OS=Homo sapiens GN=LRRFIP1 PE=1 SV=2 | 37,6 | 37,68 | 22 | 38 |
| 321 | sp|Q96I99|SUCB2_HUMAN | HUMAN | Succinyl-CoA ligase [GDP-forming] subunit beta, mitochondrial OS=Homo sapiens GN=SUCLG2 PE=1 SV=2 | 37,59 | 37,64 | 22 | 55,56 |
| 322 | sp|Q9ULV4|COR1C_HUMAN | HUMAN | Coronin-1C OS=Homo sapiens GN=CORO1C PE=1 SV=1 | 37,4 | 37,56 | 26 | 46,41 |
| 323 | sp|P14314|GLU2B_HUMAN | HUMAN | Glucosidase 2 subunit beta OS=Homo sapiens GN=PRKCSH PE=1 SV=2 | 37,37 | 37,47 | 31 | 60,98 |
| 324 | sp|P45974|UBP5_HUMAN | HUMAN | Ubiquitin carboxyl-terminal hydrolase 5 OS=Homo sapiens GN=USP5 PE=1 SV=2 | 37,16 | 37,24 | 19 | 30,89 |
| 325 | sp|P07910|HNRPC_HUMAN | HUMAN | Heterogeneous nuclear ribonucleoproteins C1/C2 OS=Homo sapiens GN=HNRNPC PE=1 SV=4 | 37,03 | 37,11 | 40 | 61,44 |
| 326 | sp|P55884|EIF3B_HUMAN | HUMAN | Eukaryotic translation initiation factor 3 subunit B OS=Homo sapiens GN=EIF3B PE=1 SV=3 | 36,86 | 36,99 | 24 | 35,63 |
| 327 | sp|O60547|GMDS_HUMAN | HUMAN | GDP-mannose 4,6 dehydratase OS=Homo sapiens GN=GMDS PE=1 SV=1 | 36,85 | 36,9 | 21 | 60,75 |
| 328 | sp|P12270|TPR_HUMAN | HUMAN | Nucleoprotein TPR OS=Homo sapiens GN=TPR PE=1 SV=3 | 36,79 | 37,54 | 17 | 8,887 |
| 329 | sp|P37802|TAGL2_HUMAN | HUMAN | Transgelin-2 OS=Homo sapiens GN=TAGLN2 PE=1 SV=3 | 36,63 | 36,81 | 51 | 79,9 |
| 330 | sp|P01008|ANT3_HUMAN | HUMAN | Antithrombin-III OS=Homo sapiens GN=SERPINC1 PE=1 SV=1 | 36,28 | 36,39 | 21 | 51,08 |
| 331 | sp|Q99613|EIF3C_HUMAN | HUMAN | Eukaryotic translation initiation factor 3 subunit C OS=Homo sapiens GN=EIF3C PE=1 SV=1 | 36,28 | 36,3 | 19 | 25,19 |
| 332 | sp|Q9NYU2|UGGG1_HUMAN | HUMAN | UDP-glucose:glycoprotein glucosyltransferase 1 OS=Homo sapiens GN=UGGT1 PE=1 SV=3 | 36,15 | 37,45 | 20 | 15,76 |
| 333 | sp|P05198|IF2A_HUMAN | HUMAN | Eukaryotic translation initiation factor 2 subunit 1 OS=Homo sapiens GN=EIF2S1 PE=1 SV=3 | 36,04 | 36,05 | 18 | 60,63 |
| 334 | sp|P01042|KNG1_HUMAN | HUMAN | Kininogen-1 OS=Homo sapiens GN=KNG1 PE=1 SV=2 | 36,02 | 36,07 | 23 | 35,71 |
| 335 | sp|P63104|1433Z_HUMAN | HUMAN | 14-3-3 protein zeta/delta OS=Homo sapiens GN=YWHAZ PE=1 SV=1 | 35,94 | 41,99 | 76 | 80,82 |
| 336 | sp|Q02818|NUCB1_HUMAN | HUMAN | Nucleobindin-1 OS=Homo sapiens GN=NUCB1 PE=1 SV=4 | 35,91 | 35,94 | 22 | 47,72 |
| 337 | sp|Q96TA1|NIBL1_HUMAN | HUMAN | Niban-like protein 1 OS=Homo sapiens GN=FAM129B PE=1 SV=3 | 35,84 | 35,94 | 22 | 35,66 |
| 338 | sp|P62136|PP1A_HUMAN | HUMAN | Serine/threonine-protein phosphatase PP1-alpha catalytic subunit OS=Homo sapiens GN=PPP1CA PE=1 SV=1 | 35,84 | 35,84 | 22 | 69,7 |
| 339 | sp|P14866|HNRPL_HUMAN | HUMAN | Heterogeneous nuclear ribonucleoprotein L OS=Homo sapiens GN=HNRNPL PE=1 SV=2 | 35,71 | 35,81 | 27 | 54,33 |
| 340 | sp|P23142|FBLN1_HUMAN | HUMAN | Fibulin-1 OS=Homo sapiens GN=FBLN1 PE=1 SV=4 | 35,61 | 35,62 | 25 | 39,26 |
| 341 | sp|Q9Y696|CLIC4_HUMAN | HUMAN | Chloride intracellular channel protein 4 OS=Homo sapiens GN=CLIC4 PE=1 SV=4 | 35,6 | 35,64 | 21 | 72,73 |
| 342 | sp|Q15084|PDIA6_HUMAN | HUMAN | Protein disulfide-isomerase A6 OS=Homo sapiens GN=PDIA6 PE=1 SV=1 | 35,58 | 36,84 | 29 | 55,23 |
| 343 | sp|P19823|ITIH2_HUMAN | HUMAN | Inter-alpha-trypsin inhibitor heavy chain H2 OS=Homo sapiens GN=ITIH2 PE=1 SV=2 | 35,5 | 35,6 | 19 | 24 |
| 344 | sp|Q9UMS6|SYNP2_HUMAN | HUMAN | Synaptopodin-2 OS=Homo sapiens GN=SYNPO2 PE=1 SV=2 | 35,47 | 35,57 | 21 | 27,81 |
| 345 | sp|Q13435|SF3B2_HUMAN | HUMAN | Splicing factor 3B subunit 2 OS=Homo sapiens GN=SF3B2 PE=1 SV=2 | 35,43 | 35,53 | 19 | 26,7 |
| 346 | sp|Q15365|PCBP1_HUMAN | HUMAN | Poly(rC)-binding protein 1 OS=Homo sapiens GN=PCBP1 PE=1 SV=2 | 35,4 | 35,46 | 31 | 75,28 |
| 347 | sp|P56192|SYMC_HUMAN | HUMAN | Methionine--tRNA ligase, cytoplasmic OS=Homo sapiens GN=MARS PE=1 SV=2 | 35,35 | 35,44 | 22 | 32,67 |
| 348 | sp|Q8NC51|PAIRB_HUMAN | HUMAN | Plasminogen activator inhibitor 1 RNA-binding protein OS=Homo sapiens GN=SERBP1 PE=1 SV=2 | 35,33 | 35,41 | 27 | 51,23 |
| 349 | sp|O00232|PSD12_HUMAN | HUMAN | 26S proteasome non-ATPase regulatory subunit 12 OS=Homo sapiens GN=PSMD12 PE=1 SV=3 | 35,31 | 35,4 | 21 | 47,81 |
| 350 | sp|P23396|RS3_HUMAN | HUMAN | 40S ribosomal protein S3 OS=Homo sapiens GN=RPS3 PE=1 SV=2 | 35,27 | 35,3 | 25 | 79,84 |
| 351 | sp|Q16822|PCKGM_HUMAN | HUMAN | Phosphoenolpyruvate carboxykinase [GTP], mitochondrial OS=Homo sapiens GN=PCK2 PE=1 SV=3 | 35,18 | 35,3 | 20 | 40,78 |
| 352 | sp|O43852|CALU_HUMAN | HUMAN | Calumenin OS=Homo sapiens GN=CALU PE=1 SV=2 | 35,18 | 35,21 | 21 | 69,21 |
| 353 | sp|Q9NR30|DDX21_HUMAN | HUMAN | Nucleolar RNA helicase 2 OS=Homo sapiens GN=DDX21 PE=1 SV=5 | 35,04 | 35,23 | 18 | 31,55 |
| 354 | sp|Q15942|ZYX_HUMAN | HUMAN | Zyxin OS=Homo sapiens GN=ZYX PE=1 SV=1 | 35,01 | 35,04 | 30 | 53,32 |
| 355 | sp|P35237|SPB6_HUMAN | HUMAN | Serpin B6 OS=Homo sapiens GN=SERPINB6 PE=1 SV=3 | 34,93 | 35,03 | 21 | 54,26 |
| 356 | sp|Q86UX7|URP2_HUMAN | HUMAN | Fermitin family homolog 3 OS=Homo sapiens GN=FERMT3 PE=1 SV=1 | 34,87 | 37,07 | 21 | 36,58 |
| 357 | sp|Q9HC35|EMAL4_HUMAN | HUMAN | Echinoderm microtubule-associated protein-like 4 OS=Homo sapiens GN=EML4 PE=1 SV=3 | 34,75 | 34,83 | 18 | 22,83 |
| 358 | sp|P35998|PRS7_HUMAN | HUMAN | 26S protease regulatory subunit 7 OS=Homo sapiens GN=PSMC2 PE=1 SV=3 | 34,69 | 35,3 | 18 | 46,42 |
| 359 | sp|Q16658|FSCN1_HUMAN | HUMAN | Fascin OS=Homo sapiens GN=FSCN1 PE=1 SV=3 | 34,68 | 34,79 | 20 | 40,57 |
| 360 | sp|P42765|THIM_HUMAN | HUMAN | 3-ketoacyl-CoA thiolase, mitochondrial OS=Homo sapiens GN=ACAA2 PE=1 SV=2 | 34,61 | 34,67 | 24 | 67,76 |
| 361 | sp|Q9H223|EHD4_HUMAN | HUMAN | EH domain-containing protein 4 OS=Homo sapiens GN=EHD4 PE=1 SV=1 | 34,57 | 39,07 | 22 | 54,34 |
| 362 | sp|Q1KMD3|HNRL2_HUMAN | HUMAN | Heterogeneous nuclear ribonucleoprotein U-like protein 2 OS=Homo sapiens GN=HNRNPUL2 PE=1 SV=1 | 34,54 | 34,82 | 24 | 34,54 |
| 363 | sp|Q9NTK5|OLA1_HUMAN | HUMAN | Obg-like ATPase 1 OS=Homo sapiens GN=OLA1 PE=1 SV=2 | 34,49 | 34,54 | 20 | 59,85 |
| 364 | sp|Q9NSD9|SYFB_HUMAN | HUMAN | Phenylalanine--tRNA ligase beta subunit OS=Homo sapiens GN=FARSB PE=1 SV=3 | 34,42 | 34,61 | 16 | 30,56 |
| 365 | sp|Q00796|DHSO_HUMAN | HUMAN | Sorbitol dehydrogenase OS=Homo sapiens GN=SORD PE=1 SV=4 | 34,4 | 34,49 | 22 | 75,35 |
| 366 | sp|Q13418|ILK_HUMAN | HUMAN | Integrin-linked protein kinase OS=Homo sapiens GN=ILK PE=1 SV=2 | 34,38 | 34,44 | 17 | 44,25 |
| 367 | sp|P07996|TSP1_HUMAN | HUMAN | Thrombospondin-1 OS=Homo sapiens GN=THBS1 PE=1 SV=2 | 34,34 | 34,42 | 20 | 19,32 |
| 368 | sp|P53814|SMTN_HUMAN | HUMAN | Smoothelin OS=Homo sapiens GN=SMTN PE=1 SV=7 | 34,25 | 34,7 | 17 | 22,79 |
| 369 | sp|O95782|AP2A1_HUMAN | HUMAN | AP-2 complex subunit alpha-1 OS=Homo sapiens GN=AP2A1 PE=1 SV=3 | 34,12 | 34,69 | 17 | 20,78 |
| 370 | sp|P27824|CALX_HUMAN | HUMAN | Calnexin OS=Homo sapiens GN=CANX PE=1 SV=2 | 34,12 | 34,18 | 29 | 46,79 |
| 371 | sp|P41091|IF2G_HUMAN | HUMAN | Eukaryotic translation initiation factor 2 subunit 3 OS=Homo sapiens GN=EIF2S3 PE=1 SV=3 | 34,06 | 34,1 | 22 | 48,94 |
| 372 | sp|Q07960|RHG01_HUMAN | HUMAN | Rho GTPase-activating protein 1 OS=Homo sapiens GN=ARHGAP1 PE=1 SV=1 | 34,02 | 34,18 | 26 | 57,63 |
| 373 | sp|P49915|GUAA_HUMAN | HUMAN | GMP synthase [glutamine-hydrolyzing] OS=Homo sapiens GN=GMPS PE=1 SV=1 | 34,01 | 34,14 | 17 | 37,81 |
| 374 | sp|P20700|LMNB1_HUMAN | HUMAN | Lamin-B1 OS=Homo sapiens GN=LMNB1 PE=1 SV=2 | 33,96 | 37,11 | 19 | 38,05 |
| 375 | sp|Q13561|DCTN2_HUMAN | HUMAN | Dynactin subunit 2 OS=Homo sapiens GN=DCTN2 PE=1 SV=4 | 33,96 | 34 | 19 | 65,34 |
| 376 | sp|P16152|CBR1_HUMAN | HUMAN | Carbonyl reductase [NADPH] 1 OS=Homo sapiens GN=CBR1 PE=1 SV=3 | 33,94 | 33,99 | 26 | 77,62 |
| 377 | sp|Q9UJU6|DBNL_HUMAN | HUMAN | Drebrin-like protein OS=Homo sapiens GN=DBNL PE=1 SV=1 | 33,91 | 33,95 | 19 | 49,77 |
| 378 | sp|Q93009|UBP7_HUMAN | HUMAN | Ubiquitin carboxyl-terminal hydrolase 7 OS=Homo sapiens GN=USP7 PE=1 SV=2 | 33,8 | 33,93 | 18 | 19,6 |
| 379 | sp|P38606|VATA_HUMAN | HUMAN | V-type proton ATPase catalytic subunit A OS=Homo sapiens GN=ATP6V1A PE=1 SV=2 | 33,77 | 33,85 | 20 | 37,6 |
| 380 | sp|P62333|PRS10_HUMAN | HUMAN | 26S protease regulatory subunit 10B OS=Homo sapiens GN=PSMC6 PE=1 SV=1 | 33,67 | 33,76 | 16 | 54,76 |
| 381 | sp|P12081|SYHC_HUMAN | HUMAN | Histidine--tRNA ligase, cytoplasmic OS=Homo sapiens GN=HARS PE=1 SV=2 | 33,65 | 34,05 | 19 | 39,69 |
| 382 | sp|P51858|HDGF_HUMAN | HUMAN | Hepatoma-derived growth factor OS=Homo sapiens GN=HDGF PE=1 SV=1 | 33,62 | 33,69 | 28 | 76,67 |
| 383 | sp|P13797|PLST_HUMAN | HUMAN | Plastin-3 OS=Homo sapiens GN=PLS3 PE=1 SV=4 | 33,42 | 52,9 | 41 | 55,56 |
| 384 | sp|O95373|IPO7_HUMAN | HUMAN | Importin-7 OS=Homo sapiens GN=IPO7 PE=1 SV=1 | 33,37 | 33,4 | 23 | 22,35 |
| 385 | sp|P62937|PPIA_HUMAN | HUMAN | Peptidyl-prolyl cis-trans isomerase A OS=Homo sapiens GN=PPIA PE=1 SV=2 | 33,27 | 33,32 | 91 | 75,15 |
| 386 | sp|P00918|CAH2_HUMAN | HUMAN | Carbonic anhydrase 2 OS=Homo sapiens GN=CA2 PE=1 SV=2 | 33,17 | 33,2 | 36 | 74,23 |
| 387 | sp|P27695|APEX1_HUMAN | HUMAN | DNA-(apurinic or apyrimidinic site) lyase OS=Homo sapiens GN=APEX1 PE=1 SV=2 | 33,15 | 33,22 | 20 | 57,86 |
| 388 | sp|P17661|DESM_HUMAN | HUMAN | Desmin OS=Homo sapiens GN=DES PE=1 SV=3 | 33,02 | 43,83 | 30 | 44,04 |
| 389 | sp|Q9Y262|EIF3L_HUMAN | HUMAN | Eukaryotic translation initiation factor 3 subunit L OS=Homo sapiens GN=EIF3L PE=1 SV=1 | 32,85 | 33,05 | 19 | 35,82 |
| 390 | sp|P09651|ROA1_HUMAN | HUMAN | Heterogeneous nuclear ribonucleoprotein A1 OS=Homo sapiens GN=HNRNPA1 PE=1 SV=5 | 32,8 | 43,22 | 40 | 56,45 |
| 391 | sp|Q96HE7|ERO1A_HUMAN | HUMAN | ERO1-like protein alpha OS=Homo sapiens GN=ERO1A PE=1 SV=2 | 32,73 | 32,79 | 19 | 54,27 |
| 392 | sp|P04843|RPN1_HUMAN | HUMAN | Dolichyl-diphosphooligosaccharide--protein glycosyltransferase subunit 1 OS=Homo sapiens GN=RPN1 PE=1 SV=1 | 32,7 | 32,8 | 18 | 37,07 |
| 393 | sp|P29144|TPP2_HUMAN | HUMAN | Tripeptidyl-peptidase 2 OS=Homo sapiens GN=TPP2 PE=1 SV=4 | 32,67 | 33,61 | 18 | 18,01 |
| 394 | sp|Q9Y3I0|RTCB_HUMAN | HUMAN | tRNA-splicing ligase RtcB homolog OS=Homo sapiens GN=RTCB PE=1 SV=1 | 32,64 | 32,68 | 20 | 44,75 |
| 395 | sp|A0AVT1|UBA6_HUMAN | HUMAN | Ubiquitin-like modifier-activating enzyme 6 OS=Homo sapiens GN=UBA6 PE=1 SV=1 | 32,63 | 32,83 | 20 | 21,2 |
| 396 | sp|P00491|PNPH_HUMAN | HUMAN | Purine nucleoside phosphorylase OS=Homo sapiens GN=PNP PE=1 SV=2 | 32,62 | 32,63 | 23 | 73,36 |
| 397 | sp|Q53EL6|PDCD4_HUMAN | HUMAN | Programmed cell death protein 4 OS=Homo sapiens GN=PDCD4 PE=1 SV=2 | 32,5 | 32,53 | 16 | 45,63 |
| 398 | sp|P78347|GTF2I_HUMAN | HUMAN | General transcription factor II-I OS=Homo sapiens GN=GTF2I PE=1 SV=2 | 32,34 | 32,43 | 21 | 24,95 |
| 399 | sp|P10645|CMGA_HUMAN | HUMAN | Chromogranin-A OS=Homo sapiens GN=CHGA PE=1 SV=7 | 32,32 | 32,38 | 19 | 57,11 |
| 400 | sp|P11766|ADHX_HUMAN | HUMAN | Alcohol dehydrogenase class-3 OS=Homo sapiens GN=ADH5 PE=1 SV=4 | 32,3 | 32,39 | 22 | 55,35 |
| 401 | sp|P07339|CATD_HUMAN | HUMAN | Cathepsin D OS=Homo sapiens GN=CTSD PE=1 SV=1 | 32,23 | 32,26 | 33 | 50,97 |
| 402 | sp|P35222|CTNB1_HUMAN | HUMAN | Catenin beta-1 OS=Homo sapiens GN=CTNNB1 PE=1 SV=1 | 32,18 | 41,43 | 21 | 30,09 |
| 403 | sp|P07954|FUMH_HUMAN | HUMAN | Fumarate hydratase, mitochondrial OS=Homo sapiens GN=FH PE=1 SV=3 | 32,17 | 32,25 | 23 | 52,16 |
| 404 | sp|P18124|RL7_HUMAN | HUMAN | 60S ribosomal protein L7 OS=Homo sapiens GN=RPL7 PE=1 SV=1 | 32,16 | 32,21 | 26 | 53,63 |
| 405 | sp|P07585|PGS2_HUMAN | HUMAN | Decorin OS=Homo sapiens GN=DCN PE=1 SV=1 | 32,15 | 32,22 | 34 | 49,03 |
| 406 | sp|Q15582|BGH3_HUMAN | HUMAN | Transforming growth factor-beta-induced protein ig-h3 OS=Homo sapiens GN=TGFBI PE=1 SV=1 | 32,13 | 32,22 | 25 | 31,92 |
| 407 | sp|Q02218|ODO1_HUMAN | HUMAN | 2-oxoglutarate dehydrogenase, mitochondrial OS=Homo sapiens GN=OGDH PE=1 SV=3 | 32,11 | 32,77 | 19 | 31,09 |
| 408 | sp|P01871|IGHM_HUMAN | HUMAN | Ig mu chain C region OS=Homo sapiens GN=IGHM PE=1 SV=3 | 32,09 | 32,21 | 23 | 44,47 |
| 409 | sp|P49419|AL7A1_HUMAN | HUMAN | Alpha-aminoadipic semialdehyde dehydrogenase OS=Homo sapiens GN=ALDH7A1 PE=1 SV=5 | 32,08 | 32,2 | 21 | 52,13 |
| 410 | sp|Q9Y230|RUVB2_HUMAN | HUMAN | RuvB-like 2 OS=Homo sapiens GN=RUVBL2 PE=1 SV=3 | 32,03 | 32,1 | 18 | 40,39 |
| 411 | sp|Q01813|PFKAP_HUMAN | HUMAN | ATP-dependent 6-phosphofructokinase, platelet type OS=Homo sapiens GN=PFKP PE=1 SV=2 | 32 | 36,74 | 18 | 29,72 |
| 412 | sp|O00571|DDX3X_HUMAN | HUMAN | ATP-dependent RNA helicase DDX3X OS=Homo sapiens GN=DDX3X PE=1 SV=3 | 32 | 36,35 | 22 | 37,46 |
| 413 | sp|P49189|AL9A1_HUMAN | HUMAN | 4-trimethylaminobutyraldehyde dehydrogenase OS=Homo sapiens GN=ALDH9A1 PE=1 SV=3 | 31,84 | 32,19 | 18 | 44,13 |
| 414 | sp|Q9C0C2|TB182_HUMAN | HUMAN | 182 kDa tankyrase-1-binding protein OS=Homo sapiens GN=TNKS1BP1 PE=1 SV=4 | 31,83 | 32,05 | 19 | 15,21 |
| 415 | sp|P62424|RL7A_HUMAN | HUMAN | 60S ribosomal protein L7a OS=Homo sapiens GN=RPL7A PE=1 SV=2 | 31,8 | 31,86 | 24 | 46,99 |
| 416 | sp|P56470|LEG4_HUMAN | HUMAN | Galectin-4 OS=Homo sapiens GN=LGALS4 PE=1 SV=1 | 31,79 | 31,91 | 47 | 63,78 |
| 417 | sp|P49321|NASP_HUMAN | HUMAN | Nuclear autoantigenic sperm protein OS=Homo sapiens GN=NASP PE=1 SV=2 | 31,73 | 31,88 | 17 | 25,89 |
| 418 | sp|Q9H4M9|EHD1_HUMAN | HUMAN | EH domain-containing protein 1 OS=Homo sapiens GN=EHD1 PE=1 SV=2 | 31,72 | 36,25 | 23 | 48,31 |
| 419 | sp|P61247|RS3A_HUMAN | HUMAN | 40S ribosomal protein S3a OS=Homo sapiens GN=RPS3A PE=1 SV=2 | 31,7 | 31,79 | 21 | 53,79 |
| 420 | sp|P30041|PRDX6_HUMAN | HUMAN | Peroxiredoxin-6 OS=Homo sapiens GN=PRDX6 PE=1 SV=3 | 31,68 | 31,7 | 28 | 76,34 |
| 421 | sp|Q15459|SF3A1_HUMAN | HUMAN | Splicing factor 3A subunit 1 OS=Homo sapiens GN=SF3A1 PE=1 SV=1 | 31,52 | 31,68 | 19 | 29,13 |
| 422 | sp|P13798|ACPH_HUMAN | HUMAN | Acylamino-acid-releasing enzyme OS=Homo sapiens GN=APEH PE=1 SV=4 | 31,38 | 31,47 | 19 | 35,52 |
| 423 | sp|P67809|YBOX1_HUMAN | HUMAN | Nuclease-sensitive element-binding protein 1 OS=Homo sapiens GN=YBX1 PE=1 SV=3 | 31,38 | 31,41 | 26 | 69,75 |
| 424 | sp|P07686|HEXB_HUMAN | HUMAN | Beta-hexosaminidase subunit beta OS=Homo sapiens GN=HEXB PE=1 SV=3 | 31,36 | 31,53 | 17 | 34,71 |
| 425 | sp|Q14258|TRI25_HUMAN | HUMAN | E3 ubiquitin/ISG15 ligase TRIM25 OS=Homo sapiens GN=TRIM25 PE=1 SV=2 | 31,29 | 33,66 | 16 | 29,52 |
| 426 | sp|P01031|CO5_HUMAN | HUMAN | Complement C5 OS=Homo sapiens GN=C5 PE=1 SV=4 | 31,29 | 31,6 | 15 | 12,71 |
| 427 | sp|P17980|PRS6A_HUMAN | HUMAN | 26S protease regulatory subunit 6A OS=Homo sapiens GN=PSMC3 PE=1 SV=3 | 31,28 | 31,45 | 22 | 47,38 |
| 428 | sp|P04792|HSPB1_HUMAN | HUMAN | Heat shock protein beta-1 OS=Homo sapiens GN=HSPB1 PE=1 SV=2 | 31,26 | 31,29 | 41 | 78,05 |
| 429 | sp|P18669|PGAM1_HUMAN | HUMAN | Phosphoglycerate mutase 1 OS=Homo sapiens GN=PGAM1 PE=1 SV=2 | 31,21 | 31,24 | 40 | 70,08 |
| 430 | sp|Q06830|PRDX1_HUMAN | HUMAN | Peroxiredoxin-1 OS=Homo sapiens GN=PRDX1 PE=1 SV=1 | 31,09 | 31,14 | 38 | 79,9 |
| 431 | sp|P54868|HMCS2_HUMAN | HUMAN | Hydroxymethylglutaryl-CoA synthase, mitochondrial OS=Homo sapiens GN=HMGCS2 PE=1 SV=1 | 31,02 | 31,14 | 25 | 43,31 |
| 432 | sp|Q15008|PSMD6_HUMAN | HUMAN | 26S proteasome non-ATPase regulatory subunit 6 OS=Homo sapiens GN=PSMD6 PE=1 SV=1 | 30,78 | 30,9 | 16 | 36,5 |
| 433 | sp|P61221|ABCE1_HUMAN | HUMAN | ATP-binding cassette sub-family E member 1 OS=Homo sapiens GN=ABCE1 PE=1 SV=1 | 30,71 | 30,86 | 17 | 37,23 |
| 434 | sp|P40925|MDHC_HUMAN | HUMAN | Malate dehydrogenase, cytoplasmic OS=Homo sapiens GN=MDH1 PE=1 SV=4 | 30,6 | 30,75 | 27 | 51,5 |
| 435 | sp|Q3LXA3|TKFC_HUMAN | HUMAN | Triokinase/FMN cyclase OS=Homo sapiens GN=TKFC PE=1 SV=2 | 30,54 | 30,65 | 15 | 42,61 |
| 436 | sp|P00390|GSHR_HUMAN | HUMAN | Glutathione reductase, mitochondrial OS=Homo sapiens GN=GSR PE=1 SV=2 | 30,52 | 30,61 | 28 | 46,93 |
| 437 | sp|Q15075|EEA1_HUMAN | HUMAN | Early endosome antigen 1 OS=Homo sapiens GN=EEA1 PE=1 SV=2 | 30,51 | 30,81 | 15 | 14,39 |
| 438 | sp|Q9HC38|GLOD4_HUMAN | HUMAN | Glyoxalase domain-containing protein 4 OS=Homo sapiens GN=GLOD4 PE=1 SV=1 | 30,44 | 30,5 | 18 | 61,02 |
| 439 | sp|O60610|DIAP1_HUMAN | HUMAN | Protein diaphanous homolog 1 OS=Homo sapiens GN=DIAPH1 PE=1 SV=2 | 30,26 | 31 | 17 | 15,64 |
| 440 | sp|Q9UL46|PSME2_HUMAN | HUMAN | Proteasome activator complex subunit 2 OS=Homo sapiens GN=PSME2 PE=1 SV=4 | 30,23 | 30,4 | 35 | 74,48 |
| 441 | sp|P60981|DEST_HUMAN | HUMAN | Destrin OS=Homo sapiens GN=DSTN PE=1 SV=3 | 30,2 | 32,3 | 28 | 81,21 |
| 442 | sp|P51884|LUM_HUMAN | HUMAN | Lumican OS=Homo sapiens GN=LUM PE=1 SV=2 | 30,11 | 30,82 | 29 | 50,89 |
| 443 | sp|O15143|ARC1B_HUMAN | HUMAN | Actin-related protein 2/3 complex subunit 1B OS=Homo sapiens GN=ARPC1B PE=1 SV=3 | 30,08 | 30,18 | 21 | 49,46 |
| 444 | sp|P15880|RS2_HUMAN | HUMAN | 40S ribosomal protein S2 OS=Homo sapiens GN=RPS2 PE=1 SV=2 | 29,93 | 30,24 | 25 | 56,66 |
| 445 | sp|Q9Y5K6|CD2AP_HUMAN | HUMAN | CD2-associated protein OS=Homo sapiens GN=CD2AP PE=1 SV=1 | 29,92 | 30,08 | 16 | 34,27 |
| 446 | sp|P21281|VATB2_HUMAN | HUMAN | V-type proton ATPase subunit B, brain isoform OS=Homo sapiens GN=ATP6V1B2 PE=1 SV=3 | 29,91 | 30 | 19 | 48,14 |
| 447 | sp|Q14980|NUMA1_HUMAN | HUMAN | Nuclear mitotic apparatus protein 1 OS=Homo sapiens GN=NUMA1 PE=1 SV=2 | 29,9 | 30,78 | 15 | 9,645 |
| 448 | sp|Q9NQW7|XPP1_HUMAN | HUMAN | Xaa-Pro aminopeptidase 1 OS=Homo sapiens GN=XPNPEP1 PE=1 SV=3 | 29,89 | 29,98 | 20 | 41,09 |
| 449 | sp|Q14166|TTL12_HUMAN | HUMAN | Tubulin--tyrosine ligase-like protein 12 OS=Homo sapiens GN=TTLL12 PE=1 SV=2 | 29,87 | 30,01 | 22 | 38,82 |
| 450 | sp|Q16762|THTR_HUMAN | HUMAN | Thiosulfate sulfurtransferase OS=Homo sapiens GN=TST PE=1 SV=4 | 29,81 | 29,88 | 17 | 57,58 |
| 451 | sp|P54727|RD23B_HUMAN | HUMAN | UV excision repair protein RAD23 homolog B OS=Homo sapiens GN=RAD23B PE=1 SV=1 | 29,74 | 29,79 | 17 | 51,83 |
| 452 | sp|P27361|MK03_HUMAN | HUMAN | Mitogen-activated protein kinase 3 OS=Homo sapiens GN=MAPK3 PE=1 SV=4 | 29,7 | 29,79 | 21 | 50,92 |
| 453 | sp|Q9BR76|COR1B_HUMAN | HUMAN | Coronin-1B OS=Homo sapiens GN=CORO1B PE=1 SV=1 | 29,68 | 30,27 | 18 | 41,72 |
| 454 | sp|O14745|NHRF1_HUMAN | HUMAN | Na(+)/H(+) exchange regulatory cofactor NHE-RF1 OS=Homo sapiens GN=SLC9A3R1 PE=1 SV=4 | 29,63 | 29,83 | 18 | 50,84 |
| 455 | sp|Q9UKM9|RALY_HUMAN | HUMAN | RNA-binding protein Raly OS=Homo sapiens GN=RALY PE=1 SV=1 | 29,62 | 29,81 | 22 | 69,61 |
| 456 | sp|P32455|GBP1_HUMAN | HUMAN | Guanylate-binding protein 1 OS=Homo sapiens GN=GBP1 PE=1 SV=2 | 29,62 | 29,71 | 25 | 30,07 |
| 457 | sp|P12268|IMDH2_HUMAN | HUMAN | Inosine-5'-monophosphate dehydrogenase 2 OS=Homo sapiens GN=IMPDH2 PE=1 SV=2 | 29,59 | 29,63 | 19 | 38,13 |
| 458 | sp|Q15691|MARE1_HUMAN | HUMAN | Microtubule-associated protein RP/EB family member 1 OS=Homo sapiens GN=MAPRE1 PE=1 SV=3 | 29,57 | 29,6 | 25 | 75,37 |
| 459 | sp|O00515|LAD1_HUMAN | HUMAN | Ladinin-1 OS=Homo sapiens GN=LAD1 PE=1 SV=2 | 29,52 | 30,04 | 18 | 39,26 |
| 460 | sp|P60228|EIF3E_HUMAN | HUMAN | Eukaryotic translation initiation factor 3 subunit E OS=Homo sapiens GN=EIF3E PE=1 SV=1 | 29,44 | 29,54 | 17 | 42,7 |
| 461 | sp|P09525|ANXA4_HUMAN | HUMAN | Annexin A4 OS=Homo sapiens GN=ANXA4 PE=1 SV=4 | 29,35 | 32,81 | 20 | 59,25 |
| 462 | sp|P30837|AL1B1_HUMAN | HUMAN | Aldehyde dehydrogenase X, mitochondrial OS=Homo sapiens GN=ALDH1B1 PE=1 SV=3 | 29,27 | 32,22 | 23 | 49,9 |
| 463 | sp|Q9NUQ9|FA49B_HUMAN | HUMAN | Protein FAM49B OS=Homo sapiens GN=FAM49B PE=1 SV=1 | 29,25 | 29,3 | 18 | 58,02 |
| 464 | sp|Q99714|HCD2_HUMAN | HUMAN | 3-hydroxyacyl-CoA dehydrogenase type-2 OS=Homo sapiens GN=HSD17B10 PE=1 SV=3 | 29,01 | 29,03 | 19 | 78,93 |
| 465 | sp|P50552|VASP_HUMAN | HUMAN | Vasodilator-stimulated phosphoprotein OS=Homo sapiens GN=VASP PE=1 SV=3 | 28,99 | 29,08 | 21 | 46,84 |
| 466 | sp|Q9Y266|NUDC_HUMAN | HUMAN | Nuclear migration protein nudC OS=Homo sapiens GN=NUDC PE=1 SV=1 | 28,99 | 29,06 | 18 | 52,87 |
| 467 | sp|P02452|CO1A1_HUMAN | HUMAN | Collagen alpha-1(I) chain OS=Homo sapiens GN=COL1A1 PE=1 SV=5 | 28,98 | 29,04 | 64 | 35,52 |
| 468 | sp|P23141|EST1_HUMAN | HUMAN | Liver carboxylesterase 1 OS=Homo sapiens GN=CES1 PE=1 SV=2 | 28,92 | 28,99 | 16 | 36,51 |
| 469 | sp|P43686|PRS6B_HUMAN | HUMAN | 26S protease regulatory subunit 6B OS=Homo sapiens GN=PSMC4 PE=1 SV=2 | 28,9 | 29,55 | 17 | 42,58 |
| 470 | sp|P06737|PYGL_HUMAN | HUMAN | Glycogen phosphorylase, liver form OS=Homo sapiens GN=PYGL PE=1 SV=4 | 28,87 | 40,79 | 21 | 30,22 |
| 471 | sp|P46060|RAGP1_HUMAN | HUMAN | Ran GTPase-activating protein 1 OS=Homo sapiens GN=RANGAP1 PE=1 SV=1 | 28,87 | 28,95 | 15 | 37,82 |
| 472 | sp|O00299|CLIC1_HUMAN | HUMAN | Chloride intracellular channel protein 1 OS=Homo sapiens GN=CLIC1 PE=1 SV=4 | 28,86 | 31,83 | 39 | 79,67 |
| 473 | sp|Q9NR45|SIAS_HUMAN | HUMAN | Sialic acid synthase OS=Homo sapiens GN=NANS PE=1 SV=2 | 28,84 | 29,02 | 30 | 61,28 |
| 474 | sp|Q99873|ANM1_HUMAN | HUMAN | Protein arginine N-methyltransferase 1 OS=Homo sapiens GN=PRMT1 PE=1 SV=2 | 28,78 | 28,84 | 15 | 36,84 |
| 475 | sp|Q9Y3F4|STRAP_HUMAN | HUMAN | Serine-threonine kinase receptor-associated protein OS=Homo sapiens GN=STRAP PE=1 SV=1 | 28,7 | 28,8 | 16 | 59,43 |
| 476 | sp|P09211|GSTP1_HUMAN | HUMAN | Glutathione S-transferase P OS=Homo sapiens GN=GSTP1 PE=1 SV=2 | 28,69 | 28,7 | 63 | 70 |
| 477 | sp|Q96HC4|PDLI5_HUMAN | HUMAN | PDZ and LIM domain protein 5 OS=Homo sapiens GN=PDLIM5 PE=1 SV=5 | 28,65 | 28,74 | 15 | 32,05 |
| 478 | sp|Q9UNZ2|NSF1C_HUMAN | HUMAN | NSFL1 cofactor p47 OS=Homo sapiens GN=NSFL1C PE=1 SV=2 | 28,63 | 28,72 | 15 | 49,19 |
| 479 | sp|Q6XQN6|PNCB_HUMAN | HUMAN | Nicotinate phosphoribosyltransferase OS=Homo sapiens GN=NAPRT PE=1 SV=2 | 28,58 | 28,67 | 24 | 53,35 |
| 480 | sp|Q08380|LG3BP_HUMAN | HUMAN | Galectin-3-binding protein OS=Homo sapiens GN=LGALS3BP PE=1 SV=1 | 28,53 | 28,63 | 24 | 36,07 |
| 481 | sp|P10768|ESTD_HUMAN | HUMAN | S-formylglutathione hydrolase OS=Homo sapiens GN=ESD PE=1 SV=2 | 28,51 | 28,56 | 19 | 73,05 |
| 482 | sp|P13611|CSPG2_HUMAN | HUMAN | Versican core protein OS=Homo sapiens GN=VCAN PE=1 SV=3 | 28,48 | 28,98 | 17 | 5,33 |
| 483 | sp|P02749|APOH_HUMAN | HUMAN | Beta-2-glycoprotein 1 OS=Homo sapiens GN=APOH PE=1 SV=3 | 28,46 | 28,5 | 18 | 57,1 |
| 484 | sp|O43390|HNRPR_HUMAN | HUMAN | Heterogeneous nuclear ribonucleoprotein R OS=Homo sapiens GN=HNRNPR PE=1 SV=1 | 28,29 | 44,96 | 30 | 39,18 |
| 485 | sp|Q14103|HNRPD_HUMAN | HUMAN | Heterogeneous nuclear ribonucleoprotein D0 OS=Homo sapiens GN=HNRNPD PE=1 SV=1 | 28,29 | 28,36 | 24 | 41,97 |
| 486 | sp|P62495|ERF1_HUMAN | HUMAN | Eukaryotic peptide chain release factor subunit 1 OS=Homo sapiens GN=ETF1 PE=1 SV=3 | 28,28 | 28,35 | 16 | 37,3 |
| 487 | sp|P78417|GSTO1_HUMAN | HUMAN | Glutathione S-transferase omega-1 OS=Homo sapiens GN=GSTO1 PE=1 SV=2 | 28,21 | 28,27 | 16 | 63,9 |
| 488 | sp|P23588|IF4B_HUMAN | HUMAN | Eukaryotic translation initiation factor 4B OS=Homo sapiens GN=EIF4B PE=1 SV=2 | 28,19 | 28,27 | 20 | 34,04 |
| 489 | sp|P32119|PRDX2_HUMAN | HUMAN | Peroxiredoxin-2 OS=Homo sapiens GN=PRDX2 PE=1 SV=5 | 28,14 | 30,26 | 38 | 73,74 |
| 490 | sp|Q9P258|RCC2_HUMAN | HUMAN | Protein RCC2 OS=Homo sapiens GN=RCC2 PE=1 SV=2 | 28,12 | 28,17 | 15 | 33,14 |
| 491 | sp|P21291|CSRP1_HUMAN | HUMAN | Cysteine and glycine-rich protein 1 OS=Homo sapiens GN=CSRP1 PE=1 SV=3 | 28,1 | 28,14 | 43 | 65,28 |
| 492 | sp|P23284|PPIB_HUMAN | HUMAN | Peptidyl-prolyl cis-trans isomerase B OS=Homo sapiens GN=PPIB PE=1 SV=2 | 27,93 | 28 | 23 | 56,94 |
| 493 | sp|O14818|PSA7_HUMAN | HUMAN | Proteasome subunit alpha type-7 OS=Homo sapiens GN=PSMA7 PE=1 SV=1 | 27,92 | 28,02 | 17 | 55,65 |
| 494 | sp|O60716|CTND1_HUMAN | HUMAN | Catenin delta-1 OS=Homo sapiens GN=CTNND1 PE=1 SV=1 | 27,87 | 28,23 | 16 | 21,8 |
| 495 | sp|O00534|VMA5A_HUMAN | HUMAN | von Willebrand factor A domain-containing protein 5A OS=Homo sapiens GN=VWA5A PE=2 SV=2 | 27,86 | 27,97 | 15 | 26,21 |
| 496 | sp|Q9UHB9|SRP68_HUMAN | HUMAN | Signal recognition particle subunit SRP68 OS=Homo sapiens GN=SRP68 PE=1 SV=2 | 27,81 | 27,95 | 15 | 29,51 |
| 497 | sp|P05388|RLA0_HUMAN | HUMAN | 60S acidic ribosomal protein P0 OS=Homo sapiens GN=RPLP0 PE=1 SV=1 | 27,8 | 27,88 | 29 | 70,98 |
| 498 | sp|Q0VD83|APOBR_HUMAN | HUMAN | Apolipoprotein B receptor OS=Homo sapiens GN=APOBR PE=1 SV=2 | 27,79 | 27,87 | 15 | 18,2 |
| 499 | sp|Q99497|PARK7_HUMAN | HUMAN | Protein deglycase DJ-1 OS=Homo sapiens GN=PARK7 PE=1 SV=2 | 27,76 | 27,84 | 27 | 73,54 |
| 500 | sp|P00915|CAH1_HUMAN | HUMAN | Carbonic anhydrase 1 OS=Homo sapiens GN=CA1 PE=1 SV=2 | 27,76 | 27,81 | 48 | 72,03 |
| 501 | sp|P04217|A1BG_HUMAN | HUMAN | Alpha-1B-glycoprotein OS=Homo sapiens GN=A1BG PE=1 SV=4 | 27,73 | 28,15 | 18 | 55,15 |
| 502 | sp|Q9UNM6|PSD13_HUMAN | HUMAN | 26S proteasome non-ATPase regulatory subunit 13 OS=Homo sapiens GN=PSMD13 PE=1 SV=2 | 27,62 | 28,04 | 20 | 43,09 |
| 503 | sp|P52597|HNRPF_HUMAN | HUMAN | Heterogeneous nuclear ribonucleoprotein F OS=Homo sapiens GN=HNRNPF PE=1 SV=3 | 27,61 | 33,44 | 26 | 60,72 |
| 504 | sp|Q9Y265|RUVB1_HUMAN | HUMAN | RuvB-like 1 OS=Homo sapiens GN=RUVBL1 PE=1 SV=1 | 27,59 | 27,81 | 16 | 44,74 |
| 505 | sp|P25788|PSA3_HUMAN | HUMAN | Proteasome subunit alpha type-3 OS=Homo sapiens GN=PSMA3 PE=1 SV=2 | 27,59 | 27,69 | 16 | 47,84 |
| 506 | sp|P12429|ANXA3_HUMAN | HUMAN | Annexin A3 OS=Homo sapiens GN=ANXA3 PE=1 SV=3 | 27,56 | 29,17 | 23 | 56,97 |
| 507 | sp|P61586|RHOA_HUMAN | HUMAN | Transforming protein RhoA OS=Homo sapiens GN=RHOA PE=1 SV=1 | 27,56 | 27,61 | 26 | 75,13 |
| 508 | sp|O94760|DDAH1_HUMAN | HUMAN | N(G),N(G)-dimethylarginine dimethylaminohydrolase 1 OS=Homo sapiens GN=DDAH1 PE=1 SV=3 | 27,53 | 27,84 | 22 | 66,67 |
| 509 | sp|P04899|GNAI2_HUMAN | HUMAN | Guanine nucleotide-binding protein G(i) subunit alpha-2 OS=Homo sapiens GN=GNAI2 PE=1 SV=3 | 27,51 | 27,66 | 15 | 54,65 |
| 510 | sp|P62820|RAB1A_HUMAN | HUMAN | Ras-related protein Rab-1A OS=Homo sapiens GN=RAB1A PE=1 SV=3 | 27,51 | 27,64 | 24 | 86,83 |
| 511 | sp|Q8TAX9|GSDMB_HUMAN | HUMAN | Gasdermin-B OS=Homo sapiens GN=GSDMB PE=2 SV=2 | 27,47 | 27,69 | 18 | 45,74 |
| 512 | sp|P42166|LAP2A_HUMAN | HUMAN | Lamina-associated polypeptide 2, isoform alpha OS=Homo sapiens GN=TMPO PE=1 SV=2 | 27,42 | 27,58 | 21 | 26,08 |
| 513 | sp|Q63HN8|RN213_HUMAN | HUMAN | E3 ubiquitin-protein ligase RNF213 OS=Homo sapiens GN=RNF213 PE=1 SV=3 | 27,36 | 28,95 | 15 | 3,726 |
| 514 | sp|P13804|ETFA_HUMAN | HUMAN | Electron transfer flavoprotein subunit alpha, mitochondrial OS=Homo sapiens GN=ETFA PE=1 SV=1 | 27,36 | 27,49 | 17 | 70,57 |
| 515 | sp|P04424|ARLY_HUMAN | HUMAN | Argininosuccinate lyase OS=Homo sapiens GN=ASL PE=1 SV=4 | 27,31 | 27,43 | 18 | 37,28 |
| 516 | sp|Q16881|TRXR1_HUMAN | HUMAN | Thioredoxin reductase 1, cytoplasmic OS=Homo sapiens GN=TXNRD1 PE=1 SV=3 | 27,3 | 27,55 | 20 | 35,59 |
| 517 | sp|O00748|EST2_HUMAN | HUMAN | Cocaine esterase OS=Homo sapiens GN=CES2 PE=1 SV=1 | 27,26 | 27,3 | 14 | 34,88 |
| 518 | sp|P60900|PSA6_HUMAN | HUMAN | Proteasome subunit alpha type-6 OS=Homo sapiens GN=PSMA6 PE=1 SV=1 | 27,25 | 27,35 | 20 | 60,57 |
| 519 | sp|Q92900|RENT1_HUMAN | HUMAN | Regulator of nonsense transcripts 1 OS=Homo sapiens GN=UPF1 PE=1 SV=2 | 27,24 | 27,48 | 15 | 17,45 |
| 520 | sp|Q9UDY2|ZO2_HUMAN | HUMAN | Tight junction protein ZO-2 OS=Homo sapiens GN=TJP2 PE=1 SV=2 | 27,22 | 27,51 | 16 | 16,05 |
| 521 | sp|Q15293|RCN1_HUMAN | HUMAN | Reticulocalbin-1 OS=Homo sapiens GN=RCN1 PE=1 SV=1 | 27,16 | 27,25 | 17 | 57,4 |
| 522 | sp|Q12905|ILF2_HUMAN | HUMAN | Interleukin enhancer-binding factor 2 OS=Homo sapiens GN=ILF2 PE=1 SV=2 | 27,16 | 27,19 | 24 | 53,33 |
| 523 | sp|P17844|DDX5_HUMAN | HUMAN | Probable ATP-dependent RNA helicase DDX5 OS=Homo sapiens GN=DDX5 PE=1 SV=1 | 27,12 | 43,74 | 33 | 42,51 |
| 524 | sp|Q9UMS4|PRP19_HUMAN | HUMAN | Pre-mRNA-processing factor 19 OS=Homo sapiens GN=PRPF19 PE=1 SV=1 | 27,12 | 28,67 | 17 | 50 |
| 525 | sp|Q15019|SEPT2_HUMAN | HUMAN | Septin-2 OS=Homo sapiens GN=SEPT2 PE=1 SV=1 | 27,1 | 29,37 | 18 | 59,56 |
| 526 | sp|Q9Y6N5|SQRD_HUMAN | HUMAN | Sulfide:quinone oxidoreductase, mitochondrial OS=Homo sapiens GN=SQRDL PE=1 SV=1 | 27,09 | 27,19 | 15 | 40 |
| 527 | sp|P27708|PYR1_HUMAN | HUMAN | CAD protein OS=Homo sapiens GN=CAD PE=1 SV=3 | 27 | 27,7 | 15 | 8,27 |
| 528 | sp|P62249|RS16_HUMAN | HUMAN | 40S ribosomal protein S16 OS=Homo sapiens GN=RPS16 PE=1 SV=2 | 26,97 | 27 | 22 | 67,81 |
| 529 | sp|P09622|DLDH_HUMAN | HUMAN | Dihydrolipoyl dehydrogenase, mitochondrial OS=Homo sapiens GN=DLD PE=1 SV=2 | 26,93 | 27,08 | 15 | 37,52 |
| 530 | sp|Q13283|G3BP1_HUMAN | HUMAN | Ras GTPase-activating protein-binding protein 1 OS=Homo sapiens GN=G3BP1 PE=1 SV=1 | 26,93 | 26,96 | 22 | 45,28 |
| 531 | sp|Q15233|NONO_HUMAN | HUMAN | Non-POU domain-containing octamer-binding protein OS=Homo sapiens GN=NONO PE=1 SV=4 | 26,92 | 29,44 | 19 | 34,61 |
| 532 | sp|Q8TEX9|IPO4_HUMAN | HUMAN | Importin-4 OS=Homo sapiens GN=IPO4 PE=1 SV=2 | 26,91 | 27,23 | 18 | 21,65 |
| 533 | sp|P39687|AN32A_HUMAN | HUMAN | Acidic leucine-rich nuclear phosphoprotein 32 family member A OS=Homo sapiens GN=ANP32A PE=1 SV=1 | 26,87 | 26,97 | 25 | 40,96 |
| 534 | sp|P08865|RSSA_HUMAN | HUMAN | 40S ribosomal protein SA OS=Homo sapiens GN=RPSA PE=1 SV=4 | 26,86 | 26,89 | 24 | 57,63 |
| 535 | sp|P47756|CAPZB_HUMAN | HUMAN | F-actin-capping protein subunit beta OS=Homo sapiens GN=CAPZB PE=1 SV=4 | 26,83 | 26,91 | 19 | 49,46 |
| 536 | sp|Q08J23|NSUN2_HUMAN | HUMAN | tRNA (cytosine(34)-C(5))-methyltransferase OS=Homo sapiens GN=NSUN2 PE=1 SV=2 | 26,79 | 27,04 | 17 | 25,55 |
| 537 | sp|Q16401|PSMD5_HUMAN | HUMAN | 26S proteasome non-ATPase regulatory subunit 5 OS=Homo sapiens GN=PSMD5 PE=1 SV=3 | 26,73 | 26,87 | 15 | 35,32 |
| 538 | sp|O75363|BCAS1_HUMAN | HUMAN | Breast carcinoma-amplified sequence 1 OS=Homo sapiens GN=BCAS1 PE=1 SV=2 | 26,67 | 26,72 | 15 | 32,71 |
| 539 | sp|Q7L1Q6|BZW1_HUMAN | HUMAN | Basic leucine zipper and W2 domain-containing protein 1 OS=Homo sapiens GN=BZW1 PE=1 SV=1 | 26,63 | 28,13 | 14 | 29,59 |
| 540 | sp|P61160|ARP2_HUMAN | HUMAN | Actin-related protein 2 OS=Homo sapiens GN=ACTR2 PE=1 SV=1 | 26,55 | 26,8 | 22 | 47,46 |
| 541 | sp|P51991|ROA3_HUMAN | HUMAN | Heterogeneous nuclear ribonucleoprotein A3 OS=Homo sapiens GN=HNRNPA3 PE=1 SV=2 | 26,52 | 35,06 | 22 | 38,62 |
| 542 | sp|Q9NR12|PDLI7_HUMAN | HUMAN | PDZ and LIM domain protein 7 OS=Homo sapiens GN=PDLIM7 PE=1 SV=1 | 26,5 | 26,69 | 14 | 44,64 |
| 543 | sp|P52907|CAZA1_HUMAN | HUMAN | F-actin-capping protein subunit alpha-1 OS=Homo sapiens GN=CAPZA1 PE=1 SV=3 | 26,49 | 26,74 | 25 | 71,33 |
| 544 | sp|P38117|ETFB_HUMAN | HUMAN | Electron transfer flavoprotein subunit beta OS=Homo sapiens GN=ETFB PE=1 SV=3 | 26,43 | 26,52 | 15 | 56,08 |
| 545 | sp|P62269|RS18_HUMAN | HUMAN | 40S ribosomal protein S18 OS=Homo sapiens GN=RPS18 PE=1 SV=3 | 26,41 | 26,5 | 24 | 62,5 |
| 546 | sp|O43399|TPD54_HUMAN | HUMAN | Tumor protein D54 OS=Homo sapiens GN=TPD52L2 PE=1 SV=2 | 26,23 | 26,29 | 20 | 73,79 |
| 547 | sp|P54819|KAD2_HUMAN | HUMAN | Adenylate kinase 2, mitochondrial OS=Homo sapiens GN=AK2 PE=1 SV=2 | 26,11 | 26,18 | 20 | 63,6 |
| 548 | sp|P31947|1433S_HUMAN | HUMAN | 14-3-3 protein sigma OS=Homo sapiens GN=SFN PE=1 SV=1 | 26,09 | 32,29 | 36 | 80,65 |
| 549 | sp|P46781|RS9_HUMAN | HUMAN | 40S ribosomal protein S9 OS=Homo sapiens GN=RPS9 PE=1 SV=3 | 26,04 | 26,22 | 22 | 52,58 |
| 550 | sp|Q9Y2Z0|SGT1_HUMAN | HUMAN | Protein SGT1 homolog OS=Homo sapiens GN=SUGT1 PE=1 SV=3 | 25,99 | 26,23 | 14 | 44,66 |
| 551 | sp|P04003|C4BPA_HUMAN | HUMAN | C4b-binding protein alpha chain OS=Homo sapiens GN=C4BPA PE=1 SV=2 | 25,94 | 27,31 | 15 | 28,48 |
| 552 | sp|Q9NZB2|F120A_HUMAN | HUMAN | Constitutive coactivator of PPAR-gamma-like protein 1 OS=Homo sapiens GN=FAM120A PE=1 SV=2 | 25,91 | 26,29 | 13 | 17,8 |
| 553 | sp|P62158|CALM_HUMAN | HUMAN | Calmodulin OS=Homo sapiens GN=CALM1 PE=1 SV=2 | 25,88 | 25,93 | 40 | 98,66 |
| 554 | sp|Q02878|RL6_HUMAN | HUMAN | 60S ribosomal protein L6 OS=Homo sapiens GN=RPL6 PE=1 SV=3 | 25,82 | 25,87 | 16 | 42,36 |
| 555 | sp|P07737|PROF1_HUMAN | HUMAN | Profilin-1 OS=Homo sapiens GN=PFN1 PE=1 SV=2 | 25,78 | 25,82 | 46 | 89,29 |
| 556 | sp|Q15404|RSU1_HUMAN | HUMAN | Ras suppressor protein 1 OS=Homo sapiens GN=RSU1 PE=1 SV=3 | 25,75 | 25,89 | 17 | 59,57 |
| 557 | sp|Q15435|PP1R7_HUMAN | HUMAN | Protein phosphatase 1 regulatory subunit 7 OS=Homo sapiens GN=PPP1R7 PE=1 SV=1 | 25,73 | 27,56 | 21 | 59,17 |
| 558 | sp|P63241|IF5A1_HUMAN | HUMAN | Eukaryotic translation initiation factor 5A-1 OS=Homo sapiens GN=EIF5A PE=1 SV=2 | 25,66 | 25,73 | 38 | 90,91 |
| 559 | sp|O14974|MYPT1_HUMAN | HUMAN | Protein phosphatase 1 regulatory subunit 12A OS=Homo sapiens GN=PPP1R12A PE=1 SV=1 | 25,65 | 26,59 | 15 | 16,89 |
| 560 | sp|Q92973|TNPO1_HUMAN | HUMAN | Transportin-1 OS=Homo sapiens GN=TNPO1 PE=1 SV=2 | 25,64 | 25,76 | 17 | 18,93 |
| 561 | sp|Q9UD71|PPR1B_HUMAN | HUMAN | Protein phosphatase 1 regulatory subunit 1B OS=Homo sapiens GN=PPP1R1B PE=1 SV=2 | 25,62 | 25,92 | 21 | 81,37 |
| 562 | sp|P15121|ALDR_HUMAN | HUMAN | Aldose reductase OS=Homo sapiens GN=AKR1B1 PE=1 SV=3 | 25,59 | 27,85 | 14 | 59,18 |
| 563 | sp|Q96CX2|KCD12_HUMAN | HUMAN | BTB/POZ domain-containing protein KCTD12 OS=Homo sapiens GN=KCTD12 PE=1 SV=1 | 25,56 | 25,67 | 17 | 52,92 |
| 564 | sp|O00203|AP3B1_HUMAN | HUMAN | AP-3 complex subunit beta-1 OS=Homo sapiens GN=AP3B1 PE=1 SV=3 | 25,5 | 26,01 | 14 | 15,45 |
| 565 | sp|Q9UKK3|PARP4_HUMAN | HUMAN | Poly [ADP-ribose] polymerase 4 OS=Homo sapiens GN=PARP4 PE=1 SV=3 | 25,49 | 25,76 | 16 | 9,571 |
| 566 | sp|P20042|IF2B_HUMAN | HUMAN | Eukaryotic translation initiation factor 2 subunit 2 OS=Homo sapiens GN=EIF2S2 PE=1 SV=2 | 25,47 | 25,62 | 18 | 53,15 |
| 567 | sp|P50453|SPB9_HUMAN | HUMAN | Serpin B9 OS=Homo sapiens GN=SERPINB9 PE=1 SV=1 | 25,44 | 28,45 | 18 | 42,82 |
| 568 | sp|Q9UHX1|PUF60_HUMAN | HUMAN | Poly(U)-binding-splicing factor PUF60 OS=Homo sapiens GN=PUF60 PE=1 SV=1 | 25,4 | 25,5 | 18 | 30,77 |
| 569 | sp|P62191|PRS4_HUMAN | HUMAN | 26S protease regulatory subunit 4 OS=Homo sapiens GN=PSMC1 PE=1 SV=1 | 25,38 | 28,47 | 16 | 45,68 |
| 570 | sp|P36955|PEDF_HUMAN | HUMAN | Pigment epithelium-derived factor OS=Homo sapiens GN=SERPINF1 PE=1 SV=4 | 25,29 | 25,88 | 14 | 40,19 |
| 571 | sp|P55084|ECHB_HUMAN | HUMAN | Trifunctional enzyme subunit beta, mitochondrial OS=Homo sapiens GN=HADHB PE=1 SV=3 | 25,21 | 25,33 | 15 | 34,39 |
| 572 | sp|Q6NZI2|PTRF_HUMAN | HUMAN | Polymerase I and transcript release factor OS=Homo sapiens GN=PTRF PE=1 SV=1 | 25,12 | 25,84 | 21 | 43,85 |
| 573 | sp|P21266|GSTM3_HUMAN | HUMAN | Glutathione S-transferase Mu 3 OS=Homo sapiens GN=GSTM3 PE=1 SV=3 | 25,1 | 25,26 | 17 | 65,78 |
| 574 | sp|P46782|RS5_HUMAN | HUMAN | 40S ribosomal protein S5 OS=Homo sapiens GN=RPS5 PE=1 SV=4 | 25,05 | 25,37 | 24 | 55,88 |
| 575 | sp|P19827|ITIH1_HUMAN | HUMAN | Inter-alpha-trypsin inhibitor heavy chain H1 OS=Homo sapiens GN=ITIH1 PE=1 SV=3 | 25,04 | 25,15 | 15 | 22,83 |
| 576 | sp|Q9UIA9|XPO7_HUMAN | HUMAN | Exportin-7 OS=Homo sapiens GN=XPO7 PE=1 SV=3 | 25,01 | 25,25 | 15 | 17,02 |
| 577 | sp|P40121|CAPG_HUMAN | HUMAN | Macrophage-capping protein OS=Homo sapiens GN=CAPG PE=1 SV=2 | 24,96 | 25 | 28 | 50 |
| 578 | sp|P78344|IF4G2_HUMAN | HUMAN | Eukaryotic translation initiation factor 4 gamma 2 OS=Homo sapiens GN=EIF4G2 PE=1 SV=1 | 24,92 | 25,17 | 14 | 16,32 |
| 579 | sp|Q9ULA0|DNPEP_HUMAN | HUMAN | Aspartyl aminopeptidase OS=Homo sapiens GN=DNPEP PE=1 SV=1 | 24,92 | 25,05 | 17 | 48,84 |
| 580 | sp|Q9Y446|PKP3_HUMAN | HUMAN | Plakophilin-3 OS=Homo sapiens GN=PKP3 PE=1 SV=1 | 24,87 | 25,02 | 12 | 19,82 |
| 581 | sp|P42330|AK1C3_HUMAN | HUMAN | Aldo-keto reductase family 1 member C3 OS=Homo sapiens GN=AKR1C3 PE=1 SV=4 | 24,81 | 24,86 | 18 | 53,87 |
| 582 | sp|P55011|S12A2_HUMAN | HUMAN | Solute carrier family 12 member 2 OS=Homo sapiens GN=SLC12A2 PE=1 SV=1 | 24,8 | 25,34 | 16 | 14,11 |
| 583 | sp|P43652|AFAM_HUMAN | HUMAN | Afamin OS=Homo sapiens GN=AFM PE=1 SV=1 | 24,78 | 25,23 | 17 | 24,54 |
| 584 | sp|O15371|EIF3D_HUMAN | HUMAN | Eukaryotic translation initiation factor 3 subunit D OS=Homo sapiens GN=EIF3D PE=1 SV=1 | 24,75 | 24,81 | 19 | 39,23 |
| 585 | sp|P35611|ADDA_HUMAN | HUMAN | Alpha-adducin OS=Homo sapiens GN=ADD1 PE=1 SV=2 | 24,74 | 25,5 | 14 | 32,16 |
| 586 | sp|Q9UBT2|SAE2_HUMAN | HUMAN | SUMO-activating enzyme subunit 2 OS=Homo sapiens GN=UBA2 PE=1 SV=2 | 24,56 | 25,63 | 15 | 31,56 |
| 587 | sp|P40763|STAT3_HUMAN | HUMAN | Signal transducer and activator of transcription 3 OS=Homo sapiens GN=STAT3 PE=1 SV=2 | 24,55 | 24,68 | 16 | 26,23 |
| 588 | sp|P02649|APOE_HUMAN | HUMAN | Apolipoprotein E OS=Homo sapiens GN=APOE PE=1 SV=1 | 24,54 | 24,69 | 15 | 51,1 |
| 589 | sp|P07858|CATB_HUMAN | HUMAN | Cathepsin B OS=Homo sapiens GN=CTSB PE=1 SV=3 | 24,53 | 24,58 | 25 | 47,79 |
| 590 | sp|P46459|NSF_HUMAN | HUMAN | Vesicle-fusing ATPase OS=Homo sapiens GN=NSF PE=1 SV=3 | 24,46 | 26,92 | 15 | 21,37 |
| 591 | sp|Q9BS26|ERP44_HUMAN | HUMAN | Endoplasmic reticulum resident protein 44 OS=Homo sapiens GN=ERP44 PE=1 SV=1 | 24,46 | 24,53 | 15 | 41,13 |
| 592 | sp|Q96C19|EFHD2_HUMAN | HUMAN | EF-hand domain-containing protein D2 OS=Homo sapiens GN=EFHD2 PE=1 SV=1 | 24,43 | 24,52 | 16 | 50,83 |
| 593 | sp|A0MZ66|SHOT1_HUMAN | HUMAN | Shootin-1 OS=Homo sapiens GN=SHTN1 PE=1 SV=4 | 24,41 | 24,64 | 14 | 28,68 |
| 594 | sp|Q29940|1B59_HUMAN | HUMAN | HLA class I histocompatibility antigen, B-59 alpha chain OS=Homo sapiens GN=HLA-B PE=1 SV=1 | 24,41 | 24,44 | 16 | 42,82 |
| 595 | sp|P12532|KCRU_HUMAN | HUMAN | Creatine kinase U-type, mitochondrial OS=Homo sapiens GN=CKMT1A PE=1 SV=1 | 24,39 | 29,9 | 26 | 50,6 |
| 596 | sp|P05155|IC1_HUMAN | HUMAN | Plasma protease C1 inhibitor OS=Homo sapiens GN=SERPING1 PE=1 SV=2 | 24,31 | 24,32 | 15 | 26,6 |
| 597 | sp|Q6UX06|OLFM4_HUMAN | HUMAN | Olfactomedin-4 OS=Homo sapiens GN=OLFM4 PE=1 SV=1 | 24,24 | 24,3 | 16 | 39,22 |
| 598 | sp|P16949|STMN1_HUMAN | HUMAN | Stathmin OS=Homo sapiens GN=STMN1 PE=1 SV=3 | 24,23 | 24,32 | 21 | 62,42 |
| 599 | sp|P27348|1433T_HUMAN | HUMAN | 14-3-3 protein theta OS=Homo sapiens GN=YWHAQ PE=1 SV=1 | 24,22 | 35,27 | 47 | 64,9 |
| 600 | sp|Q13347|EIF3I_HUMAN | HUMAN | Eukaryotic translation initiation factor 3 subunit I OS=Homo sapiens GN=EIF3I PE=1 SV=1 | 24,15 | 24,17 | 14 | 54,46 |
| 601 | sp|Q13177|PAK2_HUMAN | HUMAN | Serine/threonine-protein kinase PAK 2 OS=Homo sapiens GN=PAK2 PE=1 SV=3 | 24,14 | 24,28 | 17 | 39,31 |
| 602 | sp|O14579|COPE_HUMAN | HUMAN | Coatomer subunit epsilon OS=Homo sapiens GN=COPE PE=1 SV=3 | 24,12 | 24,63 | 14 | 48,7 |
| 603 | sp|Q07866|KLC1_HUMAN | HUMAN | Kinesin light chain 1 OS=Homo sapiens GN=KLC1 PE=1 SV=2 | 24,11 | 24,22 | 14 | 28,27 |
| 604 | sp|P67775|PP2AA_HUMAN | HUMAN | Serine/threonine-protein phosphatase 2A catalytic subunit alpha isoform OS=Homo sapiens GN=PPP2CA PE=1 SV=1 | 24,08 | 24,17 | 15 | 56,31 |
| 605 | sp|P18283|GPX2_HUMAN | HUMAN | Glutathione peroxidase 2 OS=Homo sapiens GN=GPX2 PE=1 SV=3 | 24,06 | 24,09 | 17 | 83,16 |
| 606 | sp|P52789|HXK2_HUMAN | HUMAN | Hexokinase-2 OS=Homo sapiens GN=HK2 PE=1 SV=2 | 24,01 | 32,53 | 17 | 20,39 |
| 607 | sp|P02794|FRIH_HUMAN | HUMAN | Ferritin heavy chain OS=Homo sapiens GN=FTH1 PE=1 SV=2 | 24,01 | 24,01 | 18 | 72,13 |
| 608 | sp|O60749|SNX2_HUMAN | HUMAN | Sorting nexin-2 OS=Homo sapiens GN=SNX2 PE=1 SV=2 | 23,93 | 24 | 12 | 26,2 |
| 609 | sp|Q8WWA0|ITLN1_HUMAN | HUMAN | Intelectin-1 OS=Homo sapiens GN=ITLN1 PE=1 SV=1 | 23,86 | 23,93 | 18 | 62,94 |
| 610 | sp|P30044|PRDX5_HUMAN | HUMAN | Peroxiredoxin-5, mitochondrial OS=Homo sapiens GN=PRDX5 PE=1 SV=4 | 23,82 | 23,84 | 26 | 65,89 |
| 611 | sp|P30086|PEBP1_HUMAN | HUMAN | Phosphatidylethanolamine-binding protein 1 OS=Homo sapiens GN=PEBP1 PE=1 SV=3 | 23,79 | 23,83 | 28 | 83,96 |
| 612 | sp|O95336|6PGL_HUMAN | HUMAN | 6-phosphogluconolactonase OS=Homo sapiens GN=PGLS PE=1 SV=2 | 23,72 | 23,78 | 14 | 65,5 |
| 613 | sp|P27694|RFA1_HUMAN | HUMAN | Replication protein A 70 kDa DNA-binding subunit OS=Homo sapiens GN=RPA1 PE=1 SV=2 | 23,65 | 23,89 | 13 | 34,42 |
| 614 | sp|P15924|DESP_HUMAN | HUMAN | Desmoplakin OS=Homo sapiens GN=DSP PE=1 SV=3 | 23,63 | 25,95 | 15 | 5,573 |
| 615 | sp|P55036|PSMD4_HUMAN | HUMAN | 26S proteasome non-ATPase regulatory subunit 4 OS=Homo sapiens GN=PSMD4 PE=1 SV=1 | 23,6 | 23,79 | 14 | 41,91 |
| 616 | sp|Q12904|AIMP1_HUMAN | HUMAN | Aminoacyl tRNA synthase complex-interacting multifunctional protein 1 OS=Homo sapiens GN=AIMP1 PE=1 SV=2 | 23,59 | 23,72 | 16 | 59,29 |
| 617 | sp|P31153|METK2_HUMAN | HUMAN | S-adenosylmethionine synthase isoform type-2 OS=Homo sapiens GN=MAT2A PE=1 SV=1 | 23,58 | 23,68 | 15 | 43,29 |
| 618 | sp|P02748|CO9_HUMAN | HUMAN | Complement component C9 OS=Homo sapiens GN=C9 PE=1 SV=2 | 23,55 | 23,63 | 15 | 25,94 |
| 619 | sp|P01011|AACT_HUMAN | HUMAN | Alpha-1-antichymotrypsin OS=Homo sapiens GN=SERPINA3 PE=1 SV=2 | 23,43 | 23,97 | 17 | 39,01 |
| 620 | sp|Q9Y3A5|SBDS_HUMAN | HUMAN | Ribosome maturation protein SBDS OS=Homo sapiens GN=SBDS PE=1 SV=4 | 23,39 | 23,66 | 13 | 55,6 |
| 621 | sp|P04844|RPN2_HUMAN | HUMAN | Dolichyl-diphosphooligosaccharide--protein glycosyltransferase subunit 2 OS=Homo sapiens GN=RPN2 PE=1 SV=3 | 23,39 | 23,41 | 12 | 31,38 |
| 622 | sp|Q9BZZ5|API5_HUMAN | HUMAN | Apoptosis inhibitor 5 OS=Homo sapiens GN=API5 PE=1 SV=3 | 23,38 | 23,54 | 12 | 29,39 |
| 623 | sp|Q15436|SC23A_HUMAN | HUMAN | Protein transport protein Sec23A OS=Homo sapiens GN=SEC23A PE=1 SV=2 | 23,38 | 23,41 | 13 | 24,18 |
| 624 | sp|Q07955|SRSF1_HUMAN | HUMAN | Serine/arginine-rich splicing factor 1 OS=Homo sapiens GN=SRSF1 PE=1 SV=2 | 23,37 | 23,41 | 14 | 44,35 |
| 625 | sp|O43237|DC1L2_HUMAN | HUMAN | Cytoplasmic dynein 1 light intermediate chain 2 OS=Homo sapiens GN=DYNC1LI2 PE=1 SV=1 | 23,24 | 23,34 | 13 | 37,2 |
| 626 | sp|Q99733|NP1L4_HUMAN | HUMAN | Nucleosome assembly protein 1-like 4 OS=Homo sapiens GN=NAP1L4 PE=1 SV=1 | 23,19 | 23,25 | 15 | 53,6 |
| 627 | sp|P23193|TCEA1_HUMAN | HUMAN | Transcription elongation factor A protein 1 OS=Homo sapiens GN=TCEA1 PE=1 SV=2 | 23,18 | 23,65 | 12 | 39,87 |
| 628 | sp|Q96DG6|CMBL_HUMAN | HUMAN | Carboxymethylenebutenolidase homolog OS=Homo sapiens GN=CMBL PE=1 SV=1 | 23,18 | 23,6 | 13 | 48,57 |
| 629 | sp|Q01105|SET_HUMAN | HUMAN | Protein SET OS=Homo sapiens GN=SET PE=1 SV=3 | 23,18 | 23,22 | 19 | 44,14 |
| 630 | sp|P29350|PTN6_HUMAN | HUMAN | Tyrosine-protein phosphatase non-receptor type 6 OS=Homo sapiens GN=PTPN6 PE=1 SV=1 | 23,14 | 23,33 | 14 | 28,24 |
| 631 | sp|P21810|PGS1_HUMAN | HUMAN | Biglycan OS=Homo sapiens GN=BGN PE=1 SV=2 | 23,12 | 25,55 | 23 | 49,18 |
| 632 | sp|O95793|STAU1_HUMAN | HUMAN | Double-stranded RNA-binding protein Staufen homolog 1 OS=Homo sapiens GN=STAU1 PE=1 SV=2 | 23,11 | 23,54 | 12 | 27,21 |
| 633 | sp|Q9Y376|CAB39_HUMAN | HUMAN | Calcium-binding protein 39 OS=Homo sapiens GN=CAB39 PE=1 SV=1 | 23,1 | 23,2 | 11 | 29,91 |
| 634 | sp|P52566|GDIR2_HUMAN | HUMAN | Rho GDP-dissociation inhibitor 2 OS=Homo sapiens GN=ARHGDIB PE=1 SV=3 | 23,06 | 23,11 | 17 | 65,67 |
| 635 | sp|P09104|ENOG_HUMAN | HUMAN | Gamma-enolase OS=Homo sapiens GN=ENO2 PE=1 SV=3 | 23,03 | 34,34 | 26 | 62,21 |
| 636 | sp|Q99536|VAT1_HUMAN | HUMAN | Synaptic vesicle membrane protein VAT-1 homolog OS=Homo sapiens GN=VAT1 PE=1 SV=2 | 23,03 | 23,11 | 14 | 43,51 |
| 637 | sp|P29466|CASP1_HUMAN | HUMAN | Caspase-1 OS=Homo sapiens GN=CASP1 PE=1 SV=1 | 23,01 | 23,35 | 14 | 45,05 |
| 638 | sp|O95865|DDAH2_HUMAN | HUMAN | N(G),N(G)-dimethylarginine dimethylaminohydrolase 2 OS=Homo sapiens GN=DDAH2 PE=1 SV=1 | 23 | 25,24 | 13 | 68,07 |
| 639 | sp|P14317|HCLS1_HUMAN | HUMAN | Hematopoietic lineage cell-specific protein OS=Homo sapiens GN=HCLS1 PE=1 SV=3 | 22,99 | 23,16 | 12 | 29,84 |
| 640 | Biognosys_iRT ^(a)^ | iRT | Biognosys_iRT | 22,98 | 23 | 34 | 100 |
| 641 | sp|Q9UQ35|SRRM2_HUMAN | HUMAN | Serine/arginine repetitive matrix protein 2 OS=Homo sapiens GN=SRRM2 PE=1 SV=2 | 22,92 | 23,31 | 11 | 5,669 |
| 642 | sp|P35270|SPRE_HUMAN | HUMAN | Sepiapterin reductase OS=Homo sapiens GN=SPR PE=1 SV=1 | 22,91 | 22,99 | 13 | 50,57 |
| 643 | sp|Q9NQR4|NIT2_HUMAN | HUMAN | Omega-amidase NIT2 OS=Homo sapiens GN=NIT2 PE=1 SV=1 | 22,9 | 22,98 | 13 | 61,23 |
| 644 | sp|P51888|PRELP_HUMAN | HUMAN | Prolargin OS=Homo sapiens GN=PRELP PE=1 SV=1 | 22,84 | 22,9 | 13 | 40,05 |
| 645 | sp|P80723|BASP1_HUMAN | HUMAN | Brain acid soluble protein 1 OS=Homo sapiens GN=BASP1 PE=1 SV=2 | 22,81 | 22,84 | 14 | 74,89 |
| 646 | sp|Q9BXP5|SRRT_HUMAN | HUMAN | Serrate RNA effector molecule homolog OS=Homo sapiens GN=SRRT PE=1 SV=1 | 22,8 | 23,17 | 13 | 16,55 |
| 647 | sp|P17931|LEG3_HUMAN | HUMAN | Galectin-3 OS=Homo sapiens GN=LGALS3 PE=1 SV=5 | 22,78 | 23,01 | 30 | 50,8 |
| 648 | sp|Q96Q06|PLIN4_HUMAN | HUMAN | Perilipin-4 OS=Homo sapiens GN=PLIN4 PE=1 SV=2 | 22,67 | 22,92 | 12 | 26,46 |
| 649 | sp|P51149|RAB7A_HUMAN | HUMAN | Ras-related protein Rab-7a OS=Homo sapiens GN=RAB7A PE=1 SV=1 | 22,65 | 22,7 | 15 | 65,22 |
| 650 | sp|P21980|TGM2_HUMAN | HUMAN | Protein-glutamine gamma-glutamyltransferase 2 OS=Homo sapiens GN=TGM2 PE=1 SV=2 | 22,63 | 22,79 | 15 | 22,27 |
| 651 | sp|P46777|RL5_HUMAN | HUMAN | 60S ribosomal protein L5 OS=Homo sapiens GN=RPL5 PE=1 SV=3 | 22,61 | 22,7 | 19 | 45,79 |
| 652 | sp|P01859|IGHG2_HUMAN | HUMAN | Ig gamma-2 chain C region OS=Homo sapiens GN=IGHG2 PE=1 SV=2 | 22,59 | 43,27 | 64 | 75,15 |
| 653 | sp|P04004|VTNC_HUMAN | HUMAN | Vitronectin OS=Homo sapiens GN=VTN PE=1 SV=1 | 22,55 | 22,72 | 18 | 39,96 |
| 654 | sp|P55145|MANF_HUMAN | HUMAN | Mesencephalic astrocyte-derived neurotrophic factor OS=Homo sapiens GN=MANF PE=1 SV=3 | 22,55 | 22,64 | 16 | 58,79 |
| 655 | sp|Q15785|TOM34_HUMAN | HUMAN | Mitochondrial import receptor subunit TOM34 OS=Homo sapiens GN=TOMM34 PE=1 SV=2 | 22,5 | 22,78 | 13 | 49,84 |
| 656 | sp|P39019|RS19_HUMAN | HUMAN | 40S ribosomal protein S19 OS=Homo sapiens GN=RPS19 PE=1 SV=2 | 22,5 | 22,63 | 15 | 55,86 |
| 657 | sp|Q96FW1|OTUB1_HUMAN | HUMAN | Ubiquitin thioesterase OTUB1 OS=Homo sapiens GN=OTUB1 PE=1 SV=2 | 22,5 | 22,58 | 14 | 52,03 |
| 658 | sp|P51812|KS6A3_HUMAN | HUMAN | Ribosomal protein S6 kinase alpha-3 OS=Homo sapiens GN=RPS6KA3 PE=1 SV=1 | 22,49 | 22,7 | 12 | 19,32 |
| 659 | sp|P16278|BGAL_HUMAN | HUMAN | Beta-galactosidase OS=Homo sapiens GN=GLB1 PE=1 SV=2 | 22,46 | 22,63 | 11 | 22,3 |
| 660 | sp|P26368|U2AF2_HUMAN | HUMAN | Splicing factor U2AF 65 kDa subunit OS=Homo sapiens GN=U2AF2 PE=1 SV=4 | 22,42 | 22,48 | 16 | 48,42 |
| 661 | sp|P13861|KAP2_HUMAN | HUMAN | cAMP-dependent protein kinase type II-alpha regulatory subunit OS=Homo sapiens GN=PRKAR2A PE=1 SV=2 | 22,34 | 22,51 | 12 | 36,14 |
| 662 | sp|Q15907|RB11B_HUMAN | HUMAN | Ras-related protein Rab-11B OS=Homo sapiens GN=RAB11B PE=1 SV=4 | 22,32 | 22,41 | 12 | 54,13 |
| 663 | sp|Q14376|GALE_HUMAN | HUMAN | UDP-glucose 4-epimerase OS=Homo sapiens GN=GALE PE=1 SV=2 | 22,31 | 22,38 | 11 | 42,53 |
| 664 | sp|Q13642|FHL1_HUMAN | HUMAN | Four and a half LIM domains protein 1 OS=Homo sapiens GN=FHL1 PE=1 SV=4 | 22,29 | 22,3 | 17 | 41,18 |
| 665 | sp|Q16543|CDC37_HUMAN | HUMAN | Hsp90 co-chaperone Cdc37 OS=Homo sapiens GN=CDC37 PE=1 SV=1 | 22,27 | 22,44 | 13 | 31,75 |
| 666 | sp|O75390|CISY_HUMAN | HUMAN | Citrate synthase, mitochondrial OS=Homo sapiens GN=CS PE=1 SV=2 | 22,25 | 22,48 | 25 | 37,77 |
| 667 | sp|P52790|HXK3_HUMAN | HUMAN | Hexokinase-3 OS=Homo sapiens GN=HK3 PE=1 SV=2 | 22,24 | 25,52 | 17 | 21,24 |
| 668 | sp|P25205|MCM3_HUMAN | HUMAN | DNA replication licensing factor MCM3 OS=Homo sapiens GN=MCM3 PE=1 SV=3 | 22,24 | 22,45 | 12 | 18,07 |
| 669 | sp|P07602|SAP_HUMAN | HUMAN | Prosaposin OS=Homo sapiens GN=PSAP PE=1 SV=2 | 22,23 | 22,37 | 28 | 36,26 |
| 670 | sp|P04196|HRG_HUMAN | HUMAN | Histidine-rich glycoprotein OS=Homo sapiens GN=HRG PE=1 SV=1 | 22,23 | 22,33 | 12 | 30,1 |
| 671 | sp|P26196|DDX6_HUMAN | HUMAN | Probable ATP-dependent RNA helicase DDX6 OS=Homo sapiens GN=DDX6 PE=1 SV=2 | 22,2 | 24,38 | 13 | 33,75 |
| 672 | sp|P34897|GLYM_HUMAN | HUMAN | Serine hydroxymethyltransferase, mitochondrial OS=Homo sapiens GN=SHMT2 PE=1 SV=3 | 22,19 | 22,35 | 13 | 27,38 |
| 673 | sp|P31150|GDIA_HUMAN | HUMAN | Rab GDP dissociation inhibitor alpha OS=Homo sapiens GN=GDI1 PE=1 SV=2 | 22,14 | 46,64 | 29 | 58,17 |
| 674 | sp|O76094|SRP72_HUMAN | HUMAN | Signal recognition particle subunit SRP72 OS=Homo sapiens GN=SRP72 PE=1 SV=3 | 22,14 | 22,39 | 12 | 24,59 |
| 675 | sp|P15374|UCHL3_HUMAN | HUMAN | Ubiquitin carboxyl-terminal hydrolase isozyme L3 OS=Homo sapiens GN=UCHL3 PE=1 SV=1 | 22,12 | 22,22 | 14 | 76,52 |
| 676 | sp|Q96B97|SH3K1_HUMAN | HUMAN | SH3 domain-containing kinase-binding protein 1 OS=Homo sapiens GN=SH3KBP1 PE=1 SV=2 | 22,1 | 22,35 | 14 | 21,65 |
| 677 | sp|Q92820|GGH_HUMAN | HUMAN | Gamma-glutamyl hydrolase OS=Homo sapiens GN=GGH PE=1 SV=2 | 22,09 | 22,18 | 12 | 36,16 |
| 678 | sp|P50502|F10A1_HUMAN | HUMAN | Hsc70-interacting protein OS=Homo sapiens GN=ST13 PE=1 SV=2 | 22,07 | 22,21 | 15 | 29 |
| 679 | sp|P02792|FRIL_HUMAN | HUMAN | Ferritin light chain OS=Homo sapiens GN=FTL PE=1 SV=2 | 22,01 | 22,14 | 16 | 62,86 |
| 680 | sp|Q13630|FCL_HUMAN | HUMAN | GDP-L-fucose synthase OS=Homo sapiens GN=TSTA3 PE=1 SV=1 | 21,98 | 22,06 | 16 | 50,78 |
| 681 | sp|Q96C86|DCPS_HUMAN | HUMAN | m7GpppX diphosphatase OS=Homo sapiens GN=DCPS PE=1 SV=2 | 21,98 | 22,04 | 17 | 43,62 |
| 682 | sp|Q96CW1|AP2M1_HUMAN | HUMAN | AP-2 complex subunit mu OS=Homo sapiens GN=AP2M1 PE=1 SV=2 | 21,96 | 22,28 | 13 | 35,17 |
| 683 | sp|P61981|1433G_HUMAN | HUMAN | 14-3-3 protein gamma OS=Homo sapiens GN=YWHAG PE=1 SV=2 | 21,9 | 28,58 | 35 | 71,26 |
| 684 | sp|Q7L2H7|EIF3M_HUMAN | HUMAN | Eukaryotic translation initiation factor 3 subunit M OS=Homo sapiens GN=EIF3M PE=1 SV=1 | 21,87 | 22,89 | 13 | 47,06 |
| 685 | sp|Q9BWD1|THIC_HUMAN | HUMAN | Acetyl-CoA acetyltransferase, cytosolic OS=Homo sapiens GN=ACAT2 PE=1 SV=2 | 21,84 | 21,94 | 14 | 47,86 |
| 686 | sp|P22392|NDKB_HUMAN | HUMAN | Nucleoside diphosphate kinase B OS=Homo sapiens GN=NME2 PE=1 SV=1 | 21,82 | 21,88 | 30 | 81,58 |
| 687 | sp|O60218|AK1BA_HUMAN | HUMAN | Aldo-keto reductase family 1 member B10 OS=Homo sapiens GN=AKR1B10 PE=1 SV=2 | 21,81 | 21,94 | 12 | 41,46 |
| 688 | sp|P18085|ARF4_HUMAN | HUMAN | ADP-ribosylation factor 4 OS=Homo sapiens GN=ARF4 PE=1 SV=3 | 21,75 | 21,87 | 26 | 83,33 |
| 689 | sp|Q12792|TWF1_HUMAN | HUMAN | Twinfilin-1 OS=Homo sapiens GN=TWF1 PE=1 SV=3 | 21,74 | 22,48 | 14 | 38 |
| 690 | sp|O15305|PMM2_HUMAN | HUMAN | Phosphomannomutase 2 OS=Homo sapiens GN=PMM2 PE=1 SV=1 | 21,74 | 21,86 | 12 | 41,06 |
| 691 | sp|P61604|CH10_HUMAN | HUMAN | 10 kDa heat shock protein, mitochondrial OS=Homo sapiens GN=HSPE1 PE=1 SV=2 | 21,74 | 21,84 | 19 | 77,45 |
| 692 | sp|P30040|ERP29_HUMAN | HUMAN | Endoplasmic reticulum resident protein 29 OS=Homo sapiens GN=ERP29 PE=1 SV=4 | 21,68 | 21,77 | 17 | 55,94 |
| 693 | sp|P51610|HCFC1_HUMAN | HUMAN | Host cell factor 1 OS=Homo sapiens GN=HCFC1 PE=1 SV=2 | 21,67 | 22,02 | 13 | 10,57 |
| 694 | sp|P29966|MARCS_HUMAN | HUMAN | Myristoylated alanine-rich C-kinase substrate OS=Homo sapiens GN=MARCKS PE=1 SV=4 | 21,65 | 21,69 | 22 | 92,17 |
| 695 | sp|P26583|HMGB2_HUMAN | HUMAN | High mobility group protein B2 OS=Homo sapiens GN=HMGB2 PE=1 SV=2 | 21,64 | 27,5 | 25 | 60,29 |
| 696 | sp|Q9NVA2|SEP11_HUMAN | HUMAN | Septin-11 OS=Homo sapiens GN=SEPT11 PE=1 SV=3 | 21,62 | 23,98 | 17 | 47,32 |
| 697 | sp|O95861|BPNT1_HUMAN | HUMAN | 3'(2'),5'-bisphosphate nucleotidase 1 OS=Homo sapiens GN=BPNT1 PE=1 SV=1 | 21,62 | 22,29 | 13 | 45,45 |
| 698 | sp|Q9ULZ3|ASC_HUMAN | HUMAN | Apoptosis-associated speck-like protein containing a CARD OS=Homo sapiens GN=PYCARD PE=1 SV=2 | 21,6 | 21,65 | 15 | 72,82 |
| 699 | sp|P60660|MYL6_HUMAN | HUMAN | Myosin light polypeptide 6 OS=Homo sapiens GN=MYL6 PE=1 SV=2 | 21,59 | 21,62 | 29 | 88,74 |
| 700 | sp|Q641Q2|FA21A_HUMAN | HUMAN | WASH complex subunit FAM21A OS=Homo sapiens GN=FAM21A PE=1 SV=3 | 21,55 | 21,94 | 12 | 12,3 |
| 701 | sp|Q13162|PRDX4_HUMAN | HUMAN | Peroxiredoxin-4 OS=Homo sapiens GN=PRDX4 PE=1 SV=1 | 21,51 | 25,6 | 17 | 63,1 |
| 702 | sp|Q9BUF5|TBB6_HUMAN | HUMAN | Tubulin beta-6 chain OS=Homo sapiens GN=TUBB6 PE=1 SV=1 | 21,48 | 46,92 | 50 | 70,85 |
| 703 | sp|P00568|KAD1_HUMAN | HUMAN | Adenylate kinase isoenzyme 1 OS=Homo sapiens GN=AK1 PE=1 SV=3 | 21,47 | 21,56 | 13 | 67,01 |
| 704 | sp|Q9UKV3|ACINU_HUMAN | HUMAN | Apoptotic chromatin condensation inducer in the nucleus OS=Homo sapiens GN=ACIN1 PE=1 SV=2 | 21,44 | 22,08 | 12 | 13,57 |
| 705 | sp|P05387|RLA2_HUMAN | HUMAN | 60S acidic ribosomal protein P2 OS=Homo sapiens GN=RPLP2 PE=1 SV=1 | 21,42 | 21,5 | 21 | 93,91 |
| 706 | sp|P38919|IF4A3_HUMAN | HUMAN | Eukaryotic initiation factor 4A-III OS=Homo sapiens GN=EIF4A3 PE=1 SV=4 | 21,4 | 28,77 | 20 | 36,01 |
| 707 | sp|P46926|GNPI1_HUMAN | HUMAN | Glucosamine-6-phosphate isomerase 1 OS=Homo sapiens GN=GNPDA1 PE=1 SV=1 | 21,39 | 21,73 | 11 | 57,79 |
| 708 | sp|P23368|MAOM_HUMAN | HUMAN | NAD-dependent malic enzyme, mitochondrial OS=Homo sapiens GN=ME2 PE=1 SV=1 | 21,37 | 21,79 | 15 | 34,76 |
| 709 | sp|O43175|SERA_HUMAN | HUMAN | D-3-phosphoglycerate dehydrogenase OS=Homo sapiens GN=PHGDH PE=1 SV=4 | 21,35 | 21,43 | 14 | 27,77 |
| 710 | sp|Q9UBE0|SAE1_HUMAN | HUMAN | SUMO-activating enzyme subunit 1 OS=Homo sapiens GN=SAE1 PE=1 SV=1 | 21,26 | 21,56 | 12 | 40,46 |
| 711 | sp|O95340|PAPS2_HUMAN | HUMAN | Bifunctional 3'-phosphoadenosine 5'-phosphosulfate synthase 2 OS=Homo sapiens GN=PAPSS2 PE=1 SV=2 | 21,25 | 21,49 | 13 | 23,78 |
| 712 | sp|P0DMN0|ST1A4_HUMAN | HUMAN | Sulfotransferase 1A4 OS=Homo sapiens GN=SULT1A4 PE=1 SV=1 | 21,24 | 21,33 | 15 | 47,46 |
| 713 | sp|P05141|ADT2_HUMAN | HUMAN | ADP/ATP translocase 2 OS=Homo sapiens GN=SLC25A5 PE=1 SV=7 | 21,21 | 21,41 | 11 | 43,96 |
| 714 | sp|P57737|CORO7_HUMAN | HUMAN | Coronin-7 OS=Homo sapiens GN=CORO7 PE=1 SV=2 | 21,2 | 21,82 | 13 | 18,16 |
| 715 | sp|P28066|PSA5_HUMAN | HUMAN | Proteasome subunit alpha type-5 OS=Homo sapiens GN=PSMA5 PE=1 SV=3 | 21,18 | 21,27 | 16 | 60,58 |
| 716 | sp|Q14203|DCTN1_HUMAN | HUMAN | Dynactin subunit 1 OS=Homo sapiens GN=DCTN1 PE=1 SV=3 | 21,14 | 21,95 | 10 | 11,89 |
| 717 | sp|P48506|GSH1_HUMAN | HUMAN | Glutamate--cysteine ligase catalytic subunit OS=Homo sapiens GN=GCLC PE=1 SV=2 | 21,11 | 22,27 | 11 | 22,45 |
| 718 | sp|P25789|PSA4_HUMAN | HUMAN | Proteasome subunit alpha type-4 OS=Homo sapiens GN=PSMA4 PE=1 SV=1 | 21,1 | 21,18 | 20 | 52,11 |
| 719 | sp|P15170|ERF3A_HUMAN | HUMAN | Eukaryotic peptide chain release factor GTP-binding subunit ERF3A OS=Homo sapiens GN=GSPT1 PE=1 SV=1 | 21,09 | 21,23 | 12 | 27,05 |
| 720 | sp|Q8WWI1|LMO7_HUMAN | HUMAN | LIM domain only protein 7 OS=Homo sapiens GN=LMO7 PE=1 SV=3 | 21,07 | 21,43 | 12 | 10,52 |
| 721 | sp|P25685|DNJB1_HUMAN | HUMAN | DnaJ homolog subfamily B member 1 OS=Homo sapiens GN=DNAJB1 PE=1 SV=4 | 21,01 | 21,17 | 13 | 32,94 |
| 722 | sp|Q00688|FKBP3_HUMAN | HUMAN | Peptidyl-prolyl cis-trans isomerase FKBP3 OS=Homo sapiens GN=FKBP3 PE=1 SV=1 | 21 | 21,09 | 12 | 50 |
| 723 | sp|P00492|HPRT_HUMAN | HUMAN | Hypoxanthine-guanine phosphoribosyltransferase OS=Homo sapiens GN=HPRT1 PE=1 SV=2 | 20,95 | 20,99 | 11 | 63,3 |
| 724 | sp|P55327|TPD52_HUMAN | HUMAN | Tumor protein D52 OS=Homo sapiens GN=TPD52 PE=1 SV=2 | 20,86 | 20,94 | 15 | 63,84 |
| 725 | sp|P62979|RS27A_HUMAN | HUMAN | Ubiquitin-40S ribosomal protein S27a OS=Homo sapiens GN=RPS27A PE=1 SV=2 | 20,84 | 20,92 | 38 | 66,67 |
| 726 | sp|Q96HN2|SAHH3_HUMAN | HUMAN | Adenosylhomocysteinase 3 OS=Homo sapiens GN=AHCYL2 PE=1 SV=1 | 20,8 | 25,16 | 13 | 21,44 |
| 727 | sp|P25325|THTM_HUMAN | HUMAN | 3-mercaptopyruvate sulfurtransferase OS=Homo sapiens GN=MPST PE=1 SV=3 | 20,79 | 23,58 | 12 | 52,53 |
| 728 | sp|Q9Y2A7|NCKP1_HUMAN | HUMAN | Nck-associated protein 1 OS=Homo sapiens GN=NCKAP1 PE=1 SV=1 | 20,76 | 21,41 | 13 | 13,74 |
| 729 | sp|Q53GG5|PDLI3_HUMAN | HUMAN | PDZ and LIM domain protein 3 OS=Homo sapiens GN=PDLIM3 PE=1 SV=1 | 20,73 | 20,75 | 12 | 40,38 |
| 730 | sp|P24844|MYL9_HUMAN | HUMAN | Myosin regulatory light polypeptide 9 OS=Homo sapiens GN=MYL9 PE=1 SV=4 | 20,71 | 20,79 | 21 | 83,72 |
| 731 | sp|P43034|LIS1_HUMAN | HUMAN | Platelet-activating factor acetylhydrolase IB subunit alpha OS=Homo sapiens GN=PAFAH1B1 PE=1 SV=2 | 20,66 | 23,75 | 12 | 33,41 |
| 732 | sp|P12004|PCNA_HUMAN | HUMAN | Proliferating cell nuclear antigen OS=Homo sapiens GN=PCNA PE=1 SV=1 | 20,66 | 20,76 | 19 | 52,11 |
| 733 | sp|Q15417|CNN3_HUMAN | HUMAN | Calponin-3 OS=Homo sapiens GN=CNN3 PE=1 SV=1 | 20,6 | 25,59 | 22 | 44,98 |
| 734 | sp|Q15631|TSN_HUMAN | HUMAN | Translin OS=Homo sapiens GN=TSN PE=1 SV=1 | 20,58 | 20,68 | 13 | 49,56 |
| 735 | sp|P11908|PRPS2_HUMAN | HUMAN | Ribose-phosphate pyrophosphokinase 2 OS=Homo sapiens GN=PRPS2 PE=1 SV=2 | 20,57 | 20,66 | 15 | 49,37 |
| 736 | sp|P30084|ECHM_HUMAN | HUMAN | Enoyl-CoA hydratase, mitochondrial OS=Homo sapiens GN=ECHS1 PE=1 SV=4 | 20,56 | 20,67 | 13 | 47,59 |
| 737 | sp|Q9Y2S2|CRYL1_HUMAN | HUMAN | Lambda-crystallin homolog OS=Homo sapiens GN=CRYL1 PE=1 SV=3 | 20,54 | 20,66 | 13 | 51,1 |
| 738 | sp|Q9UEY8|ADDG_HUMAN | HUMAN | Gamma-adducin OS=Homo sapiens GN=ADD3 PE=1 SV=1 | 20,5 | 20,7 | 14 | 19,55 |
| 739 | sp|O95571|ETHE1_HUMAN | HUMAN | Persulfide dioxygenase ETHE1, mitochondrial OS=Homo sapiens GN=ETHE1 PE=1 SV=2 | 20,5 | 20,62 | 15 | 66,54 |
| 740 | sp|P02760|AMBP_HUMAN | HUMAN | Protein AMBP OS=Homo sapiens GN=AMBP PE=1 SV=1 | 20,5 | 20,57 | 15 | 39,49 |
| 741 | sp|Q14019|COTL1_HUMAN | HUMAN | Coactosin-like protein OS=Homo sapiens GN=COTL1 PE=1 SV=3 | 20,45 | 20,67 | 15 | 84,51 |
| 742 | sp|P61313|RL15_HUMAN | HUMAN | 60S ribosomal protein L15 OS=Homo sapiens GN=RPL15 PE=1 SV=2 | 20,45 | 20,64 | 14 | 49,51 |
| 743 | sp|P04632|CPNS1_HUMAN | HUMAN | Calpain small subunit 1 OS=Homo sapiens GN=CAPNS1 PE=1 SV=1 | 20,42 | 20,54 | 24 | 79,85 |
| 744 | sp|Q13409|DC1I2_HUMAN | HUMAN | Cytoplasmic dynein 1 intermediate chain 2 OS=Homo sapiens GN=DYNC1I2 PE=1 SV=3 | 20,4 | 20,75 | 13 | 31,35 |
| 745 | sp|P50213|IDH3A_HUMAN | HUMAN | Isocitrate dehydrogenase [NAD] subunit alpha, mitochondrial OS=Homo sapiens GN=IDH3A PE=1 SV=1 | 20,39 | 20,5 | 11 | 37,16 |
| 746 | sp|Q9Y224|CN166_HUMAN | HUMAN | UPF0568 protein C14orf166 OS=Homo sapiens GN=C14orf166 PE=1 SV=1 | 20,37 | 20,56 | 13 | 56,56 |
| 747 | sp|P40429|RL13A_HUMAN | HUMAN | 60S ribosomal protein L13a OS=Homo sapiens GN=RPL13A PE=1 SV=2 | 20,36 | 20,95 | 11 | 43,84 |
| 748 | sp|Q9Y6E0|STK24_HUMAN | HUMAN | Serine/threonine-protein kinase 24 OS=Homo sapiens GN=STK24 PE=1 SV=1 | 20,35 | 20,46 | 11 | 31,6 |
| 749 | sp|P55209|NP1L1_HUMAN | HUMAN | Nucleosome assembly protein 1-like 1 OS=Homo sapiens GN=NAP1L1 PE=1 SV=1 | 20,34 | 22,61 | 14 | 45,52 |
| 750 | sp|Q9BX66|SRBS1_HUMAN | HUMAN | Sorbin and SH3 domain-containing protein 1 OS=Homo sapiens GN=SORBS1 PE=1 SV=3 | 20,34 | 20,75 | 13 | 13,54 |
| 751 | sp|P62826|RAN_HUMAN | HUMAN | GTP-binding nuclear protein Ran OS=Homo sapiens GN=RAN PE=1 SV=3 | 20,33 | 20,46 | 18 | 50,46 |
| 752 | sp|O15355|PPM1G_HUMAN | HUMAN | Protein phosphatase 1G OS=Homo sapiens GN=PPM1G PE=1 SV=1 | 20,3 | 20,52 | 11 | 25,82 |
| 753 | sp|O75368|SH3L1_HUMAN | HUMAN | SH3 domain-binding glutamic acid-rich-like protein OS=Homo sapiens GN=SH3BGRL PE=1 SV=1 | 20,25 | 20,44 | 18 | 79,82 |
| 754 | sp|P61106|RAB14_HUMAN | HUMAN | Ras-related protein Rab-14 OS=Homo sapiens GN=RAB14 PE=1 SV=4 | 20,23 | 22,56 | 18 | 80,47 |
| 755 | sp|P60953|CDC42_HUMAN | HUMAN | Cell division control protein 42 homolog OS=Homo sapiens GN=CDC42 PE=1 SV=2 | 20,21 | 20,29 | 22 | 83,25 |
| 756 | sp|Q15717|ELAV1_HUMAN | HUMAN | ELAV-like protein 1 OS=Homo sapiens GN=ELAVL1 PE=1 SV=2 | 20,18 | 20,3 | 17 | 46,93 |
| 757 | sp|P07203|GPX1_HUMAN | HUMAN | Glutathione peroxidase 1 OS=Homo sapiens GN=GPX1 PE=1 SV=4 | 20,13 | 22,26 | 12 | 68,47 |
| 758 | sp|P51659|DHB4_HUMAN | HUMAN | Peroxisomal multifunctional enzyme type 2 OS=Homo sapiens GN=HSD17B4 PE=1 SV=3 | 20,1 | 20,81 | 10 | 20,92 |
| 759 | sp|P26373|RL13_HUMAN | HUMAN | 60S ribosomal protein L13 OS=Homo sapiens GN=RPL13 PE=1 SV=4 | 20,01 | 20,18 | 20 | 38,86 |
| 760 | sp|Q04760|LGUL_HUMAN | HUMAN | Lactoylglutathione lyase OS=Homo sapiens GN=GLO1 PE=1 SV=4 | 20 | 20,13 | 14 | 66,85 |
| 761 | sp|P20618|PSB1_HUMAN | HUMAN | Proteasome subunit beta type-1 OS=Homo sapiens GN=PSMB1 PE=1 SV=2 | 19,97 | 20,11 | 13 | 53,53 |
| 762 | sp|Q9HAT2|SIAE_HUMAN | HUMAN | Sialate O-acetylesterase OS=Homo sapiens GN=SIAE PE=1 SV=1 | 19,97 | 20,02 | 11 | 27,34 |
| 763 | sp|Q16853|AOC3_HUMAN | HUMAN | Membrane primary amine oxidase OS=Homo sapiens GN=AOC3 PE=1 SV=3 | 19,92 | 20,5 | 13 | 21,23 |
| 764 | sp|Q9NVD7|PARVA_HUMAN | HUMAN | Alpha-parvin OS=Homo sapiens GN=PARVA PE=1 SV=1 | 19,92 | 20,19 | 15 | 34,95 |
| 765 | sp|P02763|A1AG1_HUMAN | HUMAN | Alpha-1-acid glycoprotein 1 OS=Homo sapiens GN=ORM1 PE=1 SV=1 | 19,89 | 20,05 | 29 | 49,75 |
| 766 | sp|Q9BUJ2|HNRL1_HUMAN | HUMAN | Heterogeneous nuclear ribonucleoprotein U-like protein 1 OS=Homo sapiens GN=HNRNPUL1 PE=1 SV=2 | 19,87 | 21,74 | 14 | 18,93 |
| 767 | sp|P19623|SPEE_HUMAN | HUMAN | Spermidine synthase OS=Homo sapiens GN=SRM PE=1 SV=1 | 19,86 | 19,98 | 12 | 58,94 |
| 768 | sp|Q9H2U2|IPYR2_HUMAN | HUMAN | Inorganic pyrophosphatase 2, mitochondrial OS=Homo sapiens GN=PPA2 PE=1 SV=2 | 19,85 | 22,05 | 11 | 41,32 |
| 769 | sp|Q04917|1433F_HUMAN | HUMAN | 14-3-3 protein eta OS=Homo sapiens GN=YWHAH PE=1 SV=4 | 19,84 | 28,02 | 32 | 63,01 |
| 770 | sp|O00303|EIF3F_HUMAN | HUMAN | Eukaryotic translation initiation factor 3 subunit F OS=Homo sapiens GN=EIF3F PE=1 SV=1 | 19,81 | 19,95 | 12 | 35,85 |
| 771 | sp|P30043|BLVRB_HUMAN | HUMAN | Flavin reductase (NADPH) OS=Homo sapiens GN=BLVRB PE=1 SV=3 | 19,81 | 19,88 | 14 | 63,59 |
| 772 | sp|P24752|THIL_HUMAN | HUMAN | Acetyl-CoA acetyltransferase, mitochondrial OS=Homo sapiens GN=ACAT1 PE=1 SV=1 | 19,78 | 19,98 | 12 | 42,39 |
| 773 | sp|Q99459|CDC5L_HUMAN | HUMAN | Cell division cycle 5-like protein OS=Homo sapiens GN=CDC5L PE=1 SV=2 | 19,77 | 20 | 11 | 19,95 |
| 774 | sp|Q92597|NDRG1_HUMAN | HUMAN | Protein NDRG1 OS=Homo sapiens GN=NDRG1 PE=1 SV=1 | 19,73 | 19,76 | 12 | 48,73 |
| 775 | sp|Q08257|QOR_HUMAN | HUMAN | Quinone oxidoreductase OS=Homo sapiens GN=CRYZ PE=1 SV=1 | 19,69 | 19,86 | 11 | 51,37 |
| 776 | sp|Q9Y2W1|TR150_HUMAN | HUMAN | Thyroid hormone receptor-associated protein 3 OS=Homo sapiens GN=THRAP3 PE=1 SV=2 | 19,68 | 20 | 11 | 13,61 |
| 777 | sp|Q9UBQ7|GRHPR_HUMAN | HUMAN | Glyoxylate reductase/hydroxypyruvate reductase OS=Homo sapiens GN=GRHPR PE=1 SV=1 | 19,61 | 19,77 | 12 | 51,52 |
| 778 | sp|Q9UNF0|PACN2_HUMAN | HUMAN | Protein kinase C and casein kinase substrate in neurons protein 2 OS=Homo sapiens GN=PACSIN2 PE=1 SV=2 | 19,59 | 19,74 | 11 | 27,37 |
| 779 | sp|Q8WU39|MZB1_HUMAN | HUMAN | Marginal zone B- and B1-cell-specific protein OS=Homo sapiens GN=MZB1 PE=1 SV=1 | 19,58 | 19,67 | 25 | 71,43 |
| 780 | sp|Q9BXJ9|NAA15_HUMAN | HUMAN | N-alpha-acetyltransferase 15, NatA auxiliary subunit OS=Homo sapiens GN=NAA15 PE=1 SV=1 | 19,55 | 19,99 | 12 | 13,39 |
| 781 | sp|Q14914|PTGR1_HUMAN | HUMAN | Prostaglandin reductase 1 OS=Homo sapiens GN=PTGR1 PE=1 SV=2 | 19,54 | 19,63 | 12 | 46,81 |
| 782 | sp|P02511|CRYAB_HUMAN | HUMAN | Alpha-crystallin B chain OS=Homo sapiens GN=CRYAB PE=1 SV=2 | 19,49 | 19,54 | 12 | 66,86 |
| 783 | sp|Q14566|MCM6_HUMAN | HUMAN | DNA replication licensing factor MCM6 OS=Homo sapiens GN=MCM6 PE=1 SV=1 | 19,46 | 19,79 | 11 | 15,83 |
| 784 | sp|P05109|S10A8_HUMAN | HUMAN | Protein S100-A8 OS=Homo sapiens GN=S100A8 PE=1 SV=1 | 19,46 | 19,52 | 29 | 59,14 |
| 785 | sp|P09382|LEG1_HUMAN | HUMAN | Galectin-1 OS=Homo sapiens GN=LGALS1 PE=1 SV=2 | 19,45 | 19,5 | 23 | 91,11 |
| 786 | sp|P55010|IF5_HUMAN | HUMAN | Eukaryotic translation initiation factor 5 OS=Homo sapiens GN=EIF5 PE=1 SV=2 | 19,43 | 19,63 | 10 | 29,7 |
| 787 | sp|P02750|A2GL_HUMAN | HUMAN | Leucine-rich alpha-2-glycoprotein OS=Homo sapiens GN=LRG1 PE=1 SV=2 | 19,43 | 19,62 | 13 | 40,92 |
| 788 | sp|Q14498|RBM39_HUMAN | HUMAN | RNA-binding protein 39 OS=Homo sapiens GN=RBM39 PE=1 SV=2 | 19,41 | 19,55 | 12 | 29,43 |
| 789 | sp|P61019|RAB2A_HUMAN | HUMAN | Ras-related protein Rab-2A OS=Homo sapiens GN=RAB2A PE=1 SV=1 | 19,39 | 19,54 | 14 | 59,91 |
| 790 | sp|Q15637|SF01_HUMAN | HUMAN | Splicing factor 1 OS=Homo sapiens GN=SF1 PE=1 SV=4 | 19,34 | 19,4 | 12 | 20,19 |
| 791 | sp|Q9BRF8|CPPED_HUMAN | HUMAN | Serine/threonine-protein phosphatase CPPED1 OS=Homo sapiens GN=CPPED1 PE=1 SV=3 | 19,33 | 19,36 | 10 | 42,68 |
| 792 | sp|P09972|ALDOC_HUMAN | HUMAN | Fructose-bisphosphate aldolase C OS=Homo sapiens GN=ALDOC PE=1 SV=2 | 19,28 | 33,11 | 43 | 62,91 |
| 793 | sp|P30626|SORCN_HUMAN | HUMAN | Sorcin OS=Homo sapiens GN=SRI PE=1 SV=1 | 19,27 | 19,34 | 15 | 63,64 |
| 794 | sp|P49736|MCM2_HUMAN | HUMAN | DNA replication licensing factor MCM2 OS=Homo sapiens GN=MCM2 PE=1 SV=4 | 19,24 | 19,72 | 12 | 14,71 |
| 795 | sp|P61289|PSME3_HUMAN | HUMAN | Proteasome activator complex subunit 3 OS=Homo sapiens GN=PSME3 PE=1 SV=1 | 19,24 | 19,34 | 12 | 42,91 |
| 796 | sp|P47755|CAZA2_HUMAN | HUMAN | F-actin-capping protein subunit alpha-2 OS=Homo sapiens GN=CAPZA2 PE=1 SV=3 | 19,23 | 25,23 | 26 | 76,22 |
| 797 | sp|Q04446|GLGB_HUMAN | HUMAN | 1,4-alpha-glucan-branching enzyme OS=Homo sapiens GN=GBE1 PE=1 SV=3 | 19,19 | 19,46 | 11 | 23,36 |
| 798 | sp|Q15424|SAFB1_HUMAN | HUMAN | Scaffold attachment factor B1 OS=Homo sapiens GN=SAFB PE=1 SV=4 | 19,16 | 19,4 | 14 | 23,93 |
| 799 | sp|Q14444|CAPR1_HUMAN | HUMAN | Caprin-1 OS=Homo sapiens GN=CAPRIN1 PE=1 SV=2 | 19,15 | 19,31 | 11 | 25,81 |
| 800 | sp|Q9UJ70|NAGK_HUMAN | HUMAN | N-acetyl-D-glucosamine kinase OS=Homo sapiens GN=NAGK PE=1 SV=4 | 19,14 | 19,47 | 12 | 38,66 |
| 801 | sp|Q14126|DSG2_HUMAN | HUMAN | Desmoglein-2 OS=Homo sapiens GN=DSG2 PE=1 SV=2 | 19,13 | 19,89 | 10 | 12,34 |
| 802 | sp|P54652|HSP72_HUMAN | HUMAN | Heat shock-related 70 kDa protein 2 OS=Homo sapiens GN=HSPA2 PE=1 SV=1 | 19,12 | 42,46 | 48 | 38,81 |
| 803 | sp|P61163|ACTZ_HUMAN | HUMAN | Alpha-centractin OS=Homo sapiens GN=ACTR1A PE=1 SV=1 | 19,1 | 19,34 | 15 | 43,88 |
| 804 | sp|Q10567|AP1B1_HUMAN | HUMAN | AP-1 complex subunit beta-1 OS=Homo sapiens GN=AP1B1 PE=1 SV=2 | 19,08 | 44,49 | 30 | 31,61 |
| 805 | sp|Q13011|ECH1_HUMAN | HUMAN | Delta(3,5)-Delta(2,4)-dienoyl-CoA isomerase, mitochondrial OS=Homo sapiens GN=ECH1 PE=1 SV=2 | 19,08 | 19,23 | 13 | 41,77 |
| 806 | sp|O14744|ANM5_HUMAN | HUMAN | Protein arginine N-methyltransferase 5 OS=Homo sapiens GN=PRMT5 PE=1 SV=4 | 19,03 | 19,42 | 11 | 20,25 |
| 807 | sp|Q99729|ROAA_HUMAN | HUMAN | Heterogeneous nuclear ribonucleoprotein A/B OS=Homo sapiens GN=HNRNPAB PE=1 SV=2 | 19,02 | 22,28 | 18 | 30,72 |
| 808 | sp|P10644|KAP0_HUMAN | HUMAN | cAMP-dependent protein kinase type I-alpha regulatory subunit OS=Homo sapiens GN=PRKAR1A PE=1 SV=1 | 19,01 | 19,23 | 13 | 34,91 |
| 809 | sp|Q07065|CKAP4_HUMAN | HUMAN | Cytoskeleton-associated protein 4 OS=Homo sapiens GN=CKAP4 PE=1 SV=2 | 18,97 | 19,29 | 10 | 20,43 |
| 810 | sp|Q9BT78|CSN4_HUMAN | HUMAN | COP9 signalosome complex subunit 4 OS=Homo sapiens GN=COPS4 PE=1 SV=1 | 18,94 | 19,04 | 10 | 31,03 |
| 811 | sp|Q5R3I4|TTC38_HUMAN | HUMAN | Tetratricopeptide repeat protein 38 OS=Homo sapiens GN=TTC38 PE=1 SV=1 | 18,91 | 18,96 | 10 | 23,67 |
| 812 | sp|P34913|HYES_HUMAN | HUMAN | Bifunctional epoxide hydrolase 2 OS=Homo sapiens GN=EPHX2 PE=1 SV=2 | 18,86 | 19,02 | 12 | 29,37 |
| 813 | sp|O14558|HSPB6_HUMAN | HUMAN | Heat shock protein beta-6 OS=Homo sapiens GN=HSPB6 PE=1 SV=2 | 18,83 | 18,86 | 15 | 78,75 |
| 814 | sp|P07741|APT_HUMAN | HUMAN | Adenine phosphoribosyltransferase OS=Homo sapiens GN=APRT PE=1 SV=2 | 18,79 | 18,86 | 19 | 70,56 |
| 815 | sp|P08311|CATG_HUMAN | HUMAN | Cathepsin G OS=Homo sapiens GN=CTSG PE=1 SV=2 | 18,76 | 18,84 | 12 | 43,92 |
| 816 | sp|O95994|AGR2_HUMAN | HUMAN | Anterior gradient protein 2 homolog OS=Homo sapiens GN=AGR2 PE=1 SV=1 | 18,74 | 19,44 | 30 | 68,57 |
| 817 | sp|P54886|P5CS_HUMAN | HUMAN | Delta-1-pyrroline-5-carboxylate synthase OS=Homo sapiens GN=ALDH18A1 PE=1 SV=2 | 18,7 | 18,92 | 11 | 17,99 |
| 818 | sp|Q01469|FABP5_HUMAN | HUMAN | Fatty acid-binding protein, epidermal OS=Homo sapiens GN=FABP5 PE=1 SV=3 | 18,7 | 18,83 | 20 | 72,59 |
| 819 | sp|Q14157|UBP2L_HUMAN | HUMAN | Ubiquitin-associated protein 2-like OS=Homo sapiens GN=UBAP2L PE=1 SV=2 | 18,62 | 18,74 | 11 | 13,8 |
| 820 | sp|P10253|LYAG_HUMAN | HUMAN | Lysosomal alpha-glucosidase OS=Homo sapiens GN=GAA PE=1 SV=4 | 18,59 | 19,36 | 14 | 16,81 |
| 821 | sp|O76003|GLRX3_HUMAN | HUMAN | Glutaredoxin-3 OS=Homo sapiens GN=GLRX3 PE=1 SV=2 | 18,57 | 18,68 | 12 | 45,67 |
| 822 | sp|O95834|EMAL2_HUMAN | HUMAN | Echinoderm microtubule-associated protein-like 2 OS=Homo sapiens GN=EML2 PE=1 SV=1 | 18,55 | 18,75 | 10 | 20,96 |
| 823 | sp|O75347|TBCA_HUMAN | HUMAN | Tubulin-specific chaperone A OS=Homo sapiens GN=TBCA PE=1 SV=3 | 18,54 | 18,61 | 15 | 59,26 |
| 824 | sp|Q9HB71|CYBP_HUMAN | HUMAN | Calcyclin-binding protein OS=Homo sapiens GN=CACYBP PE=1 SV=2 | 18,53 | 18,77 | 13 | 62,28 |
| 825 | sp|P25311|ZA2G_HUMAN | HUMAN | Zinc-alpha-2-glycoprotein OS=Homo sapiens GN=AZGP1 PE=1 SV=2 | 18,52 | 18,7 | 14 | 37,58 |
| 826 | sp|Q7Z4W1|DCXR_HUMAN | HUMAN | L-xylulose reductase OS=Homo sapiens GN=DCXR PE=1 SV=2 | 18,52 | 18,63 | 9 | 43,44 |
| 827 | sp|P51148|RAB5C_HUMAN | HUMAN | Ras-related protein Rab-5C OS=Homo sapiens GN=RAB5C PE=1 SV=2 | 18,48 | 18,53 | 12 | 58,8 |
| 828 | sp|Q16836|HCDH_HUMAN | HUMAN | Hydroxyacyl-coenzyme A dehydrogenase, mitochondrial OS=Homo sapiens GN=HADH PE=1 SV=3 | 18,45 | 18,67 | 16 | 50 |
| 829 | sp|P62917|RL8_HUMAN | HUMAN | 60S ribosomal protein L8 OS=Homo sapiens GN=RPL8 PE=1 SV=2 | 18,4 | 18,59 | 14 | 49,42 |
| 830 | sp|P39060|COIA1_HUMAN | HUMAN | Collagen alpha-1(XVIII) chain OS=Homo sapiens GN=COL18A1 PE=1 SV=5 | 18,37 | 18,53 | 12 | 9,635 |
| 831 | sp|Q96C23|GALM_HUMAN | HUMAN | Aldose 1-epimerase OS=Homo sapiens GN=GALM PE=1 SV=1 | 18,33 | 18,4 | 12 | 41,52 |
| 832 | sp|P21964|COMT_HUMAN | HUMAN | Catechol O-methyltransferase OS=Homo sapiens GN=COMT PE=1 SV=2 | 18,33 | 18,38 | 13 | 55,35 |
| 833 | sp|O43396|TXNL1_HUMAN | HUMAN | Thioredoxin-like protein 1 OS=Homo sapiens GN=TXNL1 PE=1 SV=3 | 18,33 | 18,38 | 11 | 50,17 |
| 834 | sp|P62280|RS11_HUMAN | HUMAN | 40S ribosomal protein S11 OS=Homo sapiens GN=RPS11 PE=1 SV=3 | 18,32 | 18,51 | 12 | 51,27 |
| 835 | sp|P00736|C1R_HUMAN | HUMAN | Complement C1r subcomponent OS=Homo sapiens GN=C1R PE=1 SV=2 | 18,31 | 18,33 | 11 | 19,15 |
| 836 | sp|O95154|ARK73_HUMAN | HUMAN | Aflatoxin B1 aldehyde reductase member 3 OS=Homo sapiens GN=AKR7A3 PE=1 SV=2 | 18,27 | 18,29 | 10 | 35,65 |
| 837 | sp|P62906|RL10A_HUMAN | HUMAN | 60S ribosomal protein L10a OS=Homo sapiens GN=RPL10A PE=1 SV=2 | 18,26 | 19,25 | 12 | 46,54 |
| 838 | sp|Q86V81|THOC4_HUMAN | HUMAN | THO complex subunit 4 OS=Homo sapiens GN=ALYREF PE=1 SV=3 | 18,26 | 18,31 | 11 | 36,19 |
| 839 | sp|P52565|GDIR1_HUMAN | HUMAN | Rho GDP-dissociation inhibitor 1 OS=Homo sapiens GN=ARHGDIA PE=1 SV=3 | 18,23 | 18,32 | 17 | 77,45 |
| 840 | sp|Q9Y6W5|WASF2_HUMAN | HUMAN | Wiskott-Aldrich syndrome protein family member 2 OS=Homo sapiens GN=WASF2 PE=1 SV=3 | 18,22 | 18,41 | 11 | 29,92 |
| 841 | sp|Q09028|RBBP4_HUMAN | HUMAN | Histone-binding protein RBBP4 OS=Homo sapiens GN=RBBP4 PE=1 SV=3 | 18,21 | 18,43 | 11 | 24,24 |
| 842 | sp|O00764|PDXK_HUMAN | HUMAN | Pyridoxal kinase OS=Homo sapiens GN=PDXK PE=1 SV=1 | 18,17 | 18,26 | 15 | 44,87 |
| 843 | sp|Q9Y6E2|BZW2_HUMAN | HUMAN | Basic leucine zipper and W2 domain-containing protein 2 OS=Homo sapiens GN=BZW2 PE=1 SV=1 | 18,16 | 26,09 | 12 | 27,45 |
| 844 | sp|P50995|ANX11_HUMAN | HUMAN | Annexin A11 OS=Homo sapiens GN=ANXA11 PE=1 SV=1 | 18,15 | 19,69 | 11 | 23,17 |
| 845 | sp|Q13618|CUL3_HUMAN | HUMAN | Cullin-3 OS=Homo sapiens GN=CUL3 PE=1 SV=2 | 18,1 | 19,66 | 11 | 14,84 |
| 846 | sp|Q9UKK9|NUDT5_HUMAN | HUMAN | ADP-sugar pyrophosphatase OS=Homo sapiens GN=NUDT5 PE=1 SV=1 | 18,1 | 18,25 | 15 | 64,38 |
| 847 | sp|P06865|HEXA_HUMAN | HUMAN | Beta-hexosaminidase subunit alpha OS=Homo sapiens GN=HEXA PE=1 SV=2 | 18,08 | 20,05 | 11 | 27,6 |
| 848 | sp|P27635|RL10_HUMAN | HUMAN | 60S ribosomal protein L10 OS=Homo sapiens GN=RPL10 PE=1 SV=4 | 18,03 | 18,24 | 18 | 50,47 |
| 849 | sp|P31946|1433B_HUMAN | HUMAN | 14-3-3 protein beta/alpha OS=Homo sapiens GN=YWHAB PE=1 SV=3 | 18,02 | 32,82 | 54 | 76,42 |
| 850 | sp|P10155|RO60_HUMAN | HUMAN | 60 kDa SS-A/Ro ribonucleoprotein OS=Homo sapiens GN=TROVE2 PE=1 SV=2 | 18,01 | 18,24 | 11 | 22,86 |
| 851 | sp|Q9Y2B0|CNPY2_HUMAN | HUMAN | Protein canopy homolog 2 OS=Homo sapiens GN=CNPY2 PE=1 SV=1 | 18,01 | 18,01 | 11 | 63,74 |
| 852 | sp|O43684|BUB3_HUMAN | HUMAN | Mitotic checkpoint protein BUB3 OS=Homo sapiens GN=BUB3 PE=1 SV=1 | 17,99 | 18,04 | 11 | 34,76 |
| 853 | sp|P46109|CRKL_HUMAN | HUMAN | Crk-like protein OS=Homo sapiens GN=CRKL PE=1 SV=1 | 17,98 | 18,02 | 11 | 49,17 |
| 854 | sp|P53634|CATC_HUMAN | HUMAN | Dipeptidyl peptidase 1 OS=Homo sapiens GN=CTSC PE=1 SV=2 | 17,96 | 18,05 | 10 | 24,41 |
| 855 | sp|P61224|RAP1B_HUMAN | HUMAN | Ras-related protein Rap-1b OS=Homo sapiens GN=RAP1B PE=1 SV=1 | 17,91 | 17,99 | 11 | 71,74 |
| 856 | sp|Q9UMX0|UBQL1_HUMAN | HUMAN | Ubiquilin-1 OS=Homo sapiens GN=UBQLN1 PE=1 SV=2 | 17,89 | 17,93 | 14 | 35,31 |
| 857 | sp|O94874|UFL1_HUMAN | HUMAN | E3 UFM1-protein ligase 1 OS=Homo sapiens GN=UFL1 PE=1 SV=2 | 17,87 | 19,43 | 13 | 17,76 |
| 858 | sp|P30566|PUR8_HUMAN | HUMAN | Adenylosuccinate lyase OS=Homo sapiens GN=ADSL PE=1 SV=2 | 17,86 | 17,99 | 9 | 29,96 |
| 859 | sp|P69892|HBG2_HUMAN | HUMAN | Hemoglobin subunit gamma-2 OS=Homo sapiens GN=HBG2 PE=1 SV=2 | 17,83 | 22,36 | 40 | 76,87 |
| 860 | sp|O95433|AHSA1_HUMAN | HUMAN | Activator of 90 kDa heat shock protein ATPase homolog 1 OS=Homo sapiens GN=AHSA1 PE=1 SV=1 | 17,77 | 18 | 13 | 39,94 |
| 861 | sp|P04179|SODM_HUMAN | HUMAN | Superoxide dismutase [Mn], mitochondrial OS=Homo sapiens GN=SOD2 PE=1 SV=2 | 17,75 | 17,86 | 31 | 66,67 |
| 862 | sp|P24534|EF1B_HUMAN | HUMAN | Elongation factor 1-beta OS=Homo sapiens GN=EEF1B2 PE=1 SV=3 | 17,73 | 24,06 | 22 | 70,22 |
| 863 | sp|P53992|SC24C_HUMAN | HUMAN | Protein transport protein Sec24C OS=Homo sapiens GN=SEC24C PE=1 SV=3 | 17,73 | 17,87 | 10 | 12,16 |
| 864 | sp|B9A064|IGLL5_HUMAN | HUMAN | Immunoglobulin lambda-like polypeptide 5 OS=Homo sapiens GN=IGLL5 PE=2 SV=2 | 17,73 | 17,78 | 28 | 44,39 |
| 865 | sp|P61088|UBE2N_HUMAN | HUMAN | Ubiquitin-conjugating enzyme E2 N OS=Homo sapiens GN=UBE2N PE=1 SV=1 | 17,66 | 17,72 | 15 | 71,71 |
| 866 | sp|P32969|RL9_HUMAN | HUMAN | 60S ribosomal protein L9 OS=Homo sapiens GN=RPL9 PE=1 SV=1 | 17,66 | 17,71 | 16 | 65,1 |
| 867 | sp|Q13442|HAP28_HUMAN | HUMAN | 28 kDa heat- and acid-stable phosphoprotein OS=Homo sapiens GN=PDAP1 PE=1 SV=1 | 17,62 | 18 | 14 | 48,07 |
| 868 | sp|Q13045|FLII_HUMAN | HUMAN | Protein flightless-1 homolog OS=Homo sapiens GN=FLII PE=1 SV=2 | 17,62 | 17,9 | 14 | 11,98 |
| 869 | sp|O00567|NOP56_HUMAN | HUMAN | Nucleolar protein 56 OS=Homo sapiens GN=NOP56 PE=1 SV=4 | 17,6 | 17,83 | 11 | 26,26 |
| 870 | sp|P01834|IGKC_HUMAN | HUMAN | Ig kappa chain C region OS=Homo sapiens GN=IGKC PE=1 SV=1 | 17,58 | 17,63 | 67 | 93,4 |
| 871 | sp|Q53H82|LACB2_HUMAN | HUMAN | Beta-lactamase-like protein 2 OS=Homo sapiens GN=LACTB2 PE=1 SV=2 | 17,52 | 17,78 | 12 | 47,22 |
| 872 | sp|O43704|ST1B1_HUMAN | HUMAN | Sulfotransferase family cytosolic 1B member 1 OS=Homo sapiens GN=SULT1B1 PE=1 SV=2 | 17,51 | 17,69 | 10 | 38,18 |
| 873 | sp|Q15056|IF4H_HUMAN | HUMAN | Eukaryotic translation initiation factor 4H OS=Homo sapiens GN=EIF4H PE=1 SV=5 | 17,49 | 17,68 | 12 | 44,35 |
| 874 | sp|P10599|THIO_HUMAN | HUMAN | Thioredoxin OS=Homo sapiens GN=TXN PE=1 SV=3 | 17,47 | 17,51 | 20 | 89,52 |
| 875 | sp|P07148|FABPL_HUMAN | HUMAN | Fatty acid-binding protein, liver OS=Homo sapiens GN=FABP1 PE=1 SV=1 | 17,43 | 17,5 | 36 | 81,1 |
| 876 | sp|P02786|TFR1_HUMAN | HUMAN | Transferrin receptor protein 1 OS=Homo sapiens GN=TFRC PE=1 SV=2 | 17,41 | 17,73 | 11 | 18,42 |
| 877 | sp|Q9Y5P6|GMPPB_HUMAN | HUMAN | Mannose-1-phosphate guanyltransferase beta OS=Homo sapiens GN=GMPPB PE=1 SV=2 | 17,41 | 17,49 | 9 | 36,94 |
| 878 | sp|P36957|ODO2_HUMAN | HUMAN | Dihydrolipoyllysine-residue succinyltransferase component of 2-oxoglutarate dehydrogenase complex, mitochondrial OS=Homo sapiens GN=DLST PE=1 SV=4 | 17,4 | 17,54 | 10 | 24,28 |
| 879 | sp|P51580|TPMT_HUMAN | HUMAN | Thiopurine S-methyltransferase OS=Homo sapiens GN=TPMT PE=1 SV=1 | 17,36 | 17,53 | 10 | 39,59 |
| 880 | sp|P83731|RL24_HUMAN | HUMAN | 60S ribosomal protein L24 OS=Homo sapiens GN=RPL24 PE=1 SV=1 | 17,36 | 17,51 | 15 | 46,5 |
| 881 | sp|P62993|GRB2_HUMAN | HUMAN | Growth factor receptor-bound protein 2 OS=Homo sapiens GN=GRB2 PE=1 SV=1 | 17,31 | 17,48 | 11 | 58,53 |
| 882 | sp|O14773|TPP1_HUMAN | HUMAN | Tripeptidyl-peptidase 1 OS=Homo sapiens GN=TPP1 PE=1 SV=2 | 17,28 | 17,34 | 13 | 30,91 |
| 883 | sp|P80188|NGAL_HUMAN | HUMAN | Neutrophil gelatinase-associated lipocalin OS=Homo sapiens GN=LCN2 PE=1 SV=2 | 17,27 | 17,4 | 15 | 65,15 |
| 884 | sp|O43747|AP1G1_HUMAN | HUMAN | AP-1 complex subunit gamma-1 OS=Homo sapiens GN=AP1G1 PE=1 SV=5 | 17,25 | 17,51 | 9 | 13,26 |
| 885 | sp|P08708|RS17_HUMAN | HUMAN | 40S ribosomal protein S17 OS=Homo sapiens GN=RPS17 PE=1 SV=2 | 17,25 | 17,29 | 18 | 61,48 |
| 886 | sp|P06731|CEAM5_HUMAN | HUMAN | Carcinoembryonic antigen-related cell adhesion molecule 5 OS=Homo sapiens GN=CEACAM5 PE=1 SV=3 | 17,24 | 17,35 | 19 | 21,79 |
| 887 | sp|P30085|KCY_HUMAN | HUMAN | UMP-CMP kinase OS=Homo sapiens GN=CMPK1 PE=1 SV=3 | 17,23 | 17,3 | 12 | 53,06 |
| 888 | sp|P22061|PIMT_HUMAN | HUMAN | Protein-L-isoaspartate(D-aspartate) O-methyltransferase OS=Homo sapiens GN=PCMT1 PE=1 SV=4 | 17,22 | 17,28 | 11 | 61,23 |
| 889 | sp|Q9H773|DCTP1_HUMAN | HUMAN | dCTP pyrophosphatase 1 OS=Homo sapiens GN=DCTPP1 PE=1 SV=1 | 17,19 | 17,27 | 11 | 54,12 |
| 890 | sp|P08294|SODE_HUMAN | HUMAN | Extracellular superoxide dismutase [Cu-Zn] OS=Homo sapiens GN=SOD3 PE=1 SV=2 | 17,18 | 17,22 | 9 | 62,5 |
| 891 | sp|P61758|PFD3_HUMAN | HUMAN | Prefoldin subunit 3 OS=Homo sapiens GN=VBP1 PE=1 SV=3 | 17,14 | 17,33 | 10 | 53,81 |
| 892 | sp|P49589|SYCC_HUMAN | HUMAN | Cysteine--tRNA ligase, cytoplasmic OS=Homo sapiens GN=CARS PE=1 SV=3 | 17,07 | 17,36 | 10 | 13,37 |
| 893 | sp|Q13126|MTAP_HUMAN | HUMAN | S-methyl-5'-thioadenosine phosphorylase OS=Homo sapiens GN=MTAP PE=1 SV=2 | 17,07 | 17,25 | 10 | 51,59 |
| 894 | sp|P63000|RAC1_HUMAN | HUMAN | Ras-related C3 botulinum toxin substrate 1 OS=Homo sapiens GN=RAC1 PE=1 SV=1 | 17,05 | 19,33 | 13 | 57,81 |
| 895 | sp|P61011|SRP54_HUMAN | HUMAN | Signal recognition particle 54 kDa protein OS=Homo sapiens GN=SRP54 PE=1 SV=1 | 17,05 | 17,27 | 9 | 25,4 |
| 896 | sp|P43487|RANG_HUMAN | HUMAN | Ran-specific GTPase-activating protein OS=Homo sapiens GN=RANBP1 PE=1 SV=1 | 17,03 | 18,25 | 12 | 70,65 |
| 897 | sp|Q02817|MUC2_HUMAN | HUMAN | Mucin-2 OS=Homo sapiens GN=MUC2 PE=1 SV=2 | 17,01 | 17,24 | 10 | 3,263 |
| 898 | sp|P06454|PTMA_HUMAN | HUMAN | Prothymosin alpha OS=Homo sapiens GN=PTMA PE=1 SV=2 | 17,01 | 17,05 | 31 | 43,24 |
| 899 | sp|P53367|ARFP1_HUMAN | HUMAN | Arfaptin-1 OS=Homo sapiens GN=ARFIP1 PE=1 SV=2 | 17 | 17,15 | 12 | 39,41 |
| 900 | sp|Q15020|SART3_HUMAN | HUMAN | Squamous cell carcinoma antigen recognized by T-cells 3 OS=Homo sapiens GN=SART3 PE=1 SV=1 | 16,97 | 17,61 | 11 | 13,19 |
| 901 | sp|Q9Y315|DEOC_HUMAN | HUMAN | Deoxyribose-phosphate aldolase OS=Homo sapiens GN=DERA PE=1 SV=2 | 16,96 | 17,08 | 10 | 40,25 |
| 902 | sp|P46783|RS10_HUMAN | HUMAN | 40S ribosomal protein S10 OS=Homo sapiens GN=RPS10 PE=1 SV=1 | 16,89 | 17,25 | 12 | 57,58 |
| 903 | sp|Q13185|CBX3_HUMAN | HUMAN | Chromobox protein homolog 3 OS=Homo sapiens GN=CBX3 PE=1 SV=4 | 16,88 | 17,02 | 10 | 56,83 |
| 904 | sp|O75821|EIF3G_HUMAN | HUMAN | Eukaryotic translation initiation factor 3 subunit G OS=Homo sapiens GN=EIF3G PE=1 SV=2 | 16,84 | 17,06 | 11 | 34,69 |
| 905 | sp|P22059|OSBP1_HUMAN | HUMAN | Oxysterol-binding protein 1 OS=Homo sapiens GN=OSBP PE=1 SV=1 | 16,82 | 17,46 | 10 | 16,48 |
| 906 | sp|P16403|H12_HUMAN | HUMAN | Histone H1.2 OS=Homo sapiens GN=HIST1H1C PE=1 SV=2 | 16,78 | 16,83 | 10 | 31,92 |
| 907 | sp|P52306|GDS1_HUMAN | HUMAN | Rap1 GTPase-GDP dissociation stimulator 1 OS=Homo sapiens GN=RAP1GDS1 PE=1 SV=3 | 16,77 | 17,04 | 9 | 20,92 |
| 908 | sp|P16219|ACADS_HUMAN | HUMAN | Short-chain specific acyl-CoA dehydrogenase, mitochondrial OS=Homo sapiens GN=ACADS PE=1 SV=1 | 16,77 | 16,89 | 9 | 31,55 |
| 909 | sp|P20591|MX1_HUMAN | HUMAN | Interferon-induced GTP-binding protein Mx1 OS=Homo sapiens GN=MX1 PE=1 SV=4 | 16,73 | 16,95 | 9 | 18,43 |
| 910 | sp|P16422|EPCAM_HUMAN | HUMAN | Epithelial cell adhesion molecule OS=Homo sapiens GN=EPCAM PE=1 SV=2 | 16,71 | 16,84 | 9 | 36,31 |
| 911 | sp|P36952|SPB5_HUMAN | HUMAN | Serpin B5 OS=Homo sapiens GN=SERPINB5 PE=1 SV=2 | 16,69 | 17,44 | 10 | 36,27 |
| 912 | sp|Q15661|TRYB1_HUMAN | HUMAN | Tryptase alpha/beta-1 OS=Homo sapiens GN=TPSAB1 PE=1 SV=1 | 16,67 | 18,75 | 13 | 37,82 |
| 913 | sp|P00441|SODC_HUMAN | HUMAN | Superoxide dismutase [Cu-Zn] OS=Homo sapiens GN=SOD1 PE=1 SV=2 | 16,66 | 16,75 | 30 | 71,43 |
| 914 | sp|Q99829|CPNE1_HUMAN | HUMAN | Copine-1 OS=Homo sapiens GN=CPNE1 PE=1 SV=1 | 16,65 | 17,35 | 9 | 20,67 |
| 915 | sp|Q13596|SNX1_HUMAN | HUMAN | Sorting nexin-1 OS=Homo sapiens GN=SNX1 PE=1 SV=3 | 16,62 | 21,34 | 11 | 24,14 |
| 916 | sp|Q13492|PICAL_HUMAN | HUMAN | Phosphatidylinositol-binding clathrin assembly protein OS=Homo sapiens GN=PICALM PE=1 SV=2 | 16,61 | 16,86 | 9 | 18,4 |
| 917 | sp|Q15257|PTPA_HUMAN | HUMAN | Serine/threonine-protein phosphatase 2A activator OS=Homo sapiens GN=PPP2R4 PE=1 SV=3 | 16,6 | 16,7 | 10 | 37,43 |
| 918 | sp|P02765|FETUA_HUMAN | HUMAN | Alpha-2-HS-glycoprotein OS=Homo sapiens GN=AHSG PE=1 SV=1 | 16,59 | 16,64 | 23 | 57,22 |
| 919 | sp|Q9Y281|COF2_HUMAN | HUMAN | Cofilin-2 OS=Homo sapiens GN=CFL2 PE=1 SV=1 | 16,57 | 24,87 | 24 | 78,31 |
| 920 | sp|P08697|A2AP_HUMAN | HUMAN | Alpha-2-antiplasmin OS=Homo sapiens GN=SERPINF2 PE=1 SV=3 | 16,57 | 16,85 | 9 | 23,42 |
| 921 | sp|Q13247|SRSF6_HUMAN | HUMAN | Serine/arginine-rich splicing factor 6 OS=Homo sapiens GN=SRSF6 PE=1 SV=2 | 16,56 | 16,85 | 12 | 29,65 |
| 922 | sp|P53004|BIEA_HUMAN | HUMAN | Biliverdin reductase A OS=Homo sapiens GN=BLVRA PE=1 SV=2 | 16,49 | 16,69 | 10 | 35,47 |
| 923 | sp|Q96AG4|LRC59_HUMAN | HUMAN | Leucine-rich repeat-containing protein 59 OS=Homo sapiens GN=LRRC59 PE=1 SV=1 | 16,49 | 16,67 | 10 | 41,04 |
| 924 | sp|Q5T4S7|UBR4_HUMAN | HUMAN | E3 ubiquitin-protein ligase UBR4 OS=Homo sapiens GN=UBR4 PE=1 SV=1 | 16,48 | 18,28 | 10 | 2,354 |
| 925 | sp|P28482|MK01_HUMAN | HUMAN | Mitogen-activated protein kinase 1 OS=Homo sapiens GN=MAPK1 PE=1 SV=3 | 16,47 | 29,27 | 19 | 56,11 |
| 926 | sp|Q6PKG0|LARP1_HUMAN | HUMAN | La-related protein 1 OS=Homo sapiens GN=LARP1 PE=1 SV=2 | 16,47 | 17,54 | 10 | 14,6 |
| 927 | sp|Q9Y371|SHLB1_HUMAN | HUMAN | Endophilin-B1 OS=Homo sapiens GN=SH3GLB1 PE=1 SV=1 | 16,47 | 17,04 | 10 | 30,14 |
| 928 | sp|P02766|TTHY_HUMAN | HUMAN | Transthyretin OS=Homo sapiens GN=TTR PE=1 SV=1 | 16,44 | 16,59 | 19 | 69,39 |
| 929 | sp|O00499|BIN1_HUMAN | HUMAN | Myc box-dependent-interacting protein 1 OS=Homo sapiens GN=BIN1 PE=1 SV=1 | 16,41 | 16,59 | 12 | 26,81 |
| 930 | sp|P09417|DHPR_HUMAN | HUMAN | Dihydropteridine reductase OS=Homo sapiens GN=QDPR PE=1 SV=2 | 16,35 | 16,52 | 10 | 56,97 |
| 931 | sp|Q6IBS0|TWF2_HUMAN | HUMAN | Twinfilin-2 OS=Homo sapiens GN=TWF2 PE=1 SV=2 | 16,32 | 21,6 | 14 | 46,42 |
| 932 | sp|O75436|VP26A_HUMAN | HUMAN | Vacuolar protein sorting-associated protein 26A OS=Homo sapiens GN=VPS26A PE=1 SV=2 | 16,32 | 16,53 | 11 | 42,81 |
| 933 | sp|Q6P4A8|PLBL1_HUMAN | HUMAN | Phospholipase B-like 1 OS=Homo sapiens GN=PLBD1 PE=1 SV=2 | 16,32 | 16,52 | 10 | 20,98 |
| 934 | sp|P30520|PURA2_HUMAN | HUMAN | Adenylosuccinate synthetase isozyme 2 OS=Homo sapiens GN=ADSS PE=1 SV=3 | 16,31 | 16,48 | 12 | 28,73 |
| 935 | sp|Q16630|CPSF6_HUMAN | HUMAN | Cleavage and polyadenylation specificity factor subunit 6 OS=Homo sapiens GN=CPSF6 PE=1 SV=2 | 16,31 | 16,46 | 9 | 20,33 |
| 936 | sp|P09467|F16P1_HUMAN | HUMAN | Fructose-1,6-bisphosphatase 1 OS=Homo sapiens GN=FBP1 PE=1 SV=5 | 16,31 | 16,34 | 10 | 36,69 |
| 937 | sp|P63208|SKP1_HUMAN | HUMAN | S-phase kinase-associated protein 1 OS=Homo sapiens GN=SKP1 PE=1 SV=2 | 16,29 | 16,42 | 17 | 66,87 |
| 938 | sp|P56537|IF6_HUMAN | HUMAN | Eukaryotic translation initiation factor 6 OS=Homo sapiens GN=EIF6 PE=1 SV=1 | 16,29 | 16,38 | 15 | 64,08 |
| 939 | sp|P33241|LSP1_HUMAN | HUMAN | Lymphocyte-specific protein 1 OS=Homo sapiens GN=LSP1 PE=1 SV=1 | 16,28 | 16,51 | 17 | 44,25 |
| 940 | sp|P31930|QCR1_HUMAN | HUMAN | Cytochrome b-c1 complex subunit 1, mitochondrial OS=Homo sapiens GN=UQCRC1 PE=1 SV=3 | 16,25 | 16,36 | 11 | 30,21 |
| 941 | sp|Q02543|RL18A_HUMAN | HUMAN | 60S ribosomal protein L18a OS=Homo sapiens GN=RPL18A PE=1 SV=2 | 16,24 | 16,39 | 8 | 45,45 |
| 942 | sp|P59998|ARPC4_HUMAN | HUMAN | Actin-related protein 2/3 complex subunit 4 OS=Homo sapiens GN=ARPC4 PE=1 SV=3 | 16,24 | 16,33 | 10 | 60,12 |
| 943 | sp|Q9Y2Q3|GSTK1_HUMAN | HUMAN | Glutathione S-transferase kappa 1 OS=Homo sapiens GN=GSTK1 PE=1 SV=3 | 16,22 | 16,33 | 12 | 59,73 |
| 944 | sp|P62241|RS8_HUMAN | HUMAN | 40S ribosomal protein S8 OS=Homo sapiens GN=RPS8 PE=1 SV=2 | 16,19 | 16,26 | 16 | 44,71 |
| 945 | sp|Q8NE71|ABCF1_HUMAN | HUMAN | ATP-binding cassette sub-family F member 1 OS=Homo sapiens GN=ABCF1 PE=1 SV=2 | 16,18 | 16,35 | 9 | 13,02 |
| 946 | sp|P48739|PIPNB_HUMAN | HUMAN | Phosphatidylinositol transfer protein beta isoform OS=Homo sapiens GN=PITPNB PE=1 SV=2 | 16,16 | 16,38 | 10 | 43,91 |
| 947 | sp|P12955|PEPD_HUMAN | HUMAN | Xaa-Pro dipeptidase OS=Homo sapiens GN=PEPD PE=1 SV=3 | 16,14 | 16,42 | 11 | 24,95 |
| 948 | sp|P46063|RECQ1_HUMAN | HUMAN | ATP-dependent DNA helicase Q1 OS=Homo sapiens GN=RECQL PE=1 SV=3 | 16,12 | 16,39 | 9 | 17,72 |
| 949 | sp|P61626|LYSC_HUMAN | HUMAN | Lysozyme C OS=Homo sapiens GN=LYZ PE=1 SV=1 | 16,12 | 16,36 | 14 | 65,54 |
| 950 | sp|O43809|CPSF5_HUMAN | HUMAN | Cleavage and polyadenylation specificity factor subunit 5 OS=Homo sapiens GN=NUDT21 PE=1 SV=1 | 16,11 | 16,29 | 11 | 41,41 |
| 951 | sp|Q92882|OSTF1_HUMAN | HUMAN | Osteoclast-stimulating factor 1 OS=Homo sapiens GN=OSTF1 PE=1 SV=2 | 16,1 | 16,15 | 9 | 53,27 |
| 952 | sp|P11047|LAMC1_HUMAN | HUMAN | Laminin subunit gamma-1 OS=Homo sapiens GN=LAMC1 PE=1 SV=3 | 16,03 | 16,49 | 10 | 10,57 |
| 953 | sp|Q14978|NOLC1_HUMAN | HUMAN | Nucleolar and coiled-body phosphoprotein 1 OS=Homo sapiens GN=NOLC1 PE=1 SV=2 | 16,03 | 16,36 | 9 | 16,17 |
| 954 | sp|P54920|SNAA_HUMAN | HUMAN | Alpha-soluble NSF attachment protein OS=Homo sapiens GN=NAPA PE=1 SV=3 | 16,01 | 16,01 | 8 | 35,25 |
| 955 | sp|O43294|TGFI1_HUMAN | HUMAN | Transforming growth factor beta-1-induced transcript 1 protein OS=Homo sapiens GN=TGFB1I1 PE=1 SV=2 | 15,99 | 16,06 | 8 | 28,2 |
| 956 | sp|Q13619|CUL4A_HUMAN | HUMAN | Cullin-4A OS=Homo sapiens GN=CUL4A PE=1 SV=3 | 15,97 | 16,37 | 8 | 12,65 |
| 957 | sp|P09661|RU2A_HUMAN | HUMAN | U2 small nuclear ribonucleoprotein A' OS=Homo sapiens GN=SNRPA1 PE=1 SV=2 | 15,96 | 16,8 | 9 | 34,9 |
| 958 | sp|P20160|CAP7_HUMAN | HUMAN | Azurocidin OS=Homo sapiens GN=AZU1 PE=1 SV=3 | 15,94 | 16,08 | 16 | 65,74 |
| 959 | sp|P55265|DSRAD_HUMAN | HUMAN | Double-stranded RNA-specific adenosine deaminase OS=Homo sapiens GN=ADAR PE=1 SV=4 | 15,93 | 16,32 | 11 | 11,75 |
| 960 | sp|Q9UIJ7|KAD3_HUMAN | HUMAN | GTP:AMP phosphotransferase AK3, mitochondrial OS=Homo sapiens GN=AK3 PE=1 SV=4 | 15,92 | 16,04 | 8 | 44,93 |
| 961 | sp|P01019|ANGT_HUMAN | HUMAN | Angiotensinogen OS=Homo sapiens GN=AGT PE=1 SV=1 | 15,9 | 15,93 | 9 | 20,41 |
| 962 | sp|P25787|PSA2_HUMAN | HUMAN | Proteasome subunit alpha type-2 OS=Homo sapiens GN=PSMA2 PE=1 SV=2 | 15,88 | 16,49 | 11 | 46,15 |
| 963 | sp|Q16666|IF16_HUMAN | HUMAN | Gamma-interferon-inducible protein 16 OS=Homo sapiens GN=IFI16 PE=1 SV=3 | 15,87 | 16,15 | 10 | 15,29 |
| 964 | sp|P08246|ELNE_HUMAN | HUMAN | Neutrophil elastase OS=Homo sapiens GN=ELANE PE=1 SV=1 | 15,84 | 15,88 | 16 | 49,06 |
| 965 | sp|Q8WU90|ZC3HF_HUMAN | HUMAN | Zinc finger CCCH domain-containing protein 15 OS=Homo sapiens GN=ZC3H15 PE=1 SV=1 | 15,81 | 15,95 | 8 | 23,94 |
| 966 | sp|P53597|SUCA_HUMAN | HUMAN | Succinyl-CoA ligase [ADP/GDP-forming] subunit alpha, mitochondrial OS=Homo sapiens GN=SUCLG1 PE=1 SV=4 | 15,81 | 15,93 | 14 | 35,26 |
| 967 | sp|Q9UNH7|SNX6_HUMAN | HUMAN | Sorting nexin-6 OS=Homo sapiens GN=SNX6 PE=1 SV=1 | 15,79 | 16,09 | 9 | 22,91 |
| 968 | sp|Q16204|CCDC6_HUMAN | HUMAN | Coiled-coil domain-containing protein 6 OS=Homo sapiens GN=CCDC6 PE=1 SV=2 | 15,78 | 16,02 | 14 | 21,52 |
| 969 | sp|Q96A49|SYAP1_HUMAN | HUMAN | Synapse-associated protein 1 OS=Homo sapiens GN=SYAP1 PE=1 SV=1 | 15,77 | 16,01 | 8 | 40,34 |
| 970 | sp|Q07666|KHDR1_HUMAN | HUMAN | KH domain-containing, RNA-binding, signal transduction-associated protein 1 OS=Homo sapiens GN=KHDRBS1 PE=1 SV=1 | 15,77 | 15,97 | 10 | 32,05 |
| 971 | sp|P12830|CADH1_HUMAN | HUMAN | Cadherin-1 OS=Homo sapiens GN=CDH1 PE=1 SV=3 | 15,71 | 15,99 | 12 | 19,05 |
| 972 | sp|P35637|FUS_HUMAN | HUMAN | RNA-binding protein FUS OS=Homo sapiens GN=FUS PE=1 SV=1 | 15,71 | 15,77 | 9 | 21,86 |
| 973 | sp|Q9Y2J8|PADI2_HUMAN | HUMAN | Protein-arginine deiminase type-2 OS=Homo sapiens GN=PADI2 PE=1 SV=2 | 15,7 | 15,9 | 10 | 19,85 |
| 974 | sp|Q86UE4|LYRIC_HUMAN | HUMAN | Protein LYRIC OS=Homo sapiens GN=MTDH PE=1 SV=2 | 15,69 | 15,81 | 9 | 20,1 |
| 975 | sp|Q7L014|DDX46_HUMAN | HUMAN | Probable ATP-dependent RNA helicase DDX46 OS=Homo sapiens GN=DDX46 PE=1 SV=2 | 15,66 | 17,12 | 10 | 11,25 |
| 976 | sp|O75534|CSDE1_HUMAN | HUMAN | Cold shock domain-containing protein E1 OS=Homo sapiens GN=CSDE1 PE=1 SV=2 | 15,65 | 15,99 | 10 | 13,03 |
| 977 | sp|P46108|CRK_HUMAN | HUMAN | Adapter molecule crk OS=Homo sapiens GN=CRK PE=1 SV=2 | 15,64 | 16,76 | 11 | 48,03 |
| 978 | sp|P09871|C1S_HUMAN | HUMAN | Complement C1s subcomponent OS=Homo sapiens GN=C1S PE=1 SV=1 | 15,64 | 15,85 | 10 | 17,44 |
| 979 | sp|O00629|IMA3_HUMAN | HUMAN | Importin subunit alpha-3 OS=Homo sapiens GN=KPNA4 PE=1 SV=1 | 15,64 | 15,79 | 8 | 30,33 |
| 980 | sp|P06702|S10A9_HUMAN | HUMAN | Protein S100-A9 OS=Homo sapiens GN=S100A9 PE=1 SV=1 | 15,61 | 15,75 | 50 | 86,84 |
| 981 | sp|Q9BQL6|FERM1_HUMAN | HUMAN | Fermitin family homolog 1 OS=Homo sapiens GN=FERMT1 PE=1 SV=1 | 15,56 | 21,94 | 14 | 22,16 |
| 982 | sp|P20290|BTF3_HUMAN | HUMAN | Transcription factor BTF3 OS=Homo sapiens GN=BTF3 PE=1 SV=1 | 15,56 | 15,69 | 14 | 57,77 |
| 983 | sp|Q92734|TFG_HUMAN | HUMAN | Protein TFG OS=Homo sapiens GN=TFG PE=1 SV=2 | 15,55 | 16,21 | 20 | 34,75 |
| 984 | sp|Q9Y285|SYFA_HUMAN | HUMAN | Phenylalanine--tRNA ligase alpha subunit OS=Homo sapiens GN=FARSA PE=1 SV=3 | 15,52 | 15,65 | 9 | 22,24 |
| 985 | sp|O00584|RNT2_HUMAN | HUMAN | Ribonuclease T2 OS=Homo sapiens GN=RNASET2 PE=1 SV=2 | 15,52 | 15,59 | 12 | 42,97 |
| 986 | sp|O43488|ARK72_HUMAN | HUMAN | Aflatoxin B1 aldehyde reductase member 2 OS=Homo sapiens GN=AKR7A2 PE=1 SV=3 | 15,49 | 17,88 | 11 | 35,93 |
| 987 | sp|Q9UKY7|CDV3_HUMAN | HUMAN | Protein CDV3 homolog OS=Homo sapiens GN=CDV3 PE=1 SV=1 | 15,47 | 15,68 | 11 | 63,18 |
| 988 | sp|Q9H0W9|CK054_HUMAN | HUMAN | Ester hydrolase C11orf54 OS=Homo sapiens GN=C11orf54 PE=1 SV=1 | 15,42 | 15,58 | 14 | 42,86 |
| 989 | sp|Q96IJ6|GMPPA_HUMAN | HUMAN | Mannose-1-phosphate guanyltransferase alpha OS=Homo sapiens GN=GMPPA PE=1 SV=1 | 15,41 | 15,52 | 8 | 27,38 |
| 990 | sp|P53999|TCP4_HUMAN | HUMAN | Activated RNA polymerase II transcriptional coactivator p15 OS=Homo sapiens GN=SUB1 PE=1 SV=3 | 15,4 | 15,9 | 11 | 74,02 |
| 991 | sp|Q16698|DECR_HUMAN | HUMAN | 2,4-dienoyl-CoA reductase, mitochondrial OS=Homo sapiens GN=DECR1 PE=1 SV=1 | 15,36 | 15,61 | 8 | 31,94 |
| 992 | sp|P05556|ITB1_HUMAN | HUMAN | Integrin beta-1 OS=Homo sapiens GN=ITGB1 PE=1 SV=2 | 15,34 | 15,64 | 10 | 15,16 |
| 993 | sp|P00326|ADH1G_HUMAN | HUMAN | Alcohol dehydrogenase 1C OS=Homo sapiens GN=ADH1C PE=1 SV=2 | 15,32 | 37,33 | 35 | 64 |
| 994 | sp|Q15185|TEBP_HUMAN | HUMAN | Prostaglandin E synthase 3 OS=Homo sapiens GN=PTGES3 PE=1 SV=1 | 15,31 | 15,43 | 18 | 51,25 |
| 995 | sp|Q03154|ACY1_HUMAN | HUMAN | Aminoacylase-1 OS=Homo sapiens GN=ACY1 PE=1 SV=1 | 15,3 | 15,9 | 8 | 27,21 |
| 996 | sp|P51452|DUS3_HUMAN | HUMAN | Dual specificity protein phosphatase 3 OS=Homo sapiens GN=DUSP3 PE=1 SV=1 | 15,27 | 15,34 | 9 | 55,14 |
| 997 | sp|Q9BSH5|HDHD3_HUMAN | HUMAN | Haloacid dehalogenase-like hydrolase domain-containing protein 3 OS=Homo sapiens GN=HDHD3 PE=1 SV=1 | 15,27 | 15,32 | 8 | 50,6 |
| 998 | sp|Q27J81|INF2_HUMAN | HUMAN | Inverted formin-2 OS=Homo sapiens GN=INF2 PE=1 SV=2 | 15,26 | 15,57 | 10 | 9,848 |
| 999 | sp|O75223|GGCT_HUMAN | HUMAN | Gamma-glutamylcyclotransferase OS=Homo sapiens GN=GGCT PE=1 SV=1 | 15,26 | 15,45 | 10 | 55,32 |
| 1000 | sp|P49773|HINT1_HUMAN | HUMAN | Histidine triad nucleotide-binding protein 1 OS=Homo sapiens GN=HINT1 PE=1 SV=2 | 15,24 | 15,3 | 15 | 75,4 |
| 1001 | sp|P55735|SEC13_HUMAN | HUMAN | Protein SEC13 homolog OS=Homo sapiens GN=SEC13 PE=1 SV=3 | 15,21 | 15,31 | 13 | 40,68 |
| 1002 | sp|P28070|PSB4_HUMAN | HUMAN | Proteasome subunit beta type-4 OS=Homo sapiens GN=PSMB4 PE=1 SV=4 | 15,2 | 15,23 | 14 | 48,48 |
| 1003 | sp|Q9UMR2|DD19B_HUMAN | HUMAN | ATP-dependent RNA helicase DDX19B OS=Homo sapiens GN=DDX19B PE=1 SV=1 | 15,17 | 15,54 | 14 | 30,06 |
| 1004 | sp|P05452|TETN_HUMAN | HUMAN | Tetranectin OS=Homo sapiens GN=CLEC3B PE=1 SV=3 | 15,12 | 15,16 | 8 | 51,98 |
| 1005 | sp|P49721|PSB2_HUMAN | HUMAN | Proteasome subunit beta type-2 OS=Homo sapiens GN=PSMB2 PE=1 SV=1 | 15,1 | 15,83 | 10 | 44,78 |
| 1006 | sp|Q7Z434|MAVS_HUMAN | HUMAN | Mitochondrial antiviral-signaling protein OS=Homo sapiens GN=MAVS PE=1 SV=2 | 15,08 | 15,19 | 8 | 25,74 |
| 1007 | sp|P55196|AFAD_HUMAN | HUMAN | Afadin OS=Homo sapiens GN=MLLT4 PE=1 SV=3 | 15,06 | 15,66 | 8 | 6,634 |
| 1008 | sp|O00233|PSMD9_HUMAN | HUMAN | 26S proteasome non-ATPase regulatory subunit 9 OS=Homo sapiens GN=PSMD9 PE=1 SV=3 | 15,05 | 15,2 | 8 | 35,87 |
| 1009 | sp|Q8TAQ2|SMRC2_HUMAN | HUMAN | SWI/SNF complex subunit SMARCC2 OS=Homo sapiens GN=SMARCC2 PE=1 SV=1 | 15,03 | 15,28 | 8 | 8,155 |
| 1010 | sp|P33316|DUT_HUMAN | HUMAN | Deoxyuridine 5'-triphosphate nucleotidohydrolase, mitochondrial OS=Homo sapiens GN=DUT PE=1 SV=4 | 15,03 | 15,1 | 9 | 38,89 |
| 1011 | sp|Q9Y3L3|3BP1_HUMAN | HUMAN | SH3 domain-binding protein 1 OS=Homo sapiens GN=SH3BP1 PE=1 SV=3 | 14,97 | 15,17 | 9 | 16,55 |
| 1012 | sp|P62244|RS15A_HUMAN | HUMAN | 40S ribosomal protein S15a OS=Homo sapiens GN=RPS15A PE=1 SV=2 | 14,96 | 15,06 | 11 | 61,54 |
| 1013 | sp|P61254|RL26_HUMAN | HUMAN | 60S ribosomal protein L26 OS=Homo sapiens GN=RPL26 PE=1 SV=1 | 14,93 | 15,07 | 11 | 48,28 |
| 1014 | sp|O14617|AP3D1_HUMAN | HUMAN | AP-3 complex subunit delta-1 OS=Homo sapiens GN=AP3D1 PE=1 SV=1 | 14,92 | 15,17 | 9 | 12,92 |
| 1015 | sp|P45880|VDAC2_HUMAN | HUMAN | Voltage-dependent anion-selective channel protein 2 OS=Homo sapiens GN=VDAC2 PE=1 SV=2 | 14,87 | 15,92 | 9 | 38,44 |
| 1016 | sp|Q8WXF1|PSPC1_HUMAN | HUMAN | Paraspeckle component 1 OS=Homo sapiens GN=PSPC1 PE=1 SV=1 | 14,85 | 16,5 | 10 | 18,16 |
| 1017 | sp|P62879|GBB2_HUMAN | HUMAN | Guanine nucleotide-binding protein G(I)/G(S)/G(T) subunit beta-2 OS=Homo sapiens GN=GNB2 PE=1 SV=3 | 14,83 | 14,92 | 8 | 35,29 |
| 1018 | sp|P19801|AOC1_HUMAN | HUMAN | Amiloride-sensitive amine oxidase [copper-containing] OS=Homo sapiens GN=AOC1 PE=1 SV=4 | 14,8 | 15,18 | 9 | 12,92 |
| 1019 | sp|Q9H0D6|XRN2_HUMAN | HUMAN | 5'-3' exoribonuclease 2 OS=Homo sapiens GN=XRN2 PE=1 SV=1 | 14,8 | 15,15 | 10 | 14,53 |
| 1020 | sp|Q9UHL4|DPP2_HUMAN | HUMAN | Dipeptidyl peptidase 2 OS=Homo sapiens GN=DPP7 PE=1 SV=3 | 14,78 | 14,94 | 9 | 21,54 |
| 1021 | sp|O15145|ARPC3_HUMAN | HUMAN | Actin-related protein 2/3 complex subunit 3 OS=Homo sapiens GN=ARPC3 PE=1 SV=3 | 14,78 | 14,89 | 11 | 58,99 |
| 1022 | sp|Q15274|NADC_HUMAN | HUMAN | Nicotinate-nucleotide pyrophosphorylase [carboxylating] OS=Homo sapiens GN=QPRT PE=1 SV=3 | 14,78 | 14,87 | 9 | 38,05 |
| 1023 | sp|Q9H6Z4|RANB3_HUMAN | HUMAN | Ran-binding protein 3 OS=Homo sapiens GN=RANBP3 PE=1 SV=1 | 14,77 | 15,42 | 9 | 20,11 |
| 1024 | sp|P02461|CO3A1_HUMAN | HUMAN | Collagen alpha-1(III) chain OS=Homo sapiens GN=COL3A1 PE=1 SV=4 | 14,75 | 14,97 | 24 | 17,67 |
| 1025 | sp|O75822|EIF3J_HUMAN | HUMAN | Eukaryotic translation initiation factor 3 subunit J OS=Homo sapiens GN=EIF3J PE=1 SV=2 | 14,73 | 14,95 | 11 | 38,76 |
| 1026 | sp|P18621|RL17_HUMAN | HUMAN | 60S ribosomal protein L17 OS=Homo sapiens GN=RPL17 PE=1 SV=3 | 14,73 | 14,87 | 9 | 50 |
| 1027 | sp|Q13510|ASAH1_HUMAN | HUMAN | Acid ceramidase OS=Homo sapiens GN=ASAH1 PE=1 SV=5 | 14,73 | 14,8 | 10 | 30,13 |
| 1028 | sp|Q16363|LAMA4_HUMAN | HUMAN | Laminin subunit alpha-4 OS=Homo sapiens GN=LAMA4 PE=1 SV=4 | 14,72 | 15,2 | 11 | 8,557 |
| 1029 | sp|Q03135|CAV1_HUMAN | HUMAN | Caveolin-1 OS=Homo sapiens GN=CAV1 PE=1 SV=4 | 14,71 | 15,06 | 9 | 57,3 |
| 1030 | sp|Q13057|COASY_HUMAN | HUMAN | Bifunctional coenzyme A synthase OS=Homo sapiens GN=COASY PE=1 SV=4 | 14,69 | 15,27 | 9 | 26,06 |
| 1031 | sp|Q9NXG2|THUM1_HUMAN | HUMAN | THUMP domain-containing protein 1 OS=Homo sapiens GN=THUMPD1 PE=1 SV=2 | 14,69 | 15,04 | 9 | 30,03 |
| 1032 | sp|P41567|EIF1_HUMAN | HUMAN | Eukaryotic translation initiation factor 1 OS=Homo sapiens GN=EIF1 PE=1 SV=1 | 14,69 | 14,74 | 14 | 76,11 |
| 1033 | sp|Q96IU4|ABHEB_HUMAN | HUMAN | Protein ABHD14B OS=Homo sapiens GN=ABHD14B PE=1 SV=1 | 14,66 | 16,3 | 15 | 75,24 |
| 1034 | sp|O75340|PDCD6_HUMAN | HUMAN | Programmed cell death protein 6 OS=Homo sapiens GN=PDCD6 PE=1 SV=1 | 14,66 | 14,72 | 13 | 58,12 |
| 1035 | sp|P99999|CYC_HUMAN | HUMAN | Cytochrome c OS=Homo sapiens GN=CYCS PE=1 SV=2 | 14,65 | 14,69 | 16 | 57,14 |
| 1036 | sp|P39748|FEN1_HUMAN | HUMAN | Flap endonuclease 1 OS=Homo sapiens GN=FEN1 PE=1 SV=1 | 14,64 | 15,81 | 9 | 31,05 |
| 1037 | sp|P04080|CYTB_HUMAN | HUMAN | Cystatin-B OS=Homo sapiens GN=CSTB PE=1 SV=2 | 14,63 | 14,85 | 30 | 85,71 |
| 1038 | sp|O14979|HNRDL_HUMAN | HUMAN | Heterogeneous nuclear ribonucleoprotein D-like OS=Homo sapiens GN=HNRNPDL PE=1 SV=3 | 14,62 | 18,86 | 14 | 25,48 |
| 1039 | sp|P04066|FUCO_HUMAN | HUMAN | Tissue alpha-L-fucosidase OS=Homo sapiens GN=FUCA1 PE=1 SV=4 | 14,6 | 14,72 | 9 | 22,32 |
| 1040 | sp|Q9NYL9|TMOD3_HUMAN | HUMAN | Tropomodulin-3 OS=Homo sapiens GN=TMOD3 PE=1 SV=1 | 14,56 | 14,73 | 8 | 28,69 |
| 1041 | sp|P61353|RL27_HUMAN | HUMAN | 60S ribosomal protein L27 OS=Homo sapiens GN=RPL27 PE=1 SV=2 | 14,51 | 14,54 | 7 | 52,21 |
| 1042 | sp|P25398|RS12_HUMAN | HUMAN | 40S ribosomal protein S12 OS=Homo sapiens GN=RPS12 PE=1 SV=3 | 14,49 | 14,52 | 13 | 71,21 |
| 1043 | sp|O94973|AP2A2_HUMAN | HUMAN | AP-2 complex subunit alpha-2 OS=Homo sapiens GN=AP2A2 PE=1 SV=2 | 14,45 | 31,87 | 17 | 26,84 |
| 1044 | sp|O60832|DKC1_HUMAN | HUMAN | H/ACA ribonucleoprotein complex subunit 4 OS=Homo sapiens GN=DKC1 PE=1 SV=3 | 14,45 | 14,62 | 9 | 21,4 |
| 1045 | sp|Q9Y365|PCTL_HUMAN | HUMAN | PCTP-like protein OS=Homo sapiens GN=STARD10 PE=1 SV=2 | 14,44 | 14,72 | 7 | 32,3 |
| 1046 | sp|Q14696|MESD_HUMAN | HUMAN | LDLR chaperone MESD OS=Homo sapiens GN=MESDC2 PE=1 SV=2 | 14,44 | 14,67 | 10 | 45,73 |
| 1047 | sp|Q16186|ADRM1_HUMAN | HUMAN | Proteasomal ubiquitin receptor ADRM1 OS=Homo sapiens GN=ADRM1 PE=1 SV=2 | 14,43 | 14,56 | 8 | 20,39 |
| 1048 | sp|Q9H3P7|GCP60_HUMAN | HUMAN | Golgi resident protein GCP60 OS=Homo sapiens GN=ACBD3 PE=1 SV=4 | 14,41 | 14,66 | 8 | 22,54 |
| 1049 | sp|P62888|RL30_HUMAN | HUMAN | 60S ribosomal protein L30 OS=Homo sapiens GN=RPL30 PE=1 SV=2 | 14,4 | 14,52 | 13 | 76,52 |
| 1050 | sp|Q93008|USP9X_HUMAN | HUMAN | Probable ubiquitin carboxyl-terminal hydrolase FAF-X OS=Homo sapiens GN=USP9X PE=1 SV=3 | 14,38 | 14,77 | 10 | 4,086 |
| 1051 | sp|O75937|DNJC8_HUMAN | HUMAN | DnaJ homolog subfamily C member 8 OS=Homo sapiens GN=DNAJC8 PE=1 SV=2 | 14,38 | 14,57 | 9 | 38,74 |
| 1052 | sp|P02743|SAMP_HUMAN | HUMAN | Serum amyloid P-component OS=Homo sapiens GN=APCS PE=1 SV=2 | 14,33 | 14,4 | 10 | 36,77 |
| 1053 | sp|P31937|3HIDH_HUMAN | HUMAN | 3-hydroxyisobutyrate dehydrogenase, mitochondrial OS=Homo sapiens GN=HIBADH PE=1 SV=2 | 14,32 | 14,34 | 8 | 33,04 |
| 1054 | sp|P14780|MMP9_HUMAN | HUMAN | Matrix metalloproteinase-9 OS=Homo sapiens GN=MMP9 PE=1 SV=3 | 14,31 | 14,54 | 9 | 13,15 |
| 1055 | sp|Q9H993|ARMT1_HUMAN | HUMAN | Protein-glutamate O-methyltransferase OS=Homo sapiens GN=ARMT1 PE=1 SV=1 | 14,3 | 14,47 | 9 | 25,17 |
| 1056 | sp|P52788|SPSY_HUMAN | HUMAN | Spermine synthase OS=Homo sapiens GN=SMS PE=1 SV=2 | 14,29 | 14,46 | 8 | 31,42 |
| 1057 | sp|Q29RF7|PDS5A_HUMAN | HUMAN | Sister chromatid cohesion protein PDS5 homolog A OS=Homo sapiens GN=PDS5A PE=1 SV=1 | 14,27 | 14,39 | 8 | 8,003 |
| 1058 | sp|O43681|ASNA_HUMAN | HUMAN | ATPase ASNA1 OS=Homo sapiens GN=ASNA1 PE=1 SV=2 | 14,26 | 14,48 | 8 | 31,03 |
| 1059 | sp|P68036|UB2L3_HUMAN | HUMAN | Ubiquitin-conjugating enzyme E2 L3 OS=Homo sapiens GN=UBE2L3 PE=1 SV=1 | 14,25 | 14,39 | 14 | 68,18 |
| 1060 | sp|Q9NZT2|OGFR_HUMAN | HUMAN | Opioid growth factor receptor OS=Homo sapiens GN=OGFR PE=1 SV=3 | 14,24 | 14,45 | 9 | 29,39 |
| 1061 | sp|Q15366|PCBP2_HUMAN | HUMAN | Poly(rC)-binding protein 2 OS=Homo sapiens GN=PCBP2 PE=1 SV=1 | 14,19 | 23,09 | 20 | 46,3 |
| 1062 | sp|Q9NQG5|RPR1B_HUMAN | HUMAN | Regulation of nuclear pre-mRNA domain-containing protein 1B OS=Homo sapiens GN=RPRD1B PE=1 SV=1 | 14,19 | 14,46 | 9 | 37,42 |
| 1063 | sp|P61086|UBE2K_HUMAN | HUMAN | Ubiquitin-conjugating enzyme E2 K OS=Homo sapiens GN=UBE2K PE=1 SV=3 | 14,18 | 15,45 | 9 | 60 |
| 1064 | sp|O75396|SC22B_HUMAN | HUMAN | Vesicle-trafficking protein SEC22b OS=Homo sapiens GN=SEC22B PE=1 SV=4 | 14,18 | 14,25 | 9 | 42,33 |
| 1065 | sp|P62750|RL23A_HUMAN | HUMAN | 60S ribosomal protein L23a OS=Homo sapiens GN=RPL23A PE=1 SV=1 | 14,17 | 14,27 | 9 | 33,33 |
| 1066 | sp|Q9Y5Z4|HEBP2_HUMAN | HUMAN | Heme-binding protein 2 OS=Homo sapiens GN=HEBP2 PE=1 SV=1 | 14,17 | 14,23 | 11 | 48,78 |
| 1067 | sp|Q14839|CHD4_HUMAN | HUMAN | Chromodomain-helicase-DNA-binding protein 4 OS=Homo sapiens GN=CHD4 PE=1 SV=2 | 14,16 | 14,75 | 8 | 4,812 |
| 1068 | sp|Q9UK76|HN1_HUMAN | HUMAN | Hematological and neurological expressed 1 protein OS=Homo sapiens GN=HN1 PE=1 SV=3 | 14,15 | 14,16 | 9 | 74,03 |
| 1069 | sp|P35442|TSP2_HUMAN | HUMAN | Thrombospondin-2 OS=Homo sapiens GN=THBS2 PE=1 SV=2 | 14,14 | 16,69 | 9 | 11,35 |
| 1070 | sp|Q9Y6Q5|AP1M2_HUMAN | HUMAN | AP-1 complex subunit mu-2 OS=Homo sapiens GN=AP1M2 PE=1 SV=4 | 14,14 | 14,5 | 9 | 24,11 |
| 1071 | sp|P62316|SMD2_HUMAN | HUMAN | Small nuclear ribonucleoprotein Sm D2 OS=Homo sapiens GN=SNRPD2 PE=1 SV=1 | 14,11 | 14,19 | 12 | 61,02 |
| 1072 | sp|P18135|KV312_HUMAN | HUMAN | Ig kappa chain V-III region HAH OS=Homo sapiens PE=2 SV=1 | 14,1 | 14,26 | 15 | 62,02 |
| 1073 | sp|Q9NQC3|RTN4_HUMAN | HUMAN | Reticulon-4 OS=Homo sapiens GN=RTN4 PE=1 SV=2 | 14,09 | 14,3 | 16 | 11,33 |
| 1074 | sp|Q13098|CSN1_HUMAN | HUMAN | COP9 signalosome complex subunit 1 OS=Homo sapiens GN=GPS1 PE=1 SV=4 | 14,08 | 14,18 | 8 | 21,38 |
| 1075 | sp|Q92747|ARC1A_HUMAN | HUMAN | Actin-related protein 2/3 complex subunit 1A OS=Homo sapiens GN=ARPC1A PE=1 SV=2 | 14,03 | 16,75 | 11 | 35,68 |
| 1076 | sp|Q12874|SF3A3_HUMAN | HUMAN | Splicing factor 3A subunit 3 OS=Homo sapiens GN=SF3A3 PE=1 SV=1 | 14,03 | 14,19 | 8 | 19,56 |
| 1077 | sp|P30046|DOPD_HUMAN | HUMAN | D-dopachrome decarboxylase OS=Homo sapiens GN=DDT PE=1 SV=3 | 14,03 | 14,14 | 15 | 94,92 |
| 1078 | sp|P14735|IDE_HUMAN | HUMAN | Insulin-degrading enzyme OS=Homo sapiens GN=IDE PE=1 SV=4 | 13,98 | 14,26 | 9 | 11,38 |
| 1079 | sp|P08123|CO1A2_HUMAN | HUMAN | Collagen alpha-2(I) chain OS=Homo sapiens GN=COL1A2 PE=1 SV=7 | 13,92 | 14,18 | 18 | 16,4 |
| 1080 | sp|P10909|CLUS_HUMAN | HUMAN | Clusterin OS=Homo sapiens GN=CLU PE=1 SV=1 | 13,88 | 14,62 | 12 | 25,84 |
| 1081 | sp|O95810|SDPR_HUMAN | HUMAN | Serum deprivation-response protein OS=Homo sapiens GN=SDPR PE=1 SV=3 | 13,88 | 13,96 | 9 | 24,24 |
| 1082 | sp|Q53FA7|QORX_HUMAN | HUMAN | Quinone oxidoreductase PIG3 OS=Homo sapiens GN=TP53I3 PE=1 SV=2 | 13,86 | 14,13 | 8 | 31,33 |
| 1083 | sp|P62753|RS6_HUMAN | HUMAN | 40S ribosomal protein S6 OS=Homo sapiens GN=RPS6 PE=1 SV=1 | 13,86 | 13,97 | 10 | 28,92 |
| 1084 | sp|P80303|NUCB2_HUMAN | HUMAN | Nucleobindin-2 OS=Homo sapiens GN=NUCB2 PE=1 SV=2 | 13,83 | 14 | 11 | 27,62 |
| 1085 | sp|Q9UL25|RAB21_HUMAN | HUMAN | Ras-related protein Rab-21 OS=Homo sapiens GN=RAB21 PE=1 SV=3 | 13,82 | 13,97 | 9 | 44 |
| 1086 | sp|P28062|PSB8_HUMAN | HUMAN | Proteasome subunit beta type-8 OS=Homo sapiens GN=PSMB8 PE=1 SV=3 | 13,77 | 14,06 | 14 | 52,17 |
| 1087 | sp|Q9H1E3|NUCKS_HUMAN | HUMAN | Nuclear ubiquitous casein and cyclin-dependent kinase substrate 1 OS=Homo sapiens GN=NUCKS1 PE=1 SV=1 | 13,76 | 13,88 | 10 | 21,4 |
| 1088 | sp|Q8WVM8|SCFD1_HUMAN | HUMAN | Sec1 family domain-containing protein 1 OS=Homo sapiens GN=SCFD1 PE=1 SV=4 | 13,74 | 13,8 | 7 | 17,91 |
| 1089 | sp|P21796|VDAC1_HUMAN | HUMAN | Voltage-dependent anion-selective channel protein 1 OS=Homo sapiens GN=VDAC1 PE=1 SV=2 | 13,73 | 13,79 | 7 | 30,04 |
| 1090 | sp|P25774|CATS_HUMAN | HUMAN | Cathepsin S OS=Homo sapiens GN=CTSS PE=1 SV=3 | 13,72 | 13,77 | 9 | 31,72 |
| 1091 | sp|P62330|ARF6_HUMAN | HUMAN | ADP-ribosylation factor 6 OS=Homo sapiens GN=ARF6 PE=1 SV=2 | 13,71 | 15,19 | 10 | 71,43 |
| 1092 | sp|P84098|RL19_HUMAN | HUMAN | 60S ribosomal protein L19 OS=Homo sapiens GN=RPL19 PE=1 SV=1 | 13,7 | 14,14 | 9 | 31,12 |
| 1093 | sp|P62277|RS13_HUMAN | HUMAN | 40S ribosomal protein S13 OS=Homo sapiens GN=RPS13 PE=1 SV=2 | 13,66 | 13,79 | 13 | 49,67 |
| 1094 | sp|P09488|GSTM1_HUMAN | HUMAN | Glutathione S-transferase Mu 1 OS=Homo sapiens GN=GSTM1 PE=1 SV=3 | 13,64 | 14,68 | 8 | 36,7 |
| 1095 | sp|Q9Y2X3|NOP58_HUMAN | HUMAN | Nucleolar protein 58 OS=Homo sapiens GN=NOP58 PE=1 SV=1 | 13,63 | 13,82 | 8 | 19,47 |
| 1096 | sp|P62081|RS7_HUMAN | HUMAN | 40S ribosomal protein S7 OS=Homo sapiens GN=RPS7 PE=1 SV=1 | 13,63 | 13,71 | 15 | 52,06 |
| 1097 | sp|P09455|RET1_HUMAN | HUMAN | Retinol-binding protein 1 OS=Homo sapiens GN=RBP1 PE=1 SV=2 | 13,59 | 13,7 | 7 | 59,26 |
| 1098 | sp|Q99426|TBCB_HUMAN | HUMAN | Tubulin-folding cofactor B OS=Homo sapiens GN=TBCB PE=1 SV=2 | 13,57 | 13,66 | 9 | 40,16 |
| 1099 | sp|O15067|PUR4_HUMAN | HUMAN | Phosphoribosylformylglycinamidine synthase OS=Homo sapiens GN=PFAS PE=1 SV=4 | 13,48 | 13,68 | 8 | 9,118 |
| 1100 | sp|O43765|SGTA_HUMAN | HUMAN | Small glutamine-rich tetratricopeptide repeat-containing protein alpha OS=Homo sapiens GN=SGTA PE=1 SV=1 | 13,4 | 13,57 | 8 | 29,71 |
| 1101 | sp|P30050|RL12_HUMAN | HUMAN | 60S ribosomal protein L12 OS=Homo sapiens GN=RPL12 PE=1 SV=1 | 13,39 | 13,5 | 14 | 59,39 |
| 1102 | sp|Q8WZA9|IRGQ_HUMAN | HUMAN | Immunity-related GTPase family Q protein OS=Homo sapiens GN=IRGQ PE=1 SV=1 | 13,37 | 13,56 | 11 | 26,48 |
| 1103 | sp|O94855|SC24D_HUMAN | HUMAN | Protein transport protein Sec24D OS=Homo sapiens GN=SEC24D PE=1 SV=2 | 13,36 | 13,68 | 8 | 12,89 |
| 1104 | sp|P13727|PRG2_HUMAN | HUMAN | Bone marrow proteoglycan OS=Homo sapiens GN=PRG2 PE=1 SV=2 | 13,36 | 13,44 | 8 | 38,29 |
| 1105 | sp|Q96P70|IPO9_HUMAN | HUMAN | Importin-9 OS=Homo sapiens GN=IPO9 PE=1 SV=3 | 13,35 | 13,71 | 8 | 9,51 |
| 1106 | sp|P46779|RL28_HUMAN | HUMAN | 60S ribosomal protein L28 OS=Homo sapiens GN=RPL28 PE=1 SV=3 | 13,34 | 13,6 | 12 | 51,09 |
| 1107 | sp|P30419|NMT1_HUMAN | HUMAN | Glycylpeptide N-tetradecanoyltransferase 1 OS=Homo sapiens GN=NMT1 PE=1 SV=2 | 13,33 | 13,46 | 8 | 21,57 |
| 1108 | sp|P49720|PSB3_HUMAN | HUMAN | Proteasome subunit beta type-3 OS=Homo sapiens GN=PSMB3 PE=1 SV=2 | 13,31 | 13,39 | 12 | 48,29 |
| 1109 | sp|P24666|PPAC_HUMAN | HUMAN | Low molecular weight phosphotyrosine protein phosphatase OS=Homo sapiens GN=ACP1 PE=1 SV=3 | 13,27 | 13,31 | 10 | 61,39 |
| 1110 | sp|P62913|RL11_HUMAN | HUMAN | 60S ribosomal protein L11 OS=Homo sapiens GN=RPL11 PE=1 SV=2 | 13,26 | 13,43 | 11 | 40,45 |
| 1111 | sp|P55263|ADK_HUMAN | HUMAN | Adenosine kinase OS=Homo sapiens GN=ADK PE=1 SV=2 | 13,21 | 13,52 | 7 | 23,2 |
| 1112 | sp|Q8NCW5|NNRE_HUMAN | HUMAN | NAD(P)H-hydrate epimerase OS=Homo sapiens GN=APOA1BP PE=1 SV=2 | 13,21 | 13,31 | 10 | 39,58 |
| 1113 | sp|P02042|HBD_HUMAN | HUMAN | Hemoglobin subunit delta OS=Homo sapiens GN=HBD PE=1 SV=2 | 13,2 | 43,39 | 179 | 99,32 |
| 1114 | sp|P11310|ACADM_HUMAN | HUMAN | Medium-chain specific acyl-CoA dehydrogenase, mitochondrial OS=Homo sapiens GN=ACADM PE=1 SV=1 | 13,16 | 13,55 | 8 | 23,04 |
| 1115 | sp|P49354|FNTA_HUMAN | HUMAN | Protein farnesyltransferase/geranylgeranyltransferase type-1 subunit alpha OS=Homo sapiens GN=FNTA PE=1 SV=1 | 13,14 | 13,23 | 7 | 26,65 |
| 1116 | sp|Q86TX2|ACOT1_HUMAN | HUMAN | Acyl-coenzyme A thioesterase 1 OS=Homo sapiens GN=ACOT1 PE=1 SV=1 | 13,12 | 13,28 | 8 | 24,94 |
| 1117 | sp|Q9UFN0|NPS3A_HUMAN | HUMAN | Protein NipSnap homolog 3A OS=Homo sapiens GN=NIPSNAP3A PE=1 SV=2 | 13,11 | 13,21 | 8 | 39,68 |
| 1118 | sp|O95394|AGM1_HUMAN | HUMAN | Phosphoacetylglucosamine mutase OS=Homo sapiens GN=PGM3 PE=1 SV=1 | 13,09 | 13,22 | 6 | 12,55 |
| 1119 | sp|P68400|CSK21_HUMAN | HUMAN | Casein kinase II subunit alpha OS=Homo sapiens GN=CSNK2A1 PE=1 SV=1 | 13,09 | 13,22 | 7 | 25,32 |
| 1120 | sp|Q13557|KCC2D_HUMAN | HUMAN | Calcium/calmodulin-dependent protein kinase type II subunit delta OS=Homo sapiens GN=CAMK2D PE=1 SV=3 | 13,08 | 13,89 | 9 | 20,44 |
| 1121 | sp|O00154|BACH_HUMAN | HUMAN | Cytosolic acyl coenzyme A thioester hydrolase OS=Homo sapiens GN=ACOT7 PE=1 SV=3 | 13,05 | 13,15 | 7 | 26,58 |
| 1122 | sp|Q07020|RL18_HUMAN | HUMAN | 60S ribosomal protein L18 OS=Homo sapiens GN=RPL18 PE=1 SV=2 | 13,04 | 13,09 | 8 | 31,38 |
| 1123 | sp|Q9Y263|PLAP_HUMAN | HUMAN | Phospholipase A-2-activating protein OS=Homo sapiens GN=PLAA PE=1 SV=2 | 13,03 | 13,43 | 9 | 12,96 |
| 1124 | sp|Q7Z4I7|LIMS2_HUMAN | HUMAN | LIM and senescent cell antigen-like-containing domain protein 2 OS=Homo sapiens GN=LIMS2 PE=1 SV=1 | 13,03 | 13,26 | 8 | 24,63 |
| 1125 | sp|O60869|EDF1_HUMAN | HUMAN | Endothelial differentiation-related factor 1 OS=Homo sapiens GN=EDF1 PE=1 SV=1 | 13,03 | 13,16 | 7 | 43,24 |
| 1126 | sp|Q8NBF2|NHLC2_HUMAN | HUMAN | NHL repeat-containing protein 2 OS=Homo sapiens GN=NHLRC2 PE=1 SV=1 | 13 | 13,16 | 7 | 16,8 |
| 1127 | sp|Q9UMY4|SNX12_HUMAN | HUMAN | Sorting nexin-12 OS=Homo sapiens GN=SNX12 PE=1 SV=3 | 12,99 | 13,06 | 10 | 53,49 |
| 1128 | sp|Q9UBR2|CATZ_HUMAN | HUMAN | Cathepsin Z OS=Homo sapiens GN=CTSZ PE=1 SV=1 | 12,98 | 13,61 | 8 | 23,76 |
| 1129 | sp|P31942|HNRH3_HUMAN | HUMAN | Heterogeneous nuclear ribonucleoprotein H3 OS=Homo sapiens GN=HNRNPH3 PE=1 SV=2 | 12,97 | 13,17 | 11 | 23,41 |
| 1130 | sp|O43324|MCA3_HUMAN | HUMAN | Eukaryotic translation elongation factor 1 epsilon-1 OS=Homo sapiens GN=EEF1E1 PE=1 SV=1 | 12,93 | 12,98 | 10 | 50 |
| 1131 | sp|O15511|ARPC5_HUMAN | HUMAN | Actin-related protein 2/3 complex subunit 5 OS=Homo sapiens GN=ARPC5 PE=1 SV=3 | 12,91 | 12,98 | 9 | 66,23 |
| 1132 | sp|Q9NQT8|KI13B_HUMAN | HUMAN | Kinesin-like protein KIF13B OS=Homo sapiens GN=KIF13B PE=1 SV=2 | 12,9 | 14,06 | 7 | 4,71 |
| 1133 | sp|O00273|DFFA_HUMAN | HUMAN | DNA fragmentation factor subunit alpha OS=Homo sapiens GN=DFFA PE=1 SV=1 | 12,9 | 13,09 | 9 | 32,63 |
| 1134 | sp|P05107|ITB2_HUMAN | HUMAN | Integrin beta-2 OS=Homo sapiens GN=ITGB2 PE=1 SV=2 | 12,88 | 13,02 | 7 | 9,623 |
| 1135 | sp|Q15102|PA1B3_HUMAN | HUMAN | Platelet-activating factor acetylhydrolase IB subunit gamma OS=Homo sapiens GN=PAFAH1B3 PE=1 SV=1 | 12,88 | 12,94 | 9 | 39,83 |
| 1136 | sp|O60844|ZG16_HUMAN | HUMAN | Zymogen granule membrane protein 16 OS=Homo sapiens GN=ZG16 PE=1 SV=2 | 12,88 | 12,91 | 20 | 70,06 |
| 1137 | sp|Q5SSJ5|HP1B3_HUMAN | HUMAN | Heterochromatin protein 1-binding protein 3 OS=Homo sapiens GN=HP1BP3 PE=1 SV=1 | 12,87 | 13 | 8 | 16,82 |
| 1138 | sp|P14324|FPPS_HUMAN | HUMAN | Farnesyl pyrophosphate synthase OS=Homo sapiens GN=FDPS PE=1 SV=4 | 12,87 | 12,91 | 11 | 28,16 |
| 1139 | sp|P22307|NLTP_HUMAN | HUMAN | Non-specific lipid-transfer protein OS=Homo sapiens GN=SCP2 PE=1 SV=2 | 12,84 | 12,99 | 8 | 11,15 |
| 1140 | sp|P30048|PRDX3_HUMAN | HUMAN | Thioredoxin-dependent peroxide reductase, mitochondrial OS=Homo sapiens GN=PRDX3 PE=1 SV=3 | 12,83 | 13,11 | 16 | 40,23 |
| 1141 | sp|Q3YEC7|RABL6_HUMAN | HUMAN | Rab-like protein 6 OS=Homo sapiens GN=RABL6 PE=1 SV=2 | 12,8 | 12,97 | 8 | 11,39 |
| 1142 | sp|P33992|MCM5_HUMAN | HUMAN | DNA replication licensing factor MCM5 OS=Homo sapiens GN=MCM5 PE=1 SV=5 | 12,77 | 13,66 | 10 | 16,49 |
| 1143 | sp|P40261|NNMT_HUMAN | HUMAN | Nicotinamide N-methyltransferase OS=Homo sapiens GN=NNMT PE=1 SV=1 | 12,77 | 13,01 | 7 | 32,95 |
| 1144 | sp|P29218|IMPA1_HUMAN | HUMAN | Inositol monophosphatase 1 OS=Homo sapiens GN=IMPA1 PE=1 SV=1 | 12,77 | 12,84 | 8 | 33,21 |
| 1145 | sp|P82979|SARNP_HUMAN | HUMAN | SAP domain-containing ribonucleoprotein OS=Homo sapiens GN=SARNP PE=1 SV=3 | 12,76 | 12,83 | 7 | 35,71 |
| 1146 | sp|Q13155|AIMP2_HUMAN | HUMAN | Aminoacyl tRNA synthase complex-interacting multifunctional protein 2 OS=Homo sapiens GN=AIMP2 PE=1 SV=2 | 12,75 | 12,83 | 6 | 30 |
| 1147 | sp|P63279|UBC9_HUMAN | HUMAN | SUMO-conjugating enzyme UBC9 OS=Homo sapiens GN=UBE2I PE=1 SV=1 | 12,74 | 12,89 | 8 | 54,43 |
| 1148 | sp|Q13547|HDAC1_HUMAN | HUMAN | Histone deacetylase 1 OS=Homo sapiens GN=HDAC1 PE=1 SV=1 | 12,73 | 13,62 | 7 | 17,43 |
| 1149 | sp|P61201|CSN2_HUMAN | HUMAN | COP9 signalosome complex subunit 2 OS=Homo sapiens GN=COPS2 PE=1 SV=1 | 12,73 | 12,86 | 7 | 22,8 |
| 1150 | sp|P07951|TPM2_HUMAN | HUMAN | Tropomyosin beta chain OS=Homo sapiens GN=TPM2 PE=1 SV=1 | 12,7 | 51,36 | 69 | 60,56 |
| 1151 | sp|Q08209|PP2BA_HUMAN | HUMAN | Serine/threonine-protein phosphatase 2B catalytic subunit alpha isoform OS=Homo sapiens GN=PPP3CA PE=1 SV=1 | 12,68 | 15,26 | 10 | 18,23 |
| 1152 | sp|Q9NZL9|MAT2B_HUMAN | HUMAN | Methionine adenosyltransferase 2 subunit beta OS=Homo sapiens GN=MAT2B PE=1 SV=1 | 12,68 | 12,8 | 7 | 26,05 |
| 1153 | sp|P62851|RS25_HUMAN | HUMAN | 40S ribosomal protein S25 OS=Homo sapiens GN=RPS25 PE=1 SV=1 | 12,66 | 12,72 | 12 | 39,2 |
| 1154 | sp|Q2TAA2|IAH1_HUMAN | HUMAN | Isoamyl acetate-hydrolyzing esterase 1 homolog OS=Homo sapiens GN=IAH1 PE=1 SV=1 | 12,66 | 12,69 | 7 | 42,34 |
| 1155 | sp|P62633|CNBP_HUMAN | HUMAN | Cellular nucleic acid-binding protein OS=Homo sapiens GN=CNBP PE=1 SV=1 | 12,63 | 12,71 | 8 | 43,5 |
| 1156 | sp|Q9Y2L1|RRP44_HUMAN | HUMAN | Exosome complex exonuclease RRP44 OS=Homo sapiens GN=DIS3 PE=1 SV=2 | 12,61 | 12,76 | 7 | 9,708 |
| 1157 | sp|Q9Y295|DRG1_HUMAN | HUMAN | Developmentally-regulated GTP-binding protein 1 OS=Homo sapiens GN=DRG1 PE=1 SV=1 | 12,59 | 13,01 | 7 | 22,07 |
| 1158 | sp|P51570|GALK1_HUMAN | HUMAN | Galactokinase OS=Homo sapiens GN=GALK1 PE=1 SV=1 | 12,53 | 12,83 | 9 | 23,21 |
| 1159 | sp|Q12805|FBLN3_HUMAN | HUMAN | EGF-containing fibulin-like extracellular matrix protein 1 OS=Homo sapiens GN=EFEMP1 PE=1 SV=2 | 12,52 | 12,69 | 7 | 19,07 |
| 1160 | sp|P35659|DEK_HUMAN | HUMAN | Protein DEK OS=Homo sapiens GN=DEK PE=1 SV=1 | 12,5 | 12,57 | 8 | 26,4 |
| 1161 | sp|P54725|RD23A_HUMAN | HUMAN | UV excision repair protein RAD23 homolog A OS=Homo sapiens GN=RAD23A PE=1 SV=1 | 12,49 | 18,04 | 11 | 46,01 |
| 1162 | sp|P33993|MCM7_HUMAN | HUMAN | DNA replication licensing factor MCM7 OS=Homo sapiens GN=MCM7 PE=1 SV=4 | 12,48 | 12,82 | 6 | 12,52 |
| 1163 | sp|Q8TBC4|UBA3_HUMAN | HUMAN | NEDD8-activating enzyme E1 catalytic subunit OS=Homo sapiens GN=UBA3 PE=1 SV=2 | 12,48 | 12,69 | 8 | 29,59 |
| 1164 | sp|Q13404|UB2V1_HUMAN | HUMAN | Ubiquitin-conjugating enzyme E2 variant 1 OS=Homo sapiens GN=UBE2V1 PE=1 SV=2 | 12,44 | 12,59 | 11 | 54,42 |
| 1165 | sp|O60784|TOM1_HUMAN | HUMAN | Target of Myb protein 1 OS=Homo sapiens GN=TOM1 PE=1 SV=2 | 12,43 | 12,9 | 7 | 19,31 |
| 1166 | sp|Q9Y6D6|BIG1_HUMAN | HUMAN | Brefeldin A-inhibited guanine nucleotide-exchange protein 1 OS=Homo sapiens GN=ARFGEF1 PE=1 SV=2 | 12,43 | 12,65 | 6 | 3,678 |
| 1167 | sp|Q9NSK0|KLC4_HUMAN | HUMAN | Kinesin light chain 4 OS=Homo sapiens GN=KLC4 PE=1 SV=3 | 12,39 | 22,24 | 12 | 22,94 |
| 1168 | sp|P29590|PML_HUMAN | HUMAN | Protein PML OS=Homo sapiens GN=PML PE=1 SV=3 | 12,37 | 12,6 | 9 | 11,11 |
| 1169 | sp|P36543|VATE1_HUMAN | HUMAN | V-type proton ATPase subunit E 1 OS=Homo sapiens GN=ATP6V1E1 PE=1 SV=1 | 12,36 | 12,54 | 7 | 27,88 |
| 1170 | sp|Q29974|2B1G_HUMAN | HUMAN | HLA class II histocompatibility antigen, DRB1-16 beta chain OS=Homo sapiens GN=HLA-DRB1 PE=1 SV=1 | 12,36 | 12,46 | 7 | 36,47 |
| 1171 | sp|Q9Y5X3|SNX5_HUMAN | HUMAN | Sorting nexin-5 OS=Homo sapiens GN=SNX5 PE=1 SV=1 | 12,33 | 12,75 | 8 | 23,51 |
| 1172 | sp|O60271|JIP4_HUMAN | HUMAN | C-Jun-amino-terminal kinase-interacting protein 4 OS=Homo sapiens GN=SPAG9 PE=1 SV=4 | 12,33 | 12,52 | 8 | 8,478 |
| 1173 | sp|P52815|RM12_HUMAN | HUMAN | 39S ribosomal protein L12, mitochondrial OS=Homo sapiens GN=MRPL12 PE=1 SV=2 | 12,31 | 12,41 | 7 | 46,97 |
| 1174 | sp|Q9Y5L0|TNPO3_HUMAN | HUMAN | Transportin-3 OS=Homo sapiens GN=TNPO3 PE=1 SV=3 | 12,3 | 12,47 | 9 | 9,426 |
| 1175 | sp|P18077|RL35A_HUMAN | HUMAN | 60S ribosomal protein L35a OS=Homo sapiens GN=RPL35A PE=1 SV=2 | 12,3 | 12,43 | 10 | 59,09 |
| 1176 | sp|O76021|RL1D1_HUMAN | HUMAN | Ribosomal L1 domain-containing protein 1 OS=Homo sapiens GN=RSL1D1 PE=1 SV=3 | 12,28 | 12,38 | 6 | 17,14 |
| 1177 | sp|P13693|TCTP_HUMAN | HUMAN | Translationally-controlled tumor protein OS=Homo sapiens GN=TPT1 PE=1 SV=1 | 12,27 | 12,45 | 23 | 53,49 |
| 1178 | sp|Q00577|PURA_HUMAN | HUMAN | Transcriptional activator protein Pur-alpha OS=Homo sapiens GN=PURA PE=1 SV=2 | 12,27 | 12,42 | 11 | 36,02 |
| 1179 | sp|O95747|OXSR1_HUMAN | HUMAN | Serine/threonine-protein kinase OSR1 OS=Homo sapiens GN=OXSR1 PE=1 SV=1 | 12,26 | 14,25 | 10 | 20,49 |
| 1180 | sp|Q8NFW8|NEUA_HUMAN | HUMAN | N-acylneuraminate cytidylyltransferase OS=Homo sapiens GN=CMAS PE=1 SV=2 | 12,24 | 12,42 | 9 | 27,42 |
| 1181 | sp|Q99623|PHB2_HUMAN | HUMAN | Prohibitin-2 OS=Homo sapiens GN=PHB2 PE=1 SV=2 | 12,24 | 12,37 | 7 | 23,08 |
| 1182 | sp|Q9NR28|DBLOH_HUMAN | HUMAN | Diablo homolog, mitochondrial OS=Homo sapiens GN=DIABLO PE=1 SV=1 | 12,24 | 12,31 | 7 | 32,64 |
| 1183 | sp|Q9NTX5|ECHD1_HUMAN | HUMAN | Ethylmalonyl-CoA decarboxylase OS=Homo sapiens GN=ECHDC1 PE=1 SV=2 | 12,21 | 12,35 | 7 | 27,04 |
| 1184 | sp|P06703|S10A6_HUMAN | HUMAN | Protein S100-A6 OS=Homo sapiens GN=S100A6 PE=1 SV=1 | 12,21 | 12,32 | 18 | 66,67 |
| 1185 | sp|Q9H074|PAIP1_HUMAN | HUMAN | Polyadenylate-binding protein-interacting protein 1 OS=Homo sapiens GN=PAIP1 PE=1 SV=1 | 12,19 | 12,43 | 8 | 20,25 |
| 1186 | sp|Q07654|TFF3_HUMAN | HUMAN | Trefoil factor 3 OS=Homo sapiens GN=TFF3 PE=1 SV=1 | 12,16 | 12,31 | 16 | 67,5 |
| 1187 | sp|P48556|PSMD8_HUMAN | HUMAN | 26S proteasome non-ATPase regulatory subunit 8 OS=Homo sapiens GN=PSMD8 PE=1 SV=2 | 12,15 | 12,39 | 7 | 20,86 |
| 1188 | sp|P84103|SRSF3_HUMAN | HUMAN | Serine/arginine-rich splicing factor 3 OS=Homo sapiens GN=SRSF3 PE=1 SV=1 | 12,15 | 12,37 | 11 | 35,37 |
| 1189 | sp|P63173|RL38_HUMAN | HUMAN | 60S ribosomal protein L38 OS=Homo sapiens GN=RPL38 PE=1 SV=2 | 12,15 | 12,28 | 7 | 50 |
| 1190 | sp|Q9UHY7|ENOPH_HUMAN | HUMAN | Enolase-phosphatase E1 OS=Homo sapiens GN=ENOPH1 PE=1 SV=1 | 12,15 | 12,16 | 6 | 37,93 |
| 1191 | sp|P04264|K2C1_HUMAN | HUMAN | Keratin, type II cytoskeletal 1 OS=Homo sapiens GN=KRT1 PE=1 SV=6 | 12,12 | 22,65 | 16 | 16,3 |
| 1192 | sp|P09496|CLCA_HUMAN | HUMAN | Clathrin light chain A OS=Homo sapiens GN=CLTA PE=1 SV=1 | 12,1 | 12,37 | 10 | 31,05 |
| 1193 | sp|O00148|DX39A_HUMAN | HUMAN | ATP-dependent RNA helicase DDX39A OS=Homo sapiens GN=DDX39A PE=1 SV=2 | 12,04 | 34,84 | 22 | 52,69 |
| 1194 | sp|O94776|MTA2_HUMAN | HUMAN | Metastasis-associated protein MTA2 OS=Homo sapiens GN=MTA2 PE=1 SV=1 | 12,03 | 12,32 | 9 | 12,13 |
| 1195 | sp|P08571|CD14_HUMAN | HUMAN | Monocyte differentiation antigen CD14 OS=Homo sapiens GN=CD14 PE=1 SV=2 | 12,03 | 12,18 | 8 | 32,27 |
| 1196 | sp|P52943|CRIP2_HUMAN | HUMAN | Cysteine-rich protein 2 OS=Homo sapiens GN=CRIP2 PE=1 SV=1 | 12,03 | 12,12 | 9 | 51,44 |
| 1197 | sp|Q53T59|H1BP3_HUMAN | HUMAN | HCLS1-binding protein 3 OS=Homo sapiens GN=HS1BP3 PE=1 SV=1 | 12,03 | 12,12 | 7 | 26,79 |
| 1198 | sp|Q7Z5L9|I2BP2_HUMAN | HUMAN | Interferon regulatory factor 2-binding protein 2 OS=Homo sapiens GN=IRF2BP2 PE=1 SV=2 | 12,02 | 12,15 | 7 | 20,78 |
| 1199 | sp|P68366|TBA4A_HUMAN | HUMAN | Tubulin alpha-4A chain OS=Homo sapiens GN=TUBA4A PE=1 SV=1 | 12 | 56,88 | 77 | 79,69 |
| 1200 | sp|Q9Y617|SERC_HUMAN | HUMAN | Phosphoserine aminotransferase OS=Homo sapiens GN=PSAT1 PE=1 SV=2 | 11,99 | 12,8 | 10 | 31,89 |
| 1201 | sp|P56545|CTBP2_HUMAN | HUMAN | C-terminal-binding protein 2 OS=Homo sapiens GN=CTBP2 PE=1 SV=1 | 11,99 | 12,26 | 8 | 19,78 |
| 1202 | sp|Q99879|H2B1M_HUMAN | HUMAN | Histone H2B type 1-M OS=Homo sapiens GN=HIST1H2BM PE=1 SV=3 | 11,98 | 12,14 | 14 | 46,83 |
| 1203 | sp|P19525|E2AK2_HUMAN | HUMAN | Interferon-induced, double-stranded RNA-activated protein kinase OS=Homo sapiens GN=EIF2AK2 PE=1 SV=2 | 11,97 | 12,16 | 6 | 11,62 |
| 1204 | sp|O94903|PROSC_HUMAN | HUMAN | Proline synthase co-transcribed bacterial homolog protein OS=Homo sapiens GN=PROSC PE=1 SV=1 | 11,96 | 12,2 | 8 | 28,36 |
| 1205 | sp|P16070|CD44_HUMAN | HUMAN | CD44 antigen OS=Homo sapiens GN=CD44 PE=1 SV=3 | 11,93 | 12,11 | 10 | 11,86 |
| 1206 | sp|P19652|A1AG2_HUMAN | HUMAN | Alpha-1-acid glycoprotein 2 OS=Homo sapiens GN=ORM2 PE=1 SV=2 | 11,89 | 19,83 | 16 | 48,76 |
| 1207 | sp|O95372|LYPA2_HUMAN | HUMAN | Acyl-protein thioesterase 2 OS=Homo sapiens GN=LYPLA2 PE=1 SV=1 | 11,88 | 12,1 | 7 | 45,45 |
| 1208 | sp|O75351|VPS4B_HUMAN | HUMAN | Vacuolar protein sorting-associated protein 4B OS=Homo sapiens GN=VPS4B PE=1 SV=2 | 11,83 | 12,1 | 8 | 22,07 |
| 1209 | sp|O60884|DNJA2_HUMAN | HUMAN | DnaJ homolog subfamily A member 2 OS=Homo sapiens GN=DNAJA2 PE=1 SV=1 | 11,81 | 11,97 | 7 | 28,64 |
| 1210 | sp|Q9NTZ6|RBM12_HUMAN | HUMAN | RNA-binding protein 12 OS=Homo sapiens GN=RBM12 PE=1 SV=1 | 11,8 | 11,96 | 7 | 7,511 |
| 1211 | sp|P16930|FAAA_HUMAN | HUMAN | Fumarylacetoacetase OS=Homo sapiens GN=FAH PE=1 SV=2 | 11,79 | 11,9 | 6 | 20,53 |
| 1212 | sp|Q6NVY1|HIBCH_HUMAN | HUMAN | 3-hydroxyisobutyryl-CoA hydrolase, mitochondrial OS=Homo sapiens GN=HIBCH PE=1 SV=2 | 11,77 | 12,62 | 8 | 22,54 |
| 1213 | sp|Q99439|CNN2_HUMAN | HUMAN | Calponin-2 OS=Homo sapiens GN=CNN2 PE=1 SV=4 | 11,76 | 16,17 | 11 | 29,45 |
| 1214 | sp|Q9BY32|ITPA_HUMAN | HUMAN | Inosine triphosphate pyrophosphatase OS=Homo sapiens GN=ITPA PE=1 SV=2 | 11,74 | 11,96 | 7 | 47,42 |
| 1215 | sp|Q01581|HMCS1_HUMAN | HUMAN | Hydroxymethylglutaryl-CoA synthase, cytoplasmic OS=Homo sapiens GN=HMGCS1 PE=1 SV=2 | 11,7 | 13,94 | 9 | 18,65 |
| 1216 | sp|P62805|H4_HUMAN | HUMAN | Histone H4 OS=Homo sapiens GN=HIST1H4A PE=1 SV=2 | 11,7 | 11,77 | 14 | 53,4 |
| 1217 | sp|Q8TCD5|NT5C_HUMAN | HUMAN | 5'(3')-deoxyribonucleotidase, cytosolic type OS=Homo sapiens GN=NT5C PE=1 SV=2 | 11,69 | 11,76 | 7 | 54,23 |
| 1218 | sp|Q9NRV9|HEBP1_HUMAN | HUMAN | Heme-binding protein 1 OS=Homo sapiens GN=HEBP1 PE=1 SV=1 | 11,67 | 11,79 | 6 | 41,8 |
| 1219 | sp|P42126|ECI1_HUMAN | HUMAN | Enoyl-CoA delta isomerase 1, mitochondrial OS=Homo sapiens GN=ECI1 PE=1 SV=1 | 11,63 | 11,98 | 6 | 26,82 |
| 1220 | sp|P33240|CSTF2_HUMAN | HUMAN | Cleavage stimulation factor subunit 2 OS=Homo sapiens GN=CSTF2 PE=1 SV=1 | 11,63 | 11,78 | 6 | 14,56 |
| 1221 | sp|P09936|UCHL1_HUMAN | HUMAN | Ubiquitin carboxyl-terminal hydrolase isozyme L1 OS=Homo sapiens GN=UCHL1 PE=1 SV=2 | 11,63 | 11,7 | 6 | 40,36 |
| 1222 | sp|P62841|RS15_HUMAN | HUMAN | 40S ribosomal protein S15 OS=Homo sapiens GN=RPS15 PE=1 SV=2 | 11,6 | 11,78 | 6 | 53,79 |
| 1223 | sp|P02730|B3AT_HUMAN | HUMAN | Band 3 anion transport protein OS=Homo sapiens GN=SLC4A1 PE=1 SV=3 | 11,6 | 11,73 | 8 | 13,28 |
| 1224 | sp|Q9UNS2|CSN3_HUMAN | HUMAN | COP9 signalosome complex subunit 3 OS=Homo sapiens GN=COPS3 PE=1 SV=3 | 11,58 | 11,74 | 7 | 24,35 |
| 1225 | sp|P31949|S10AB_HUMAN | HUMAN | Protein S100-A11 OS=Homo sapiens GN=S100A11 PE=1 SV=2 | 11,56 | 11,63 | 15 | 80 |
| 1226 | sp|Q969H8|MYDGF_HUMAN | HUMAN | Myeloid-derived growth factor OS=Homo sapiens GN=MYDGF PE=1 SV=1 | 11,55 | 11,65 | 8 | 43,93 |
| 1227 | sp|P05026|AT1B1_HUMAN | HUMAN | Sodium/potassium-transporting ATPase subunit beta-1 OS=Homo sapiens GN=ATP1B1 PE=1 SV=1 | 11,55 | 11,62 | 7 | 29,7 |
| 1228 | sp|P02652|APOA2_HUMAN | HUMAN | Apolipoprotein A-II OS=Homo sapiens GN=APOA2 PE=1 SV=1 | 11,55 | 11,59 | 14 | 64 |
| 1229 | sp|Q9HAB8|PPCS_HUMAN | HUMAN | Phosphopantothenate--cysteine ligase OS=Homo sapiens GN=PPCS PE=1 SV=2 | 11,54 | 11,67 | 6 | 18,65 |
| 1230 | sp|Q9H8Y8|GORS2_HUMAN | HUMAN | Golgi reassembly-stacking protein 2 OS=Homo sapiens GN=GORASP2 PE=1 SV=3 | 11,53 | 11,67 | 8 | 25 |
| 1231 | sp|Q9NSE4|SYIM_HUMAN | HUMAN | Isoleucine--tRNA ligase, mitochondrial OS=Homo sapiens GN=IARS2 PE=1 SV=2 | 11,53 | 11,61 | 6 | 7,806 |
| 1232 | sp|Q8IY67|RAVR1_HUMAN | HUMAN | Ribonucleoprotein PTB-binding 1 OS=Homo sapiens GN=RAVER1 PE=1 SV=1 | 11,53 | 11,58 | 6 | 21,62 |
| 1233 | sp|Q95604|1C17_HUMAN | HUMAN | HLA class I histocompatibility antigen, Cw-17 alpha chain OS=Homo sapiens GN=HLA-C PE=1 SV=1 | 11,52 | 17,89 | 10 | 30,11 |
| 1234 | sp|P61970|NTF2_HUMAN | HUMAN | Nuclear transport factor 2 OS=Homo sapiens GN=NUTF2 PE=1 SV=1 | 11,5 | 11,58 | 6 | 74,8 |
| 1235 | sp|P42566|EPS15_HUMAN | HUMAN | Epidermal growth factor receptor substrate 15 OS=Homo sapiens GN=EPS15 PE=1 SV=2 | 11,47 | 12,32 | 8 | 11,16 |
| 1236 | sp|O15212|PFD6_HUMAN | HUMAN | Prefoldin subunit 6 OS=Homo sapiens GN=PFDN6 PE=1 SV=1 | 11,47 | 11,64 | 8 | 44,19 |
| 1237 | sp|Q9BZK7|TBL1R_HUMAN | HUMAN | F-box-like/WD repeat-containing protein TBL1XR1 OS=Homo sapiens GN=TBL1XR1 PE=1 SV=1 | 11,47 | 11,62 | 8 | 21,79 |
| 1238 | sp|P21695|GPDA_HUMAN | HUMAN | Glycerol-3-phosphate dehydrogenase [NAD(+)], cytoplasmic OS=Homo sapiens GN=GPD1 PE=1 SV=4 | 11,46 | 11,74 | 6 | 20,92 |
| 1239 | sp|Q09161|NCBP1_HUMAN | HUMAN | Nuclear cap-binding protein subunit 1 OS=Homo sapiens GN=NCBP1 PE=1 SV=1 | 11,45 | 11,72 | 8 | 9,494 |
| 1240 | sp|Q05315|LEG10_HUMAN | HUMAN | Galectin-10 OS=Homo sapiens GN=CLC PE=1 SV=3 | 11,45 | 11,57 | 27 | 71,83 |
| 1241 | sp|Q14558|KPRA_HUMAN | HUMAN | Phosphoribosyl pyrophosphate synthase-associated protein 1 OS=Homo sapiens GN=PRPSAP1 PE=1 SV=2 | 11,43 | 11,58 | 7 | 30,06 |
| 1242 | sp|Q9BUL8|PDC10_HUMAN | HUMAN | Programmed cell death protein 10 OS=Homo sapiens GN=PDCD10 PE=1 SV=1 | 11,42 | 11,46 | 6 | 30,66 |
| 1243 | sp|Q9BY44|EIF2A_HUMAN | HUMAN | Eukaryotic translation initiation factor 2A OS=Homo sapiens GN=EIF2A PE=1 SV=3 | 11,41 | 11,67 | 7 | 17,09 |
| 1244 | sp|Q96D15|RCN3_HUMAN | HUMAN | Reticulocalbin-3 OS=Homo sapiens GN=RCN3 PE=1 SV=1 | 11,41 | 11,48 | 6 | 28,05 |
| 1245 | sp|P01903|DRA_HUMAN | HUMAN | HLA class II histocompatibility antigen, DR alpha chain OS=Homo sapiens GN=HLA-DRA PE=1 SV=1 | 11,4 | 11,46 | 7 | 32,28 |
| 1246 | sp|Q9UBB4|ATX10_HUMAN | HUMAN | Ataxin-10 OS=Homo sapiens GN=ATXN10 PE=1 SV=1 | 11,39 | 11,96 | 7 | 19,79 |
| 1247 | sp|Q9Y520|PRC2C_HUMAN | HUMAN | Protein PRRC2C OS=Homo sapiens GN=PRRC2C PE=1 SV=4 | 11,39 | 11,82 | 9 | 3,28 |
| 1248 | sp|Q5VW32|BROX_HUMAN | HUMAN | BRO1 domain-containing protein BROX OS=Homo sapiens GN=BROX PE=1 SV=1 | 11,37 | 11,55 | 8 | 23,6 |
| 1249 | sp|P41218|MNDA_HUMAN | HUMAN | Myeloid cell nuclear differentiation antigen OS=Homo sapiens GN=MNDA PE=1 SV=1 | 11,34 | 12,01 | 7 | 18,18 |
| 1250 | sp|P50914|RL14_HUMAN | HUMAN | 60S ribosomal protein L14 OS=Homo sapiens GN=RPL14 PE=1 SV=4 | 11,33 | 12,9 | 10 | 30,23 |
| 1251 | sp|P20774|MIME_HUMAN | HUMAN | Mimecan OS=Homo sapiens GN=OGN PE=1 SV=1 | 11,3 | 11,45 | 7 | 26,51 |
| 1252 | sp|P38159|RBMX_HUMAN | HUMAN | RNA-binding motif protein, X chromosome OS=Homo sapiens GN=RBMX PE=1 SV=3 | 11,29 | 11,57 | 9 | 16,37 |
| 1253 | sp|P68402|PA1B2_HUMAN | HUMAN | Platelet-activating factor acetylhydrolase IB subunit beta OS=Homo sapiens GN=PAFAH1B2 PE=1 SV=1 | 11,28 | 12,49 | 10 | 43,67 |
| 1254 | sp|P05166|PCCB_HUMAN | HUMAN | Propionyl-CoA carboxylase beta chain, mitochondrial OS=Homo sapiens GN=PCCB PE=1 SV=3 | 11,28 | 11,43 | 7 | 18,55 |
| 1255 | sp|P61026|RAB10_HUMAN | HUMAN | Ras-related protein Rab-10 OS=Homo sapiens GN=RAB10 PE=1 SV=1 | 11,26 | 15,96 | 11 | 39 |
| 1256 | sp|O15400|STX7_HUMAN | HUMAN | Syntaxin-7 OS=Homo sapiens GN=STX7 PE=1 SV=4 | 11,25 | 11,54 | 8 | 39,08 |
| 1257 | sp|Q9NVS9|PNPO_HUMAN | HUMAN | Pyridoxine-5'-phosphate oxidase OS=Homo sapiens GN=PNPO PE=1 SV=1 | 11,25 | 11,43 | 8 | 44,83 |
| 1258 | sp|O75608|LYPA1_HUMAN | HUMAN | Acyl-protein thioesterase 1 OS=Homo sapiens GN=LYPLA1 PE=1 SV=1 | 11,23 | 11,3 | 9 | 57,83 |
| 1259 | sp|Q8N6H7|ARFG2_HUMAN | HUMAN | ADP-ribosylation factor GTPase-activating protein 2 OS=Homo sapiens GN=ARFGAP2 PE=1 SV=1 | 11,21 | 11,38 | 6 | 13,63 |
| 1260 | sp|O43583|DENR_HUMAN | HUMAN | Density-regulated protein OS=Homo sapiens GN=DENR PE=1 SV=2 | 11,19 | 11,3 | 8 | 49,49 |
| 1261 | sp|P07108|ACBP_HUMAN | HUMAN | Acyl-CoA-binding protein OS=Homo sapiens GN=DBI PE=1 SV=2 | 11,17 | 11,72 | 12 | 72,41 |
| 1262 | sp|Q13148|TADBP_HUMAN | HUMAN | TAR DNA-binding protein 43 OS=Homo sapiens GN=TARDBP PE=1 SV=1 | 11,16 | 11,4 | 8 | 25,85 |
| 1263 | sp|Q9H444|CHM4B_HUMAN | HUMAN | Charged multivesicular body protein 4b OS=Homo sapiens GN=CHMP4B PE=1 SV=1 | 11,14 | 11,2 | 8 | 37,95 |
| 1264 | sp|P16444|DPEP1_HUMAN | HUMAN | Dipeptidase 1 OS=Homo sapiens GN=DPEP1 PE=1 SV=3 | 11,12 | 11,25 | 8 | 21,17 |
| 1265 | sp|P26447|S10A4_HUMAN | HUMAN | Protein S100-A4 OS=Homo sapiens GN=S100A4 PE=1 SV=1 | 11,12 | 11,25 | 10 | 51,49 |
| 1266 | sp|P31350|RIR2_HUMAN | HUMAN | Ribonucleoside-diphosphate reductase subunit M2 OS=Homo sapiens GN=RRM2 PE=1 SV=1 | 11,11 | 11,46 | 7 | 23,91 |
| 1267 | sp|Q9NR31|SAR1A_HUMAN | HUMAN | GTP-binding protein SAR1a OS=Homo sapiens GN=SAR1A PE=1 SV=1 | 11,1 | 11,23 | 14 | 48,99 |
| 1268 | sp|P58546|MTPN_HUMAN | HUMAN | Myotrophin OS=Homo sapiens GN=MTPN PE=1 SV=2 | 11,09 | 11,14 | 10 | 57,63 |
| 1269 | sp|O15372|EIF3H_HUMAN | HUMAN | Eukaryotic translation initiation factor 3 subunit H OS=Homo sapiens GN=EIF3H PE=1 SV=1 | 11,07 | 11,34 | 6 | 19,6 |
| 1270 | sp|Q99961|SH3G1_HUMAN | HUMAN | Endophilin-A2 OS=Homo sapiens GN=SH3GL1 PE=1 SV=1 | 11,06 | 11,32 | 10 | 23,64 |
| 1271 | sp|Q14240|IF4A2_HUMAN | HUMAN | Eukaryotic initiation factor 4A-II OS=Homo sapiens GN=EIF4A2 PE=1 SV=2 | 11,05 | 36,46 | 31 | 53,56 |
| 1272 | sp|Q8IVT2|MISP_HUMAN | HUMAN | Mitotic interactor and substrate of PLK1 OS=Homo sapiens GN=MISP PE=1 SV=1 | 11,05 | 11,23 | 6 | 14,29 |
| 1273 | sp|P67870|CSK2B_HUMAN | HUMAN | Casein kinase II subunit beta OS=Homo sapiens GN=CSNK2B PE=1 SV=1 | 11,02 | 11,13 | 6 | 41,86 |
| 1274 | sp|P43121|MUC18_HUMAN | HUMAN | Cell surface glycoprotein MUC18 OS=Homo sapiens GN=MCAM PE=1 SV=2 | 11 | 11,2 | 8 | 15,94 |
| 1275 | sp|P08621|RU17_HUMAN | HUMAN | U1 small nuclear ribonucleoprotein 70 kDa OS=Homo sapiens GN=SNRNP70 PE=1 SV=2 | 10,99 | 11,25 | 9 | 15,1 |
| 1276 | sp|P40306|PSB10_HUMAN | HUMAN | Proteasome subunit beta type-10 OS=Homo sapiens GN=PSMB10 PE=1 SV=1 | 10,99 | 11,1 | 10 | 28,21 |
| 1277 | sp|Q9UBF2|COPG2_HUMAN | HUMAN | Coatomer subunit gamma-2 OS=Homo sapiens GN=COPG2 PE=1 SV=1 | 10,94 | 18,76 | 10 | 14,24 |
| 1278 | sp|P21283|VATC1_HUMAN | HUMAN | V-type proton ATPase subunit C 1 OS=Homo sapiens GN=ATP6V1C1 PE=1 SV=4 | 10,94 | 11,16 | 6 | 18,85 |
| 1279 | sp|P30533|AMRP_HUMAN | HUMAN | Alpha-2-macroglobulin receptor-associated protein OS=Homo sapiens GN=LRPAP1 PE=1 SV=1 | 10,93 | 11,21 | 7 | 19,89 |
| 1280 | sp|O43768|ENSA_HUMAN | HUMAN | Alpha-endosulfine OS=Homo sapiens GN=ENSA PE=1 SV=1 | 10,93 | 11,04 | 7 | 60,33 |
| 1281 | sp|P61081|UBC12_HUMAN | HUMAN | NEDD8-conjugating enzyme Ubc12 OS=Homo sapiens GN=UBE2M PE=1 SV=1 | 10,92 | 11,04 | 6 | 34,97 |
| 1282 | sp|Q5JRX3|PREP_HUMAN | HUMAN | Presequence protease, mitochondrial OS=Homo sapiens GN=PITRM1 PE=1 SV=3 | 10,89 | 10,97 | 6 | 10,51 |
| 1283 | sp|Q07021|C1QBP_HUMAN | HUMAN | Complement component 1 Q subcomponent-binding protein, mitochondrial OS=Homo sapiens GN=C1QBP PE=1 SV=1 | 10,88 | 10,95 | 10 | 31,91 |
| 1284 | sp|Q99417|MYCBP_HUMAN | HUMAN | C-Myc-binding protein OS=Homo sapiens GN=MYCBP PE=1 SV=3 | 10,87 | 11,03 | 6 | 71,84 |
| 1285 | sp|Q15370|ELOB_HUMAN | HUMAN | Transcription elongation factor B polypeptide 2 OS=Homo sapiens GN=TCEB2 PE=1 SV=1 | 10,87 | 11,01 | 8 | 62,71 |
| 1286 | sp|Q9BRP8|PYM1_HUMAN | HUMAN | Partner of Y14 and mago OS=Homo sapiens GN=PYM1 PE=1 SV=1 | 10,87 | 10,92 | 6 | 50 |
| 1287 | sp|P13716|HEM2_HUMAN | HUMAN | Delta-aminolevulinic acid dehydratase OS=Homo sapiens GN=ALAD PE=1 SV=1 | 10,86 | 10,99 | 6 | 26,67 |
| 1288 | sp|Q5VYK3|ECM29_HUMAN | HUMAN | Proteasome-associated protein ECM29 homolog OS=Homo sapiens GN=ECM29 PE=1 SV=2 | 10,85 | 11,29 | 7 | 5,691 |
| 1289 | sp|Q8WZA0|LZIC_HUMAN | HUMAN | Protein LZIC OS=Homo sapiens GN=LZIC PE=1 SV=1 | 10,85 | 11,07 | 8 | 52,11 |
| 1290 | sp|Q9Y2V2|CHSP1_HUMAN | HUMAN | Calcium-regulated heat stable protein 1 OS=Homo sapiens GN=CARHSP1 PE=1 SV=2 | 10,84 | 11 | 6 | 72,11 |
| 1291 | sp|O95881|TXD12_HUMAN | HUMAN | Thioredoxin domain-containing protein 12 OS=Homo sapiens GN=TXNDC12 PE=1 SV=1 | 10,81 | 10,87 | 7 | 36,05 |
| 1292 | sp|Q8TD55|PKHO2_HUMAN | HUMAN | Pleckstrin homology domain-containing family O member 2 OS=Homo sapiens GN=PLEKHO2 PE=1 SV=1 | 10,8 | 10,85 | 7 | 18,78 |
| 1293 | sp|Q00325|MPCP_HUMAN | HUMAN | Phosphate carrier protein, mitochondrial OS=Homo sapiens GN=SLC25A3 PE=1 SV=2 | 10,79 | 11 | 9 | 21,27 |
| 1294 | sp|Q9Y383|LC7L2_HUMAN | HUMAN | Putative RNA-binding protein Luc7-like 2 OS=Homo sapiens GN=LUC7L2 PE=1 SV=2 | 10,78 | 10,98 | 5 | 16,84 |
| 1295 | sp|O15498|YKT6_HUMAN | HUMAN | Synaptobrevin homolog YKT6 OS=Homo sapiens GN=YKT6 PE=1 SV=1 | 10,78 | 10,87 | 6 | 39,39 |
| 1296 | sp|Q14451|GRB7_HUMAN | HUMAN | Growth factor receptor-bound protein 7 OS=Homo sapiens GN=GRB7 PE=1 SV=2 | 10,78 | 10,87 | 6 | 17,48 |
| 1297 | sp|Q9BRA2|TXD17_HUMAN | HUMAN | Thioredoxin domain-containing protein 17 OS=Homo sapiens GN=TXNDC17 PE=1 SV=1 | 10,77 | 10,81 | 6 | 60,16 |
| 1298 | sp|Q52LJ0|FA98B_HUMAN | HUMAN | Protein FAM98B OS=Homo sapiens GN=FAM98B PE=1 SV=1 | 10,76 | 11 | 7 | 29,09 |
| 1299 | sp|Q15121|PEA15_HUMAN | HUMAN | Astrocytic phosphoprotein PEA-15 OS=Homo sapiens GN=PEA15 PE=1 SV=2 | 10,76 | 10,81 | 5 | 44,62 |
| 1300 | sp|Q9BW30|TPPP3_HUMAN | HUMAN | Tubulin polymerization-promoting protein family member 3 OS=Homo sapiens GN=TPPP3 PE=1 SV=1 | 10,75 | 10,87 | 8 | 47,73 |
| 1301 | sp|Q13564|ULA1_HUMAN | HUMAN | NEDD8-activating enzyme E1 regulatory subunit OS=Homo sapiens GN=NAE1 PE=1 SV=1 | 10,73 | 10,92 | 9 | 17,04 |
| 1302 | sp|O75832|PSD10_HUMAN | HUMAN | 26S proteasome non-ATPase regulatory subunit 10 OS=Homo sapiens GN=PSMD10 PE=1 SV=1 | 10,71 | 10,75 | 9 | 35,4 |
| 1303 | sp|A6NI72|NCF1B_HUMAN | HUMAN | Putative neutrophil cytosol factor 1B OS=Homo sapiens GN=NCF1B PE=5 SV=2 | 10,68 | 10,91 | 7 | 28,64 |
| 1304 | sp|Q01970|PLCB3_HUMAN | HUMAN | 1-phosphatidylinositol 4,5-bisphosphate phosphodiesterase beta-3 OS=Homo sapiens GN=PLCB3 PE=1 SV=2 | 10,68 | 10,87 | 5 | 5,348 |
| 1305 | sp|P50579|MAP2_HUMAN | HUMAN | Methionine aminopeptidase 2 OS=Homo sapiens GN=METAP2 PE=1 SV=1 | 10,67 | 10,88 | 6 | 18,83 |
| 1306 | sp|O15347|HMGB3_HUMAN | HUMAN | High mobility group protein B3 OS=Homo sapiens GN=HMGB3 PE=1 SV=4 | 10,66 | 11,95 | 9 | 43,5 |
| 1307 | sp|P05413|FABPH_HUMAN | HUMAN | Fatty acid-binding protein, heart OS=Homo sapiens GN=FABP3 PE=1 SV=4 | 10,65 | 10,82 | 7 | 54,14 |
| 1308 | sp|P53990|IST1_HUMAN | HUMAN | IST1 homolog OS=Homo sapiens GN=IST1 PE=1 SV=1 | 10,64 | 10,69 | 5 | 17,86 |
| 1309 | sp|Q9H7Z7|PGES2_HUMAN | HUMAN | Prostaglandin E synthase 2 OS=Homo sapiens GN=PTGES2 PE=1 SV=1 | 10,64 | 10,69 | 5 | 20,95 |
| 1310 | sp|Q969G5|PRDBP_HUMAN | HUMAN | Protein kinase C delta-binding protein OS=Homo sapiens GN=PRKCDBP PE=1 SV=3 | 10,64 | 10,68 | 6 | 27,59 |
| 1311 | sp|Q9BTT0|AN32E_HUMAN | HUMAN | Acidic leucine-rich nuclear phosphoprotein 32 family member E OS=Homo sapiens GN=ANP32E PE=1 SV=1 | 10,64 | 10,68 | 7 | 23,13 |
| 1312 | sp|Q9H3K6|BOLA2_HUMAN | HUMAN | BolA-like protein 2 OS=Homo sapiens GN=BOLA2 PE=1 SV=1 | 10,61 | 10,65 | 6 | 72,09 |
| 1313 | sp|P30711|GSTT1_HUMAN | HUMAN | Glutathione S-transferase theta-1 OS=Homo sapiens GN=GSTT1 PE=1 SV=4 | 10,59 | 10,78 | 8 | 32,92 |
| 1314 | sp|Q8NFV4|ABHDB_HUMAN | HUMAN | Protein ABHD11 OS=Homo sapiens GN=ABHD11 PE=1 SV=1 | 10,59 | 10,73 | 7 | 27,62 |
| 1315 | sp|Q13509|TBB3_HUMAN | HUMAN | Tubulin beta-3 chain OS=Homo sapiens GN=TUBB3 PE=1 SV=2 | 10,57 | 42,9 | 64 | 61,11 |
| 1316 | sp|P46087|NOP2_HUMAN | HUMAN | Probable 28S rRNA (cytosine(4447)-C(5))-methyltransferase OS=Homo sapiens GN=NOP2 PE=1 SV=2 | 10,57 | 10,62 | 5 | 7,635 |
| 1317 | sp|P62899|RL31_HUMAN | HUMAN | 60S ribosomal protein L31 OS=Homo sapiens GN=RPL31 PE=1 SV=1 | 10,56 | 10,72 | 7 | 44,8 |
| 1318 | sp|Q9H6S3|ES8L2_HUMAN | HUMAN | Epidermal growth factor receptor kinase substrate 8-like protein 2 OS=Homo sapiens GN=EPS8L2 PE=1 SV=2 | 10,55 | 10,83 | 7 | 17,06 |
| 1319 | sp|P62263|RS14_HUMAN | HUMAN | 40S ribosomal protein S14 OS=Homo sapiens GN=RPS14 PE=1 SV=3 | 10,54 | 10,57 | 12 | 53,64 |
| 1320 | sp|P02746|C1QB_HUMAN | HUMAN | Complement C1q subcomponent subunit B OS=Homo sapiens GN=C1QB PE=1 SV=3 | 10,53 | 10,75 | 6 | 26,09 |
| 1321 | sp|P58107|EPIPL_HUMAN | HUMAN | Epiplakin OS=Homo sapiens GN=EPPK1 PE=1 SV=2 | 10,52 | 19,78 | 11 | 13,4 |
| 1322 | sp|O75131|CPNE3_HUMAN | HUMAN | Copine-3 OS=Homo sapiens GN=CPNE3 PE=1 SV=1 | 10,52 | 10,68 | 6 | 16,39 |
| 1323 | sp|P80217|IN35_HUMAN | HUMAN | Interferon-induced 35 kDa protein OS=Homo sapiens GN=IFI35 PE=1 SV=5 | 10,51 | 10,71 | 5 | 24,83 |
| 1324 | sp|P35914|HMGCL_HUMAN | HUMAN | Hydroxymethylglutaryl-CoA lyase, mitochondrial OS=Homo sapiens GN=HMGCL PE=1 SV=2 | 10,5 | 10,75 | 8 | 25,54 |
| 1325 | sp|P08579|RU2B_HUMAN | HUMAN | U2 small nuclear ribonucleoprotein B'' OS=Homo sapiens GN=SNRPB2 PE=1 SV=1 | 10,49 | 10,71 | 8 | 27,56 |
| 1326 | sp|P49902|5NTC_HUMAN | HUMAN | Cytosolic purine 5'-nucleotidase OS=Homo sapiens GN=NT5C2 PE=1 SV=1 | 10,43 | 10,59 | 7 | 18,54 |
| 1327 | sp|P33991|MCM4_HUMAN | HUMAN | DNA replication licensing factor MCM4 OS=Homo sapiens GN=MCM4 PE=1 SV=5 | 10,38 | 11,93 | 7 | 8,111 |
| 1328 | sp|P30042|ES1_HUMAN | HUMAN | ES1 protein homolog, mitochondrial OS=Homo sapiens GN=C21orf33 PE=1 SV=3 | 10,38 | 10,51 | 8 | 37,69 |
| 1329 | sp|Q9NXV6|CARF_HUMAN | HUMAN | CDKN2A-interacting protein OS=Homo sapiens GN=CDKN2AIP PE=1 SV=3 | 10,37 | 10,62 | 7 | 16,55 |
| 1330 | sp|P09668|CATH_HUMAN | HUMAN | Pro-cathepsin H OS=Homo sapiens GN=CTSH PE=1 SV=4 | 10,37 | 10,51 | 6 | 20,9 |
| 1331 | sp|P19835|CEL_HUMAN | HUMAN | Bile salt-activated lipase OS=Homo sapiens GN=CEL PE=1 SV=3 | 10,36 | 10,52 | 6 | 10,23 |
| 1332 | sp|Q9NR46|SHLB2_HUMAN | HUMAN | Endophilin-B2 OS=Homo sapiens GN=SH3GLB2 PE=1 SV=1 | 10,34 | 11,53 | 10 | 25,32 |
| 1333 | sp|Q02252|MMSA_HUMAN | HUMAN | Methylmalonate-semialdehyde dehydrogenase [acylating], mitochondrial OS=Homo sapiens GN=ALDH6A1 PE=1 SV=2 | 10,32 | 10,49 | 6 | 13,46 |
| 1334 | sp|Q9NT62|ATG3_HUMAN | HUMAN | Ubiquitin-like-conjugating enzyme ATG3 OS=Homo sapiens GN=ATG3 PE=1 SV=1 | 10,3 | 10,37 | 5 | 19,43 |
| 1335 | sp|Q9ULC4|MCTS1_HUMAN | HUMAN | Malignant T-cell-amplified sequence 1 OS=Homo sapiens GN=MCTS1 PE=1 SV=1 | 10,29 | 10,46 | 7 | 46,96 |
| 1336 | sp|Q9P2M7|CING_HUMAN | HUMAN | Cingulin OS=Homo sapiens GN=CGN PE=1 SV=2 | 10,28 | 11,03 | 6 | 5,43 |
| 1337 | sp|Q96BQ1|FAM3D_HUMAN | HUMAN | Protein FAM3D OS=Homo sapiens GN=FAM3D PE=1 SV=1 | 10,27 | 10,38 | 5 | 31,25 |
| 1338 | sp|P84090|ERH_HUMAN | HUMAN | Enhancer of rudimentary homolog OS=Homo sapiens GN=ERH PE=1 SV=1 | 10,27 | 10,29 | 6 | 44,23 |
| 1339 | sp|Q9BUH6|PAXX_HUMAN | HUMAN | Protein PAXX OS=Homo sapiens GN=C9orf142 PE=1 SV=2 | 10,25 | 10,47 | 6 | 39,71 |
| 1340 | sp|P12724|ECP_HUMAN | HUMAN | Eosinophil cationic protein OS=Homo sapiens GN=RNASE3 PE=1 SV=2 | 10,25 | 10,41 | 9 | 40,63 |
| 1341 | sp|P46778|RL21_HUMAN | HUMAN | 60S ribosomal protein L21 OS=Homo sapiens GN=RPL21 PE=1 SV=2 | 10,24 | 10,41 | 7 | 37,5 |
| 1342 | sp|P05546|HEP2_HUMAN | HUMAN | Heparin cofactor 2 OS=Homo sapiens GN=SERPIND1 PE=1 SV=3 | 10,23 | 11,5 | 7 | 10,82 |
| 1343 | sp|P11177|ODPB_HUMAN | HUMAN | Pyruvate dehydrogenase E1 component subunit beta, mitochondrial OS=Homo sapiens GN=PDHB PE=1 SV=3 | 10,23 | 10,4 | 7 | 23,12 |
| 1344 | sp|Q9BVG4|PBDC1_HUMAN | HUMAN | Protein PBDC1 OS=Homo sapiens GN=PBDC1 PE=1 SV=1 | 10,21 | 10,34 | 5 | 24,46 |
| 1345 | sp|Q8IWE2|NXP20_HUMAN | HUMAN | Protein NOXP20 OS=Homo sapiens GN=FAM114A1 PE=1 SV=2 | 10,19 | 10,32 | 6 | 12,61 |
| 1346 | sp|O43598|DNPH1_HUMAN | HUMAN | 2'-deoxynucleoside 5'-phosphate N-hydrolase 1 OS=Homo sapiens GN=DNPH1 PE=1 SV=1 | 10,19 | 10,31 | 6 | 44,83 |
| 1347 | sp|Q8IVF2|AHNK2_HUMAN | HUMAN | Protein AHNAK2 OS=Homo sapiens GN=AHNAK2 PE=1 SV=2 | 10,18 | 10,86 | 8 | 11,35 |
| 1348 | sp|P17900|SAP3_HUMAN | HUMAN | Ganglioside GM2 activator OS=Homo sapiens GN=GM2A PE=1 SV=4 | 10,18 | 10,28 | 7 | 37,82 |
| 1349 | sp|P36405|ARL3_HUMAN | HUMAN | ADP-ribosylation factor-like protein 3 OS=Homo sapiens GN=ARL3 PE=1 SV=2 | 10,17 | 10,2 | 5 | 34,07 |
| 1350 | sp|Q9UBQ0|VPS29_HUMAN | HUMAN | Vacuolar protein sorting-associated protein 29 OS=Homo sapiens GN=VPS29 PE=1 SV=1 | 10,16 | 10,29 | 5 | 37,36 |
| 1351 | sp|P62942|FKB1A_HUMAN | HUMAN | Peptidyl-prolyl cis-trans isomerase FKBP1A OS=Homo sapiens GN=FKBP1A PE=1 SV=2 | 10,16 | 10,18 | 9 | 71,3 |
| 1352 | sp|O14737|PDCD5_HUMAN | HUMAN | Programmed cell death protein 5 OS=Homo sapiens GN=PDCD5 PE=1 SV=3 | 10,15 | 10,34 | 8 | 54,4 |
| 1353 | sp|Q9BQG0|MBB1A_HUMAN | HUMAN | Myb-binding protein 1A OS=Homo sapiens GN=MYBBP1A PE=1 SV=2 | 10,13 | 10,49 | 6 | 6,551 |
| 1354 | sp|Q9UM54|MYO6_HUMAN | HUMAN | Unconventional myosin-VI OS=Homo sapiens GN=MYO6 PE=1 SV=4 | 10,12 | 10,63 | 6 | 6,414 |
| 1355 | sp|P17612|KAPCA_HUMAN | HUMAN | cAMP-dependent protein kinase catalytic subunit alpha OS=Homo sapiens GN=PRKACA PE=1 SV=2 | 10,11 | 10,32 | 8 | 31,05 |
| 1356 | sp|Q92688|AN32B_HUMAN | HUMAN | Acidic leucine-rich nuclear phosphoprotein 32 family member B OS=Homo sapiens GN=ANP32B PE=1 SV=1 | 10,1 | 24,35 | 25 | 48,21 |
| 1357 | sp|P05451|REG1A_HUMAN | HUMAN | Lithostathine-1-alpha OS=Homo sapiens GN=REG1A PE=1 SV=3 | 10,1 | 10,16 | 6 | 45,78 |
| 1358 | sp|P01591|IGJ_HUMAN | HUMAN | Immunoglobulin J chain OS=Homo sapiens GN=JCHAIN PE=1 SV=4 | 10,09 | 10,09 | 10 | 42,14 |
| 1359 | sp|Q8IYB3|SRRM1_HUMAN | HUMAN | Serine/arginine repetitive matrix protein 1 OS=Homo sapiens GN=SRRM1 PE=1 SV=2 | 10,08 | 10,28 | 7 | 9,956 |
| 1360 | sp|Q86U42|PABP2_HUMAN | HUMAN | Polyadenylate-binding protein 2 OS=Homo sapiens GN=PABPN1 PE=1 SV=3 | 10,08 | 10,23 | 7 | 37,25 |
| 1361 | sp|P51665|PSMD7_HUMAN | HUMAN | 26S proteasome non-ATPase regulatory subunit 7 OS=Homo sapiens GN=PSMD7 PE=1 SV=2 | 10,07 | 10,29 | 6 | 28,09 |
| 1362 | sp|Q16222|UAP1_HUMAN | HUMAN | UDP-N-acetylhexosamine pyrophosphorylase OS=Homo sapiens GN=UAP1 PE=1 SV=3 | 10,06 | 10,21 | 6 | 14,18 |
| 1363 | sp|Q9Y2W2|WBP11_HUMAN | HUMAN | WW domain-binding protein 11 OS=Homo sapiens GN=WBP11 PE=1 SV=1 | 10,06 | 10,16 | 5 | 13,42 |
| 1364 | sp|Q9BSJ8|ESYT1_HUMAN | HUMAN | Extended synaptotagmin-1 OS=Homo sapiens GN=ESYT1 PE=1 SV=1 | 10,06 | 10,08 | 5 | 6,793 |
| 1365 | sp|P49903|SPS1_HUMAN | HUMAN | Selenide, water dikinase 1 OS=Homo sapiens GN=SEPHS1 PE=1 SV=2 | 10,05 | 10,23 | 6 | 16,33 |
| 1366 | sp|Q9HA64|KT3K_HUMAN | HUMAN | Ketosamine-3-kinase OS=Homo sapiens GN=FN3KRP PE=1 SV=2 | 10,04 | 10,31 | 6 | 18,77 |
| 1367 | sp|P46976|GLYG_HUMAN | HUMAN | Glycogenin-1 OS=Homo sapiens GN=GYG1 PE=1 SV=4 | 10,04 | 10,05 | 5 | 17,43 |
| 1368 | sp|P16615|AT2A2_HUMAN | HUMAN | Sarcoplasmic/endoplasmic reticulum calcium ATPase 2 OS=Homo sapiens GN=ATP2A2 PE=1 SV=1 | 10,03 | 10,4 | 6 | 9,213 |
| 1369 | sp|P16401|H15_HUMAN | HUMAN | Histone H1.5 OS=Homo sapiens GN=HIST1H1B PE=1 SV=3 | 10,02 | 12,52 | 6 | 25,66 |
| 1370 | sp|Q07812|BAX_HUMAN | HUMAN | Apoptosis regulator BAX OS=Homo sapiens GN=BAX PE=1 SV=1 | 10,02 | 10,03 | 5 | 35,94 |
| 1371 | sp|P49792|RBP2_HUMAN | HUMAN | E3 SUMO-protein ligase RanBP2 OS=Homo sapiens GN=RANBP2 PE=1 SV=2 | 10,01 | 11,25 | 8 | 3,784 |
| 1372 | sp|P52888|THOP1_HUMAN | HUMAN | Thimet oligopeptidase OS=Homo sapiens GN=THOP1 PE=1 SV=2 | 10,01 | 10,32 | 6 | 10,45 |
| 1373 | sp|Q8TD06|AGR3_HUMAN | HUMAN | Anterior gradient protein 3 OS=Homo sapiens GN=AGR3 PE=1 SV=1 | 10 | 12 | 9 | 40,36 |
| 1374 | sp|Q06124|PTN11_HUMAN | HUMAN | Tyrosine-protein phosphatase non-receptor type 11 OS=Homo sapiens GN=PTPN11 PE=1 SV=2 | 10 | 10,26 | 6 | 14,24 |
| 1375 | sp|Q86XP3|DDX42_HUMAN | HUMAN | ATP-dependent RNA helicase DDX42 OS=Homo sapiens GN=DDX42 PE=1 SV=1 | 10 | 10,21 | 6 | 10,55 |
| 1376 | sp|Q16775|GLO2_HUMAN | HUMAN | Hydroxyacylglutathione hydrolase, mitochondrial OS=Homo sapiens GN=HAGH PE=1 SV=2 | 10 | 10,02 | 5 | 19,81 |
| 1377 | sp|Q02750|MP2K1_HUMAN | HUMAN | Dual specificity mitogen-activated protein kinase kinase 1 OS=Homo sapiens GN=MAP2K1 PE=1 SV=2 | 9,99 | 10,31 | 7 | 24,17 |
| 1378 | sp|P14678|RSMB_HUMAN | HUMAN | Small nuclear ribonucleoprotein-associated proteins B and B' OS=Homo sapiens GN=SNRPB PE=1 SV=2 | 9,96 | 10,04 | 9 | 34,17 |
| 1379 | sp|Q9P2R7|SUCB1_HUMAN | HUMAN | Succinyl-CoA ligase [ADP-forming] subunit beta, mitochondrial OS=Homo sapiens GN=SUCLA2 PE=1 SV=3 | 9,95 | 11,22 | 7 | 17,49 |
| 1380 | sp|P23921|RIR1_HUMAN | HUMAN | Ribonucleoside-diphosphate reductase large subunit OS=Homo sapiens GN=RRM1 PE=1 SV=1 | 9,95 | 10,22 | 6 | 9,091 |
| 1381 | sp|P60983|GMFB_HUMAN | HUMAN | Glia maturation factor beta OS=Homo sapiens GN=GMFB PE=1 SV=2 | 9,93 | 10 | 6 | 48,59 |
| 1382 | sp|P61923|COPZ1_HUMAN | HUMAN | Coatomer subunit zeta-1 OS=Homo sapiens GN=COPZ1 PE=1 SV=1 | 9,92 | 9,96 | 7 | 39,55 |
| 1383 | sp|Q14738|2A5D_HUMAN | HUMAN | Serine/threonine-protein phosphatase 2A 56 kDa regulatory subunit delta isoform OS=Homo sapiens GN=PPP2R5D PE=1 SV=1 | 9,91 | 10,02 | 5 | 11,79 |
| 1384 | sp|Q15437|SC23B_HUMAN | HUMAN | Protein transport protein Sec23B OS=Homo sapiens GN=SEC23B PE=1 SV=2 | 9,9 | 16,99 | 11 | 18,9 |
| 1385 | sp|Q8N1F7|NUP93_HUMAN | HUMAN | Nuclear pore complex protein Nup93 OS=Homo sapiens GN=NUP93 PE=1 SV=2 | 9,9 | 10,27 | 6 | 9,768 |
| 1386 | sp|P08236|BGLR_HUMAN | HUMAN | Beta-glucuronidase OS=Homo sapiens GN=GUSB PE=1 SV=2 | 9,89 | 11,13 | 7 | 11,06 |
| 1387 | sp|P62847|RS24_HUMAN | HUMAN | 40S ribosomal protein S24 OS=Homo sapiens GN=RPS24 PE=1 SV=1 | 9,89 | 10,01 | 5 | 34,59 |
| 1388 | sp|Q9Y6Y8|S23IP_HUMAN | HUMAN | SEC23-interacting protein OS=Homo sapiens GN=SEC23IP PE=1 SV=1 | 9,88 | 10,11 | 6 | 8,7 |
| 1389 | sp|Q9P2R3|ANFY1_HUMAN | HUMAN | Rabankyrin-5 OS=Homo sapiens GN=ANKFY1 PE=1 SV=2 | 9,84 | 10,15 | 6 | 6,416 |
| 1390 | sp|Q5VV41|ARHGG_HUMAN | HUMAN | Rho guanine nucleotide exchange factor 16 OS=Homo sapiens GN=ARHGEF16 PE=1 SV=1 | 9,8 | 9,87 | 5 | 9,309 |
| 1391 | sp|O43795|MYO1B_HUMAN | HUMAN | Unconventional myosin-Ib OS=Homo sapiens GN=MYO1B PE=1 SV=3 | 9,77 | 11,96 | 7 | 7,746 |
| 1392 | sp|Q6P1N9|TATD1_HUMAN | HUMAN | Putative deoxyribonuclease TATDN1 OS=Homo sapiens GN=TATDN1 PE=1 SV=2 | 9,76 | 9,95 | 7 | 27,61 |
| 1393 | sp|Q9BQA1|MEP50_HUMAN | HUMAN | Methylosome protein 50 OS=Homo sapiens GN=WDR77 PE=1 SV=1 | 9,72 | 9,77 | 5 | 21,93 |
| 1394 | sp|Q12765|SCRN1_HUMAN | HUMAN | Secernin-1 OS=Homo sapiens GN=SCRN1 PE=1 SV=2 | 9,7 | 9,78 | 5 | 16,67 |
| 1395 | sp|P62328|TYB4_HUMAN | HUMAN | Thymosin beta-4 OS=Homo sapiens GN=TMSB4X PE=1 SV=2 | 9,68 | 10,29 | 18 | 81,82 |
| 1396 | sp|Q8NBJ7|SUMF2_HUMAN | HUMAN | Sulfatase-modifying factor 2 OS=Homo sapiens GN=SUMF2 PE=1 SV=2 | 9,67 | 9,89 | 6 | 24,58 |
| 1397 | sp|Q01130|SRSF2_HUMAN | HUMAN | Serine/arginine-rich splicing factor 2 OS=Homo sapiens GN=SRSF2 PE=1 SV=4 | 9,65 | 10,05 | 8 | 30,77 |
| 1398 | sp|P26440|IVD_HUMAN | HUMAN | Isovaleryl-CoA dehydrogenase, mitochondrial OS=Homo sapiens GN=IVD PE=1 SV=1 | 9,65 | 9,77 | 6 | 15,6 |
| 1399 | sp|P39656|OST48_HUMAN | HUMAN | Dolichyl-diphosphooligosaccharide--protein glycosyltransferase 48 kDa subunit OS=Homo sapiens GN=DDOST PE=1 SV=4 | 9,63 | 10,19 | 7 | 21,93 |
| 1400 | sp|Q9Y5K5|UCHL5_HUMAN | HUMAN | Ubiquitin carboxyl-terminal hydrolase isozyme L5 OS=Homo sapiens GN=UCHL5 PE=1 SV=3 | 9,63 | 9,82 | 6 | 20,06 |
| 1401 | sp|Q96RP9|EFGM_HUMAN | HUMAN | Elongation factor G, mitochondrial OS=Homo sapiens GN=GFM1 PE=1 SV=2 | 9,61 | 9,91 | 6 | 9,587 |
| 1402 | sp|P23919|KTHY_HUMAN | HUMAN | Thymidylate kinase OS=Homo sapiens GN=DTYMK PE=1 SV=4 | 9,6 | 9,88 | 7 | 33,02 |
| 1403 | sp|Q9GZZ1|NAA50_HUMAN | HUMAN | N-alpha-acetyltransferase 50 OS=Homo sapiens GN=NAA50 PE=1 SV=1 | 9,6 | 9,8 | 6 | 40,83 |
| 1404 | sp|P62910|RL32_HUMAN | HUMAN | 60S ribosomal protein L32 OS=Homo sapiens GN=RPL32 PE=1 SV=2 | 9,6 | 9,69 | 6 | 45,93 |
| 1405 | sp|Q6FI81|CPIN1_HUMAN | HUMAN | Anamorsin OS=Homo sapiens GN=CIAPIN1 PE=1 SV=2 | 9,6 | 9,68 | 5 | 17,95 |
| 1406 | sp|Q9NR19|ACSA_HUMAN | HUMAN | Acetyl-coenzyme A synthetase, cytoplasmic OS=Homo sapiens GN=ACSS2 PE=1 SV=1 | 9,58 | 9,7 | 5 | 10,27 |
| 1407 | sp|Q9H910|HN1L_HUMAN | HUMAN | Hematological and neurological expressed 1-like protein OS=Homo sapiens GN=HN1L PE=1 SV=1 | 9,58 | 9,69 | 6 | 44,21 |
| 1408 | sp|Q13153|PAK1_HUMAN | HUMAN | Serine/threonine-protein kinase PAK 1 OS=Homo sapiens GN=PAK1 PE=1 SV=2 | 9,55 | 18,99 | 15 | 25,87 |
| 1409 | sp|Q16629|SRSF7_HUMAN | HUMAN | Serine/arginine-rich splicing factor 7 OS=Homo sapiens GN=SRSF7 PE=1 SV=1 | 9,53 | 12,39 | 9 | 28,15 |
| 1410 | sp|Q6GMV3|PTRD1_HUMAN | HUMAN | Putative peptidyl-tRNA hydrolase PTRHD1 OS=Homo sapiens GN=PTRHD1 PE=1 SV=1 | 9,53 | 9,66 | 8 | 82,14 |
| 1411 | sp|P63151|2ABA_HUMAN | HUMAN | Serine/threonine-protein phosphatase 2A 55 kDa regulatory subunit B alpha isoform OS=Homo sapiens GN=PPP2R2A PE=1 SV=1 | 9,52 | 9,64 | 5 | 13,2 |
| 1412 | sp|Q9NZL4|HPBP1_HUMAN | HUMAN | Hsp70-binding protein 1 OS=Homo sapiens GN=HSPBP1 PE=1 SV=1 | 9,5 | 9,62 | 6 | 22,38 |
| 1413 | sp|P13284|GILT_HUMAN | HUMAN | Gamma-interferon-inducible lysosomal thiol reductase OS=Homo sapiens GN=IFI30 PE=1 SV=3 | 9,49 | 9,58 | 15 | 33,6 |
| 1414 | sp|Q9NWV4|CA123_HUMAN | HUMAN | UPF0587 protein C1orf123 OS=Homo sapiens GN=C1orf123 PE=1 SV=1 | 9,49 | 9,56 | 5 | 41,25 |
| 1415 | sp|Q99795|GPA33_HUMAN | HUMAN | Cell surface A33 antigen OS=Homo sapiens GN=GPA33 PE=1 SV=1 | 9,48 | 9,57 | 7 | 29,15 |
| 1416 | sp|O14618|CCS_HUMAN | HUMAN | Copper chaperone for superoxide dismutase OS=Homo sapiens GN=CCS PE=1 SV=1 | 9,48 | 9,55 | 6 | 26,64 |
| 1417 | sp|P31689|DNJA1_HUMAN | HUMAN | DnaJ homolog subfamily A member 1 OS=Homo sapiens GN=DNAJA1 PE=1 SV=2 | 9,45 | 9,66 | 6 | 20,65 |
| 1418 | sp|Q9NUV9|GIMA4_HUMAN | HUMAN | GTPase IMAP family member 4 OS=Homo sapiens GN=GIMAP4 PE=1 SV=1 | 9,44 | 9,55 | 5 | 24,01 |
| 1419 | sp|Q9BZQ8|NIBAN_HUMAN | HUMAN | Protein Niban OS=Homo sapiens GN=FAM129A PE=1 SV=1 | 9,42 | 9,66 | 7 | 11,96 |
| 1420 | sp|P26885|FKBP2_HUMAN | HUMAN | Peptidyl-prolyl cis-trans isomerase FKBP2 OS=Homo sapiens GN=FKBP2 PE=1 SV=2 | 9,41 | 9,48 | 8 | 43,66 |
| 1421 | sp|Q99471|PFD5_HUMAN | HUMAN | Prefoldin subunit 5 OS=Homo sapiens GN=PFDN5 PE=1 SV=2 | 9,4 | 9,45 | 5 | 41,56 |
| 1422 | sp|Q13151|ROA0_HUMAN | HUMAN | Heterogeneous nuclear ribonucleoprotein A0 OS=Homo sapiens GN=HNRNPA0 PE=1 SV=1 | 9,39 | 13,86 | 11 | 34,1 |
| 1423 | sp|P10301|RRAS_HUMAN | HUMAN | Ras-related protein R-Ras OS=Homo sapiens GN=RRAS PE=1 SV=1 | 9,38 | 9,47 | 6 | 33,03 |
| 1424 | sp|Q92600|RCD1_HUMAN | HUMAN | Cell differentiation protein RCD1 homolog OS=Homo sapiens GN=RQCD1 PE=1 SV=1 | 9,37 | 9,49 | 6 | 23,75 |
| 1425 | sp|P01033|TIMP1_HUMAN | HUMAN | Metalloproteinase inhibitor 1 OS=Homo sapiens GN=TIMP1 PE=1 SV=1 | 9,36 | 9,44 | 5 | 39,13 |
| 1426 | sp|Q6YN16|HSDL2_HUMAN | HUMAN | Hydroxysteroid dehydrogenase-like protein 2 OS=Homo sapiens GN=HSDL2 PE=1 SV=1 | 9,36 | 9,42 | 6 | 16,75 |
| 1427 | sp|P28065|PSB9_HUMAN | HUMAN | Proteasome subunit beta type-9 OS=Homo sapiens GN=PSMB9 PE=1 SV=2 | 9,36 | 9,42 | 5 | 30,59 |
| 1428 | sp|Q9UBY9|HSPB7_HUMAN | HUMAN | Heat shock protein beta-7 OS=Homo sapiens GN=HSPB7 PE=1 SV=1 | 9,34 | 9,42 | 5 | 45,29 |
| 1429 | sp|Q8WX92|NELFB_HUMAN | HUMAN | Negative elongation factor B OS=Homo sapiens GN=NELFB PE=1 SV=1 | 9,33 | 9,7 | 7 | 17,76 |
| 1430 | sp|O75569|PRKRA_HUMAN | HUMAN | Interferon-inducible double-stranded RNA-dependent protein kinase activator A OS=Homo sapiens GN=PRKRA PE=1 SV=1 | 9,33 | 9,37 | 5 | 23 |
| 1431 | sp|P32456|GBP2_HUMAN | HUMAN | Guanylate-binding protein 2 OS=Homo sapiens GN=GBP2 PE=1 SV=3 | 9,31 | 14,66 | 12 | 15,74 |
| 1432 | sp|P00387|NB5R3_HUMAN | HUMAN | NADH-cytochrome b5 reductase 3 OS=Homo sapiens GN=CYB5R3 PE=1 SV=3 | 9,3 | 9,41 | 5 | 20,6 |
| 1433 | sp|Q53GQ0|DHB12_HUMAN | HUMAN | Very-long-chain 3-oxoacyl-CoA reductase OS=Homo sapiens GN=HSD17B12 PE=1 SV=2 | 9,3 | 9,36 | 5 | 25,32 |
| 1434 | sp|Q01085|TIAR_HUMAN | HUMAN | Nucleolysin TIAR OS=Homo sapiens GN=TIAL1 PE=1 SV=1 | 9,3 | 9,35 | 6 | 20 |
| 1435 | sp|P10515|ODP2_HUMAN | HUMAN | Dihydrolipoyllysine-residue acetyltransferase component of pyruvate dehydrogenase complex, mitochondrial OS=Homo sapiens GN=DLAT PE=1 SV=3 | 9,29 | 9,69 | 7 | 16,69 |
| 1436 | sp|P78318|IGBP1_HUMAN | HUMAN | Immunoglobulin-binding protein 1 OS=Homo sapiens GN=IGBP1 PE=1 SV=1 | 9,29 | 9,34 | 5 | 19,76 |
| 1437 | sp|P48681|NEST_HUMAN | HUMAN | Nestin OS=Homo sapiens GN=NES PE=1 SV=2 | 9,28 | 9,63 | 6 | 4,195 |
| 1438 | sp|Q96EP5|DAZP1_HUMAN | HUMAN | DAZ-associated protein 1 OS=Homo sapiens GN=DAZAP1 PE=1 SV=1 | 9,26 | 9,33 | 6 | 20,64 |
| 1439 | sp|O60493|SNX3_HUMAN | HUMAN | Sorting nexin-3 OS=Homo sapiens GN=SNX3 PE=1 SV=3 | 9,25 | 11,53 | 6 | 35,8 |
| 1440 | sp|P62829|RL23_HUMAN | HUMAN | 60S ribosomal protein L23 OS=Homo sapiens GN=RPL23 PE=1 SV=1 | 9,24 | 9,33 | 8 | 43,57 |
| 1441 | sp|P24158|PRTN3_HUMAN | HUMAN | Myeloblastin OS=Homo sapiens GN=PRTN3 PE=1 SV=3 | 9,23 | 9,29 | 10 | 40,23 |
| 1442 | sp|P16104|H2AX_HUMAN | HUMAN | Histone H2AX OS=Homo sapiens GN=H2AFX PE=1 SV=2 | 9,21 | 9,29 | 12 | 32,17 |
| 1443 | sp|P11172|UMPS_HUMAN | HUMAN | Uridine 5'-monophosphate synthase OS=Homo sapiens GN=UMPS PE=1 SV=1 | 9,17 | 9,37 | 4 | 13,33 |
| 1444 | sp|Q8WXX5|DNJC9_HUMAN | HUMAN | DnaJ homolog subfamily C member 9 OS=Homo sapiens GN=DNAJC9 PE=1 SV=1 | 9,16 | 9,46 | 7 | 37,31 |
| 1445 | sp|P52292|IMA1_HUMAN | HUMAN | Importin subunit alpha-1 OS=Homo sapiens GN=KPNA2 PE=1 SV=1 | 9,16 | 9,3 | 6 | 13,42 |
| 1446 | sp|Q15126|PMVK_HUMAN | HUMAN | Phosphomevalonate kinase OS=Homo sapiens GN=PMVK PE=1 SV=3 | 9,16 | 9,24 | 5 | 31,25 |
| 1447 | sp|Q13242|SRSF9_HUMAN | HUMAN | Serine/arginine-rich splicing factor 9 OS=Homo sapiens GN=SRSF9 PE=1 SV=1 | 9,15 | 9,33 | 6 | 30,77 |
| 1448 | sp|Q9Y570|PPME1_HUMAN | HUMAN | Protein phosphatase methylesterase 1 OS=Homo sapiens GN=PPME1 PE=1 SV=3 | 9,15 | 9,29 | 4 | 12,95 |
| 1449 | sp|P02753|RET4_HUMAN | HUMAN | Retinol-binding protein 4 OS=Homo sapiens GN=RBP4 PE=1 SV=3 | 9,14 | 9,26 | 7 | 33,33 |
| 1450 | sp|P47813|IF1AX_HUMAN | HUMAN | Eukaryotic translation initiation factor 1A, X-chromosomal OS=Homo sapiens GN=EIF1AX PE=1 SV=2 | 9,12 | 9,23 | 5 | 36,11 |
| 1451 | sp|Q8NFU3|TSTD1_HUMAN | HUMAN | Thiosulfate sulfurtransferase/rhodanese-like domain-containing protein 1 OS=Homo sapiens GN=TSTD1 PE=1 SV=3 | 9,07 | 9,19 | 12 | 80 |
| 1452 | sp|O96019|ACL6A_HUMAN | HUMAN | Actin-like protein 6A OS=Homo sapiens GN=ACTL6A PE=1 SV=1 | 9,06 | 9,76 | 6 | 19,35 |
| 1453 | sp|Q99816|TS101_HUMAN | HUMAN | Tumor susceptibility gene 101 protein OS=Homo sapiens GN=TSG101 PE=1 SV=2 | 9,04 | 9,22 | 5 | 16,15 |
| 1454 | sp|P49207|RL34_HUMAN | HUMAN | 60S ribosomal protein L34 OS=Homo sapiens GN=RPL34 PE=1 SV=3 | 9,04 | 9,13 | 5 | 29,06 |
| 1455 | sp|O95777|LSM8_HUMAN | HUMAN | U6 snRNA-associated Sm-like protein LSm8 OS=Homo sapiens GN=LSM8 PE=1 SV=3 | 9,04 | 9,07 | 7 | 69,79 |
| 1456 | sp|P57764|GSDMD_HUMAN | HUMAN | Gasdermin-D OS=Homo sapiens GN=GSDMD PE=1 SV=1 | 9 | 9,15 | 7 | 19,83 |
| 1457 | sp|Q9Y3C8|UFC1_HUMAN | HUMAN | Ubiquitin-fold modifier-conjugating enzyme 1 OS=Homo sapiens GN=UFC1 PE=1 SV=3 | 8,99 | 9,03 | 5 | 29,34 |
| 1458 | sp|P35754|GLRX1_HUMAN | HUMAN | Glutaredoxin-1 OS=Homo sapiens GN=GLRX PE=1 SV=2 | 8,97 | 9,02 | 8 | 50,94 |
| 1459 | sp|P62140|PP1B_HUMAN | HUMAN | Serine/threonine-protein phosphatase PP1-beta catalytic subunit OS=Homo sapiens GN=PPP1CB PE=1 SV=3 | 8,91 | 32 | 20 | 64,53 |
| 1460 | sp|Q8N335|GPD1L_HUMAN | HUMAN | Glycerol-3-phosphate dehydrogenase 1-like protein OS=Homo sapiens GN=GPD1L PE=1 SV=1 | 8,89 | 10,63 | 9 | 29,91 |
| 1461 | sp|Q13451|FKBP5_HUMAN | HUMAN | Peptidyl-prolyl cis-trans isomerase FKBP5 OS=Homo sapiens GN=FKBP5 PE=1 SV=2 | 8,89 | 9,62 | 7 | 19,04 |
| 1462 | sp|Q92530|PSMF1_HUMAN | HUMAN | Proteasome inhibitor PI31 subunit OS=Homo sapiens GN=PSMF1 PE=1 SV=2 | 8,88 | 8,92 | 5 | 23,62 |
| 1463 | sp|Q99959|PKP2_HUMAN | HUMAN | Plakophilin-2 OS=Homo sapiens GN=PKP2 PE=1 SV=2 | 8,87 | 9,11 | 5 | 7,264 |
| 1464 | sp|P49756|RBM25_HUMAN | HUMAN | RNA-binding protein 25 OS=Homo sapiens GN=RBM25 PE=1 SV=3 | 8,87 | 8,99 | 5 | 8,66 |
| 1465 | sp|P06753|TPM3_HUMAN | HUMAN | Tropomyosin alpha-3 chain OS=Homo sapiens GN=TPM3 PE=1 SV=2 | 8,86 | 46,86 | 52 | 50,18 |
| 1466 | sp|P19838|NFKB1_HUMAN | HUMAN | Nuclear factor NF-kappa-B p105 subunit OS=Homo sapiens GN=NFKB1 PE=1 SV=2 | 8,83 | 9,26 | 7 | 8,264 |
| 1467 | sp|P06730|IF4E_HUMAN | HUMAN | Eukaryotic translation initiation factor 4E OS=Homo sapiens GN=EIF4E PE=1 SV=2 | 8,83 | 9,03 | 7 | 42,4 |
| 1468 | sp|P15531|NDKA_HUMAN | HUMAN | Nucleoside diphosphate kinase A OS=Homo sapiens GN=NME1 PE=1 SV=1 | 8,82 | 17,41 | 21 | 70,39 |
| 1469 | sp|P31040|SDHA_HUMAN | HUMAN | Succinate dehydrogenase [ubiquinone] flavoprotein subunit, mitochondrial OS=Homo sapiens GN=SDHA PE=1 SV=2 | 8,82 | 9,01 | 4 | 7,982 |
| 1470 | sp|P01861|IGHG4_HUMAN | HUMAN | Ig gamma-4 chain C region OS=Homo sapiens GN=IGHG4 PE=1 SV=1 | 8,81 | 36,2 | 70 | 78,59 |
| 1471 | sp|P55212|CASP6_HUMAN | HUMAN | Caspase-6 OS=Homo sapiens GN=CASP6 PE=1 SV=2 | 8,81 | 8,91 | 4 | 15,7 |
| 1472 | sp|P62854|RS26_HUMAN | HUMAN | 40S ribosomal protein S26 OS=Homo sapiens GN=RPS26 PE=1 SV=3 | 8,8 | 8,91 | 6 | 50,43 |
| 1473 | sp|Q4VC31|CCD58_HUMAN | HUMAN | Coiled-coil domain-containing protein 58 OS=Homo sapiens GN=CCDC58 PE=1 SV=1 | 8,8 | 8,9 | 5 | 45,14 |
| 1474 | sp|Q9UBQ5|EIF3K_HUMAN | HUMAN | Eukaryotic translation initiation factor 3 subunit K OS=Homo sapiens GN=EIF3K PE=1 SV=1 | 8,79 | 8,88 | 5 | 31,65 |
| 1475 | sp|Q7Z4V5|HDGR2_HUMAN | HUMAN | Hepatoma-derived growth factor-related protein 2 OS=Homo sapiens GN=HDGFRP2 PE=1 SV=1 | 8,76 | 10,02 | 9 | 16,1 |
| 1476 | sp|Q9NQ88|TIGAR_HUMAN | HUMAN | Fructose-2,6-bisphosphatase TIGAR OS=Homo sapiens GN=TIGAR PE=1 SV=1 | 8,76 | 9,42 | 7 | 33,33 |
| 1477 | sp|Q15642|CIP4_HUMAN | HUMAN | Cdc42-interacting protein 4 OS=Homo sapiens GN=TRIP10 PE=1 SV=3 | 8,75 | 9,06 | 5 | 10,82 |
| 1478 | sp|P51572|BAP31_HUMAN | HUMAN | B-cell receptor-associated protein 31 OS=Homo sapiens GN=BCAP31 PE=1 SV=3 | 8,73 | 8,82 | 4 | 15,04 |
| 1479 | sp|Q9BQ61|CS043_HUMAN | HUMAN | Uncharacterized protein C19orf43 OS=Homo sapiens GN=C19orf43 PE=1 SV=1 | 8,7 | 8,84 | 5 | 34,66 |
| 1480 | sp|P37108|SRP14_HUMAN | HUMAN | Signal recognition particle 14 kDa protein OS=Homo sapiens GN=SRP14 PE=1 SV=2 | 8,68 | 8,73 | 6 | 33,09 |
| 1481 | sp|Q86X76|NIT1_HUMAN | HUMAN | Nitrilase homolog 1 OS=Homo sapiens GN=NIT1 PE=1 SV=2 | 8,66 | 8,9 | 5 | 20,8 |
| 1482 | sp|P46379|BAG6_HUMAN | HUMAN | Large proline-rich protein BAG6 OS=Homo sapiens GN=BAG6 PE=1 SV=2 | 8,66 | 8,77 | 5 | 8,569 |
| 1483 | sp|E9PAV3|NACAM_HUMAN | HUMAN | Nascent polypeptide-associated complex subunit alpha, muscle-specific form OS=Homo sapiens GN=NACA PE=1 SV=1 | 8,66 | 8,71 | 8 | 5,005 |
| 1484 | sp|P80511|S10AC_HUMAN | HUMAN | Protein S100-A12 OS=Homo sapiens GN=S100A12 PE=1 SV=2 | 8,66 | 8,7 | 7 | 40,22 |
| 1485 | sp|Q96QR8|PURB_HUMAN | HUMAN | Transcriptional activator protein Pur-beta OS=Homo sapiens GN=PURB PE=1 SV=3 | 8,62 | 10,95 | 9 | 33,97 |
| 1486 | sp|Q99598|TSNAX_HUMAN | HUMAN | Translin-associated protein X OS=Homo sapiens GN=TSNAX PE=1 SV=1 | 8,62 | 9,38 | 6 | 28,97 |
| 1487 | sp|P35269|T2FA_HUMAN | HUMAN | General transcription factor IIF subunit 1 OS=Homo sapiens GN=GTF2F1 PE=1 SV=2 | 8,61 | 9,5 | 6 | 16,63 |
| 1488 | sp|Q9UH65|SWP70_HUMAN | HUMAN | Switch-associated protein 70 OS=Homo sapiens GN=SWAP70 PE=1 SV=1 | 8,61 | 8,89 | 6 | 12,31 |
| 1489 | sp|P13645|K1C10_HUMAN | HUMAN | Keratin, type I cytoskeletal 10 OS=Homo sapiens GN=KRT10 PE=1 SV=6 | 8,6 | 14,86 | 13 | 20,72 |
| 1490 | sp|P01034|CYTC_HUMAN | HUMAN | Cystatin-C OS=Homo sapiens GN=CST3 PE=1 SV=1 | 8,57 | 8,65 | 5 | 36,99 |
| 1491 | sp|P35573|GDE_HUMAN | HUMAN | Glycogen debranching enzyme OS=Homo sapiens GN=AGL PE=1 SV=3 | 8,56 | 8,72 | 4 | 4,178 |
| 1492 | sp|P50135|HNMT_HUMAN | HUMAN | Histamine N-methyltransferase OS=Homo sapiens GN=HNMT PE=1 SV=1 | 8,54 | 8,68 | 6 | 27,4 |
| 1493 | sp|P18065|IBP2_HUMAN | HUMAN | Insulin-like growth factor-binding protein 2 OS=Homo sapiens GN=IGFBP2 PE=1 SV=2 | 8,54 | 8,62 | 5 | 22,46 |
| 1494 | sp|O00754|MA2B1_HUMAN | HUMAN | Lysosomal alpha-mannosidase OS=Homo sapiens GN=MAN2B1 PE=1 SV=3 | 8,53 | 8,95 | 6 | 6,33 |
| 1495 | sp|Q15418|KS6A1_HUMAN | HUMAN | Ribosomal protein S6 kinase alpha-1 OS=Homo sapiens GN=RPS6KA1 PE=1 SV=2 | 8,52 | 15,43 | 9 | 16,05 |
| 1496 | sp|Q9H488|OFUT1_HUMAN | HUMAN | GDP-fucose protein O-fucosyltransferase 1 OS=Homo sapiens GN=POFUT1 PE=1 SV=1 | 8,51 | 8,6 | 6 | 23,71 |
| 1497 | sp|P24539|AT5F1_HUMAN | HUMAN | ATP synthase F(0) complex subunit B1, mitochondrial OS=Homo sapiens GN=ATP5F1 PE=1 SV=2 | 8,5 | 8,64 | 4 | 17,97 |
| 1498 | sp|Q5TDH0|DDI2_HUMAN | HUMAN | Protein DDI1 homolog 2 OS=Homo sapiens GN=DDI2 PE=1 SV=1 | 8,48 | 8,61 | 5 | 15,29 |
| 1499 | sp|Q15369|ELOC_HUMAN | HUMAN | Transcription elongation factor B polypeptide 1 OS=Homo sapiens GN=TCEB1 PE=1 SV=1 | 8,48 | 8,51 | 6 | 50 |
| 1500 | sp|Q9BRT3|MIEN1_HUMAN | HUMAN | Migration and invasion enhancer 1 OS=Homo sapiens GN=MIEN1 PE=1 SV=1 | 8,46 | 8,75 | 6 | 64,35 |
| 1501 | sp|P62266|RS23_HUMAN | HUMAN | 40S ribosomal protein S23 OS=Homo sapiens GN=RPS23 PE=1 SV=3 | 8,46 | 8,54 | 5 | 47,55 |
| 1502 | sp|Q9NYF8|BCLF1_HUMAN | HUMAN | Bcl-2-associated transcription factor 1 OS=Homo sapiens GN=BCLAF1 PE=1 SV=2 | 8,45 | 9,99 | 8 | 8,696 |
| 1503 | sp|Q96I24|FUBP3_HUMAN | HUMAN | Far upstream element-binding protein 3 OS=Homo sapiens GN=FUBP3 PE=1 SV=2 | 8,43 | 16,92 | 9 | 17,66 |
| 1504 | sp|Q96PK6|RBM14_HUMAN | HUMAN | RNA-binding protein 14 OS=Homo sapiens GN=RBM14 PE=1 SV=2 | 8,43 | 8,61 | 5 | 10,76 |
| 1505 | sp|P78356|PI42B_HUMAN | HUMAN | Phosphatidylinositol 5-phosphate 4-kinase type-2 beta OS=Homo sapiens GN=PIP4K2B PE=1 SV=1 | 8,43 | 8,55 | 5 | 16,11 |
| 1506 | sp|Q9UHV9|PFD2_HUMAN | HUMAN | Prefoldin subunit 2 OS=Homo sapiens GN=PFDN2 PE=1 SV=1 | 8,43 | 8,53 | 6 | 32,47 |
| 1507 | sp|Q14232|EI2BA_HUMAN | HUMAN | Translation initiation factor eIF-2B subunit alpha OS=Homo sapiens GN=EIF2B1 PE=1 SV=1 | 8,42 | 8,65 | 7 | 22,95 |
| 1508 | sp|P48047|ATPO_HUMAN | HUMAN | ATP synthase subunit O, mitochondrial OS=Homo sapiens GN=ATP5O PE=1 SV=1 | 8,42 | 8,46 | 5 | 30,05 |
| 1509 | sp|P34949|MPI_HUMAN | HUMAN | Mannose-6-phosphate isomerase OS=Homo sapiens GN=MPI PE=1 SV=2 | 8,41 | 8,59 | 7 | 25,06 |
| 1510 | sp|P41240|CSK_HUMAN | HUMAN | Tyrosine-protein kinase CSK OS=Homo sapiens GN=CSK PE=1 SV=1 | 8,41 | 8,52 | 5 | 14,67 |
| 1511 | sp|Q14677|EPN4_HUMAN | HUMAN | Clathrin interactor 1 OS=Homo sapiens GN=CLINT1 PE=1 SV=1 | 8,39 | 8,73 | 6 | 12,48 |
| 1512 | sp|P55957|BID_HUMAN | HUMAN | BH3-interacting domain death agonist OS=Homo sapiens GN=BID PE=1 SV=1 | 8,39 | 8,41 | 5 | 34,36 |
| 1513 | sp|P15090|FABP4_HUMAN | HUMAN | Fatty acid-binding protein, adipocyte OS=Homo sapiens GN=FABP4 PE=1 SV=3 | 8,37 | 8,97 | 9 | 53,03 |
| 1514 | sp|Q9Y333|LSM2_HUMAN | HUMAN | U6 snRNA-associated Sm-like protein LSm2 OS=Homo sapiens GN=LSM2 PE=1 SV=1 | 8,36 | 8,39 | 5 | 60 |
| 1515 | sp|Q03591|FHR1_HUMAN | HUMAN | Complement factor H-related protein 1 OS=Homo sapiens GN=CFHR1 PE=1 SV=2 | 8,34 | 13,75 | 9 | 35,15 |
| 1516 | sp|P30039|PBLD_HUMAN | HUMAN | Phenazine biosynthesis-like domain-containing protein OS=Homo sapiens GN=PBLD PE=1 SV=2 | 8,32 | 8,45 | 5 | 20,83 |
| 1517 | sp|Q13363|CTBP1_HUMAN | HUMAN | C-terminal-binding protein 1 OS=Homo sapiens GN=CTBP1 PE=1 SV=2 | 8,31 | 11,73 | 9 | 17,95 |
| 1518 | sp|Q99447|PCY2_HUMAN | HUMAN | Ethanolamine-phosphate cytidylyltransferase OS=Homo sapiens GN=PCYT2 PE=1 SV=1 | 8,31 | 8,46 | 4 | 13,62 |
| 1519 | sp|P61960|UFM1_HUMAN | HUMAN | Ubiquitin-fold modifier 1 OS=Homo sapiens GN=UFM1 PE=1 SV=1 | 8,29 | 8,35 | 6 | 68,24 |
| 1520 | sp|P84077|ARF1_HUMAN | HUMAN | ADP-ribosylation factor 1 OS=Homo sapiens GN=ARF1 PE=1 SV=2 | 8,28 | 18,48 | 30 | 74,59 |
| 1521 | sp|P08237|PFKAM_HUMAN | HUMAN | ATP-dependent 6-phosphofructokinase, muscle type OS=Homo sapiens GN=PFKM PE=1 SV=2 | 8,28 | 12,68 | 7 | 9,231 |
| 1522 | sp|Q9H3U1|UN45A_HUMAN | HUMAN | Protein unc-45 homolog A OS=Homo sapiens GN=UNC45A PE=1 SV=1 | 8,26 | 8,62 | 8 | 8,581 |
| 1523 | sp|O43813|LANC1_HUMAN | HUMAN | LanC-like protein 1 OS=Homo sapiens GN=LANCL1 PE=1 SV=1 | 8,26 | 8,44 | 10 | 20,55 |
| 1524 | sp|P49913|CAMP_HUMAN | HUMAN | Cathelicidin antimicrobial peptide OS=Homo sapiens GN=CAMP PE=1 SV=1 | 8,25 | 8,33 | 5 | 22,35 |
| 1525 | sp|P61916|NPC2_HUMAN | HUMAN | Epididymal secretory protein E1 OS=Homo sapiens GN=NPC2 PE=1 SV=1 | 8,24 | 8,29 | 5 | 38,41 |
| 1526 | sp|P63220|RS21_HUMAN | HUMAN | 40S ribosomal protein S21 OS=Homo sapiens GN=RPS21 PE=1 SV=1 | 8,24 | 8,28 | 13 | 59,04 |
| 1527 | sp|P50225|ST1A1_HUMAN | HUMAN | Sulfotransferase 1A1 OS=Homo sapiens GN=SULT1A1 PE=1 SV=3 | 8,23 | 20,49 | 11 | 43,73 |
| 1528 | sp|Q9Y6I3|EPN1_HUMAN | HUMAN | Epsin-1 OS=Homo sapiens GN=EPN1 PE=1 SV=2 | 8,23 | 8,36 | 5 | 15,63 |
| 1529 | sp|Q96EK6|GNA1_HUMAN | HUMAN | Glucosamine 6-phosphate N-acetyltransferase OS=Homo sapiens GN=GNPNAT1 PE=1 SV=1 | 8,23 | 8,25 | 4 | 29,35 |
| 1530 | sp|Q7Z739|YTHD3_HUMAN | HUMAN | YTH domain-containing family protein 3 OS=Homo sapiens GN=YTHDF3 PE=1 SV=1 | 8,22 | 8,8 | 8 | 16,07 |
| 1531 | sp|P14174|MIF_HUMAN | HUMAN | Macrophage migration inhibitory factor OS=Homo sapiens GN=MIF PE=1 SV=4 | 8,21 | 8,23 | 18 | 83,48 |
| 1532 | sp|O00170|AIP_HUMAN | HUMAN | AH receptor-interacting protein OS=Homo sapiens GN=AIP PE=1 SV=2 | 8,19 | 8,36 | 4 | 17,27 |
| 1533 | sp|P49959|MRE11_HUMAN | HUMAN | Double-strand break repair protein MRE11A OS=Homo sapiens GN=MRE11A PE=1 SV=3 | 8,18 | 8,76 | 5 | 7,768 |
| 1534 | sp|Q9BPX5|ARP5L_HUMAN | HUMAN | Actin-related protein 2/3 complex subunit 5-like protein OS=Homo sapiens GN=ARPC5L PE=1 SV=1 | 8,17 | 10,21 | 8 | 58,82 |
| 1535 | sp|P01860|IGHG3_HUMAN | HUMAN | Ig gamma-3 chain C region OS=Homo sapiens GN=IGHG3 PE=1 SV=2 | 8,16 | 35,55 | 55 | 75,86 |
| 1536 | sp|Q92896|GSLG1_HUMAN | HUMAN | Golgi apparatus protein 1 OS=Homo sapiens GN=GLG1 PE=1 SV=2 | 8,16 | 8,33 | 4 | 4,835 |
| 1537 | sp|P29536|LMOD1_HUMAN | HUMAN | Leiomodin-1 OS=Homo sapiens GN=LMOD1 PE=1 SV=3 | 8,16 | 8,3 | 5 | 10 |
| 1538 | sp|O14561|ACPM_HUMAN | HUMAN | Acyl carrier protein, mitochondrial OS=Homo sapiens GN=NDUFAB1 PE=1 SV=3 | 8,15 | 8,28 | 6 | 33,97 |
| 1539 | sp|P61513|RL37A_HUMAN | HUMAN | 60S ribosomal protein L37a OS=Homo sapiens GN=RPL37A PE=1 SV=2 | 8,15 | 8,23 | 5 | 51,09 |
| 1540 | sp|Q8IZP0|ABI1_HUMAN | HUMAN | Abl interactor 1 OS=Homo sapiens GN=ABI1 PE=1 SV=4 | 8,14 | 8,24 | 5 | 13,58 |
| 1541 | sp|P52594|AGFG1_HUMAN | HUMAN | Arf-GAP domain and FG repeat-containing protein 1 OS=Homo sapiens GN=AGFG1 PE=1 SV=2 | 8,13 | 8,68 | 5 | 15,48 |
| 1542 | sp|O14776|TCRG1_HUMAN | HUMAN | Transcription elongation regulator 1 OS=Homo sapiens GN=TCERG1 PE=1 SV=2 | 8,12 | 8,39 | 5 | 6,557 |
| 1543 | sp|O60825|F262_HUMAN | HUMAN | 6-phosphofructo-2-kinase/fructose-2,6-bisphosphatase 2 OS=Homo sapiens GN=PFKFB2 PE=1 SV=2 | 8,11 | 8,32 | 5 | 10,1 |
| 1544 | sp|P49458|SRP09_HUMAN | HUMAN | Signal recognition particle 9 kDa protein OS=Homo sapiens GN=SRP9 PE=1 SV=2 | 8,11 | 8,13 | 6 | 45,35 |
| 1545 | sp|P13674|P4HA1_HUMAN | HUMAN | Prolyl 4-hydroxylase subunit alpha-1 OS=Homo sapiens GN=P4HA1 PE=1 SV=2 | 8,1 | 8,18 | 4 | 10,11 |
| 1546 | sp|P35268|RL22_HUMAN | HUMAN | 60S ribosomal protein L22 OS=Homo sapiens GN=RPL22 PE=1 SV=2 | 8,1 | 8,11 | 13 | 50,78 |
| 1547 | sp|P55039|DRG2_HUMAN | HUMAN | Developmentally-regulated GTP-binding protein 2 OS=Homo sapiens GN=DRG2 PE=1 SV=1 | 8,07 | 8,3 | 4 | 14,56 |
| 1548 | sp|Q68EM7|RHG17_HUMAN | HUMAN | Rho GTPase-activating protein 17 OS=Homo sapiens GN=ARHGAP17 PE=1 SV=1 | 8,07 | 8,19 | 5 | 7,378 |
| 1549 | sp|Q14244|MAP7_HUMAN | HUMAN | Ensconsin OS=Homo sapiens GN=MAP7 PE=1 SV=1 | 8,06 | 8,64 | 6 | 7,877 |
| 1550 | sp|Q9BVA1|TBB2B_HUMAN | HUMAN | Tubulin beta-2B chain OS=Homo sapiens GN=TUBB2B PE=1 SV=1 | 8,04 | 55,55 | 94 | 80,9 |
| 1551 | sp|Q92599|SEPT8_HUMAN | HUMAN | Septin-8 OS=Homo sapiens GN=SEPT8 PE=1 SV=4 | 8,04 | 13,4 | 10 | 24,84 |
| 1552 | sp|Q06141|REG3A_HUMAN | HUMAN | Regenerating islet-derived protein 3-alpha OS=Homo sapiens GN=REG3A PE=1 SV=1 | 8,04 | 8,15 | 5 | 36,57 |
| 1553 | sp|P42766|RL35_HUMAN | HUMAN | 60S ribosomal protein L35 OS=Homo sapiens GN=RPL35 PE=1 SV=2 | 8,03 | 8,37 | 8 | 32,52 |
| 1554 | sp|Q9Y3U8|RL36_HUMAN | HUMAN | 60S ribosomal protein L36 OS=Homo sapiens GN=RPL36 PE=1 SV=3 | 8,03 | 8,11 | 4 | 30,48 |
| 1555 | sp|Q8WWY3|PRP31_HUMAN | HUMAN | U4/U6 small nuclear ribonucleoprotein Prp31 OS=Homo sapiens GN=PRPF31 PE=1 SV=2 | 8,02 | 8,16 | 5 | 15,83 |
| 1556 | sp|Q8IV56|PRR15_HUMAN | HUMAN | Proline-rich protein 15 OS=Homo sapiens GN=PRR15 PE=2 SV=1 | 8,02 | 8,03 | 4 | 58,91 |
| 1557 | sp|Q99627|CSN8_HUMAN | HUMAN | COP9 signalosome complex subunit 8 OS=Homo sapiens GN=COPS8 PE=1 SV=1 | 8,02 | 8,02 | 5 | 36,36 |
| 1558 | sp|Q96C90|PP14B_HUMAN | HUMAN | Protein phosphatase 1 regulatory subunit 14B OS=Homo sapiens GN=PPP1R14B PE=1 SV=3 | 8,02 | 8,02 | 5 | 48,3 |
| 1559 | sp|P01877|IGHA2_HUMAN | HUMAN | Ig alpha-2 chain C region OS=Homo sapiens GN=IGHA2 PE=1 SV=3 | 8,01 | 31,51 | 71 | 71,18 |
| 1560 | sp|Q9Y5L4|TIM13_HUMAN | HUMAN | Mitochondrial import inner membrane translocase subunit Tim13 OS=Homo sapiens GN=TIMM13 PE=1 SV=1 | 8,01 | 8,01 | 5 | 69,47 |
| 1561 | sp|Q92878|RAD50_HUMAN | HUMAN | DNA repair protein RAD50 OS=Homo sapiens GN=RAD50 PE=1 SV=1 | 8 | 9,04 | 4 | 3,811 |
| 1562 | sp|P08195|4F2_HUMAN | HUMAN | 4F2 cell-surface antigen heavy chain OS=Homo sapiens GN=SLC3A2 PE=1 SV=3 | 8 | 8 | 4 | 9,524 |
| 1563 | sp|Q9H8S9|MOB1A_HUMAN | HUMAN | MOB kinase activator 1A OS=Homo sapiens GN=MOB1A PE=1 SV=4 | 8 | 8 | 4 | 19,44 |
| 1564 | sp|P20962|PTMS_HUMAN | HUMAN | Parathymosin OS=Homo sapiens GN=PTMS PE=1 SV=2 | 8 | 8 | 6 | 23,53 |
| 1565 | sp|Q9P0L0|VAPA_HUMAN | HUMAN | Vesicle-associated membrane protein-associated protein A OS=Homo sapiens GN=VAPA PE=1 SV=3 | 8 | 8 | 4 | 15,66 |
| 1566 | sp|P04216|THY1_HUMAN | HUMAN | Thy-1 membrane glycoprotein OS=Homo sapiens GN=THY1 PE=1 SV=2 | 8 | 8 | 4 | 24,84 |
| 1567 | sp|Q9UN86|G3BP2_HUMAN | HUMAN | Ras GTPase-activating protein-binding protein 2 OS=Homo sapiens GN=G3BP2 PE=1 SV=2 | 7,98 | 10,18 | 6 | 13,49 |
| 1568 | sp|P14854|CX6B1_HUMAN | HUMAN | Cytochrome c oxidase subunit 6B1 OS=Homo sapiens GN=COX6B1 PE=1 SV=2 | 7,97 | 8,08 | 6 | 66,28 |
| 1569 | sp|P16144|ITB4_HUMAN | HUMAN | Integrin beta-4 OS=Homo sapiens GN=ITGB4 PE=1 SV=5 | 7,96 | 8,4 | 8 | 5,104 |
| 1570 | sp|Q9GZP4|PITH1_HUMAN | HUMAN | PITH domain-containing protein 1 OS=Homo sapiens GN=PITHD1 PE=1 SV=1 | 7,96 | 8,21 | 6 | 30,81 |
| 1571 | sp|P10619|PPGB_HUMAN | HUMAN | Lysosomal protective protein OS=Homo sapiens GN=CTSA PE=1 SV=2 | 7,95 | 8,06 | 6 | 14,37 |
| 1572 | sp|O75494|SRS10_HUMAN | HUMAN | Serine/arginine-rich splicing factor 10 OS=Homo sapiens GN=SRSF10 PE=1 SV=1 | 7,95 | 8,04 | 5 | 19,47 |
| 1573 | sp|Q9HB40|RISC_HUMAN | HUMAN | Retinoid-inducible serine carboxypeptidase OS=Homo sapiens GN=SCPEP1 PE=1 SV=1 | 7,94 | 7,98 | 4 | 8,186 |
| 1574 | sp|Q66K74|MAP1S_HUMAN | HUMAN | Microtubule-associated protein 1S OS=Homo sapiens GN=MAP1S PE=1 SV=2 | 7,93 | 8,22 | 5 | 6,799 |
| 1575 | sp|P05062|ALDOB_HUMAN | HUMAN | Fructose-bisphosphate aldolase B OS=Homo sapiens GN=ALDOB PE=1 SV=2 | 7,92 | 11,56 | 9 | 26,37 |
| 1576 | sp|P09497|CLCB_HUMAN | HUMAN | Clathrin light chain B OS=Homo sapiens GN=CLTB PE=1 SV=1 | 7,92 | 8,31 | 8 | 23,58 |
| 1577 | sp|Q96AB3|ISOC2_HUMAN | HUMAN | Isochorismatase domain-containing protein 2 OS=Homo sapiens GN=ISOC2 PE=1 SV=1 | 7,92 | 8,04 | 4 | 42,93 |
| 1578 | sp|Q8WW12|PCNP_HUMAN | HUMAN | PEST proteolytic signal-containing nuclear protein OS=Homo sapiens GN=PCNP PE=1 SV=2 | 7,91 | 8,05 | 4 | 29,78 |
| 1579 | sp|O00487|PSDE_HUMAN | HUMAN | 26S proteasome non-ATPase regulatory subunit 14 OS=Homo sapiens GN=PSMD14 PE=1 SV=1 | 7,9 | 8 | 5 | 22,26 |
| 1580 | sp|Q13217|DNJC3_HUMAN | HUMAN | DnaJ homolog subfamily C member 3 OS=Homo sapiens GN=DNAJC3 PE=1 SV=1 | 7,89 | 8,45 | 5 | 15,08 |
| 1581 | sp|O15264|MK13_HUMAN | HUMAN | Mitogen-activated protein kinase 13 OS=Homo sapiens GN=MAPK13 PE=1 SV=1 | 7,87 | 10,54 | 7 | 20,27 |
| 1582 | sp|P04181|OAT_HUMAN | HUMAN | Ornithine aminotransferase, mitochondrial OS=Homo sapiens GN=OAT PE=1 SV=1 | 7,85 | 8,01 | 5 | 13,44 |
| 1583 | sp|Q99436|PSB7_HUMAN | HUMAN | Proteasome subunit beta type-7 OS=Homo sapiens GN=PSMB7 PE=1 SV=1 | 7,85 | 7,91 | 6 | 20,22 |
| 1584 | sp|Q9UHD9|UBQL2_HUMAN | HUMAN | Ubiquilin-2 OS=Homo sapiens GN=UBQLN2 PE=1 SV=2 | 7,83 | 16,03 | 10 | 20,51 |
| 1585 | sp|Q9NUP9|LIN7C_HUMAN | HUMAN | Protein lin-7 homolog C OS=Homo sapiens GN=LIN7C PE=1 SV=1 | 7,83 | 8,01 | 5 | 29,44 |
| 1586 | sp|O15027|SC16A_HUMAN | HUMAN | Protein transport protein Sec16A OS=Homo sapiens GN=SEC16A PE=1 SV=3 | 7,8 | 8,03 | 5 | 2,524 |
| 1587 | sp|Q9H3G5|CPVL_HUMAN | HUMAN | Probable serine carboxypeptidase CPVL OS=Homo sapiens GN=CPVL PE=1 SV=2 | 7,8 | 7,87 | 4 | 9,034 |
| 1588 | sp|Q96JB5|CK5P3_HUMAN | HUMAN | CDK5 regulatory subunit-associated protein 3 OS=Homo sapiens GN=CDK5RAP3 PE=1 SV=2 | 7,79 | 11,73 | 7 | 13,24 |
| 1589 | sp|Q8NBJ5|GT251_HUMAN | HUMAN | Procollagen galactosyltransferase 1 OS=Homo sapiens GN=COLGALT1 PE=1 SV=1 | 7,79 | 8 | 4 | 7,556 |
| 1590 | sp|P35244|RFA3_HUMAN | HUMAN | Replication protein A 14 kDa subunit OS=Homo sapiens GN=RPA3 PE=1 SV=1 | 7,77 | 7,97 | 4 | 51,24 |
| 1591 | sp|P49247|RPIA_HUMAN | HUMAN | Ribose-5-phosphate isomerase OS=Homo sapiens GN=RPIA PE=1 SV=3 | 7,76 | 8 | 6 | 25,4 |
| 1592 | sp|Q04323|UBXN1_HUMAN | HUMAN | UBX domain-containing protein 1 OS=Homo sapiens GN=UBXN1 PE=1 SV=2 | 7,75 | 7,88 | 5 | 25,93 |
| 1593 | sp|Q9NZD2|GLTP_HUMAN | HUMAN | Glycolipid transfer protein OS=Homo sapiens GN=GLTP PE=1 SV=3 | 7,74 | 7,83 | 6 | 27,75 |
| 1594 | sp|O75935|DCTN3_HUMAN | HUMAN | Dynactin subunit 3 OS=Homo sapiens GN=DCTN3 PE=1 SV=1 | 7,73 | 8,42 | 5 | 30,65 |
| 1595 | sp|P55809|SCOT1_HUMAN | HUMAN | Succinyl-CoA:3-ketoacid coenzyme A transferase 1, mitochondrial OS=Homo sapiens GN=OXCT1 PE=1 SV=1 | 7,72 | 7,88 | 4 | 13,85 |
| 1596 | sp|P40222|TXLNA_HUMAN | HUMAN | Alpha-taxilin OS=Homo sapiens GN=TXLNA PE=1 SV=3 | 7,7 | 7,94 | 5 | 11,72 |
| 1597 | sp|Q9NZU5|LMCD1_HUMAN | HUMAN | LIM and cysteine-rich domains protein 1 OS=Homo sapiens GN=LMCD1 PE=1 SV=1 | 7,7 | 7,83 | 4 | 15,07 |
| 1598 | sp|P08185|CBG_HUMAN | HUMAN | Corticosteroid-binding globulin OS=Homo sapiens GN=SERPINA6 PE=1 SV=1 | 7,68 | 7,75 | 4 | 11,6 |
| 1599 | sp|P15153|RAC2_HUMAN | HUMAN | Ras-related C3 botulinum toxin substrate 2 OS=Homo sapiens GN=RAC2 PE=1 SV=1 | 7,67 | 18,29 | 12 | 57,81 |
| 1600 | sp|Q16539|MK14_HUMAN | HUMAN | Mitogen-activated protein kinase 14 OS=Homo sapiens GN=MAPK14 PE=1 SV=3 | 7,66 | 9,96 | 6 | 19,17 |
| 1601 | sp|Q96IZ0|PAWR_HUMAN | HUMAN | PRKC apoptosis WT1 regulator protein OS=Homo sapiens GN=PAWR PE=1 SV=1 | 7,62 | 7,69 | 4 | 22,65 |
| 1602 | sp|Q9NQP4|PFD4_HUMAN | HUMAN | Prefoldin subunit 4 OS=Homo sapiens GN=PFDN4 PE=1 SV=1 | 7,62 | 7,68 | 4 | 29,1 |
| 1603 | sp|Q9H299|SH3L3_HUMAN | HUMAN | SH3 domain-binding glutamic acid-rich-like protein 3 OS=Homo sapiens GN=SH3BGRL3 PE=1 SV=1 | 7,62 | 7,68 | 6 | 39,78 |
| 1604 | sp|P22352|GPX3_HUMAN | HUMAN | Glutathione peroxidase 3 OS=Homo sapiens GN=GPX3 PE=1 SV=2 | 7,59 | 7,69 | 5 | 28,32 |
| 1605 | sp|P46776|RL27A_HUMAN | HUMAN | 60S ribosomal protein L27a OS=Homo sapiens GN=RPL27A PE=1 SV=2 | 7,57 | 7,65 | 7 | 34,46 |
| 1606 | sp|Q8TAT6|NPL4_HUMAN | HUMAN | Nuclear protein localization protein 4 homolog OS=Homo sapiens GN=NPLOC4 PE=1 SV=3 | 7,55 | 8,14 | 6 | 10,2 |
| 1607 | sp|Q5H9R7|PP6R3_HUMAN | HUMAN | Serine/threonine-protein phosphatase 6 regulatory subunit 3 OS=Homo sapiens GN=PPP6R3 PE=1 SV=2 | 7,53 | 7,84 | 6 | 8,935 |
| 1608 | sp|P28074|PSB5_HUMAN | HUMAN | Proteasome subunit beta type-5 OS=Homo sapiens GN=PSMB5 PE=1 SV=3 | 7,53 | 7,71 | 7 | 22,81 |
| 1609 | sp|Q9H2G2|SLK_HUMAN | HUMAN | STE20-like serine/threonine-protein kinase OS=Homo sapiens GN=SLK PE=1 SV=1 | 7,52 | 7,82 | 7 | 5,749 |
| 1610 | sp|P07360|CO8G_HUMAN | HUMAN | Complement component C8 gamma chain OS=Homo sapiens GN=C8G PE=1 SV=3 | 7,52 | 7,74 | 5 | 34,16 |
| 1611 | sp|Q9H008|LHPP_HUMAN | HUMAN | Phospholysine phosphohistidine inorganic pyrophosphate phosphatase OS=Homo sapiens GN=LHPP PE=1 SV=2 | 7,52 | 7,58 | 4 | 30 |
| 1612 | sp|O75367|H2AY_HUMAN | HUMAN | Core histone macro-H2A.1 OS=Homo sapiens GN=H2AFY PE=1 SV=4 | 7,51 | 7,66 | 4 | 16,94 |
| 1613 | sp|Q9BQ69|MACD1_HUMAN | HUMAN | O-acetyl-ADP-ribose deacetylase MACROD1 OS=Homo sapiens GN=MACROD1 PE=1 SV=2 | 7,51 | 7,66 | 4 | 17,85 |
| 1614 | sp|Q96FQ6|S10AG_HUMAN | HUMAN | Protein S100-A16 OS=Homo sapiens GN=S100A16 PE=1 SV=1 | 7,5 | 7,54 | 4 | 44,66 |
| 1615 | sp|Q96PD5|PGRP2_HUMAN | HUMAN | N-acetylmuramoyl-L-alanine amidase OS=Homo sapiens GN=PGLYRP2 PE=1 SV=1 | 7,49 | 7,63 | 4 | 10,24 |
| 1616 | sp|Q14683|SMC1A_HUMAN | HUMAN | Structural maintenance of chromosomes protein 1A OS=Homo sapiens GN=SMC1A PE=1 SV=2 | 7,48 | 8,38 | 5 | 4,217 |
| 1617 | sp|Q7L5N1|CSN6_HUMAN | HUMAN | COP9 signalosome complex subunit 6 OS=Homo sapiens GN=COPS6 PE=1 SV=1 | 7,46 | 7,72 | 5 | 16,82 |
| 1618 | sp|P61769|B2MG_HUMAN | HUMAN | Beta-2-microglobulin OS=Homo sapiens GN=B2M PE=1 SV=1 | 7,46 | 7,54 | 8 | 52,94 |
| 1619 | sp|Q96CT7|CC124_HUMAN | HUMAN | Coiled-coil domain-containing protein 124 OS=Homo sapiens GN=CCDC124 PE=1 SV=1 | 7,43 | 7,56 | 4 | 20,18 |
| 1620 | sp|P29622|KAIN_HUMAN | HUMAN | Kallistatin OS=Homo sapiens GN=SERPINA4 PE=1 SV=3 | 7,42 | 8,11 | 5 | 13,82 |
| 1621 | sp|P53041|PPP5_HUMAN | HUMAN | Serine/threonine-protein phosphatase 5 OS=Homo sapiens GN=PPP5C PE=1 SV=1 | 7,42 | 7,52 | 5 | 13,23 |
| 1622 | sp|Q13232|NDK3_HUMAN | HUMAN | Nucleoside diphosphate kinase 3 OS=Homo sapiens GN=NME3 PE=1 SV=2 | 7,41 | 7,6 | 5 | 34,91 |
| 1623 | sp|Q96CN7|ISOC1_HUMAN | HUMAN | Isochorismatase domain-containing protein 1 OS=Homo sapiens GN=ISOC1 PE=1 SV=3 | 7,39 | 7,51 | 4 | 17,79 |
| 1624 | sp|Q9UJW0|DCTN4_HUMAN | HUMAN | Dynactin subunit 4 OS=Homo sapiens GN=DCTN4 PE=1 SV=1 | 7,36 | 7,58 | 5 | 19,13 |
| 1625 | sp|Q9BWF3|RBM4_HUMAN | HUMAN | RNA-binding protein 4 OS=Homo sapiens GN=RBM4 PE=1 SV=1 | 7,36 | 7,53 | 4 | 17,03 |
| 1626 | sp|P06313|KV403_HUMAN | HUMAN | Ig kappa chain V-IV region JI OS=Homo sapiens PE=4 SV=1 | 7,36 | 7,53 | 9 | 41,35 |
| 1627 | sp|Q05209|PTN12_HUMAN | HUMAN | Tyrosine-protein phosphatase non-receptor type 12 OS=Homo sapiens GN=PTPN12 PE=1 SV=3 | 7,34 | 7,65 | 5 | 9,103 |
| 1628 | sp|Q9BRG1|VPS25_HUMAN | HUMAN | Vacuolar protein-sorting-associated protein 25 OS=Homo sapiens GN=VPS25 PE=1 SV=1 | 7,34 | 7,5 | 5 | 27,84 |
| 1629 | sp|Q08945|SSRP1_HUMAN | HUMAN | FACT complex subunit SSRP1 OS=Homo sapiens GN=SSRP1 PE=1 SV=1 | 7,31 | 7,58 | 5 | 9,873 |
| 1630 | sp|Q5VZK9|CARL1_HUMAN | HUMAN | F-actin-uncapping protein LRRC16A OS=Homo sapiens GN=LRRC16A PE=1 SV=1 | 7,3 | 7,68 | 5 | 4,595 |
| 1631 | sp|P05090|APOD_HUMAN | HUMAN | Apolipoprotein D OS=Homo sapiens GN=APOD PE=1 SV=1 | 7,3 | 7,52 | 6 | 28,57 |
| 1632 | sp|Q14008|CKAP5_HUMAN | HUMAN | Cytoskeleton-associated protein 5 OS=Homo sapiens GN=CKAP5 PE=1 SV=3 | 7,29 | 7,86 | 6 | 3,248 |
| 1633 | sp|Q9Y3C6|PPIL1_HUMAN | HUMAN | Peptidyl-prolyl cis-trans isomerase-like 1 OS=Homo sapiens GN=PPIL1 PE=1 SV=1 | 7,29 | 7,46 | 4 | 31,33 |
| 1634 | sp|P08243|ASNS_HUMAN | HUMAN | Asparagine synthetase [glutamine-hydrolyzing] OS=Homo sapiens GN=ASNS PE=1 SV=4 | 7,28 | 8,36 | 6 | 12,48 |
| 1635 | sp|O14936|CSKP_HUMAN | HUMAN | Peripheral plasma membrane protein CASK OS=Homo sapiens GN=CASK PE=1 SV=3 | 7,26 | 7,6 | 6 | 7,127 |
| 1636 | sp|O00505|IMA4_HUMAN | HUMAN | Importin subunit alpha-4 OS=Homo sapiens GN=KPNA3 PE=1 SV=2 | 7,24 | 14,07 | 8 | 22,46 |
| 1637 | sp|Q14005|IL16_HUMAN | HUMAN | Pro-interleukin-16 OS=Homo sapiens GN=IL16 PE=1 SV=4 | 7,24 | 7,54 | 6 | 7,282 |
| 1638 | sp|P21397|AOFA_HUMAN | HUMAN | Amine oxidase [flavin-containing] A OS=Homo sapiens GN=MAOA PE=1 SV=1 | 7,23 | 7,38 | 5 | 10,06 |
| 1639 | sp|Q92890|UFD1_HUMAN | HUMAN | Ubiquitin fusion degradation protein 1 homolog OS=Homo sapiens GN=UFD1L PE=1 SV=3 | 7,22 | 7,45 | 4 | 17,26 |
| 1640 | sp|Q6P1N0|C2D1A_HUMAN | HUMAN | Coiled-coil and C2 domain-containing protein 1A OS=Homo sapiens GN=CC2D1A PE=1 SV=1 | 7,22 | 7,34 | 4 | 5,573 |
| 1641 | sp|P54619|AAKG1_HUMAN | HUMAN | 5'-AMP-activated protein kinase subunit gamma-1 OS=Homo sapiens GN=PRKAG1 PE=1 SV=1 | 7,22 | 7,3 | 4 | 14,8 |
| 1642 | sp|P07942|LAMB1_HUMAN | HUMAN | Laminin subunit beta-1 OS=Homo sapiens GN=LAMB1 PE=1 SV=2 | 7,21 | 7,79 | 6 | 3,751 |
| 1643 | sp|P52758|UK114_HUMAN | HUMAN | Ribonuclease UK114 OS=Homo sapiens GN=HRSP12 PE=1 SV=1 | 7,21 | 7,28 | 4 | 40,88 |
| 1644 | sp|P28072|PSB6_HUMAN | HUMAN | Proteasome subunit beta type-6 OS=Homo sapiens GN=PSMB6 PE=1 SV=4 | 7,2 | 7,44 | 7 | 20,08 |
| 1645 | sp|O43719|HTSF1_HUMAN | HUMAN | HIV Tat-specific factor 1 OS=Homo sapiens GN=HTATSF1 PE=1 SV=1 | 7,19 | 7,43 | 4 | 6,49 |
| 1646 | sp|Q9UDT6|CLIP2_HUMAN | HUMAN | CAP-Gly domain-containing linker protein 2 OS=Homo sapiens GN=CLIP2 PE=1 SV=1 | 7,18 | 7,75 | 5 | 6,692 |
| 1647 | sp|Q16527|CSRP2_HUMAN | HUMAN | Cysteine and glycine-rich protein 2 OS=Homo sapiens GN=CSRP2 PE=1 SV=3 | 7,18 | 7,28 | 4 | 25,91 |
| 1648 | sp|O00391|QSOX1_HUMAN | HUMAN | Sulfhydryl oxidase 1 OS=Homo sapiens GN=QSOX1 PE=1 SV=3 | 7,16 | 7,99 | 5 | 8,835 |
| 1649 | sp|Q9UBC2|EP15R_HUMAN | HUMAN | Epidermal growth factor receptor substrate 15-like 1 OS=Homo sapiens GN=EPS15L1 PE=1 SV=1 | 7,16 | 7,56 | 6 | 8,102 |
| 1650 | sp|Q9H9E3|COG4_HUMAN | HUMAN | Conserved oligomeric Golgi complex subunit 4 OS=Homo sapiens GN=COG4 PE=1 SV=3 | 7,15 | 7,45 | 5 | 8,662 |
| 1651 | sp|Q9H9Q2|CSN7B_HUMAN | HUMAN | COP9 signalosome complex subunit 7b OS=Homo sapiens GN=COPS7B PE=1 SV=1 | 7,15 | 7,25 | 4 | 25,38 |
| 1652 | sp|Q9UKX7|NUP50_HUMAN | HUMAN | Nuclear pore complex protein Nup50 OS=Homo sapiens GN=NUP50 PE=1 SV=2 | 7,15 | 7,24 | 4 | 15,17 |
| 1653 | sp|P22695|QCR2_HUMAN | HUMAN | Cytochrome b-c1 complex subunit 2, mitochondrial OS=Homo sapiens GN=UQCRC2 PE=1 SV=3 | 7,14 | 7,21 | 4 | 12,8 |
| 1654 | sp|A0AV96|RBM47_HUMAN | HUMAN | RNA-binding protein 47 OS=Homo sapiens GN=RBM47 PE=1 SV=2 | 7,13 | 7,2 | 5 | 12,31 |
| 1655 | sp|Q9BX68|HINT2_HUMAN | HUMAN | Histidine triad nucleotide-binding protein 2, mitochondrial OS=Homo sapiens GN=HINT2 PE=1 SV=1 | 7,12 | 7,29 | 4 | 41,72 |
| 1656 | sp|P0DJI9|SAA2_HUMAN | HUMAN | Serum amyloid A-2 protein OS=Homo sapiens GN=SAA2 PE=1 SV=1 | 7,12 | 7,17 | 4 | 31,97 |
| 1657 | sp|P27105|STOM_HUMAN | HUMAN | Erythrocyte band 7 integral membrane protein OS=Homo sapiens GN=STOM PE=1 SV=3 | 7,11 | 7,19 | 4 | 26,74 |
| 1658 | sp|P13073|COX41_HUMAN | HUMAN | Cytochrome c oxidase subunit 4 isoform 1, mitochondrial OS=Homo sapiens GN=COX4I1 PE=1 SV=1 | 7,1 | 7,25 | 4 | 23,67 |
| 1659 | sp|Q9H7C9|AAMDC_HUMAN | HUMAN | Mth938 domain-containing protein OS=Homo sapiens GN=AAMDC PE=1 SV=1 | 7,1 | 7,2 | 4 | 37,7 |
| 1660 | sp|P59666|DEF3_HUMAN | HUMAN | Neutrophil defensin 3 OS=Homo sapiens GN=DEFA3 PE=1 SV=1 | 7,1 | 7,15 | 4 | 26,6 |
| 1661 | sp|Q99805|TM9S2_HUMAN | HUMAN | Transmembrane 9 superfamily member 2 OS=Homo sapiens GN=TM9SF2 PE=1 SV=1 | 7,08 | 7,31 | 5 | 12,22 |
| 1662 | sp|Q96AT9|RPE_HUMAN | HUMAN | Ribulose-phosphate 3-epimerase OS=Homo sapiens GN=RPE PE=1 SV=1 | 7,08 | 7,24 | 5 | 24,56 |
| 1663 | sp|O00160|MYO1F_HUMAN | HUMAN | Unconventional myosin-If OS=Homo sapiens GN=MYO1F PE=1 SV=3 | 7,07 | 7,4 | 4 | 4,463 |
| 1664 | sp|Q9ULC3|RAB23_HUMAN | HUMAN | Ras-related protein Rab-23 OS=Homo sapiens GN=RAB23 PE=1 SV=1 | 7,07 | 7,34 | 4 | 25,74 |
| 1665 | sp|O43866|CD5L_HUMAN | HUMAN | CD5 antigen-like OS=Homo sapiens GN=CD5L PE=1 SV=1 | 7,07 | 7,22 | 4 | 13,26 |
| 1666 | sp|P63167|DYL1_HUMAN | HUMAN | Dynein light chain 1, cytoplasmic OS=Homo sapiens GN=DYNLL1 PE=1 SV=1 | 7,07 | 7,15 | 8 | 57,3 |
| 1667 | sp|P01764|HV303_HUMAN | HUMAN | Ig heavy chain V-III region 23 OS=Homo sapiens GN=IGHV3-23 PE=1 SV=2 | 7,06 | 7,1 | 12 | 35,04 |
| 1668 | sp|P55795|HNRH2_HUMAN | HUMAN | Heterogeneous nuclear ribonucleoprotein H2 OS=Homo sapiens GN=HNRNPH2 PE=1 SV=1 | 7,03 | 26,68 | 19 | 35,41 |
| 1669 | sp|Q92905|CSN5_HUMAN | HUMAN | COP9 signalosome complex subunit 5 OS=Homo sapiens GN=COPS5 PE=1 SV=4 | 7,03 | 7,53 | 5 | 14,67 |
| 1670 | sp|P49407|ARRB1_HUMAN | HUMAN | Beta-arrestin-1 OS=Homo sapiens GN=ARRB1 PE=1 SV=2 | 7,03 | 7,11 | 4 | 17,7 |
| 1671 | sp|Q9BSD7|NTPCR_HUMAN | HUMAN | Cancer-related nucleoside-triphosphatase OS=Homo sapiens GN=NTPCR PE=1 SV=1 | 7,02 | 7,26 | 6 | 37,37 |
| 1672 | sp|Q15555|MARE2_HUMAN | HUMAN | Microtubule-associated protein RP/EB family member 2 OS=Homo sapiens GN=MAPRE2 PE=1 SV=1 | 7,01 | 8,23 | 5 | 21,41 |
| 1673 | sp|Q96RS6|NUDC1_HUMAN | HUMAN | NudC domain-containing protein 1 OS=Homo sapiens GN=NUDCD1 PE=1 SV=2 | 7,01 | 7,19 | 5 | 10,81 |
| 1674 | sp|Q12959|DLG1_HUMAN | HUMAN | Disks large homolog 1 OS=Homo sapiens GN=DLG1 PE=1 SV=2 | 7 | 7,28 | 6 | 9,181 |
| 1675 | sp|P13671|CO6_HUMAN | HUMAN | Complement component C6 OS=Homo sapiens GN=C6 PE=1 SV=3 | 7 | 7,23 | 5 | 8,458 |
| 1676 | sp|P20073|ANXA7_HUMAN | HUMAN | Annexin A7 OS=Homo sapiens GN=ANXA7 PE=1 SV=3 | 6,98 | 7,19 | 5 | 12,7 |
| 1677 | sp|Q9UI30|TR112_HUMAN | HUMAN | Multifunctional methyltransferase subunit TRM112-like protein OS=Homo sapiens GN=TRMT112 PE=1 SV=1 | 6,98 | 7,11 | 7 | 42,4 |
| 1678 | sp|Q9Y5S9|RBM8A_HUMAN | HUMAN | RNA-binding protein 8A OS=Homo sapiens GN=RBM8A PE=1 SV=1 | 6,97 | 7,22 | 8 | 51,72 |
| 1679 | sp|O60684|IMA7_HUMAN | HUMAN | Importin subunit alpha-7 OS=Homo sapiens GN=KPNA6 PE=1 SV=1 | 6,96 | 7,18 | 4 | 7,836 |
| 1680 | sp|P49006|MRP_HUMAN | HUMAN | MARCKS-related protein OS=Homo sapiens GN=MARCKSL1 PE=1 SV=2 | 6,95 | 7,05 | 7 | 40,51 |
| 1681 | sp|P84095|RHOG_HUMAN | HUMAN | Rho-related GTP-binding protein RhoG OS=Homo sapiens GN=RHOG PE=1 SV=1 | 6,93 | 9,27 | 6 | 41,88 |
| 1682 | sp|Q9Y4E8|UBP15_HUMAN | HUMAN | Ubiquitin carboxyl-terminal hydrolase 15 OS=Homo sapiens GN=USP15 PE=1 SV=3 | 6,93 | 7,06 | 4 | 5,81 |
| 1683 | sp|Q8WWM9|CYGB_HUMAN | HUMAN | Cytoglobin OS=Homo sapiens GN=CYGB PE=1 SV=1 | 6,92 | 7,14 | 5 | 30 |
| 1684 | sp|P15559|NQO1_HUMAN | HUMAN | NAD(P)H dehydrogenase [quinone] 1 OS=Homo sapiens GN=NQO1 PE=1 SV=1 | 6,91 | 7,33 | 4 | 17,88 |
| 1685 | sp|Q96A00|PP14A_HUMAN | HUMAN | Protein phosphatase 1 regulatory subunit 14A OS=Homo sapiens GN=PPP1R14A PE=1 SV=1 | 6,91 | 6,98 | 6 | 31,97 |
| 1686 | sp|O75947|ATP5H_HUMAN | HUMAN | ATP synthase subunit d, mitochondrial OS=Homo sapiens GN=ATP5H PE=1 SV=3 | 6,87 | 7,08 | 4 | 34,78 |
| 1687 | sp|P30038|AL4A1_HUMAN | HUMAN | Delta-1-pyrroline-5-carboxylate dehydrogenase, mitochondrial OS=Homo sapiens GN=ALDH4A1 PE=1 SV=3 | 6,86 | 7,07 | 5 | 12,43 |
| 1688 | sp|O43592|XPOT_HUMAN | HUMAN | Exportin-T OS=Homo sapiens GN=XPOT PE=1 SV=2 | 6,86 | 7,03 | 4 | 7,277 |
| 1689 | sp|P17480|UBF1_HUMAN | HUMAN | Nucleolar transcription factor 1 OS=Homo sapiens GN=UBTF PE=1 SV=1 | 6,86 | 7,01 | 4 | 7,33 |
| 1690 | sp|Q8N8S7|ENAH_HUMAN | HUMAN | Protein enabled homolog OS=Homo sapiens GN=ENAH PE=1 SV=2 | 6,86 | 7,01 | 5 | 5,245 |
| 1691 | sp|Q08752|PPID_HUMAN | HUMAN | Peptidyl-prolyl cis-trans isomerase D OS=Homo sapiens GN=PPID PE=1 SV=3 | 6,85 | 7,68 | 9 | 20,54 |
| 1692 | sp|Q01081|U2AF1_HUMAN | HUMAN | Splicing factor U2AF 35 kDa subunit OS=Homo sapiens GN=U2AF1 PE=1 SV=3 | 6,84 | 6,9 | 6 | 21,25 |
| 1693 | sp|Q13310|PABP4_HUMAN | HUMAN | Polyadenylate-binding protein 4 OS=Homo sapiens GN=PABPC4 PE=1 SV=1 | 6,83 | 22,86 | 15 | 22,2 |
| 1694 | sp|Q13131|AAPK1_HUMAN | HUMAN | 5'-AMP-activated protein kinase catalytic subunit alpha-1 OS=Homo sapiens GN=PRKAA1 PE=1 SV=4 | 6,82 | 7,05 | 4 | 8,587 |
| 1695 | sp|P00403|COX2_HUMAN | HUMAN | Cytochrome c oxidase subunit 2 OS=Homo sapiens GN=MT-CO2 PE=1 SV=1 | 6,82 | 6,96 | 4 | 24,67 |
| 1696 | sp|Q92888|ARHG1_HUMAN | HUMAN | Rho guanine nucleotide exchange factor 1 OS=Homo sapiens GN=ARHGEF1 PE=1 SV=2 | 6,81 | 7,32 | 5 | 6,14 |
| 1697 | sp|O00193|SMAP_HUMAN | HUMAN | Small acidic protein OS=Homo sapiens GN=SMAP PE=1 SV=1 | 6,81 | 6,97 | 4 | 26,23 |
| 1698 | sp|Q96I25|SPF45_HUMAN | HUMAN | Splicing factor 45 OS=Homo sapiens GN=RBM17 PE=1 SV=1 | 6,8 | 7,04 | 6 | 14,46 |
| 1699 | sp|O00182|LEG9_HUMAN | HUMAN | Galectin-9 OS=Homo sapiens GN=LGALS9 PE=1 SV=2 | 6,79 | 6,92 | 5 | 17,18 |
| 1700 | sp|Q13617|CUL2_HUMAN | HUMAN | Cullin-2 OS=Homo sapiens GN=CUL2 PE=1 SV=2 | 6,76 | 8,56 | 5 | 7,919 |
| 1701 | sp|P62310|LSM3_HUMAN | HUMAN | U6 snRNA-associated Sm-like protein LSm3 OS=Homo sapiens GN=LSM3 PE=1 SV=2 | 6,75 | 6,84 | 5 | 49,02 |
| 1702 | sp|P36776|LONM_HUMAN | HUMAN | Lon protease homolog, mitochondrial OS=Homo sapiens GN=LONP1 PE=1 SV=2 | 6,74 | 7,04 | 5 | 7,299 |
| 1703 | sp|Q5JSH3|WDR44_HUMAN | HUMAN | WD repeat-containing protein 44 OS=Homo sapiens GN=WDR44 PE=1 SV=1 | 6,7 | 6,98 | 6 | 9,529 |
| 1704 | sp|Q13867|BLMH_HUMAN | HUMAN | Bleomycin hydrolase OS=Homo sapiens GN=BLMH PE=1 SV=1 | 6,69 | 6,93 | 5 | 14,29 |
| 1705 | sp|P20674|COX5A_HUMAN | HUMAN | Cytochrome c oxidase subunit 5A, mitochondrial OS=Homo sapiens GN=COX5A PE=1 SV=2 | 6,65 | 6,84 | 5 | 49,33 |
| 1706 | sp|P13807|GYS1_HUMAN | HUMAN | Glycogen [starch] synthase, muscle OS=Homo sapiens GN=GYS1 PE=1 SV=2 | 6,65 | 6,77 | 4 | 9,227 |
| 1707 | sp|P12235|ADT1_HUMAN | HUMAN | ADP/ATP translocase 1 OS=Homo sapiens GN=SLC25A4 PE=1 SV=4 | 6,64 | 17,16 | 9 | 35,91 |
| 1708 | sp|P55008|AIF1_HUMAN | HUMAN | Allograft inflammatory factor 1 OS=Homo sapiens GN=AIF1 PE=1 SV=1 | 6,64 | 6,68 | 3 | 23,81 |
| 1709 | sp|Q9NVZ3|NECP2_HUMAN | HUMAN | Adaptin ear-binding coat-associated protein 2 OS=Homo sapiens GN=NECAP2 PE=1 SV=1 | 6,63 | 6,75 | 5 | 28,52 |
| 1710 | sp|Q9BY43|CHM4A_HUMAN | HUMAN | Charged multivesicular body protein 4a OS=Homo sapiens GN=CHMP4A PE=1 SV=3 | 6,62 | 6,92 | 5 | 27,48 |
| 1711 | sp|Q8WWM7|ATX2L_HUMAN | HUMAN | Ataxin-2-like protein OS=Homo sapiens GN=ATXN2L PE=1 SV=2 | 6,62 | 6,9 | 4 | 5,302 |
| 1712 | sp|Q96DH6|MSI2H_HUMAN | HUMAN | RNA-binding protein Musashi homolog 2 OS=Homo sapiens GN=MSI2 PE=1 SV=1 | 6,62 | 6,65 | 4 | 18,9 |
| 1713 | sp|P30405|PPIF_HUMAN | HUMAN | Peptidyl-prolyl cis-trans isomerase F, mitochondrial OS=Homo sapiens GN=PPIF PE=1 SV=1 | 6,59 | 7,44 | 5 | 46,86 |
| 1714 | sp|O00743|PPP6_HUMAN | HUMAN | Serine/threonine-protein phosphatase 6 catalytic subunit OS=Homo sapiens GN=PPP6C PE=1 SV=1 | 6,59 | 6,71 | 4 | 18,36 |
| 1715 | sp|P62318|SMD3_HUMAN | HUMAN | Small nuclear ribonucleoprotein Sm D3 OS=Homo sapiens GN=SNRPD3 PE=1 SV=1 | 6,59 | 6,67 | 7 | 53,17 |
| 1716 | sp|Q9Y2T2|AP3M1_HUMAN | HUMAN | AP-3 complex subunit mu-1 OS=Homo sapiens GN=AP3M1 PE=1 SV=1 | 6,56 | 6,76 | 5 | 19,62 |
| 1717 | sp|P28161|GSTM2_HUMAN | HUMAN | Glutathione S-transferase Mu 2 OS=Homo sapiens GN=GSTM2 PE=1 SV=2 | 6,55 | 11,52 | 10 | 32,57 |
| 1718 | sp|Q12907|LMAN2_HUMAN | HUMAN | Vesicular integral-membrane protein VIP36 OS=Homo sapiens GN=LMAN2 PE=1 SV=1 | 6,55 | 7,47 | 5 | 20,79 |
| 1719 | sp|P22087|FBRL_HUMAN | HUMAN | rRNA 2'-O-methyltransferase fibrillarin OS=Homo sapiens GN=FBL PE=1 SV=2 | 6,55 | 6,77 | 4 | 13,4 |
| 1720 | sp|Q9UMX5|NENF_HUMAN | HUMAN | Neudesin OS=Homo sapiens GN=NENF PE=1 SV=1 | 6,55 | 6,63 | 4 | 26,16 |
| 1721 | sp|P42226|STAT6_HUMAN | HUMAN | Signal transducer and activator of transcription 6 OS=Homo sapiens GN=STAT6 PE=1 SV=1 | 6,53 | 6,67 | 4 | 7,084 |
| 1722 | sp|O00442|RTCA_HUMAN | HUMAN | RNA 3'-terminal phosphate cyclase OS=Homo sapiens GN=RTCA PE=1 SV=1 | 6,52 | 6,74 | 6 | 18,58 |
| 1723 | sp|Q92783|STAM1_HUMAN | HUMAN | Signal transducing adapter molecule 1 OS=Homo sapiens GN=STAM PE=1 SV=3 | 6,5 | 6,57 | 4 | 8,889 |
| 1724 | sp|P23946|CMA1_HUMAN | HUMAN | Chymase OS=Homo sapiens GN=CMA1 PE=1 SV=1 | 6,49 | 6,79 | 4 | 24,29 |
| 1725 | sp|O15173|PGRC2_HUMAN | HUMAN | Membrane-associated progesterone receptor component 2 OS=Homo sapiens GN=PGRMC2 PE=1 SV=1 | 6,49 | 6,65 | 4 | 22,42 |
| 1726 | sp|Q9NY12|GAR1_HUMAN | HUMAN | H/ACA ribonucleoprotein complex subunit 1 OS=Homo sapiens GN=GAR1 PE=1 SV=1 | 6,48 | 6,6 | 5 | 19,82 |
| 1727 | sp|Q96FV2|SCRN2_HUMAN | HUMAN | Secernin-2 OS=Homo sapiens GN=SCRN2 PE=1 SV=3 | 6,48 | 6,59 | 4 | 13,88 |
| 1728 | sp|P23634|AT2B4_HUMAN | HUMAN | Plasma membrane calcium-transporting ATPase 4 OS=Homo sapiens GN=ATP2B4 PE=1 SV=2 | 6,46 | 6,62 | 5 | 5,802 |
| 1729 | sp|P05161|ISG15_HUMAN | HUMAN | Ubiquitin-like protein ISG15 OS=Homo sapiens GN=ISG15 PE=1 SV=5 | 6,45 | 6,55 | 5 | 35,15 |
| 1730 | sp|P98082|DAB2_HUMAN | HUMAN | Disabled homolog 2 OS=Homo sapiens GN=DAB2 PE=1 SV=3 | 6,44 | 6,55 | 4 | 6,494 |
| 1731 | sp|P16189|1A31_HUMAN | HUMAN | HLA class I histocompatibility antigen, A-31 alpha chain OS=Homo sapiens GN=HLA-A PE=1 SV=2 | 6,43 | 19,24 | 12 | 33,97 |
| 1732 | sp|Q9Y305|ACOT9_HUMAN | HUMAN | Acyl-coenzyme A thioesterase 9, mitochondrial OS=Homo sapiens GN=ACOT9 PE=1 SV=2 | 6,43 | 6,56 | 4 | 13,67 |
| 1733 | sp|Q53H96|P5CR3_HUMAN | HUMAN | Pyrroline-5-carboxylate reductase 3 OS=Homo sapiens GN=PYCRL PE=1 SV=3 | 6,42 | 6,52 | 5 | 27,01 |
| 1734 | sp|O75607|NPM3_HUMAN | HUMAN | Nucleoplasmin-3 OS=Homo sapiens GN=NPM3 PE=1 SV=3 | 6,42 | 6,47 | 4 | 41,01 |
| 1735 | sp|Q709C8|VP13C_HUMAN | HUMAN | Vacuolar protein sorting-associated protein 13C OS=Homo sapiens GN=VPS13C PE=1 SV=1 | 6,41 | 6,73 | 5 | 1,705 |
| 1736 | sp|P06309|KV205_HUMAN | HUMAN | Ig kappa chain V-II region GM607 (Fragment) OS=Homo sapiens PE=4 SV=1 | 6,41 | 6,53 | 10 | 41,03 |
| 1737 | sp|Q8IXQ6|PARP9_HUMAN | HUMAN | Poly [ADP-ribose] polymerase 9 OS=Homo sapiens GN=PARP9 PE=1 SV=2 | 6,4 | 6,52 | 4 | 5,855 |
| 1738 | sp|Q96SB4|SRPK1_HUMAN | HUMAN | SRSF protein kinase 1 OS=Homo sapiens GN=SRPK1 PE=1 SV=2 | 6,4 | 6,5 | 5 | 8,55 |
| 1739 | sp|P09132|SRP19_HUMAN | HUMAN | Signal recognition particle 19 kDa protein OS=Homo sapiens GN=SRP19 PE=1 SV=3 | 6,4 | 6,43 | 5 | 38,19 |
| 1740 | sp|P20340|RAB6A_HUMAN | HUMAN | Ras-related protein Rab-6A OS=Homo sapiens GN=RAB6A PE=1 SV=3 | 6,39 | 9,19 | 8 | 26,44 |
| 1741 | sp|Q9Y6G9|DC1L1_HUMAN | HUMAN | Cytoplasmic dynein 1 light intermediate chain 1 OS=Homo sapiens GN=DYNC1LI1 PE=1 SV=3 | 6,39 | 9,03 | 5 | 12,81 |
| 1742 | sp|Q05519|SRS11_HUMAN | HUMAN | Serine/arginine-rich splicing factor 11 OS=Homo sapiens GN=SRSF11 PE=1 SV=1 | 6,39 | 6,58 | 4 | 12,4 |
| 1743 | sp|O14841|OPLA_HUMAN | HUMAN | 5-oxoprolinase OS=Homo sapiens GN=OPLAH PE=1 SV=3 | 6,38 | 6,89 | 5 | 5,512 |
| 1744 | sp|P01111|RASN_HUMAN | HUMAN | GTPase NRas OS=Homo sapiens GN=NRAS PE=1 SV=1 | 6,37 | 8,43 | 4 | 25,4 |
| 1745 | sp|Q7Z2W4|ZCCHV_HUMAN | HUMAN | Zinc finger CCCH-type antiviral protein 1 OS=Homo sapiens GN=ZC3HAV1 PE=1 SV=3 | 6,37 | 6,48 | 3 | 4,767 |
| 1746 | sp|Q8WYA6|CTBL1_HUMAN | HUMAN | Beta-catenin-like protein 1 OS=Homo sapiens GN=CTNNBL1 PE=1 SV=1 | 6,37 | 6,4 | 3 | 4,618 |
| 1747 | sp|P17812|PYRG1_HUMAN | HUMAN | CTP synthase 1 OS=Homo sapiens GN=CTPS1 PE=1 SV=2 | 6,36 | 6,59 | 5 | 11,51 |
| 1748 | sp|P62995|TRA2B_HUMAN | HUMAN | Transformer-2 protein homolog beta OS=Homo sapiens GN=TRA2B PE=1 SV=1 | 6,36 | 6,38 | 5 | 15,28 |
| 1749 | sp|Q14141|SEPT6_HUMAN | HUMAN | Septin-6 OS=Homo sapiens GN=SEPT6 PE=1 SV=4 | 6,35 | 16,67 | 11 | 32,49 |
| 1750 | sp|P50897|PPT1_HUMAN | HUMAN | Palmitoyl-protein thioesterase 1 OS=Homo sapiens GN=PPT1 PE=1 SV=1 | 6,34 | 6,36 | 3 | 18,3 |
| 1751 | sp|P25815|S100P_HUMAN | HUMAN | Protein S100-P OS=Homo sapiens GN=S100P PE=1 SV=2 | 6,34 | 6,36 | 7 | 53,68 |
| 1752 | sp|Q9GZT8|NIF3L_HUMAN | HUMAN | NIF3-like protein 1 OS=Homo sapiens GN=NIF3L1 PE=1 SV=2 | 6,33 | 6,35 | 4 | 9,814 |
| 1753 | sp|P10153|RNAS2_HUMAN | HUMAN | Non-secretory ribonuclease OS=Homo sapiens GN=RNASE2 PE=1 SV=2 | 6,33 | 6,35 | 14 | 43,48 |
| 1754 | sp|Q96KG9|NTKL_HUMAN | HUMAN | N-terminal kinase-like protein OS=Homo sapiens GN=SCYL1 PE=1 SV=1 | 6,3 | 6,4 | 3 | 5,569 |
| 1755 | sp|O14929|HAT1_HUMAN | HUMAN | Histone acetyltransferase type B catalytic subunit OS=Homo sapiens GN=HAT1 PE=1 SV=1 | 6,3 | 6,39 | 3 | 11,22 |
| 1756 | sp|Q99757|THIOM_HUMAN | HUMAN | Thioredoxin, mitochondrial OS=Homo sapiens GN=TXN2 PE=1 SV=2 | 6,3 | 6,34 | 3 | 30,12 |
| 1757 | sp|Q9NZZ3|CHMP5_HUMAN | HUMAN | Charged multivesicular body protein 5 OS=Homo sapiens GN=CHMP5 PE=1 SV=1 | 6,29 | 6,38 | 4 | 27,85 |
| 1758 | sp|Q9NP97|DLRB1_HUMAN | HUMAN | Dynein light chain roadblock-type 1 OS=Homo sapiens GN=DYNLRB1 PE=1 SV=3 | 6,29 | 6,37 | 4 | 60,42 |
| 1759 | sp|Q6P587|FAHD1_HUMAN | HUMAN | Acylpyruvase FAHD1, mitochondrial OS=Homo sapiens GN=FAHD1 PE=1 SV=2 | 6,29 | 6,32 | 3 | 27,23 |
| 1760 | sp|Q9Y6B6|SAR1B_HUMAN | HUMAN | GTP-binding protein SAR1b OS=Homo sapiens GN=SAR1B PE=1 SV=1 | 6,28 | 10,66 | 7 | 40,91 |
| 1761 | sp|Q9UN36|NDRG2_HUMAN | HUMAN | Protein NDRG2 OS=Homo sapiens GN=NDRG2 PE=1 SV=2 | 6,28 | 6,43 | 4 | 18,87 |
| 1762 | sp|Q9NQX3|GEPH_HUMAN | HUMAN | Gephyrin OS=Homo sapiens GN=GPHN PE=1 SV=1 | 6,28 | 6,42 | 5 | 8,832 |
| 1763 | sp|Q6UWP2|DHR11_HUMAN | HUMAN | Dehydrogenase/reductase SDR family member 11 OS=Homo sapiens GN=DHRS11 PE=1 SV=1 | 6,28 | 6,42 | 5 | 24,62 |
| 1764 | sp|Q9NRX4|PHP14_HUMAN | HUMAN | 14 kDa phosphohistidine phosphatase OS=Homo sapiens GN=PHPT1 PE=1 SV=1 | 6,28 | 6,4 | 4 | 51,2 |
| 1765 | sp|Q86X55|CARM1_HUMAN | HUMAN | Histone-arginine methyltransferase CARM1 OS=Homo sapiens GN=CARM1 PE=1 SV=3 | 6,27 | 6,48 | 9 | 14,8 |
| 1766 | sp|P50479|PDLI4_HUMAN | HUMAN | PDZ and LIM domain protein 4 OS=Homo sapiens GN=PDLIM4 PE=1 SV=2 | 6,26 | 6,45 | 4 | 22,42 |
| 1767 | sp|O14933|UB2L6_HUMAN | HUMAN | Ubiquitin/ISG15-conjugating enzyme E2 L6 OS=Homo sapiens GN=UBE2L6 PE=1 SV=4 | 6,24 | 6,26 | 5 | 35,29 |
| 1768 | sp|Q9HCY8|S10AE_HUMAN | HUMAN | Protein S100-A14 OS=Homo sapiens GN=S100A14 PE=1 SV=1 | 6,24 | 6,26 | 3 | 35,58 |
| 1769 | sp|P02747|C1QC_HUMAN | HUMAN | Complement C1q subcomponent subunit C OS=Homo sapiens GN=C1QC PE=1 SV=3 | 6,24 | 6,25 | 3 | 17,55 |
| 1770 | sp|O75153|CLU_HUMAN | HUMAN | Clustered mitochondria protein homolog OS=Homo sapiens GN=CLUH PE=1 SV=2 | 6,23 | 6,74 | 3 | 2,521 |
| 1771 | sp|Q6P1M3|L2GL2_HUMAN | HUMAN | Lethal(2) giant larvae protein homolog 2 OS=Homo sapiens GN=LLGL2 PE=1 SV=2 | 6,21 | 6,49 | 5 | 4,314 |
| 1772 | sp|P46937|YAP1_HUMAN | HUMAN | Transcriptional coactivator YAP1 OS=Homo sapiens GN=YAP1 PE=1 SV=2 | 6,21 | 6,45 | 7 | 23,02 |
| 1773 | sp|P08397|HEM3_HUMAN | HUMAN | Porphobilinogen deaminase OS=Homo sapiens GN=HMBS PE=1 SV=2 | 6,21 | 6,41 | 4 | 17,45 |
| 1774 | sp|P12109|CO6A1_HUMAN | HUMAN | Collagen alpha-1(VI) chain OS=Homo sapiens GN=COL6A1 PE=1 SV=3 | 6,21 | 6,35 | 4 | 5,642 |
| 1775 | sp|Q01844|EWS_HUMAN | HUMAN | RNA-binding protein EWS OS=Homo sapiens GN=EWSR1 PE=1 SV=1 | 6,2 | 6,27 | 5 | 8,232 |
| 1776 | sp|P62306|RUXF_HUMAN | HUMAN | Small nuclear ribonucleoprotein F OS=Homo sapiens GN=SNRPF PE=1 SV=1 | 6,2 | 6,22 | 5 | 50 |
| 1777 | sp|P11441|UBL4A_HUMAN | HUMAN | Ubiquitin-like protein 4A OS=Homo sapiens GN=UBL4A PE=1 SV=1 | 6,18 | 6,25 | 3 | 22,93 |
| 1778 | sp|Q12996|CSTF3_HUMAN | HUMAN | Cleavage stimulation factor subunit 3 OS=Homo sapiens GN=CSTF3 PE=1 SV=1 | 6,17 | 6,26 | 3 | 6,695 |
| 1779 | sp|Q8WWX9|SELM_HUMAN | HUMAN | Selenoprotein M OS=Homo sapiens GN=SELM PE=1 SV=3 | 6,17 | 6,26 | 4 | 35,86 |
| 1780 | sp|P05386|RLA1_HUMAN | HUMAN | 60S acidic ribosomal protein P1 OS=Homo sapiens GN=RPLP1 PE=1 SV=1 | 6,16 | 10,59 | 21 | 89,47 |
| 1781 | sp|P63165|SUMO1_HUMAN | HUMAN | Small ubiquitin-related modifier 1 OS=Homo sapiens GN=SUMO1 PE=1 SV=1 | 6,14 | 6,24 | 5 | 42,57 |
| 1782 | sp|O15247|CLIC2_HUMAN | HUMAN | Chloride intracellular channel protein 2 OS=Homo sapiens GN=CLIC2 PE=1 SV=3 | 6,13 | 6,68 | 4 | 21,86 |
| 1783 | sp|Q5VWZ2|LYPL1_HUMAN | HUMAN | Lysophospholipase-like protein 1 OS=Homo sapiens GN=LYPLAL1 PE=1 SV=3 | 6,13 | 6,33 | 6 | 24,89 |
| 1784 | sp|P61077|UB2D3_HUMAN | HUMAN | Ubiquitin-conjugating enzyme E2 D3 OS=Homo sapiens GN=UBE2D3 PE=1 SV=1 | 6,13 | 6,15 | 5 | 36,05 |
| 1785 | sp|Q9P2T1|GMPR2_HUMAN | HUMAN | GMP reductase 2 OS=Homo sapiens GN=GMPR2 PE=1 SV=1 | 6,07 | 6,37 | 5 | 16,38 |
| 1786 | sp|P18754|RCC1_HUMAN | HUMAN | Regulator of chromosome condensation OS=Homo sapiens GN=RCC1 PE=1 SV=1 | 6,07 | 6,21 | 3 | 9,976 |
| 1787 | sp|Q9GZT3|SLIRP_HUMAN | HUMAN | SRA stem-loop-interacting RNA-binding protein, mitochondrial OS=Homo sapiens GN=SLIRP PE=1 SV=1 | 6,06 | 6,26 | 6 | 58,72 |
| 1788 | sp|Q9Y3D6|FIS1_HUMAN | HUMAN | Mitochondrial fission 1 protein OS=Homo sapiens GN=FIS1 PE=1 SV=2 | 6,06 | 6,07 | 5 | 34,21 |
| 1789 | sp|O60234|GMFG_HUMAN | HUMAN | Glia maturation factor gamma OS=Homo sapiens GN=GMFG PE=1 SV=1 | 6,05 | 9,54 | 7 | 46,48 |
| 1790 | sp|Q96GG9|DCNL1_HUMAN | HUMAN | DCN1-like protein 1 OS=Homo sapiens GN=DCUN1D1 PE=1 SV=1 | 6,05 | 6,06 | 3 | 14,67 |
| 1791 | sp|Q9BWJ5|SF3B5_HUMAN | HUMAN | Splicing factor 3B subunit 5 OS=Homo sapiens GN=SF3B5 PE=1 SV=1 | 6,05 | 6,05 | 3 | 40,7 |
| 1792 | sp|O95232|LC7L3_HUMAN | HUMAN | Luc7-like protein 3 OS=Homo sapiens GN=LUC7L3 PE=1 SV=2 | 6,04 | 6,15 | 3 | 10,42 |
| 1793 | sp|P61457|PHS_HUMAN | HUMAN | Pterin-4-alpha-carbinolamine dehydratase OS=Homo sapiens GN=PCBD1 PE=1 SV=2 | 6,04 | 6,13 | 3 | 29,81 |
| 1794 | sp|O60888|CUTA_HUMAN | HUMAN | Protein CutA OS=Homo sapiens GN=CUTA PE=1 SV=2 | 6,04 | 6,05 | 6 | 52,51 |
| 1795 | sp|P42677|RS27_HUMAN | HUMAN | 40S ribosomal protein S27 OS=Homo sapiens GN=RPS27 PE=1 SV=3 | 6,04 | 6,05 | 5 | 39,29 |
| 1796 | sp|P01275|GLUC_HUMAN | HUMAN | Glucagon OS=Homo sapiens GN=GCG PE=1 SV=3 | 6,04 | 6,04 | 3 | 24,44 |
| 1797 | sp|Q15847|ADIRF_HUMAN | HUMAN | Adipogenesis regulatory factor OS=Homo sapiens GN=ADIRF PE=1 SV=1 | 6,03 | 6,07 | 9 | 69,74 |
| 1798 | sp|Q16643|DREB_HUMAN | HUMAN | Drebrin OS=Homo sapiens GN=DBN1 PE=1 SV=4 | 6,02 | 6,18 | 3 | 7,55 |
| 1799 | sp|Q9H9H4|VP37B_HUMAN | HUMAN | Vacuolar protein sorting-associated protein 37B OS=Homo sapiens GN=VPS37B PE=1 SV=1 | 6,01 | 6,03 | 4 | 22,46 |
| 1800 | sp|Q5JTJ3|COA6_HUMAN | HUMAN | Cytochrome c oxidase assembly factor 6 homolog OS=Homo sapiens GN=COA6 PE=1 SV=1 | 6,01 | 6,02 | 3 | 32 |
| 1801 | sp|Q14011|CIRBP_HUMAN | HUMAN | Cold-inducible RNA-binding protein OS=Homo sapiens GN=CIRBP PE=1 SV=1 | 6,01 | 6,01 | 5 | 26,74 |
| 1802 | sp|P68371|TBB4B_HUMAN | HUMAN | Tubulin beta-4B chain OS=Homo sapiens GN=TUBB4B PE=1 SV=1 | 6 | 64,54 | 112 | 84,49 |
| 1803 | sp|Q9BQE3|TBA1C_HUMAN | HUMAN | Tubulin alpha-1C chain OS=Homo sapiens GN=TUBA1C PE=1 SV=1 | 6 | 62,03 | 95 | 90,87 |
| 1804 | sp|P35241|RADI_HUMAN | HUMAN | Radixin OS=Homo sapiens GN=RDX PE=1 SV=1 | 6 | 36,77 | 29 | 32,08 |
| 1805 | sp|Q9H0U4|RAB1B_HUMAN | HUMAN | Ras-related protein Rab-1B OS=Homo sapiens GN=RAB1B PE=1 SV=1 | 6 | 23,79 | 19 | 73,13 |
| 1806 | sp|P30461|1B13_HUMAN | HUMAN | HLA class I histocompatibility antigen, B-13 alpha chain OS=Homo sapiens GN=HLA-B PE=1 SV=1 | 6 | 21,07 | 13 | 40,61 |
| 1807 | sp|P19105|ML12A_HUMAN | HUMAN | Myosin regulatory light chain 12A OS=Homo sapiens GN=MYL12A PE=1 SV=2 | 6 | 20,19 | 20 | 72,51 |
| 1808 | sp|Q9GZU8|F192A_HUMAN | HUMAN | Protein FAM192A OS=Homo sapiens GN=FAM192A PE=1 SV=1 | 6 | 6,01 | 3 | 16,14 |
| 1809 | sp|Q8IXM2|BAP18_HUMAN | HUMAN | Chromatin complexes subunit BAP18 OS=Homo sapiens GN=BAP18 PE=1 SV=1 | 6 | 6 | 3 | 28,49 |
| 1810 | sp|Q9NX55|HYPK_HUMAN | HUMAN | Huntingtin-interacting protein K OS=Homo sapiens GN=HYPK PE=1 SV=2 | 6 | 6 | 3 | 33,33 |
| 1811 | sp|P61956|SUMO2_HUMAN | HUMAN | Small ubiquitin-related modifier 2 OS=Homo sapiens GN=SUMO2 PE=1 SV=3 | 6 | 6 | 16 | 58,95 |
| 1812 | sp|P62857|RS28_HUMAN | HUMAN | 40S ribosomal protein S28 OS=Homo sapiens GN=RPS28 PE=1 SV=1 | 6 | 6 | 6 | 46,38 |
| 1813 | sp|O43670|ZN207_HUMAN | HUMAN | BUB3-interacting and GLEBS motif-containing protein ZNF207 OS=Homo sapiens GN=ZNF207 PE=1 SV=1 | 6 | 6 | 3 | 7,95 |
| 1814 | sp|Q13542|4EBP2_HUMAN | HUMAN | Eukaryotic translation initiation factor 4E-binding protein 2 OS=Homo sapiens GN=EIF4EBP2 PE=1 SV=1 | 6 | 6 | 3 | 61,67 |
| 1815 | sp|P01743|HV102_HUMAN | HUMAN | Ig heavy chain V-I region HG3 OS=Homo sapiens PE=3 SV=1 | 6 | 6 | 8 | 32,48 |
| 1816 | sp|P01037|CYTN_HUMAN | HUMAN | Cystatin-SN OS=Homo sapiens GN=CST1 PE=1 SV=3 | 5,96 | 6,11 | 4 | 43,97 |
| 1817 | sp|Q16576|RBBP7_HUMAN | HUMAN | Histone-binding protein RBBP7 OS=Homo sapiens GN=RBBP7 PE=1 SV=1 | 5,94 | 18,03 | 11 | 29,18 |
| 1818 | sp|P12104|FABPI_HUMAN | HUMAN | Fatty acid-binding protein, intestinal OS=Homo sapiens GN=FABP2 PE=1 SV=2 | 5,93 | 6,23 | 5 | 35,61 |
| 1819 | sp|P80748|LV302_HUMAN | HUMAN | Ig lambda chain V-III region LOI OS=Homo sapiens PE=1 SV=1 | 5,93 | 5,97 | 7 | 45,05 |
| 1820 | sp|P49257|LMAN1_HUMAN | HUMAN | Protein ERGIC-53 OS=Homo sapiens GN=LMAN1 PE=1 SV=2 | 5,92 | 6,01 | 3 | 7,647 |
| 1821 | sp|O75348|VATG1_HUMAN | HUMAN | V-type proton ATPase subunit G 1 OS=Homo sapiens GN=ATP6V1G1 PE=1 SV=3 | 5,91 | 6,03 | 4 | 36,44 |
| 1822 | sp|P62253|UB2G1_HUMAN | HUMAN | Ubiquitin-conjugating enzyme E2 G1 OS=Homo sapiens GN=UBE2G1 PE=1 SV=3 | 5,89 | 5,93 | 3 | 23,53 |
| 1823 | sp|P15927|RFA2_HUMAN | HUMAN | Replication protein A 32 kDa subunit OS=Homo sapiens GN=RPA2 PE=1 SV=1 | 5,87 | 6,02 | 7 | 26,67 |
| 1824 | sp|Q9P1F3|ABRAL_HUMAN | HUMAN | Costars family protein ABRACL OS=Homo sapiens GN=ABRACL PE=1 SV=1 | 5,87 | 5,94 | 5 | 43,21 |
| 1825 | sp|P08559|ODPA_HUMAN | HUMAN | Pyruvate dehydrogenase E1 component subunit alpha, somatic form, mitochondrial OS=Homo sapiens GN=PDHA1 PE=1 SV=3 | 5,86 | 5,97 | 6 | 18,97 |
| 1826 | sp|Q9NP79|VTA1_HUMAN | HUMAN | Vacuolar protein sorting-associated protein VTA1 homolog OS=Homo sapiens GN=VTA1 PE=1 SV=1 | 5,86 | 5,95 | 3 | 14,33 |
| 1827 | sp|P40616|ARL1_HUMAN | HUMAN | ADP-ribosylation factor-like protein 1 OS=Homo sapiens GN=ARL1 PE=1 SV=1 | 5,86 | 5,93 | 4 | 33,7 |
| 1828 | sp|P16885|PLCG2_HUMAN | HUMAN | 1-phosphatidylinositol 4,5-bisphosphate phosphodiesterase gamma-2 OS=Homo sapiens GN=PLCG2 PE=1 SV=4 | 5,84 | 6,44 | 4 | 2,688 |
| 1829 | sp|Q9H8H3|MET7A_HUMAN | HUMAN | Methyltransferase-like protein 7A OS=Homo sapiens GN=METTL7A PE=1 SV=1 | 5,82 | 5,85 | 3 | 16,8 |
| 1830 | sp|Q7LG56|RIR2B_HUMAN | HUMAN | Ribonucleoside-diphosphate reductase subunit M2 B OS=Homo sapiens GN=RRM2B PE=1 SV=1 | 5,81 | 7,62 | 4 | 14,25 |
| 1831 | sp|O00401|WASL_HUMAN | HUMAN | Neural Wiskott-Aldrich syndrome protein OS=Homo sapiens GN=WASL PE=1 SV=2 | 5,81 | 6,13 | 4 | 14,46 |
| 1832 | sp|Q8WUW1|BRK1_HUMAN | HUMAN | Protein BRICK1 OS=Homo sapiens GN=BRK1 PE=1 SV=1 | 5,81 | 6,09 | 3 | 42,67 |
| 1833 | sp|Q9UEE9|CFDP1_HUMAN | HUMAN | Craniofacial development protein 1 OS=Homo sapiens GN=CFDP1 PE=1 SV=1 | 5,8 | 5,9 | 3 | 15,38 |
| 1834 | sp|Q9Y5Y2|NUBP2_HUMAN | HUMAN | Cytosolic Fe-S cluster assembly factor NUBP2 OS=Homo sapiens GN=NUBP2 PE=1 SV=1 | 5,8 | 5,86 | 3 | 18,82 |
| 1835 | sp|Q96RU3|FNBP1_HUMAN | HUMAN | Formin-binding protein 1 OS=Homo sapiens GN=FNBP1 PE=1 SV=2 | 5,79 | 5,91 | 4 | 5,186 |
| 1836 | sp|Q13907|IDI1_HUMAN | HUMAN | Isopentenyl-diphosphate Delta-isomerase 1 OS=Homo sapiens GN=IDI1 PE=1 SV=2 | 5,79 | 5,88 | 3 | 19,38 |
| 1837 | sp|O60256|KPRB_HUMAN | HUMAN | Phosphoribosyl pyrophosphate synthase-associated protein 2 OS=Homo sapiens GN=PRPSAP2 PE=1 SV=1 | 5,78 | 8,66 | 6 | 18,97 |
| 1838 | sp|P51571|SSRD_HUMAN | HUMAN | Translocon-associated protein subunit delta OS=Homo sapiens GN=SSR4 PE=1 SV=1 | 5,77 | 5,86 | 4 | 30,64 |
| 1839 | sp|Q9BS40|LXN_HUMAN | HUMAN | Latexin OS=Homo sapiens GN=LXN PE=1 SV=2 | 5,76 | 5,86 | 4 | 19,37 |
| 1840 | sp|P61421|VA0D1_HUMAN | HUMAN | V-type proton ATPase subunit d 1 OS=Homo sapiens GN=ATP6V0D1 PE=1 SV=1 | 5,76 | 5,83 | 3 | 13,11 |
| 1841 | sp|P09012|SNRPA_HUMAN | HUMAN | U1 small nuclear ribonucleoprotein A OS=Homo sapiens GN=SNRPA PE=1 SV=3 | 5,75 | 9,66 | 7 | 24,82 |
| 1842 | sp|Q9Y5X1|SNX9_HUMAN | HUMAN | Sorting nexin-9 OS=Homo sapiens GN=SNX9 PE=1 SV=1 | 5,75 | 5,9 | 4 | 10,08 |
| 1843 | sp|Q6JBY9|CPZIP_HUMAN | HUMAN | CapZ-interacting protein OS=Homo sapiens GN=RCSD1 PE=1 SV=1 | 5,75 | 5,84 | 3 | 10,58 |
| 1844 | sp|P60866|RS20_HUMAN | HUMAN | 40S ribosomal protein S20 OS=Homo sapiens GN=RPS20 PE=1 SV=1 | 5,74 | 5,8 | 4 | 25,21 |
| 1845 | sp|Q9NY27|PP4R2_HUMAN | HUMAN | Serine/threonine-protein phosphatase 4 regulatory subunit 2 OS=Homo sapiens GN=PPP4R2 PE=1 SV=3 | 5,72 | 5,92 | 3 | 10,31 |
| 1846 | sp|P22894|MMP8_HUMAN | HUMAN | Neutrophil collagenase OS=Homo sapiens GN=MMP8 PE=1 SV=1 | 5,72 | 5,77 | 4 | 9,208 |
| 1847 | sp|P35908|K22E_HUMAN | HUMAN | Keratin, type II cytoskeletal 2 epidermal OS=Homo sapiens GN=KRT2 PE=1 SV=2 | 5,69 | 12,9 | 8 | 11,42 |
| 1848 | sp|Q5JTH9|RRP12_HUMAN | HUMAN | RRP12-like protein OS=Homo sapiens GN=RRP12 PE=1 SV=2 | 5,69 | 5,89 | 4 | 4,009 |
| 1849 | sp|P18428|LBP_HUMAN | HUMAN | Lipopolysaccharide-binding protein OS=Homo sapiens GN=LBP PE=1 SV=3 | 5,69 | 5,85 | 4 | 12,27 |
| 1850 | sp|Q9BRJ6|CG050_HUMAN | HUMAN | Uncharacterized protein C7orf50 OS=Homo sapiens GN=C7orf50 PE=1 SV=1 | 5,69 | 5,78 | 3 | 24,23 |
| 1851 | sp|Q13938|CAYP1_HUMAN | HUMAN | Calcyphosin OS=Homo sapiens GN=CAPS PE=1 SV=1 | 5,68 | 5,72 | 3 | 21,16 |
| 1852 | sp|P23786|CPT2_HUMAN | HUMAN | Carnitine O-palmitoyltransferase 2, mitochondrial OS=Homo sapiens GN=CPT2 PE=1 SV=2 | 5,67 | 5,9 | 5 | 8,815 |
| 1853 | sp|Q9BVK6|TMED9_HUMAN | HUMAN | Transmembrane emp24 domain-containing protein 9 OS=Homo sapiens GN=TMED9 PE=1 SV=2 | 5,67 | 5,8 | 4 | 20 |
| 1854 | sp|Q9HB07|MYG1_HUMAN | HUMAN | UPF0160 protein MYG1, mitochondrial OS=Homo sapiens GN=C12orf10 PE=1 SV=2 | 5,67 | 5,78 | 3 | 7,979 |
| 1855 | sp|P55769|NH2L1_HUMAN | HUMAN | NHP2-like protein 1 OS=Homo sapiens GN=SNU13 PE=1 SV=3 | 5,67 | 5,74 | 4 | 27,34 |
| 1856 | sp|P10606|COX5B_HUMAN | HUMAN | Cytochrome c oxidase subunit 5B, mitochondrial OS=Homo sapiens GN=COX5B PE=1 SV=2 | 5,66 | 5,75 | 4 | 32,56 |
| 1857 | sp|Q9Y5A7|NUB1_HUMAN | HUMAN | NEDD8 ultimate buster 1 OS=Homo sapiens GN=NUB1 PE=1 SV=2 | 5,63 | 5,96 | 6 | 10,73 |
| 1858 | sp|P36542|ATPG_HUMAN | HUMAN | ATP synthase subunit gamma, mitochondrial OS=Homo sapiens GN=ATP5C1 PE=1 SV=1 | 5,63 | 5,77 | 3 | 11,74 |
| 1859 | sp|Q9UBC5|MYO1A_HUMAN | HUMAN | Unconventional myosin-Ia OS=Homo sapiens GN=MYO1A PE=1 SV=1 | 5,62 | 10,15 | 6 | 6,328 |
| 1860 | sp|P20036|DPA1_HUMAN | HUMAN | HLA class II histocompatibility antigen, DP alpha 1 chain OS=Homo sapiens GN=HLA-DPA1 PE=1 SV=1 | 5,62 | 5,66 | 3 | 17,69 |
| 1861 | sp|P17213|BPI_HUMAN | HUMAN | Bactericidal permeability-increasing protein OS=Homo sapiens GN=BPI PE=1 SV=4 | 5,61 | 5,75 | 4 | 12,73 |
| 1862 | sp|Q9Y2T3|GUAD_HUMAN | HUMAN | Guanine deaminase OS=Homo sapiens GN=GDA PE=1 SV=1 | 5,59 | 5,73 | 3 | 7,93 |
| 1863 | sp|Q9NZJ9|NUDT4_HUMAN | HUMAN | Diphosphoinositol polyphosphate phosphohydrolase 2 OS=Homo sapiens GN=NUDT4 PE=1 SV=2 | 5,58 | 5,74 | 4 | 17,22 |
| 1864 | sp|Q9NRF8|PYRG2_HUMAN | HUMAN | CTP synthase 2 OS=Homo sapiens GN=CTPS2 PE=1 SV=1 | 5,57 | 5,68 | 3 | 6,655 |
| 1865 | sp|P46821|MAP1B_HUMAN | HUMAN | Microtubule-associated protein 1B OS=Homo sapiens GN=MAP1B PE=1 SV=2 | 5,56 | 5,84 | 4 | 2,229 |
| 1866 | sp|Q14BN4|SLMAP_HUMAN | HUMAN | Sarcolemmal membrane-associated protein OS=Homo sapiens GN=SLMAP PE=1 SV=1 | 5,56 | 5,77 | 3 | 3,986 |
| 1867 | sp|Q01415|GALK2_HUMAN | HUMAN | N-acetylgalactosamine kinase OS=Homo sapiens GN=GALK2 PE=1 SV=1 | 5,54 | 5,8 | 4 | 13,1 |
| 1868 | sp|Q6P2E9|EDC4_HUMAN | HUMAN | Enhancer of mRNA-decapping protein 4 OS=Homo sapiens GN=EDC4 PE=1 SV=1 | 5,54 | 5,71 | 3 | 4,14 |
| 1869 | sp|Q15758|AAAT_HUMAN | HUMAN | Neutral amino acid transporter B(0) OS=Homo sapiens GN=SLC1A5 PE=1 SV=2 | 5,54 | 5,61 | 3 | 8,688 |
| 1870 | sp|P50238|CRIP1_HUMAN | HUMAN | Cysteine-rich protein 1 OS=Homo sapiens GN=CRIP1 PE=1 SV=3 | 5,54 | 5,6 | 5 | 63,64 |
| 1871 | sp|Q14165|MLEC_HUMAN | HUMAN | Malectin OS=Homo sapiens GN=MLEC PE=1 SV=1 | 5,53 | 5,83 | 5 | 22,6 |
| 1872 | sp|Q63ZY3|KANK2_HUMAN | HUMAN | KN motif and ankyrin repeat domain-containing protein 2 OS=Homo sapiens GN=KANK2 PE=1 SV=1 | 5,53 | 5,73 | 4 | 6,345 |
| 1873 | sp|Q15057|ACAP2_HUMAN | HUMAN | Arf-GAP with coiled-coil, ANK repeat and PH domain-containing protein 2 OS=Homo sapiens GN=ACAP2 PE=1 SV=3 | 5,53 | 5,7 | 3 | 4,37 |
| 1874 | sp|Q8NBQ5|DHB11_HUMAN | HUMAN | Estradiol 17-beta-dehydrogenase 11 OS=Homo sapiens GN=HSD17B11 PE=1 SV=3 | 5,52 | 5,61 | 4 | 16,67 |
| 1875 | sp|Q9Y606|TRUA_HUMAN | HUMAN | tRNA pseudouridine synthase A, mitochondrial OS=Homo sapiens GN=PUS1 PE=1 SV=3 | 5,51 | 5,58 | 3 | 7,728 |
| 1876 | sp|Q9H4G0|E41L1_HUMAN | HUMAN | Band 4.1-like protein 1 OS=Homo sapiens GN=EPB41L1 PE=1 SV=2 | 5,5 | 6,68 | 7 | 10,1 |
| 1877 | sp|Q96T51|RUFY1_HUMAN | HUMAN | RUN and FYVE domain-containing protein 1 OS=Homo sapiens GN=RUFY1 PE=1 SV=2 | 5,5 | 5,8 | 4 | 8,757 |
| 1878 | sp|P04839|CY24B_HUMAN | HUMAN | Cytochrome b-245 heavy chain OS=Homo sapiens GN=CYBB PE=1 SV=2 | 5,5 | 5,64 | 5 | 12,46 |
| 1879 | sp|O76070|SYUG_HUMAN | HUMAN | Gamma-synuclein OS=Homo sapiens GN=SNCG PE=1 SV=2 | 5,5 | 5,58 | 4 | 51,18 |
| 1880 | sp|P16989|YBOX3_HUMAN | HUMAN | Y-box-binding protein 3 OS=Homo sapiens GN=YBX3 PE=1 SV=4 | 5,49 | 14,24 | 12 | 45,7 |
| 1881 | sp|P09486|SPRC_HUMAN | HUMAN | SPARC OS=Homo sapiens GN=SPARC PE=1 SV=1 | 5,49 | 5,68 | 4 | 15,51 |
| 1882 | sp|P28676|GRAN_HUMAN | HUMAN | Grancalcin OS=Homo sapiens GN=GCA PE=1 SV=2 | 5,49 | 5,65 | 6 | 22,12 |
| 1883 | sp|Q5TBC7|B2L15_HUMAN | HUMAN | Bcl-2-like protein 15 OS=Homo sapiens GN=BCL2L15 PE=1 SV=1 | 5,46 | 5,56 | 4 | 27,61 |
| 1884 | sp|Q6UXH1|CREL2_HUMAN | HUMAN | Cysteine-rich with EGF-like domain protein 2 OS=Homo sapiens GN=CRELD2 PE=1 SV=1 | 5,45 | 5,59 | 3 | 9,915 |
| 1885 | sp|O15160|RPAC1_HUMAN | HUMAN | DNA-directed RNA polymerases I and III subunit RPAC1 OS=Homo sapiens GN=POLR1C PE=1 SV=1 | 5,45 | 5,56 | 3 | 13,01 |
| 1886 | sp|Q14694|UBP10_HUMAN | HUMAN | Ubiquitin carboxyl-terminal hydrolase 10 OS=Homo sapiens GN=USP10 PE=1 SV=2 | 5,44 | 5,64 | 4 | 8,396 |
| 1887 | sp|Q03252|LMNB2_HUMAN | HUMAN | Lamin-B2 OS=Homo sapiens GN=LMNB2 PE=1 SV=4 | 5,42 | 8,59 | 5 | 8,71 |
| 1888 | sp|O60879|DIAP2_HUMAN | HUMAN | Protein diaphanous homolog 2 OS=Homo sapiens GN=DIAPH2 PE=1 SV=1 | 5,41 | 5,87 | 4 | 3,451 |
| 1889 | sp|Q9UBW8|CSN7A_HUMAN | HUMAN | COP9 signalosome complex subunit 7a OS=Homo sapiens GN=COPS7A PE=1 SV=1 | 5,41 | 5,59 | 4 | 20,73 |
| 1890 | sp|O43847|NRDC_HUMAN | HUMAN | Nardilysin OS=Homo sapiens GN=NRDC PE=1 SV=2 | 5,4 | 5,85 | 6 | 4,435 |
| 1891 | sp|Q969Q0|RL36L_HUMAN | HUMAN | 60S ribosomal protein L36a-like OS=Homo sapiens GN=RPL36AL PE=1 SV=3 | 5,4 | 5,59 | 6 | 35,85 |
| 1892 | sp|Q96M27|PRRC1_HUMAN | HUMAN | Protein PRRC1 OS=Homo sapiens GN=PRRC1 PE=1 SV=1 | 5,4 | 5,58 | 4 | 11,69 |
| 1893 | sp|Q9UPN7|PP6R1_HUMAN | HUMAN | Serine/threonine-protein phosphatase 6 regulatory subunit 1 OS=Homo sapiens GN=PPP6R1 PE=1 SV=5 | 5,39 | 5,43 | 3 | 8,967 |
| 1894 | sp|Q12768|STRUM_HUMAN | HUMAN | WASH complex subunit strumpellin OS=Homo sapiens GN=KIAA0196 PE=1 SV=1 | 5,38 | 5,95 | 6 | 4,745 |
| 1895 | sp|Q99538|LGMN_HUMAN | HUMAN | Legumain OS=Homo sapiens GN=LGMN PE=1 SV=1 | 5,38 | 5,51 | 3 | 9,7 |
| 1896 | sp|Q9Y3B4|SF3B6_HUMAN | HUMAN | Splicing factor 3B subunit 6 OS=Homo sapiens GN=SF3B6 PE=1 SV=1 | 5,38 | 5,46 | 3 | 23,2 |
| 1897 | sp|Q969G3|SMCE1_HUMAN | HUMAN | SWI/SNF-related matrix-associated actin-dependent regulator of chromatin subfamily E member 1 OS=Homo sapiens GN=SMARCE1 PE=1 SV=2 | 5,38 | 5,45 | 3 | 9,246 |
| 1898 | sp|Q9UHJ6|SHPK_HUMAN | HUMAN | Sedoheptulokinase OS=Homo sapiens GN=SHPK PE=1 SV=3 | 5,37 | 5,52 | 4 | 13,18 |
| 1899 | sp|Q04837|SSBP_HUMAN | HUMAN | Single-stranded DNA-binding protein, mitochondrial OS=Homo sapiens GN=SSBP1 PE=1 SV=1 | 5,36 | 5,51 | 5 | 31,08 |
| 1900 | sp|Q8WVY7|UBCP1_HUMAN | HUMAN | Ubiquitin-like domain-containing CTD phosphatase 1 OS=Homo sapiens GN=UBLCP1 PE=1 SV=2 | 5,35 | 5,51 | 5 | 19,5 |
| 1901 | sp|Q9H0P0|5NT3A_HUMAN | HUMAN | Cytosolic 5'-nucleotidase 3A OS=Homo sapiens GN=NT5C3A PE=1 SV=3 | 5,35 | 5,45 | 5 | 11,31 |
| 1902 | sp|Q9P000|COMD9_HUMAN | HUMAN | COMM domain-containing protein 9 OS=Homo sapiens GN=COMMD9 PE=1 SV=2 | 5,35 | 5,41 | 3 | 26,77 |
| 1903 | sp|Q9H0R4|HDHD2_HUMAN | HUMAN | Haloacid dehalogenase-like hydrolase domain-containing protein 2 OS=Homo sapiens GN=HDHD2 PE=1 SV=1 | 5,34 | 5,43 | 4 | 27,8 |
| 1904 | sp|Q99614|TTC1_HUMAN | HUMAN | Tetratricopeptide repeat protein 1 OS=Homo sapiens GN=TTC1 PE=1 SV=1 | 5,33 | 5,38 | 3 | 13,7 |
| 1905 | sp|Q01650|LAT1_HUMAN | HUMAN | Large neutral amino acids transporter small subunit 1 OS=Homo sapiens GN=SLC7A5 PE=1 SV=2 | 5,32 | 5,37 | 3 | 7,692 |
| 1906 | sp|Q15714|T22D1_HUMAN | HUMAN | TSC22 domain family protein 1 OS=Homo sapiens GN=TSC22D1 PE=1 SV=3 | 5,31 | 5,44 | 4 | 6,71 |
| 1907 | sp|P11233|RALA_HUMAN | HUMAN | Ras-related protein Ral-A OS=Homo sapiens GN=RALA PE=1 SV=1 | 5,31 | 5,43 | 3 | 16,02 |
| 1908 | sp|Q9Y4Z0|LSM4_HUMAN | HUMAN | U6 snRNA-associated Sm-like protein LSm4 OS=Homo sapiens GN=LSM4 PE=1 SV=1 | 5,31 | 5,37 | 3 | 20,14 |
| 1909 | sp|Q9UHD1|CHRD1_HUMAN | HUMAN | Cysteine and histidine-rich domain-containing protein 1 OS=Homo sapiens GN=CHORDC1 PE=1 SV=2 | 5,3 | 5,48 | 3 | 13,25 |
| 1910 | sp|Q53FT3|HIKES_HUMAN | HUMAN | Protein Hikeshi OS=Homo sapiens GN=C11orf73 PE=1 SV=2 | 5,3 | 5,35 | 3 | 15,74 |
| 1911 | sp|P06681|CO2_HUMAN | HUMAN | Complement C2 OS=Homo sapiens GN=C2 PE=1 SV=2 | 5,29 | 5,52 | 4 | 6,383 |
| 1912 | sp|Q9NP61|ARFG3_HUMAN | HUMAN | ADP-ribosylation factor GTPase-activating protein 3 OS=Homo sapiens GN=ARFGAP3 PE=1 SV=1 | 5,29 | 5,5 | 4 | 8,14 |
| 1913 | sp|Q04206|TF65_HUMAN | HUMAN | Transcription factor p65 OS=Homo sapiens GN=RELA PE=1 SV=2 | 5,28 | 5,45 | 3 | 6,534 |
| 1914 | sp|P62312|LSM6_HUMAN | HUMAN | U6 snRNA-associated Sm-like protein LSm6 OS=Homo sapiens GN=LSM6 PE=1 SV=1 | 5,28 | 5,44 | 4 | 43,75 |
| 1915 | sp|Q9H4G4|GAPR1_HUMAN | HUMAN | Golgi-associated plant pathogenesis-related protein 1 OS=Homo sapiens GN=GLIPR2 PE=1 SV=3 | 5,27 | 5,32 | 3 | 27,92 |
| 1916 | sp|O75439|MPPB_HUMAN | HUMAN | Mitochondrial-processing peptidase subunit beta OS=Homo sapiens GN=PMPCB PE=1 SV=2 | 5,26 | 5,44 | 3 | 8,793 |
| 1917 | sp|P56134|ATPK_HUMAN | HUMAN | ATP synthase subunit f, mitochondrial OS=Homo sapiens GN=ATP5J2 PE=1 SV=3 | 5,26 | 5,39 | 4 | 39,36 |
| 1918 | sp|Q99584|S10AD_HUMAN | HUMAN | Protein S100-A13 OS=Homo sapiens GN=S100A13 PE=1 SV=1 | 5,26 | 5,38 | 3 | 32,65 |
| 1919 | sp|Q96DI7|SNR40_HUMAN | HUMAN | U5 small nuclear ribonucleoprotein 40 kDa protein OS=Homo sapiens GN=SNRNP40 PE=1 SV=1 | 5,25 | 6,21 | 4 | 12,04 |
| 1920 | sp|P15104|GLNA_HUMAN | HUMAN | Glutamine synthetase OS=Homo sapiens GN=GLUL PE=1 SV=4 | 5,25 | 5,55 | 4 | 14,21 |
| 1921 | sp|Q8IUX7|AEBP1_HUMAN | HUMAN | Adipocyte enhancer-binding protein 1 OS=Homo sapiens GN=AEBP1 PE=1 SV=1 | 5,24 | 5,78 | 5 | 4,491 |
| 1922 | sp|Q9Y5J9|TIM8B_HUMAN | HUMAN | Mitochondrial import inner membrane translocase subunit Tim8 B OS=Homo sapiens GN=TIMM8B PE=1 SV=1 | 5,24 | 5,46 | 3 | 37,35 |
| 1923 | sp|Q8WU79|SMAP2_HUMAN | HUMAN | Stromal membrane-associated protein 2 OS=Homo sapiens GN=SMAP2 PE=1 SV=1 | 5,24 | 5,42 | 4 | 10,72 |
| 1924 | sp|Q9Y223|GLCNE_HUMAN | HUMAN | Bifunctional UDP-N-acetylglucosamine 2-epimerase/N-acetylmannosamine kinase OS=Homo sapiens GN=GNE PE=1 SV=1 | 5,24 | 5,37 | 3 | 7,064 |
| 1925 | sp|Q9UMS0|NFU1_HUMAN | HUMAN | NFU1 iron-sulfur cluster scaffold homolog, mitochondrial OS=Homo sapiens GN=NFU1 PE=1 SV=2 | 5,24 | 5,3 | 3 | 15,75 |
| 1926 | sp|Q5T6V5|CI064_HUMAN | HUMAN | UPF0553 protein C9orf64 OS=Homo sapiens GN=C9orf64 PE=1 SV=1 | 5,22 | 5,36 | 2 | 9,677 |
| 1927 | sp|O00244|ATOX1_HUMAN | HUMAN | Copper transport protein ATOX1 OS=Homo sapiens GN=ATOX1 PE=1 SV=1 | 5,21 | 5,26 | 3 | 50 |
| 1928 | sp|P04271|S100B_HUMAN | HUMAN | Protein S100-B OS=Homo sapiens GN=S100B PE=1 SV=2 | 5,19 | 5,26 | 3 | 56,52 |
| 1929 | sp|Q9BUP3|HTAI2_HUMAN | HUMAN | Oxidoreductase HTATIP2 OS=Homo sapiens GN=HTATIP2 PE=1 SV=2 | 5,18 | 5,64 | 4 | 23,14 |
| 1930 | sp|O60826|CCD22_HUMAN | HUMAN | Coiled-coil domain-containing protein 22 OS=Homo sapiens GN=CCDC22 PE=1 SV=1 | 5,18 | 5,36 | 5 | 10,37 |
| 1931 | sp|Q9NVJ2|ARL8B_HUMAN | HUMAN | ADP-ribosylation factor-like protein 8B OS=Homo sapiens GN=ARL8B PE=1 SV=1 | 5,16 | 5,27 | 3 | 22,04 |
| 1932 | sp|P22105|TENX_HUMAN | HUMAN | Tenascin-X OS=Homo sapiens GN=TNXB PE=1 SV=4 | 5,15 | 5,55 | 4 | 3,984 |
| 1933 | sp|Q9Y3A3|PHOCN_HUMAN | HUMAN | MOB-like protein phocein OS=Homo sapiens GN=MOB4 PE=1 SV=1 | 5,15 | 5,21 | 3 | 19,11 |
| 1934 | sp|C4AMC7|WASH3_HUMAN | HUMAN | Putative WAS protein family homolog 3 OS=Homo sapiens GN=WASH3P PE=1 SV=2 | 5,14 | 5,33 | 3 | 9,719 |
| 1935 | sp|P41236|IPP2_HUMAN | HUMAN | Protein phosphatase inhibitor 2 OS=Homo sapiens GN=PPP1R2 PE=1 SV=2 | 5,13 | 5,27 | 3 | 12,68 |
| 1936 | sp|Q9UNN8|EPCR_HUMAN | HUMAN | Endothelial protein C receptor OS=Homo sapiens GN=PROCR PE=1 SV=1 | 5,13 | 5,18 | 3 | 14,29 |
| 1937 | sp|Q9Y2H0|DLGP4_HUMAN | HUMAN | Disks large-associated protein 4 OS=Homo sapiens GN=DLGAP4 PE=1 SV=3 | 5,12 | 5,24 | 3 | 4,234 |
| 1938 | sp|P15941|MUC1_HUMAN | HUMAN | Mucin-1 OS=Homo sapiens GN=MUC1 PE=1 SV=3 | 5,12 | 5,17 | 4 | 2,629 |
| 1939 | sp|P0C0L5|CO4B_HUMAN | HUMAN | Complement C4-B OS=Homo sapiens GN=C4B PE=1 SV=2 | 5,11 | 119,57 | 70 | 42,95 |
| 1940 | sp|P34896|GLYC_HUMAN | HUMAN | Serine hydroxymethyltransferase, cytosolic OS=Homo sapiens GN=SHMT1 PE=1 SV=1 | 5,11 | 7,82 | 5 | 13,25 |
| 1941 | sp|Q00765|REEP5_HUMAN | HUMAN | Receptor expression-enhancing protein 5 OS=Homo sapiens GN=REEP5 PE=1 SV=3 | 5,11 | 5,28 | 3 | 15,34 |
| 1942 | sp|P32322|P5CR1_HUMAN | HUMAN | Pyrroline-5-carboxylate reductase 1, mitochondrial OS=Homo sapiens GN=PYCR1 PE=1 SV=2 | 5,11 | 5,25 | 4 | 18,5 |
| 1943 | sp|Q8IV08|PLD3_HUMAN | HUMAN | Phospholipase D3 OS=Homo sapiens GN=PLD3 PE=1 SV=1 | 5,11 | 5,25 | 3 | 6,327 |
| 1944 | sp|Q8N684|CPSF7_HUMAN | HUMAN | Cleavage and polyadenylation specificity factor subunit 7 OS=Homo sapiens GN=CPSF7 PE=1 SV=1 | 5,1 | 5,37 | 4 | 9,342 |
| 1945 | sp|O00115|DNS2A_HUMAN | HUMAN | Deoxyribonuclease-2-alpha OS=Homo sapiens GN=DNASE2 PE=1 SV=2 | 5,1 | 5,24 | 3 | 10 |
| 1946 | sp|Q9NX46|ARHL2_HUMAN | HUMAN | Poly(ADP-ribose) glycohydrolase ARH3 OS=Homo sapiens GN=ADPRHL2 PE=1 SV=1 | 5,07 | 5,17 | 3 | 11,85 |
| 1947 | sp|P09234|RU1C_HUMAN | HUMAN | U1 small nuclear ribonucleoprotein C OS=Homo sapiens GN=SNRPC PE=1 SV=1 | 5,07 | 5,11 | 3 | 24,53 |
| 1948 | sp|Q8TDP1|RNH2C_HUMAN | HUMAN | Ribonuclease H2 subunit C OS=Homo sapiens GN=RNASEH2C PE=1 SV=1 | 5,06 | 5,14 | 3 | 32,32 |
| 1949 | sp|Q9BT09|CNPY3_HUMAN | HUMAN | Protein canopy homolog 3 OS=Homo sapiens GN=CNPY3 PE=1 SV=1 | 5,05 | 5,2 | 4 | 17,27 |
| 1950 | sp|Q9C040|TRIM2_HUMAN | HUMAN | Tripartite motif-containing protein 2 OS=Homo sapiens GN=TRIM2 PE=1 SV=1 | 5,03 | 5,16 | 3 | 4,839 |
| 1951 | sp|P15586|GNS_HUMAN | HUMAN | N-acetylglucosamine-6-sulfatase OS=Homo sapiens GN=GNS PE=1 SV=3 | 5,03 | 5,13 | 3 | 3,804 |
| 1952 | sp|O43447|PPIH_HUMAN | HUMAN | Peptidyl-prolyl cis-trans isomerase H OS=Homo sapiens GN=PPIH PE=1 SV=1 | 5,02 | 5,57 | 4 | 22,03 |
| 1953 | sp|P05165|PCCA_HUMAN | HUMAN | Propionyl-CoA carboxylase alpha chain, mitochondrial OS=Homo sapiens GN=PCCA PE=1 SV=4 | 5,01 | 5,13 | 3 | 5,632 |
| 1954 | sp|P98179|RBM3_HUMAN | HUMAN | RNA-binding protein 3 OS=Homo sapiens GN=RBM3 PE=1 SV=1 | 5,01 | 5,09 | 6 | 47,77 |
| 1955 | sp|Q8IZ83|A16A1_HUMAN | HUMAN | Aldehyde dehydrogenase family 16 member A1 OS=Homo sapiens GN=ALDH16A1 PE=1 SV=2 | 5 | 5,12 | 3 | 5,237 |
| 1956 | sp|O43290|SNUT1_HUMAN | HUMAN | U4/U6.U5 tri-snRNP-associated protein 1 OS=Homo sapiens GN=SART1 PE=1 SV=1 | 5 | 5,1 | 3 | 4 |
| 1957 | sp|P55160|NCKPL_HUMAN | HUMAN | Nck-associated protein 1-like OS=Homo sapiens GN=NCKAP1L PE=1 SV=3 | 4,99 | 5,25 | 3 | 4,259 |
| 1958 | sp|Q9H4A6|GOLP3_HUMAN | HUMAN | Golgi phosphoprotein 3 OS=Homo sapiens GN=GOLPH3 PE=1 SV=1 | 4,99 | 5,05 | 4 | 22,15 |
| 1959 | sp|Q08623|HDHD1_HUMAN | HUMAN | Pseudouridine-5'-phosphatase OS=Homo sapiens GN=PUDP PE=1 SV=3 | 4,98 | 5,05 | 3 | 16,67 |
| 1960 | sp|Q99615|DNJC7_HUMAN | HUMAN | DnaJ homolog subfamily C member 7 OS=Homo sapiens GN=DNAJC7 PE=1 SV=2 | 4,95 | 5,27 | 5 | 13,36 |
| 1961 | sp|Q9HCC0|MCCB_HUMAN | HUMAN | Methylcrotonoyl-CoA carboxylase beta chain, mitochondrial OS=Homo sapiens GN=MCCC2 PE=1 SV=1 | 4,95 | 5,18 | 4 | 11,37 |
| 1962 | sp|O75400|PR40A_HUMAN | HUMAN | Pre-mRNA-processing factor 40 homolog A OS=Homo sapiens GN=PRPF40A PE=1 SV=2 | 4,95 | 5,09 | 3 | 3,971 |
| 1963 | sp|Q13243|SRSF5_HUMAN | HUMAN | Serine/arginine-rich splicing factor 5 OS=Homo sapiens GN=SRSF5 PE=1 SV=1 | 4,94 | 9,46 | 5 | 22,43 |
| 1964 | sp|P39059|COFA1_HUMAN | HUMAN | Collagen alpha-1(XV) chain OS=Homo sapiens GN=COL15A1 PE=1 SV=2 | 4,94 | 5,05 | 3 | 2,81 |
| 1965 | sp|O75915|PRAF3_HUMAN | HUMAN | PRA1 family protein 3 OS=Homo sapiens GN=ARL6IP5 PE=1 SV=1 | 4,94 | 4,99 | 4 | 19,68 |
| 1966 | sp|Q02487|DSC2_HUMAN | HUMAN | Desmocollin-2 OS=Homo sapiens GN=DSC2 PE=1 SV=1 | 4,93 | 5,05 | 3 | 5,993 |
| 1967 | sp|P62273|RS29_HUMAN | HUMAN | 40S ribosomal protein S29 OS=Homo sapiens GN=RPS29 PE=1 SV=2 | 4,93 | 5,05 | 4 | 58,93 |
| 1968 | sp|O95831|AIFM1_HUMAN | HUMAN | Apoptosis-inducing factor 1, mitochondrial OS=Homo sapiens GN=AIFM1 PE=1 SV=1 | 4,91 | 5,08 | 4 | 7,993 |
| 1969 | sp|Q96SZ5|AEDO_HUMAN | HUMAN | 2-aminoethanethiol dioxygenase OS=Homo sapiens GN=ADO PE=1 SV=2 | 4,9 | 5,12 | 3 | 16,3 |
| 1970 | sp|Q9UP83|COG5_HUMAN | HUMAN | Conserved oligomeric Golgi complex subunit 5 OS=Homo sapiens GN=COG5 PE=1 SV=3 | 4,88 | 5,25 | 3 | 4,768 |
| 1971 | sp|Q9BV57|MTND_HUMAN | HUMAN | 1,2-dihydroxy-3-keto-5-methylthiopentene dioxygenase OS=Homo sapiens GN=ADI1 PE=1 SV=1 | 4,88 | 5,08 | 5 | 30,17 |
| 1972 | sp|P36969|GPX4_HUMAN | HUMAN | Phospholipid hydroperoxide glutathione peroxidase, mitochondrial OS=Homo sapiens GN=GPX4 PE=1 SV=3 | 4,87 | 5,04 | 3 | 16,75 |
| 1973 | sp|Q13501|SQSTM_HUMAN | HUMAN | Sequestosome-1 OS=Homo sapiens GN=SQSTM1 PE=1 SV=1 | 4,86 | 4,91 | 3 | 11,36 |
| 1974 | sp|Q6UXN9|WDR82_HUMAN | HUMAN | WD repeat-containing protein 82 OS=Homo sapiens GN=WDR82 PE=1 SV=1 | 4,85 | 5,85 | 4 | 16,61 |
| 1975 | sp|P35232|PHB_HUMAN | HUMAN | Prohibitin OS=Homo sapiens GN=PHB PE=1 SV=1 | 4,85 | 5,14 | 3 | 17,28 |
| 1976 | sp|Q13573|SNW1_HUMAN | HUMAN | SNW domain-containing protein 1 OS=Homo sapiens GN=SNW1 PE=1 SV=1 | 4,85 | 5,04 | 4 | 10,07 |
| 1977 | sp|P07476|INVO_HUMAN | HUMAN | Involucrin OS=Homo sapiens GN=IVL PE=1 SV=2 | 4,85 | 4,93 | 4 | 10,09 |
| 1978 | sp|Q9Y4K1|AIM1_HUMAN | HUMAN | Absent in melanoma 1 protein OS=Homo sapiens GN=AIM1 PE=1 SV=3 | 4,84 | 5,07 | 5 | 4,063 |
| 1979 | sp|Q9UL18|AGO1_HUMAN | HUMAN | Protein argonaute-1 OS=Homo sapiens GN=AGO1 PE=1 SV=3 | 4,84 | 5 | 4 | 5,601 |
| 1980 | sp|O14964|HGS_HUMAN | HUMAN | Hepatocyte growth factor-regulated tyrosine kinase substrate OS=Homo sapiens GN=HGS PE=1 SV=1 | 4,84 | 4,98 | 3 | 4,376 |
| 1981 | sp|Q6PCE3|PGM2L_HUMAN | HUMAN | Glucose 1,6-bisphosphate synthase OS=Homo sapiens GN=PGM2L1 PE=1 SV=3 | 4,83 | 5,06 | 4 | 7,717 |
| 1982 | sp|P61966|AP1S1_HUMAN | HUMAN | AP-1 complex subunit sigma-1A OS=Homo sapiens GN=AP1S1 PE=1 SV=1 | 4,82 | 4,94 | 3 | 20,25 |
| 1983 | sp|P18859|ATP5J_HUMAN | HUMAN | ATP synthase-coupling factor 6, mitochondrial OS=Homo sapiens GN=ATP5J PE=1 SV=1 | 4,81 | 4,92 | 4 | 30,56 |
| 1984 | sp|P17050|NAGAB_HUMAN | HUMAN | Alpha-N-acetylgalactosaminidase OS=Homo sapiens GN=NAGA PE=1 SV=2 | 4,81 | 4,87 | 3 | 8,273 |
| 1985 | sp|O95479|G6PE_HUMAN | HUMAN | GDH/6PGL endoplasmic bifunctional protein OS=Homo sapiens GN=H6PD PE=1 SV=2 | 4,8 | 5,11 | 4 | 5,942 |
| 1986 | sp|O60264|SMCA5_HUMAN | HUMAN | SWI/SNF-related matrix-associated actin-dependent regulator of chromatin subfamily A member 5 OS=Homo sapiens GN=SMARCA5 PE=1 SV=1 | 4,79 | 5,08 | 4 | 3,517 |
| 1987 | sp|P53602|MVD1_HUMAN | HUMAN | Diphosphomevalonate decarboxylase OS=Homo sapiens GN=MVD PE=1 SV=1 | 4,78 | 4,91 | 3 | 11 |
| 1988 | sp|O95456|PSMG1_HUMAN | HUMAN | Proteasome assembly chaperone 1 OS=Homo sapiens GN=PSMG1 PE=1 SV=1 | 4,77 | 4,89 | 4 | 15,97 |
| 1989 | sp|O60936|NOL3_HUMAN | HUMAN | Nucleolar protein 3 OS=Homo sapiens GN=NOL3 PE=1 SV=2 | 4,77 | 4,86 | 3 | 26,44 |
| 1990 | sp|P54105|ICLN_HUMAN | HUMAN | Methylosome subunit pICln OS=Homo sapiens GN=CLNS1A PE=1 SV=1 | 4,75 | 4,8 | 4 | 27,85 |
| 1991 | sp|Q0ZGT2|NEXN_HUMAN | HUMAN | Nexilin OS=Homo sapiens GN=NEXN PE=1 SV=1 | 4,74 | 4,98 | 3 | 4,296 |
| 1992 | sp|P04208|LV106_HUMAN | HUMAN | Ig lambda chain V-I region WAH OS=Homo sapiens PE=1 SV=1 | 4,74 | 4,78 | 3 | 37,61 |
| 1993 | sp|Q9P265|DIP2B_HUMAN | HUMAN | Disco-interacting protein 2 homolog B OS=Homo sapiens GN=DIP2B PE=1 SV=3 | 4,73 | 4,86 | 3 | 2,919 |
| 1994 | sp|O15116|LSM1_HUMAN | HUMAN | U6 snRNA-associated Sm-like protein LSm1 OS=Homo sapiens GN=LSM1 PE=1 SV=1 | 4,72 | 4,8 | 3 | 30,83 |
| 1995 | sp|P09110|THIK_HUMAN | HUMAN | 3-ketoacyl-CoA thiolase, peroxisomal OS=Homo sapiens GN=ACAA1 PE=1 SV=2 | 4,71 | 4,88 | 3 | 14,39 |
| 1996 | sp|Q7Z2Z2|ETUD1_HUMAN | HUMAN | Elongation factor Tu GTP-binding domain-containing protein 1 OS=Homo sapiens GN=EFTUD1 PE=1 SV=2 | 4,68 | 5,38 | 4 | 6,071 |
| 1997 | sp|P06280|AGAL_HUMAN | HUMAN | Alpha-galactosidase A OS=Homo sapiens GN=GLA PE=1 SV=1 | 4,67 | 4,88 | 3 | 8,392 |
| 1998 | sp|Q9UI08|EVL_HUMAN | HUMAN | Ena/VASP-like protein OS=Homo sapiens GN=EVL PE=1 SV=2 | 4,67 | 4,82 | 3 | 10,1 |
| 1999 | sp|Q99933|BAG1_HUMAN | HUMAN | BAG family molecular chaperone regulator 1 OS=Homo sapiens GN=BAG1 PE=1 SV=4 | 4,67 | 4,77 | 3 | 9,855 |
| 2000 | sp|Q13555|KCC2G_HUMAN | HUMAN | Calcium/calmodulin-dependent protein kinase type II subunit gamma OS=Homo sapiens GN=CAMK2G PE=1 SV=3 | 4,66 | 8,57 | 6 | 11,83 |
| 2001 | sp|O60502|OGA_HUMAN | HUMAN | Protein O-GlcNAcase OS=Homo sapiens GN=MGEA5 PE=1 SV=2 | 4,65 | 4,78 | 3 | 5,022 |
| 2002 | sp|P11279|LAMP1_HUMAN | HUMAN | Lysosome-associated membrane glycoprotein 1 OS=Homo sapiens GN=LAMP1 PE=1 SV=3 | 4,65 | 4,71 | 5 | 5,995 |
| 2003 | sp|Q13526|PIN1_HUMAN | HUMAN | Peptidyl-prolyl cis-trans isomerase NIMA-interacting 1 OS=Homo sapiens GN=PIN1 PE=1 SV=1 | 4,65 | 4,68 | 4 | 30,06 |
| 2004 | sp|P28331|NDUS1_HUMAN | HUMAN | NADH-ubiquinone oxidoreductase 75 kDa subunit, mitochondrial OS=Homo sapiens GN=NDUFS1 PE=1 SV=3 | 4,64 | 4,75 | 3 | 8,391 |
| 2005 | sp|P01893|HLAH_HUMAN | HUMAN | Putative HLA class I histocompatibility antigen, alpha chain H OS=Homo sapiens GN=HLA-H PE=5 SV=3 | 4,63 | 15,16 | 8 | 27,35 |
| 2006 | sp|O00264|PGRC1_HUMAN | HUMAN | Membrane-associated progesterone receptor component 1 OS=Homo sapiens GN=PGRMC1 PE=1 SV=3 | 4,63 | 6,47 | 4 | 31,79 |
| 2007 | sp|Q16864|VATF_HUMAN | HUMAN | V-type proton ATPase subunit F OS=Homo sapiens GN=ATP6V1F PE=1 SV=2 | 4,63 | 4,72 | 3 | 33,61 |
| 2008 | sp|P32321|DCTD_HUMAN | HUMAN | Deoxycytidylate deaminase OS=Homo sapiens GN=DCTD PE=1 SV=2 | 4,62 | 4,72 | 3 | 22,47 |
| 2009 | sp|Q9P287|BCCIP_HUMAN | HUMAN | BRCA2 and CDKN1A-interacting protein OS=Homo sapiens GN=BCCIP PE=1 SV=1 | 4,62 | 4,66 | 4 | 16,88 |
| 2010 | sp|P61020|RAB5B_HUMAN | HUMAN | Ras-related protein Rab-5B OS=Homo sapiens GN=RAB5B PE=1 SV=1 | 4,61 | 10,57 | 7 | 33,49 |
| 2011 | sp|P32320|CDD_HUMAN | HUMAN | Cytidine deaminase OS=Homo sapiens GN=CDA PE=1 SV=2 | 4,61 | 4,64 | 4 | 41,78 |
| 2012 | sp|Q14112|NID2_HUMAN | HUMAN | Nidogen-2 OS=Homo sapiens GN=NID2 PE=1 SV=3 | 4,6 | 4,63 | 2 | 2,909 |
| 2013 | sp|Q9UK45|LSM7_HUMAN | HUMAN | U6 snRNA-associated Sm-like protein LSm7 OS=Homo sapiens GN=LSM7 PE=1 SV=1 | 4,59 | 4,71 | 4 | 40,78 |
| 2014 | sp|Q53GS9|SNUT2_HUMAN | HUMAN | U4/U6.U5 tri-snRNP-associated protein 2 OS=Homo sapiens GN=USP39 PE=1 SV=2 | 4,58 | 5,62 | 4 | 9,204 |
| 2015 | sp|P13473|LAMP2_HUMAN | HUMAN | Lysosome-associated membrane glycoprotein 2 OS=Homo sapiens GN=LAMP2 PE=1 SV=2 | 4,58 | 4,66 | 4 | 8,293 |
| 2016 | sp|Q15113|PCOC1_HUMAN | HUMAN | Procollagen C-endopeptidase enhancer 1 OS=Homo sapiens GN=PCOLCE PE=1 SV=2 | 4,58 | 4,61 | 3 | 9,354 |
| 2017 | sp|Q15427|SF3B4_HUMAN | HUMAN | Splicing factor 3B subunit 4 OS=Homo sapiens GN=SF3B4 PE=1 SV=1 | 4,58 | 4,61 | 3 | 9,434 |
| 2018 | sp|Q9C005|DPY30_HUMAN | HUMAN | Protein dpy-30 homolog OS=Homo sapiens GN=DPY30 PE=1 SV=1 | 4,58 | 4,61 | 3 | 27,27 |
| 2019 | sp|Q9HAV4|XPO5_HUMAN | HUMAN | Exportin-5 OS=Homo sapiens GN=XPO5 PE=1 SV=1 | 4,56 | 4,99 | 3 | 2,99 |
| 2020 | sp|Q13616|CUL1_HUMAN | HUMAN | Cullin-1 OS=Homo sapiens GN=CUL1 PE=1 SV=2 | 4,56 | 4,85 | 3 | 5,284 |
| 2021 | sp|P08567|PLEK_HUMAN | HUMAN | Pleckstrin OS=Homo sapiens GN=PLEK PE=1 SV=3 | 4,55 | 4,63 | 2 | 10,86 |
| 2022 | sp|Q9BXN1|ASPN_HUMAN | HUMAN | Asporin OS=Homo sapiens GN=ASPN PE=1 SV=2 | 4,55 | 4,63 | 3 | 12,89 |
| 2023 | sp|Q9NX24|NHP2_HUMAN | HUMAN | H/ACA ribonucleoprotein complex subunit 2 OS=Homo sapiens GN=NHP2 PE=1 SV=1 | 4,55 | 4,63 | 3 | 31,37 |
| 2024 | sp|P15529|MCP_HUMAN | HUMAN | Membrane cofactor protein OS=Homo sapiens GN=CD46 PE=1 SV=3 | 4,54 | 4,62 | 4 | 11,99 |
| 2025 | sp|Q14108|SCRB2_HUMAN | HUMAN | Lysosome membrane protein 2 OS=Homo sapiens GN=SCARB2 PE=1 SV=2 | 4,53 | 4,61 | 3 | 7,322 |
| 2026 | sp|Q9BUT1|BDH2_HUMAN | HUMAN | 3-hydroxybutyrate dehydrogenase type 2 OS=Homo sapiens GN=BDH2 PE=1 SV=2 | 4,52 | 5,01 | 3 | 12,65 |
| 2027 | sp|P53680|AP2S1_HUMAN | HUMAN | AP-2 complex subunit sigma OS=Homo sapiens GN=AP2S1 PE=1 SV=2 | 4,52 | 4,63 | 4 | 16,9 |
| 2028 | sp|Q9Y6M1|IF2B2_HUMAN | HUMAN | Insulin-like growth factor 2 mRNA-binding protein 2 OS=Homo sapiens GN=IGF2BP2 PE=1 SV=2 | 4,5 | 4,61 | 3 | 7,179 |
| 2029 | sp|P19474|RO52_HUMAN | HUMAN | E3 ubiquitin-protein ligase TRIM21 OS=Homo sapiens GN=TRIM21 PE=1 SV=1 | 4,5 | 4,59 | 3 | 7,368 |
| 2030 | sp|Q9Y277|VDAC3_HUMAN | HUMAN | Voltage-dependent anion-selective channel protein 3 OS=Homo sapiens GN=VDAC3 PE=1 SV=1 | 4,48 | 6,55 | 4 | 18,02 |
| 2031 | sp|Q9H446|RWDD1_HUMAN | HUMAN | RWD domain-containing protein 1 OS=Homo sapiens GN=RWDD1 PE=1 SV=1 | 4,48 | 4,97 | 3 | 21,4 |
| 2032 | sp|P46013|KI67_HUMAN | HUMAN | Antigen KI-67 OS=Homo sapiens GN=MKI67 PE=1 SV=2 | 4,46 | 4,76 | 3 | 1,935 |
| 2033 | sp|Q96HY6|DDRGK_HUMAN | HUMAN | DDRGK domain-containing protein 1 OS=Homo sapiens GN=DDRGK1 PE=1 SV=2 | 4,44 | 4,52 | 2 | 11,15 |
| 2034 | sp|Q9NR56|MBNL1_HUMAN | HUMAN | Muscleblind-like protein 1 OS=Homo sapiens GN=MBNL1 PE=1 SV=2 | 4,44 | 4,52 | 3 | 9,794 |
| 2035 | sp|O95352|ATG7_HUMAN | HUMAN | Ubiquitin-like modifier-activating enzyme ATG7 OS=Homo sapiens GN=ATG7 PE=1 SV=1 | 4,44 | 4,47 | 3 | 3,983 |
| 2036 | sp|A6NDG6|PGP_HUMAN | HUMAN | Phosphoglycolate phosphatase OS=Homo sapiens GN=PGP PE=1 SV=1 | 4,43 | 4,46 | 2 | 9,034 |
| 2037 | sp|P84085|ARF5_HUMAN | HUMAN | ADP-ribosylation factor 5 OS=Homo sapiens GN=ARF5 PE=1 SV=2 | 4,42 | 16,64 | 24 | 58,89 |
| 2038 | sp|Q00169|PIPNA_HUMAN | HUMAN | Phosphatidylinositol transfer protein alpha isoform OS=Homo sapiens GN=PITPNA PE=1 SV=2 | 4,42 | 9,81 | 7 | 24,81 |
| 2039 | sp|Q9UKG1|DP13A_HUMAN | HUMAN | DCC-interacting protein 13-alpha OS=Homo sapiens GN=APPL1 PE=1 SV=1 | 4,41 | 4,84 | 3 | 5,078 |
| 2040 | sp|Q9BZE9|ASPC1_HUMAN | HUMAN | Tether containing UBX domain for GLUT4 OS=Homo sapiens GN=ASPSCR1 PE=1 SV=1 | 4,41 | 4,45 | 2 | 5,787 |
| 2041 | sp|P10620|MGST1_HUMAN | HUMAN | Microsomal glutathione S-transferase 1 OS=Homo sapiens GN=MGST1 PE=1 SV=1 | 4,41 | 4,43 | 2 | 18,71 |
| 2042 | sp|P45877|PPIC_HUMAN | HUMAN | Peptidyl-prolyl cis-trans isomerase C OS=Homo sapiens GN=PPIC PE=1 SV=1 | 4,39 | 4,58 | 3 | 14,15 |
| 2043 | sp|Q86Y82|STX12_HUMAN | HUMAN | Syntaxin-12 OS=Homo sapiens GN=STX12 PE=1 SV=1 | 4,38 | 4,48 | 4 | 17,75 |
| 2044 | sp|Q86VN1|VPS36_HUMAN | HUMAN | Vacuolar protein-sorting-associated protein 36 OS=Homo sapiens GN=VPS36 PE=1 SV=1 | 4,38 | 4,4 | 4 | 8,808 |
| 2045 | sp|Q9UBI6|GBG12_HUMAN | HUMAN | Guanine nucleotide-binding protein G(I)/G(S)/G(O) subunit gamma-12 OS=Homo sapiens GN=GNG12 PE=1 SV=3 | 4,38 | 4,4 | 2 | 37,5 |
| 2046 | sp|P35813|PPM1A_HUMAN | HUMAN | Protein phosphatase 1A OS=Homo sapiens GN=PPM1A PE=1 SV=1 | 4,37 | 4,53 | 4 | 12,3 |
| 2047 | sp|Q9UPT8|ZC3H4_HUMAN | HUMAN | Zinc finger CCCH domain-containing protein 4 OS=Homo sapiens GN=ZC3H4 PE=1 SV=3 | 4,36 | 4,48 | 4 | 4,144 |
| 2048 | sp|P02655|APOC2_HUMAN | HUMAN | Apolipoprotein C-II OS=Homo sapiens GN=APOC2 PE=1 SV=1 | 4,36 | 4,48 | 3 | 38,61 |
| 2049 | sp|O43665|RGS10_HUMAN | HUMAN | Regulator of G-protein signaling 10 OS=Homo sapiens GN=RGS10 PE=1 SV=2 | 4,34 | 4,42 | 3 | 26,01 |
| 2050 | sp|P04732|MT1E_HUMAN | HUMAN | Metallothionein-1E OS=Homo sapiens GN=MT1E PE=1 SV=1 | 4,34 | 4,41 | 4 | 34,43 |
| 2051 | sp|Q16774|KGUA_HUMAN | HUMAN | Guanylate kinase OS=Homo sapiens GN=GUK1 PE=1 SV=2 | 4,34 | 4,36 | 2 | 17,26 |
| 2052 | sp|Q86Y56|DAAF5_HUMAN | HUMAN | Dynein assembly factor 5, axonemal OS=Homo sapiens GN=DNAAF5 PE=1 SV=4 | 4,33 | 4,44 | 3 | 4,795 |
| 2053 | sp|Q9GZS3|WDR61_HUMAN | HUMAN | WD repeat-containing protein 61 OS=Homo sapiens GN=WDR61 PE=1 SV=1 | 4,33 | 4,43 | 3 | 16,07 |
| 2054 | sp|P08174|DAF_HUMAN | HUMAN | Complement decay-accelerating factor OS=Homo sapiens GN=CD55 PE=1 SV=4 | 4,33 | 4,42 | 3 | 9,186 |
| 2055 | sp|Q9NP72|RAB18_HUMAN | HUMAN | Ras-related protein Rab-18 OS=Homo sapiens GN=RAB18 PE=1 SV=1 | 4,33 | 4,35 | 2 | 14,56 |
| 2056 | sp|P61006|RAB8A_HUMAN | HUMAN | Ras-related protein Rab-8A OS=Homo sapiens GN=RAB8A PE=1 SV=1 | 4,31 | 9,65 | 11 | 38,16 |
| 2057 | sp|P09543|CN37_HUMAN | HUMAN | 2',3'-cyclic-nucleotide 3'-phosphodiesterase OS=Homo sapiens GN=CNP PE=1 SV=2 | 4,31 | 4,77 | 4 | 14,25 |
| 2058 | sp|P23229|ITA6_HUMAN | HUMAN | Integrin alpha-6 OS=Homo sapiens GN=ITGA6 PE=1 SV=5 | 4,31 | 4,42 | 4 | 3,097 |
| 2059 | sp|O76038|SEGN_HUMAN | HUMAN | Secretagogin OS=Homo sapiens GN=SCGN PE=2 SV=2 | 4,29 | 4,46 | 4 | 17,03 |
| 2060 | sp|Q6IAA8|LTOR1_HUMAN | HUMAN | Ragulator complex protein LAMTOR1 OS=Homo sapiens GN=LAMTOR1 PE=1 SV=2 | 4,29 | 4,45 | 3 | 27,33 |
| 2061 | sp|Q15286|RAB35_HUMAN | HUMAN | Ras-related protein Rab-35 OS=Homo sapiens GN=RAB35 PE=1 SV=1 | 4,28 | 8,42 | 8 | 24,38 |
| 2062 | sp|Q9UJC5|SH3L2_HUMAN | HUMAN | SH3 domain-binding glutamic acid-rich-like protein 2 OS=Homo sapiens GN=SH3BGRL2 PE=1 SV=2 | 4,27 | 4,29 | 4 | 40,19 |
| 2063 | sp|Q9BYZ8|REG4_HUMAN | HUMAN | Regenerating islet-derived protein 4 OS=Homo sapiens GN=REG4 PE=1 SV=1 | 4,27 | 4,28 | 3 | 17,09 |
| 2064 | sp|P04207|KV308_HUMAN | HUMAN | Ig kappa chain V-III region CLL OS=Homo sapiens PE=4 SV=2 | 4,26 | 6,45 | 4 | 34,11 |
| 2065 | sp|Q07157|ZO1_HUMAN | HUMAN | Tight junction protein ZO-1 OS=Homo sapiens GN=TJP1 PE=1 SV=3 | 4,26 | 4,38 | 3 | 1,831 |
| 2066 | sp|O60220|TIM8A_HUMAN | HUMAN | Mitochondrial import inner membrane translocase subunit Tim8 A OS=Homo sapiens GN=TIMM8A PE=1 SV=1 | 4,26 | 4,28 | 4 | 52,58 |
| 2067 | sp|P27169|PON1_HUMAN | HUMAN | Serum paraoxonase/arylesterase 1 OS=Homo sapiens GN=PON1 PE=1 SV=3 | 4,25 | 4,3 | 4 | 7,324 |
| 2068 | sp|Q9Y4Y9|LSM5_HUMAN | HUMAN | U6 snRNA-associated Sm-like protein LSm5 OS=Homo sapiens GN=LSM5 PE=1 SV=3 | 4,24 | 4,31 | 6 | 52,75 |
| 2069 | sp|P15088|CBPA3_HUMAN | HUMAN | Mast cell carboxypeptidase A OS=Homo sapiens GN=CPA3 PE=1 SV=2 | 4,24 | 4,26 | 2 | 5,516 |
| 2070 | sp|P51003|PAPOA_HUMAN | HUMAN | Poly(A) polymerase alpha OS=Homo sapiens GN=PAPOLA PE=1 SV=4 | 4,22 | 4,34 | 2 | 4,966 |
| 2071 | sp|Q9BXW7|CECR5_HUMAN | HUMAN | Cat eye syndrome critical region protein 5 OS=Homo sapiens GN=CECR5 PE=1 SV=1 | 4,22 | 4,28 | 2 | 6,619 |
| 2072 | sp|Q9BTY7|HGH1_HUMAN | HUMAN | Protein HGH1 homolog OS=Homo sapiens GN=HGH1 PE=1 SV=1 | 4,19 | 4,36 | 3 | 17,69 |
| 2073 | sp|O00139|KIF2A_HUMAN | HUMAN | Kinesin-like protein KIF2A OS=Homo sapiens GN=KIF2A PE=1 SV=3 | 4,19 | 4,28 | 3 | 4,249 |
| 2074 | sp|Q13144|EI2BE_HUMAN | HUMAN | Translation initiation factor eIF-2B subunit epsilon OS=Homo sapiens GN=EIF2B5 PE=1 SV=3 | 4,19 | 4,27 | 3 | 4,161 |
| 2075 | sp|Q9BW04|SARG_HUMAN | HUMAN | Specifically androgen-regulated gene protein OS=Homo sapiens GN=SARG PE=1 SV=2 | 4,19 | 4,22 | 2 | 6,489 |
| 2076 | sp|Q9UGM3|DMBT1_HUMAN | HUMAN | Deleted in malignant brain tumors 1 protein OS=Homo sapiens GN=DMBT1 PE=1 SV=2 | 4,19 | 4,2 | 2 | 10,69 |
| 2077 | sp|Q12797|ASPH_HUMAN | HUMAN | Aspartyl/asparaginyl beta-hydroxylase OS=Homo sapiens GN=ASPH PE=1 SV=3 | 4,18 | 4,27 | 3 | 5,145 |
| 2078 | sp|Q9C0B1|FTO_HUMAN | HUMAN | Alpha-ketoglutarate-dependent dioxygenase FTO OS=Homo sapiens GN=FTO PE=1 SV=3 | 4,17 | 4,25 | 3 | 6,931 |
| 2079 | sp|Q08170|SRSF4_HUMAN | HUMAN | Serine/arginine-rich splicing factor 4 OS=Homo sapiens GN=SRSF4 PE=1 SV=2 | 4,16 | 12,99 | 9 | 15,99 |
| 2080 | sp|Q15819|UB2V2_HUMAN | HUMAN | Ubiquitin-conjugating enzyme E2 variant 2 OS=Homo sapiens GN=UBE2V2 PE=1 SV=4 | 4,16 | 10,31 | 7 | 33,1 |
| 2081 | sp|P08575|PTPRC_HUMAN | HUMAN | Receptor-type tyrosine-protein phosphatase C OS=Homo sapiens GN=PTPRC PE=1 SV=2 | 4,16 | 4,31 | 3 | 3,221 |
| 2082 | sp|Q5T5P2|SKT_HUMAN | HUMAN | Sickle tail protein homolog OS=Homo sapiens GN=KIAA1217 PE=1 SV=2 | 4,16 | 4,3 | 2 | 1,132 |
| 2083 | sp|P15289|ARSA_HUMAN | HUMAN | Arylsulfatase A OS=Homo sapiens GN=ARSA PE=1 SV=3 | 4,16 | 4,17 | 2 | 8,087 |
| 2084 | sp|P48059|LIMS1_HUMAN | HUMAN | LIM and senescent cell antigen-like-containing domain protein 1 OS=Homo sapiens GN=LIMS1 PE=1 SV=4 | 4,14 | 12 | 7 | 21,85 |
| 2085 | sp|O00423|EMAL1_HUMAN | HUMAN | Echinoderm microtubule-associated protein-like 1 OS=Homo sapiens GN=EML1 PE=1 SV=3 | 4,14 | 4,79 | 4 | 5,031 |
| 2086 | sp|Q9H2J4|PDCL3_HUMAN | HUMAN | Phosducin-like protein 3 OS=Homo sapiens GN=PDCL3 PE=1 SV=1 | 4,14 | 4,27 | 3 | 17,15 |
| 2087 | sp|P41227|NAA10_HUMAN | HUMAN | N-alpha-acetyltransferase 10 OS=Homo sapiens GN=NAA10 PE=1 SV=1 | 4,14 | 4,15 | 2 | 11,49 |
| 2088 | sp|P01610|KV118_HUMAN | HUMAN | Ig kappa chain V-I region WEA OS=Homo sapiens PE=1 SV=1 | 4,13 | 4,14 | 10 | 37,04 |
| 2089 | sp|P51397|DAP1_HUMAN | HUMAN | Death-associated protein 1 OS=Homo sapiens GN=DAP PE=1 SV=3 | 4,13 | 4,14 | 2 | 18,63 |
| 2090 | sp|Q7KZI7|MARK2_HUMAN | HUMAN | Serine/threonine-protein kinase MARK2 OS=Homo sapiens GN=MARK2 PE=1 SV=2 | 4,12 | 4,19 | 2 | 3,553 |
| 2091 | sp|Q15843|NEDD8_HUMAN | HUMAN | NEDD8 OS=Homo sapiens GN=NEDD8 PE=1 SV=1 | 4,12 | 4,13 | 2 | 17,28 |
| 2092 | sp|P07225|PROS_HUMAN | HUMAN | Vitamin K-dependent protein S OS=Homo sapiens GN=PROS1 PE=1 SV=1 | 4,11 | 5,15 | 4 | 4,882 |
| 2093 | sp|Q12888|TP53B_HUMAN | HUMAN | Tumor suppressor p53-binding protein 1 OS=Homo sapiens GN=TP53BP1 PE=1 SV=2 | 4,11 | 4,26 | 3 | 2,231 |
| 2094 | sp|O75934|SPF27_HUMAN | HUMAN | Pre-mRNA-splicing factor SPF27 OS=Homo sapiens GN=BCAS2 PE=1 SV=1 | 4,11 | 4,2 | 3 | 20,44 |
| 2095 | sp|P05114|HMGN1_HUMAN | HUMAN | Non-histone chromosomal protein HMG-14 OS=Homo sapiens GN=HMGN1 PE=1 SV=3 | 4,11 | 4,16 | 3 | 34 |
| 2096 | sp|P47914|RL29_HUMAN | HUMAN | 60S ribosomal protein L29 OS=Homo sapiens GN=RPL29 PE=1 SV=2 | 4,11 | 4,12 | 2 | 14,47 |
| 2097 | sp|Q8WW59|SPRY4_HUMAN | HUMAN | SPRY domain-containing protein 4 OS=Homo sapiens GN=SPRYD4 PE=1 SV=2 | 4,1 | 4,22 | 2 | 10,14 |
| 2098 | sp|Q13158|FADD_HUMAN | HUMAN | FAS-associated death domain protein OS=Homo sapiens GN=FADD PE=1 SV=1 | 4,09 | 4,25 | 2 | 14,9 |
| 2099 | sp|Q9P2B2|FPRP_HUMAN | HUMAN | Prostaglandin F2 receptor negative regulator OS=Homo sapiens GN=PTGFRN PE=1 SV=2 | 4,09 | 4,19 | 3 | 3,641 |
| 2100 | sp|Q96GX9|MTNB_HUMAN | HUMAN | Methylthioribulose-1-phosphate dehydratase OS=Homo sapiens GN=APIP PE=1 SV=1 | 4,09 | 4,1 | 2 | 8,264 |
| 2101 | sp|Q01524|DEF6_HUMAN | HUMAN | Defensin-6 OS=Homo sapiens GN=DEFA6 PE=1 SV=1 | 4,09 | 4,1 | 3 | 43 |
| 2102 | sp|Q9UKD2|MRT4_HUMAN | HUMAN | mRNA turnover protein 4 homolog OS=Homo sapiens GN=MRTO4 PE=1 SV=2 | 4,08 | 4,21 | 3 | 14,23 |
| 2103 | sp|P49755|TMEDA_HUMAN | HUMAN | Transmembrane emp24 domain-containing protein 10 OS=Homo sapiens GN=TMED10 PE=1 SV=2 | 4,08 | 4,12 | 2 | 9,132 |
| 2104 | sp|O15446|RPA34_HUMAN | HUMAN | DNA-directed RNA polymerase I subunit RPA34 OS=Homo sapiens GN=CD3EAP PE=1 SV=1 | 4,06 | 4,07 | 2 | 10,39 |
| 2105 | sp|Q04726|TLE3_HUMAN | HUMAN | Transducin-like enhancer protein 3 OS=Homo sapiens GN=TLE3 PE=1 SV=2 | 4,06 | 4,06 | 2 | 2,332 |
| 2106 | sp|O60645|EXOC3_HUMAN | HUMAN | Exocyst complex component 3 OS=Homo sapiens GN=EXOC3 PE=1 SV=2 | 4,05 | 4,23 | 3 | 4,894 |
| 2107 | sp|Q92696|PGTA_HUMAN | HUMAN | Geranylgeranyl transferase type-2 subunit alpha OS=Homo sapiens GN=RABGGTA PE=1 SV=2 | 4,05 | 4,18 | 3 | 9,171 |
| 2108 | sp|P47895|AL1A3_HUMAN | HUMAN | Aldehyde dehydrogenase family 1 member A3 OS=Homo sapiens GN=ALDH1A3 PE=1 SV=2 | 4,04 | 7,7 | 4 | 8,008 |
| 2109 | sp|Q92876|KLK6_HUMAN | HUMAN | Kallikrein-6 OS=Homo sapiens GN=KLK6 PE=1 SV=1 | 4,04 | 4,18 | 4 | 25,82 |
| 2110 | sp|Q10713|MPPA_HUMAN | HUMAN | Mitochondrial-processing peptidase subunit alpha OS=Homo sapiens GN=PMPCA PE=1 SV=2 | 4,04 | 4,14 | 3 | 8,762 |
| 2111 | sp|Q96QA5|GSDMA_HUMAN | HUMAN | Gasdermin-A OS=Homo sapiens GN=GSDMA PE=1 SV=4 | 4,04 | 4,12 | 2 | 8,989 |
| 2112 | sp|O43760|SNG2_HUMAN | HUMAN | Synaptogyrin-2 OS=Homo sapiens GN=SYNGR2 PE=1 SV=1 | 4,04 | 4,09 | 2 | 8,929 |
| 2113 | sp|O15382|BCAT2_HUMAN | HUMAN | Branched-chain-amino-acid aminotransferase, mitochondrial OS=Homo sapiens GN=BCAT2 PE=1 SV=2 | 4,04 | 4,06 | 2 | 8,163 |
| 2114 | sp|Q7Z4H3|HDDC2_HUMAN | HUMAN | HD domain-containing protein 2 OS=Homo sapiens GN=HDDC2 PE=1 SV=1 | 4,04 | 4,05 | 3 | 14,71 |
| 2115 | sp|P02656|APOC3_HUMAN | HUMAN | Apolipoprotein C-III OS=Homo sapiens GN=APOC3 PE=1 SV=1 | 4,04 | 4,04 | 2 | 27,27 |
| 2116 | sp|P60891|PRPS1_HUMAN | HUMAN | Ribose-phosphate pyrophosphokinase 1 OS=Homo sapiens GN=PRPS1 PE=1 SV=2 | 4,03 | 15,64 | 12 | 38,36 |
| 2117 | sp|P49747|COMP_HUMAN | HUMAN | Cartilage oligomeric matrix protein OS=Homo sapiens GN=COMP PE=1 SV=2 | 4,03 | 4,16 | 3 | 6,473 |
| 2118 | sp|P50583|AP4A_HUMAN | HUMAN | Bis(5'-nucleosyl)-tetraphosphatase [asymmetrical] OS=Homo sapiens GN=NUDT2 PE=1 SV=3 | 4,03 | 4,09 | 2 | 21,77 |
| 2119 | sp|Q8TF74|WIPF2_HUMAN | HUMAN | WAS/WASL-interacting protein family member 2 OS=Homo sapiens GN=WIPF2 PE=1 SV=1 | 4,03 | 4,06 | 2 | 7,273 |
| 2120 | sp|P30049|ATPD_HUMAN | HUMAN | ATP synthase subunit delta, mitochondrial OS=Homo sapiens GN=ATP5D PE=1 SV=2 | 4,03 | 4,04 | 2 | 13,69 |
| 2121 | sp|P62314|SMD1_HUMAN | HUMAN | Small nuclear ribonucleoprotein Sm D1 OS=Homo sapiens GN=SNRPD1 PE=1 SV=1 | 4,03 | 4,03 | 2 | 20,17 |
| 2122 | sp|P24928|RPB1_HUMAN | HUMAN | DNA-directed RNA polymerase II subunit RPB1 OS=Homo sapiens GN=POLR2A PE=1 SV=2 | 4,02 | 4,11 | 2 | 2,132 |
| 2123 | sp|Q6UN15|FIP1_HUMAN | HUMAN | Pre-mRNA 3'-end-processing factor FIP1 OS=Homo sapiens GN=FIP1L1 PE=1 SV=1 | 4,02 | 4,02 | 2 | 6,902 |
| 2124 | sp|P21926|CD9_HUMAN | HUMAN | CD9 antigen OS=Homo sapiens GN=CD9 PE=1 SV=4 | 4,02 | 4,02 | 2 | 15,35 |
| 2125 | sp|O43252|PAPS1_HUMAN | HUMAN | Bifunctional 3'-phosphoadenosine 5'-phosphosulfate synthase 1 OS=Homo sapiens GN=PAPSS1 PE=1 SV=2 | 4,01 | 5,64 | 4 | 10,26 |
| 2126 | sp|Q13405|RM49_HUMAN | HUMAN | 39S ribosomal protein L49, mitochondrial OS=Homo sapiens GN=MRPL49 PE=1 SV=1 | 4,01 | 4,14 | 2 | 15,66 |
| 2127 | sp|P51649|SSDH_HUMAN | HUMAN | Succinate-semialdehyde dehydrogenase, mitochondrial OS=Homo sapiens GN=ALDH5A1 PE=1 SV=2 | 4,01 | 4,09 | 3 | 9,72 |
| 2128 | sp|Q16718|NDUA5_HUMAN | HUMAN | NADH dehydrogenase [ubiquinone] 1 alpha subcomplex subunit 5 OS=Homo sapiens GN=NDUFA5 PE=1 SV=3 | 4,01 | 4,01 | 2 | 31,03 |
| 2129 | sp|Q9UHA4|LTOR3_HUMAN | HUMAN | Ragulator complex protein LAMTOR3 OS=Homo sapiens GN=LAMTOR3 PE=1 SV=1 | 4,01 | 4,01 | 2 | 30,65 |
| 2130 | sp|Q9NPJ3|ACO13_HUMAN | HUMAN | Acyl-coenzyme A thioesterase 13 OS=Homo sapiens GN=ACOT13 PE=1 SV=1 | 4,01 | 4,01 | 2 | 15,71 |
| 2131 | sp|Q04828|AK1C1_HUMAN | HUMAN | Aldo-keto reductase family 1 member C1 OS=Homo sapiens GN=AKR1C1 PE=1 SV=1 | 4 | 18,79 | 12 | 37,15 |
| 2132 | sp|P0CG06|LAC3_HUMAN | HUMAN | Ig lambda-3 chain C regions OS=Homo sapiens GN=IGLC3 PE=1 SV=1 | 4 | 15,64 | 27 | 88,68 |
| 2133 | sp|Q9BQ67|GRWD1_HUMAN | HUMAN | Glutamate-rich WD repeat-containing protein 1 OS=Homo sapiens GN=GRWD1 PE=1 SV=1 | 4 | 4,86 | 4 | 8,969 |
| 2134 | sp|A6NHR9|SMHD1_HUMAN | HUMAN | Structural maintenance of chromosomes flexible hinge domain-containing protein 1 OS=Homo sapiens GN=SMCHD1 PE=1 SV=2 | 4 | 4,19 | 2 | 1,047 |
| 2135 | sp|P01602|KV110_HUMAN | HUMAN | Ig heavy chain V-I region 5 (Fragment) OS=Homo sapiens GN=IGKV1-5 PE=4 SV=2 | 4 | 4,13 | 6 | 40,17 |
| 2136 | sp|Q71DI3|H32_HUMAN | HUMAN | Histone H3.2 OS=Homo sapiens GN=HIST2H3A PE=1 SV=3 | 4 | 4,1 | 7 | 27,21 |
| 2137 | sp|O43172|PRP4_HUMAN | HUMAN | U4/U6 small nuclear ribonucleoprotein Prp4 OS=Homo sapiens GN=PRPF4 PE=1 SV=2 | 4 | 4,05 | 2 | 5,747 |
| 2138 | sp|Q9Y3D0|MIP18_HUMAN | HUMAN | Mitotic spindle-associated MMXD complex subunit MIP18 OS=Homo sapiens GN=FAM96B PE=1 SV=1 | 4 | 4,01 | 2 | 26,38 |
| 2139 | sp|P14406|CX7A2_HUMAN | HUMAN | Cytochrome c oxidase subunit 7A2, mitochondrial OS=Homo sapiens GN=COX7A2 PE=1 SV=1 | 4 | 4,01 | 2 | 27,71 |
| 2140 | sp|O43504|LTOR5_HUMAN | HUMAN | Ragulator complex protein LAMTOR5 OS=Homo sapiens GN=LAMTOR5 PE=1 SV=1 | 4 | 4 | 2 | 43,96 |
| 2141 | sp|Q16563|SYPL1_HUMAN | HUMAN | Synaptophysin-like protein 1 OS=Homo sapiens GN=SYPL1 PE=1 SV=1 | 4 | 4 | 2 | 10,04 |
| 2142 | sp|P01779|HV318_HUMAN | HUMAN | Ig heavy chain V-III region TUR OS=Homo sapiens PE=1 SV=1 | 4 | 4 | 3 | 26,72 |
| 2143 | sp|Q9HD89|RETN_HUMAN | HUMAN | Resistin OS=Homo sapiens GN=RETN PE=1 SV=1 | 4 | 4 | 4 | 24,07 |
| 2144 | sp|Q9GZP8|IMUP_HUMAN | HUMAN | Immortalization up-regulated protein OS=Homo sapiens GN=IMUP PE=1 SV=1 | 4 | 4 | 2 | 33,02 |
| 2145 | sp|Q16790|CAH9_HUMAN | HUMAN | Carbonic anhydrase 9 OS=Homo sapiens GN=CA9 PE=1 SV=2 | 4 | 4 | 2 | 9,368 |
| 2146 | sp|Q14257|RCN2_HUMAN | HUMAN | Reticulocalbin-2 OS=Homo sapiens GN=RCN2 PE=1 SV=1 | 4 | 4 | 3 | 12,3 |
| 2147 | sp|P00748|FA12_HUMAN | HUMAN | Coagulation factor XII OS=Homo sapiens GN=F12 PE=1 SV=3 | 3,99 | 4,13 | 2 | 4,39 |
| 2148 | sp|O94888|UBXN7_HUMAN | HUMAN | UBX domain-containing protein 7 OS=Homo sapiens GN=UBXN7 PE=1 SV=2 | 3,98 | 4,09 | 3 | 7,157 |
| 2149 | sp|P19404|NDUV2_HUMAN | HUMAN | NADH dehydrogenase [ubiquinone] flavoprotein 2, mitochondrial OS=Homo sapiens GN=NDUFV2 PE=1 SV=2 | 3,96 | 4,04 | 2 | 9,237 |
| 2150 | sp|O43708|MAAI_HUMAN | HUMAN | Maleylacetoacetate isomerase OS=Homo sapiens GN=GSTZ1 PE=1 SV=3 | 3,96 | 4 | 2 | 15,74 |
| 2151 | sp|Q96A72|MGN2_HUMAN | HUMAN | Protein mago nashi homolog 2 OS=Homo sapiens GN=MAGOHB PE=1 SV=1 | 3,95 | 4,06 | 4 | 37,16 |
| 2152 | sp|Q9UHK6|AMACR_HUMAN | HUMAN | Alpha-methylacyl-CoA racemase OS=Homo sapiens GN=AMACR PE=1 SV=2 | 3,94 | 4,05 | 3 | 10,21 |
| 2153 | sp|P35542|SAA4_HUMAN | HUMAN | Serum amyloid A-4 protein OS=Homo sapiens GN=SAA4 PE=1 SV=2 | 3,92 | 3,99 | 3 | 21,54 |
| 2154 | sp|Q14956|GPNMB_HUMAN | HUMAN | Transmembrane glycoprotein NMB OS=Homo sapiens GN=GPNMB PE=1 SV=2 | 3,92 | 3,97 | 2 | 4,72 |
| 2155 | sp|Q9Y6H1|CHCH2_HUMAN | HUMAN | Coiled-coil-helix-coiled-coil-helix domain-containing protein 2 OS=Homo sapiens GN=CHCHD2 PE=1 SV=1 | 3,92 | 3,96 | 4 | 24,5 |
| 2156 | sp|Q9H9S4|CB39L_HUMAN | HUMAN | Calcium-binding protein 39-like OS=Homo sapiens GN=CAB39L PE=1 SV=3 | 3,91 | 10,91 | 8 | 21,96 |
| 2157 | sp|Q6PI48|SYDM_HUMAN | HUMAN | Aspartate--tRNA ligase, mitochondrial OS=Homo sapiens GN=DARS2 PE=1 SV=1 | 3,9 | 4,14 | 2 | 4,496 |
| 2158 | sp|Q8WTW3|COG1_HUMAN | HUMAN | Conserved oligomeric Golgi complex subunit 1 OS=Homo sapiens GN=COG1 PE=1 SV=1 | 3,9 | 4 | 3 | 4,184 |
| 2159 | sp|O95999|BCL10_HUMAN | HUMAN | B-cell lymphoma/leukemia 10 OS=Homo sapiens GN=BCL10 PE=1 SV=1 | 3,9 | 3,99 | 3 | 12,45 |
| 2160 | sp|P53582|MAP11_HUMAN | HUMAN | Methionine aminopeptidase 1 OS=Homo sapiens GN=METAP1 PE=1 SV=2 | 3,9 | 3,96 | 2 | 8,031 |
| 2161 | sp|Q96RF0|SNX18_HUMAN | HUMAN | Sorting nexin-18 OS=Homo sapiens GN=SNX18 PE=1 SV=2 | 3,9 | 3,93 | 2 | 4,618 |
| 2162 | sp|P43246|MSH2_HUMAN | HUMAN | DNA mismatch repair protein Msh2 OS=Homo sapiens GN=MSH2 PE=1 SV=1 | 3,89 | 4,07 | 4 | 5,782 |
| 2163 | sp|Q96H20|SNF8_HUMAN | HUMAN | Vacuolar-sorting protein SNF8 OS=Homo sapiens GN=SNF8 PE=1 SV=1 | 3,89 | 3,95 | 3 | 14,34 |
| 2164 | sp|Q9Y6K5|OAS3_HUMAN | HUMAN | 2'-5'-oligoadenylate synthase 3 OS=Homo sapiens GN=OAS3 PE=1 SV=3 | 3,87 | 4,02 | 2 | 1,84 |
| 2165 | sp|O14562|UBFD1_HUMAN | HUMAN | Ubiquitin domain-containing protein UBFD1 OS=Homo sapiens GN=UBFD1 PE=1 SV=2 | 3,87 | 4,01 | 3 | 11 |
| 2166 | sp|P22676|CALB2_HUMAN | HUMAN | Calretinin OS=Homo sapiens GN=CALB2 PE=2 SV=2 | 3,87 | 3,99 | 4 | 16,97 |
| 2167 | sp|P02654|APOC1_HUMAN | HUMAN | Apolipoprotein C-I OS=Homo sapiens GN=APOC1 PE=1 SV=1 | 3,87 | 3,97 | 5 | 37,35 |
| 2168 | sp|Q6IN85|P4R3A_HUMAN | HUMAN | Serine/threonine-protein phosphatase 4 regulatory subunit 3A OS=Homo sapiens GN=PPP4R3A PE=1 SV=1 | 3,87 | 3,91 | 2 | 3,121 |
| 2169 | sp|Q16740|CLPP_HUMAN | HUMAN | ATP-dependent Clp protease proteolytic subunit, mitochondrial OS=Homo sapiens GN=CLPP PE=1 SV=1 | 3,86 | 3,89 | 2 | 11,91 |
| 2170 | sp|Q7Z5R6|AB1IP_HUMAN | HUMAN | Amyloid beta A4 precursor protein-binding family B member 1-interacting protein OS=Homo sapiens GN=APBB1IP PE=1 SV=1 | 3,85 | 4,67 | 3 | 5,255 |
| 2171 | sp|P42285|SK2L2_HUMAN | HUMAN | Superkiller viralicidic activity 2-like 2 OS=Homo sapiens GN=SKIV2L2 PE=1 SV=3 | 3,85 | 4,07 | 4 | 6,046 |
| 2172 | sp|Q9H3P2|NELFA_HUMAN | HUMAN | Negative elongation factor A OS=Homo sapiens GN=NELFA PE=1 SV=3 | 3,85 | 4 | 3 | 9,47 |
| 2173 | sp|P54709|AT1B3_HUMAN | HUMAN | Sodium/potassium-transporting ATPase subunit beta-3 OS=Homo sapiens GN=ATP1B3 PE=1 SV=1 | 3,83 | 4,15 | 3 | 14,34 |
| 2174 | sp|Q9H0C8|ILKAP_HUMAN | HUMAN | Integrin-linked kinase-associated serine/threonine phosphatase 2C OS=Homo sapiens GN=ILKAP PE=1 SV=1 | 3,83 | 4,02 | 3 | 7,398 |
| 2175 | sp|O60925|PFD1_HUMAN | HUMAN | Prefoldin subunit 1 OS=Homo sapiens GN=PFDN1 PE=1 SV=2 | 3,83 | 3,95 | 2 | 17,21 |
| 2176 | sp|Q92541|RTF1_HUMAN | HUMAN | RNA polymerase-associated protein RTF1 homolog OS=Homo sapiens GN=RTF1 PE=1 SV=4 | 3,83 | 3,92 | 2 | 3,099 |
| 2177 | sp|P00995|ISK1_HUMAN | HUMAN | Serine protease inhibitor Kazal-type 1 OS=Homo sapiens GN=SPINK1 PE=1 SV=2 | 3,83 | 3,91 | 3 | 53,16 |
| 2178 | sp|Q86VB7|C163A_HUMAN | HUMAN | Scavenger receptor cysteine-rich type 1 protein M130 OS=Homo sapiens GN=CD163 PE=1 SV=2 | 3,82 | 3,99 | 3 | 3,374 |
| 2179 | sp|Q15428|SF3A2_HUMAN | HUMAN | Splicing factor 3A subunit 2 OS=Homo sapiens GN=SF3A2 PE=1 SV=2 | 3,82 | 3,93 | 3 | 8,836 |
| 2180 | sp|Q96AY3|FKB10_HUMAN | HUMAN | Peptidyl-prolyl cis-trans isomerase FKBP10 OS=Homo sapiens GN=FKBP10 PE=1 SV=1 | 3,82 | 3,91 | 2 | 3,608 |
| 2181 | sp|P10643|CO7_HUMAN | HUMAN | Complement component C7 OS=Homo sapiens GN=C7 PE=1 SV=2 | 3,82 | 3,86 | 2 | 4,152 |
| 2182 | sp|Q14746|COG2_HUMAN | HUMAN | Conserved oligomeric Golgi complex subunit 2 OS=Homo sapiens GN=COG2 PE=1 SV=1 | 3,81 | 4,03 | 4 | 6,911 |
| 2183 | sp|Q14669|TRIPC_HUMAN | HUMAN | E3 ubiquitin-protein ligase TRIP12 OS=Homo sapiens GN=TRIP12 PE=1 SV=1 | 3,81 | 4 | 2 | 1,205 |
| 2184 | sp|O43617|TPPC3_HUMAN | HUMAN | Trafficking protein particle complex subunit 3 OS=Homo sapiens GN=TRAPPC3 PE=1 SV=1 | 3,81 | 3,92 | 3 | 16,11 |
| 2185 | sp|P56211|ARP19_HUMAN | HUMAN | cAMP-regulated phosphoprotein 19 OS=Homo sapiens GN=ARPP19 PE=1 SV=2 | 3,8 | 5,74 | 5 | 50,89 |
| 2186 | sp|O75312|ZPR1_HUMAN | HUMAN | Zinc finger protein ZPR1 OS=Homo sapiens GN=ZPR1 PE=1 SV=1 | 3,8 | 3,95 | 3 | 8,497 |
| 2187 | sp|O14493|CLD4_HUMAN | HUMAN | Claudin-4 OS=Homo sapiens GN=CLDN4 PE=1 SV=1 | 3,8 | 3,85 | 2 | 12,92 |
| 2188 | sp|Q6P5R6|RL22L_HUMAN | HUMAN | 60S ribosomal protein L22-like 1 OS=Homo sapiens GN=RPL22L1 PE=1 SV=2 | 3,8 | 3,82 | 2 | 29,51 |
| 2189 | sp|Q14155|ARHG7_HUMAN | HUMAN | Rho guanine nucleotide exchange factor 7 OS=Homo sapiens GN=ARHGEF7 PE=1 SV=2 | 3,79 | 3,97 | 4 | 5,355 |
| 2190 | sp|P01714|LV301_HUMAN | HUMAN | Ig lambda chain V-III region SH OS=Homo sapiens PE=1 SV=1 | 3,79 | 3,84 | 4 | 32,41 |
| 2191 | sp|O43395|PRPF3_HUMAN | HUMAN | U4/U6 small nuclear ribonucleoprotein Prp3 OS=Homo sapiens GN=PRPF3 PE=1 SV=2 | 3,78 | 3,94 | 3 | 4,539 |
| 2192 | sp|Q99848|EBP2_HUMAN | HUMAN | Probable rRNA-processing protein EBP2 OS=Homo sapiens GN=EBNA1BP2 PE=1 SV=2 | 3,78 | 3,9 | 4 | 12,09 |
| 2193 | sp|Q9BXK5|B2L13_HUMAN | HUMAN | Bcl-2-like protein 13 OS=Homo sapiens GN=BCL2L13 PE=1 SV=1 | 3,78 | 3,84 | 2 | 7,216 |
| 2194 | sp|Q9NPD3|EXOS4_HUMAN | HUMAN | Exosome complex component RRP41 OS=Homo sapiens GN=EXOSC4 PE=1 SV=3 | 3,78 | 3,84 | 2 | 8,98 |
| 2195 | sp|O75489|NDUS3_HUMAN | HUMAN | NADH dehydrogenase [ubiquinone] iron-sulfur protein 3, mitochondrial OS=Homo sapiens GN=NDUFS3 PE=1 SV=1 | 3,77 | 4,05 | 4 | 17,05 |
| 2196 | sp|Q69YN2|C19L1_HUMAN | HUMAN | CWF19-like protein 1 OS=Homo sapiens GN=CWF19L1 PE=1 SV=2 | 3,77 | 3,85 | 3 | 4,461 |
| 2197 | sp|P37198|NUP62_HUMAN | HUMAN | Nuclear pore glycoprotein p62 OS=Homo sapiens GN=NUP62 PE=1 SV=3 | 3,77 | 3,82 | 2 | 4,598 |
| 2198 | sp|Q96B36|AKTS1_HUMAN | HUMAN | Proline-rich AKT1 substrate 1 OS=Homo sapiens GN=AKT1S1 PE=1 SV=1 | 3,76 | 3,82 | 2 | 11,72 |
| 2199 | sp|Q14554|PDIA5_HUMAN | HUMAN | Protein disulfide-isomerase A5 OS=Homo sapiens GN=PDIA5 PE=1 SV=1 | 3,75 | 3,87 | 3 | 8,285 |
| 2200 | sp|Q9H0A0|NAT10_HUMAN | HUMAN | RNA cytidine acetyltransferase OS=Homo sapiens GN=NAT10 PE=1 SV=2 | 3,74 | 3,95 | 2 | 2,537 |
| 2201 | sp|Q8N4P3|MESH1_HUMAN | HUMAN | Guanosine-3',5'-bis(diphosphate) 3'-pyrophosphohydrolase MESH1 OS=Homo sapiens GN=HDDC3 PE=1 SV=3 | 3,74 | 3,79 | 2 | 16,76 |
| 2202 | sp|Q8NCA5|FA98A_HUMAN | HUMAN | Protein FAM98A OS=Homo sapiens GN=FAM98A PE=1 SV=1 | 3,72 | 5,91 | 5 | 13,49 |
| 2203 | sp|P19878|NCF2_HUMAN | HUMAN | Neutrophil cytosol factor 2 OS=Homo sapiens GN=NCF2 PE=1 SV=2 | 3,72 | 3,92 | 2 | 4,563 |
| 2204 | sp|Q9NXR7|BRE_HUMAN | HUMAN | BRCA1-A complex subunit BRE OS=Homo sapiens GN=BRE PE=1 SV=2 | 3,72 | 3,88 | 3 | 9,922 |
| 2205 | sp|P63267|ACTH_HUMAN | HUMAN | Actin, gamma-enteric smooth muscle OS=Homo sapiens GN=ACTG2 PE=1 SV=1 | 3,71 | 66,77 | 227 | 80,85 |
| 2206 | sp|Q86TI2|DPP9_HUMAN | HUMAN | Dipeptidyl peptidase 9 OS=Homo sapiens GN=DPP9 PE=1 SV=3 | 3,7 | 3,83 | 3 | 3,476 |
| 2207 | sp|O94925|GLSK_HUMAN | HUMAN | Glutaminase kidney isoform, mitochondrial OS=Homo sapiens GN=GLS PE=1 SV=1 | 3,7 | 3,75 | 3 | 7,324 |
| 2208 | sp|P13987|CD59_HUMAN | HUMAN | CD59 glycoprotein OS=Homo sapiens GN=CD59 PE=1 SV=1 | 3,7 | 3,74 | 3 | 26,56 |
| 2209 | sp|P07947|YES_HUMAN | HUMAN | Tyrosine-protein kinase Yes OS=Homo sapiens GN=YES1 PE=1 SV=3 | 3,69 | 5 | 3 | 5,341 |
| 2210 | sp|Q9HDC9|APMAP_HUMAN | HUMAN | Adipocyte plasma membrane-associated protein OS=Homo sapiens GN=APMAP PE=1 SV=2 | 3,69 | 3,82 | 3 | 11,54 |
| 2211 | sp|Q8N5K1|CISD2_HUMAN | HUMAN | CDGSH iron-sulfur domain-containing protein 2 OS=Homo sapiens GN=CISD2 PE=1 SV=1 | 3,69 | 3,77 | 2 | 19,26 |
| 2212 | sp|Q8N129|CNPY4_HUMAN | HUMAN | Protein canopy homolog 4 OS=Homo sapiens GN=CNPY4 PE=2 SV=1 | 3,69 | 3,76 | 2 | 9,274 |
| 2213 | sp|P08519|APOA_HUMAN | HUMAN | Apolipoprotein(a) OS=Homo sapiens GN=LPA PE=1 SV=1 | 3,68 | 3,98 | 3 | 15,28 |
| 2214 | sp|Q96MW1|CCD43_HUMAN | HUMAN | Coiled-coil domain-containing protein 43 OS=Homo sapiens GN=CCDC43 PE=1 SV=2 | 3,66 | 3,84 | 3 | 22,77 |
| 2215 | sp|Q96GA7|SDSL_HUMAN | HUMAN | Serine dehydratase-like OS=Homo sapiens GN=SDSL PE=1 SV=1 | 3,66 | 3,75 | 2 | 13,07 |
| 2216 | sp|Q0VDF9|HSP7E_HUMAN | HUMAN | Heat shock 70 kDa protein 14 OS=Homo sapiens GN=HSPA14 PE=1 SV=1 | 3,65 | 3,86 | 3 | 9,234 |
| 2217 | sp|Q96D71|REPS1_HUMAN | HUMAN | RalBP1-associated Eps domain-containing protein 1 OS=Homo sapiens GN=REPS1 PE=1 SV=3 | 3,65 | 3,76 | 4 | 6,156 |
| 2218 | sp|Q14739|LBR_HUMAN | HUMAN | Lamin-B receptor OS=Homo sapiens GN=LBR PE=1 SV=2 | 3,65 | 3,74 | 2 | 3,415 |
| 2219 | sp|Q8WVJ2|NUDC2_HUMAN | HUMAN | NudC domain-containing protein 2 OS=Homo sapiens GN=NUDCD2 PE=1 SV=1 | 3,64 | 3,74 | 4 | 29,94 |
| 2220 | sp|P52434|RPAB3_HUMAN | HUMAN | DNA-directed RNA polymerases I, II, and III subunit RPABC3 OS=Homo sapiens GN=POLR2H PE=1 SV=4 | 3,64 | 3,68 | 2 | 19,33 |
| 2221 | sp|O60216|RAD21_HUMAN | HUMAN | Double-strand-break repair protein rad21 homolog OS=Homo sapiens GN=RAD21 PE=1 SV=2 | 3,63 | 3,82 | 3 | 5,705 |
| 2222 | sp|P08240|SRPRA_HUMAN | HUMAN | Signal recognition particle receptor subunit alpha OS=Homo sapiens GN=SRPRA PE=1 SV=2 | 3,62 | 3,78 | 3 | 5,643 |
| 2223 | sp|P27701|CD82_HUMAN | HUMAN | CD82 antigen OS=Homo sapiens GN=CD82 PE=1 SV=1 | 3,61 | 3,71 | 2 | 9,363 |
| 2224 | sp|Q9BWS9|CHID1_HUMAN | HUMAN | Chitinase domain-containing protein 1 OS=Homo sapiens GN=CHID1 PE=1 SV=1 | 3,61 | 3,69 | 2 | 5,344 |
| 2225 | sp|P60468|SC61B_HUMAN | HUMAN | Protein transport protein Sec61 subunit beta OS=Homo sapiens GN=SEC61B PE=1 SV=2 | 3,6 | 3,73 | 2 | 26,04 |
| 2226 | sp|Q15836|VAMP3_HUMAN | HUMAN | Vesicle-associated membrane protein 3 OS=Homo sapiens GN=VAMP3 PE=1 SV=3 | 3,6 | 3,69 | 3 | 45 |
| 2227 | sp|P23434|GCSH_HUMAN | HUMAN | Glycine cleavage system H protein, mitochondrial OS=Homo sapiens GN=GCSH PE=1 SV=2 | 3,6 | 3,66 | 2 | 32,95 |
| 2228 | sp|P36873|PP1G_HUMAN | HUMAN | Serine/threonine-protein phosphatase PP1-gamma catalytic subunit OS=Homo sapiens GN=PPP1CC PE=1 SV=1 | 3,59 | 30,32 | 19 | 55,42 |
| 2229 | sp|O94875|SRBS2_HUMAN | HUMAN | Sorbin and SH3 domain-containing protein 2 OS=Homo sapiens GN=SORBS2 PE=1 SV=3 | 3,58 | 3,79 | 2 | 2,182 |
| 2230 | sp|Q9BTW9|TBCD_HUMAN | HUMAN | Tubulin-specific chaperone D OS=Homo sapiens GN=TBCD PE=1 SV=2 | 3,58 | 3,76 | 2 | 3,188 |
| 2231 | sp|Q96RQ9|OXLA_HUMAN | HUMAN | L-amino-acid oxidase OS=Homo sapiens GN=IL4I1 PE=1 SV=1 | 3,58 | 3,71 | 2 | 4,233 |
| 2232 | sp|Q3KQU3|MA7D1_HUMAN | HUMAN | MAP7 domain-containing protein 1 OS=Homo sapiens GN=MAP7D1 PE=1 SV=1 | 3,58 | 3,69 | 4 | 6,064 |
| 2233 | sp|P62304|RUXE_HUMAN | HUMAN | Small nuclear ribonucleoprotein E OS=Homo sapiens GN=SNRPE PE=1 SV=1 | 3,58 | 3,61 | 3 | 25 |
| 2234 | sp|O75165|DJC13_HUMAN | HUMAN | DnaJ homolog subfamily C member 13 OS=Homo sapiens GN=DNAJC13 PE=1 SV=5 | 3,57 | 3,65 | 2 | 0,8025 |
| 2235 | sp|Q9NSC7|SIA7A_HUMAN | HUMAN | Alpha-N-acetylgalactosaminide alpha-2,6-sialyltransferase 1 OS=Homo sapiens GN=ST6GALNAC1 PE=2 SV=1 | 3,56 | 3,72 | 3 | 6,333 |
| 2236 | sp|Q8WVV9|HNRLL_HUMAN | HUMAN | Heterogeneous nuclear ribonucleoprotein L-like OS=Homo sapiens GN=HNRNPLL PE=1 SV=1 | 3,55 | 4,62 | 4 | 9,779 |
| 2237 | sp|Q8TE67|ES8L3_HUMAN | HUMAN | Epidermal growth factor receptor kinase substrate 8-like protein 3 OS=Homo sapiens GN=EPS8L3 PE=1 SV=2 | 3,55 | 3,74 | 3 | 6,239 |
| 2238 | sp|Q6NZY7|BORG3_HUMAN | HUMAN | Cdc42 effector protein 5 OS=Homo sapiens GN=CDC42EP5 PE=2 SV=1 | 3,55 | 3,66 | 2 | 17,57 |
| 2239 | sp|P49593|PPM1F_HUMAN | HUMAN | Protein phosphatase 1F OS=Homo sapiens GN=PPM1F PE=1 SV=3 | 3,55 | 3,6 | 2 | 6,388 |
| 2240 | sp|Q9ULC5|ACSL5_HUMAN | HUMAN | Long-chain-fatty-acid--CoA ligase 5 OS=Homo sapiens GN=ACSL5 PE=1 SV=1 | 3,54 | 3,59 | 2 | 3,514 |
| 2241 | sp|P62308|RUXG_HUMAN | HUMAN | Small nuclear ribonucleoprotein G OS=Homo sapiens GN=SNRPG PE=1 SV=1 | 3,54 | 3,59 | 2 | 25 |
| 2242 | sp|P48960|CD97_HUMAN | HUMAN | CD97 antigen OS=Homo sapiens GN=CD97 PE=1 SV=4 | 3,53 | 3,61 | 2 | 4,311 |
| 2243 | sp|P02462|CO4A1_HUMAN | HUMAN | Collagen alpha-1(IV) chain OS=Homo sapiens GN=COL4A1 PE=1 SV=3 | 3,53 | 3,6 | 2 | 1,738 |
| 2244 | sp|P07919|QCR6_HUMAN | HUMAN | Cytochrome b-c1 complex subunit 6, mitochondrial OS=Homo sapiens GN=UQCRH PE=1 SV=2 | 3,53 | 3,6 | 3 | 35,16 |
| 2245 | sp|O94811|TPPP_HUMAN | HUMAN | Tubulin polymerization-promoting protein OS=Homo sapiens GN=TPPP PE=1 SV=1 | 3,52 | 3,64 | 3 | 22,83 |
| 2246 | sp|Q6L8Q7|PDE12_HUMAN | HUMAN | 2',5'-phosphodiesterase 12 OS=Homo sapiens GN=PDE12 PE=1 SV=2 | 3,51 | 3,69 | 3 | 5,911 |
| 2247 | sp|Q96C01|F136A_HUMAN | HUMAN | Protein FAM136A OS=Homo sapiens GN=FAM136A PE=1 SV=1 | 3,51 | 3,6 | 3 | 26,09 |
| 2248 | sp|O43615|TIM44_HUMAN | HUMAN | Mitochondrial import inner membrane translocase subunit TIM44 OS=Homo sapiens GN=TIMM44 PE=1 SV=2 | 3,51 | 3,58 | 2 | 5,088 |
| 2249 | sp|P00167|CYB5_HUMAN | HUMAN | Cytochrome b5 OS=Homo sapiens GN=CYB5A PE=1 SV=2 | 3,5 | 3,58 | 3 | 35,82 |
| 2250 | sp|P55058|PLTP_HUMAN | HUMAN | Phospholipid transfer protein OS=Homo sapiens GN=PLTP PE=1 SV=1 | 3,49 | 3,68 | 4 | 8,519 |
| 2251 | sp|Q7L266|ASGL1_HUMAN | HUMAN | Isoaspartyl peptidase/L-asparaginase OS=Homo sapiens GN=ASRGL1 PE=1 SV=2 | 3,49 | 3,57 | 2 | 10,71 |
| 2252 | sp|Q16719|KYNU_HUMAN | HUMAN | Kynureninase OS=Homo sapiens GN=KYNU PE=1 SV=1 | 3,49 | 3,55 | 3 | 8,817 |
| 2253 | sp|Q9Y5B9|SP16H_HUMAN | HUMAN | FACT complex subunit SPT16 OS=Homo sapiens GN=SUPT16H PE=1 SV=1 | 3,48 | 3,73 | 3 | 3,438 |
| 2254 | sp|Q9NX58|LYAR_HUMAN | HUMAN | Cell growth-regulating nucleolar protein OS=Homo sapiens GN=LYAR PE=1 SV=2 | 3,48 | 3,56 | 2 | 6,596 |
| 2255 | sp|Q9H6T3|RPAP3_HUMAN | HUMAN | RNA polymerase II-associated protein 3 OS=Homo sapiens GN=RPAP3 PE=1 SV=2 | 3,47 | 3,57 | 2 | 4,361 |
| 2256 | sp|Q9BTD8|RBM42_HUMAN | HUMAN | RNA-binding protein 42 OS=Homo sapiens GN=RBM42 PE=1 SV=1 | 3,46 | 3,57 | 3 | 13,75 |
| 2257 | sp|P49585|PCY1A_HUMAN | HUMAN | Choline-phosphate cytidylyltransferase A OS=Homo sapiens GN=PCYT1A PE=1 SV=2 | 3,46 | 3,55 | 2 | 8,992 |
| 2258 | sp|Q13724|MOGS_HUMAN | HUMAN | Mannosyl-oligosaccharide glucosidase OS=Homo sapiens GN=MOGS PE=1 SV=5 | 3,46 | 3,54 | 3 | 5,735 |
| 2259 | sp|Q9HAV7|GRPE1_HUMAN | HUMAN | GrpE protein homolog 1, mitochondrial OS=Homo sapiens GN=GRPEL1 PE=1 SV=2 | 3,46 | 3,53 | 2 | 9,217 |
| 2260 | sp|P19075|TSN8_HUMAN | HUMAN | Tetraspanin-8 OS=Homo sapiens GN=TSPAN8 PE=1 SV=1 | 3,46 | 3,53 | 3 | 11,81 |
| 2261 | sp|P51692|STA5B_HUMAN | HUMAN | Signal transducer and activator of transcription 5B OS=Homo sapiens GN=STAT5B PE=1 SV=2 | 3,45 | 4,22 | 4 | 4,066 |
| 2262 | sp|P14543|NID1_HUMAN | HUMAN | Nidogen-1 OS=Homo sapiens GN=NID1 PE=1 SV=3 | 3,45 | 4 | 3 | 2,566 |
| 2263 | sp|Q6IA69|NADE_HUMAN | HUMAN | Glutamine-dependent NAD(+) synthetase OS=Homo sapiens GN=NADSYN1 PE=1 SV=3 | 3,45 | 3,61 | 3 | 5,241 |
| 2264 | sp|O75964|ATP5L_HUMAN | HUMAN | ATP synthase subunit g, mitochondrial OS=Homo sapiens GN=ATP5L PE=1 SV=3 | 3,45 | 3,54 | 3 | 37,86 |
| 2265 | sp|Q6Y7W6|PERQ2_HUMAN | HUMAN | PERQ amino acid-rich with GYF domain-containing protein 2 OS=Homo sapiens GN=GIGYF2 PE=1 SV=1 | 3,44 | 4,78 | 3 | 2,694 |
| 2266 | sp|P07099|HYEP_HUMAN | HUMAN | Epoxide hydrolase 1 OS=Homo sapiens GN=EPHX1 PE=1 SV=1 | 3,44 | 3,73 | 3 | 7,912 |
| 2267 | sp|Q9UK41|VPS28_HUMAN | HUMAN | Vacuolar protein sorting-associated protein 28 homolog OS=Homo sapiens GN=VPS28 PE=1 SV=1 | 3,43 | 3,8 | 2 | 22,62 |
| 2268 | sp|Q9NQ48|LZTL1_HUMAN | HUMAN | Leucine zipper transcription factor-like protein 1 OS=Homo sapiens GN=LZTFL1 PE=1 SV=1 | 3,42 | 3,65 | 3 | 8,027 |
| 2269 | sp|Q7Z422|SZRD1_HUMAN | HUMAN | SUZ domain-containing protein 1 OS=Homo sapiens GN=SZRD1 PE=1 SV=1 | 3,42 | 3,51 | 2 | 21,71 |
| 2270 | sp|Q5TFE4|NT5D1_HUMAN | HUMAN | 5'-nucleotidase domain-containing protein 1 OS=Homo sapiens GN=NT5DC1 PE=1 SV=1 | 3,4 | 3,45 | 2 | 5,495 |
| 2271 | sp|P31431|SDC4_HUMAN | HUMAN | Syndecan-4 OS=Homo sapiens GN=SDC4 PE=1 SV=2 | 3,4 | 3,45 | 2 | 13,13 |
| 2272 | sp|Q96KP1|EXOC2_HUMAN | HUMAN | Exocyst complex component 2 OS=Homo sapiens GN=EXOC2 PE=1 SV=1 | 3,39 | 3,48 | 2 | 2,165 |
| 2273 | sp|Q8IVD9|NUDC3_HUMAN | HUMAN | NudC domain-containing protein 3 OS=Homo sapiens GN=NUDCD3 PE=1 SV=3 | 3,39 | 3,48 | 2 | 11,08 |
| 2274 | sp|Q9H4A3|WNK1_HUMAN | HUMAN | Serine/threonine-protein kinase WNK1 OS=Homo sapiens GN=WNK1 PE=1 SV=2 | 3,38 | 3,53 | 2 | 0,9236 |
| 2275 | sp|O75663|TIPRL_HUMAN | HUMAN | TIP41-like protein OS=Homo sapiens GN=TIPRL PE=1 SV=2 | 3,38 | 3,48 | 4 | 17,65 |
| 2276 | sp|Q12972|PP1R8_HUMAN | HUMAN | Nuclear inhibitor of protein phosphatase 1 OS=Homo sapiens GN=PPP1R8 PE=1 SV=2 | 3,38 | 3,46 | 3 | 11,4 |
| 2277 | sp|Q9H2D6|TARA_HUMAN | HUMAN | TRIO and F-actin-binding protein OS=Homo sapiens GN=TRIOBP PE=1 SV=3 | 3,37 | 3,51 | 2 | 0,8457 |
| 2278 | sp|P35249|RFC4_HUMAN | HUMAN | Replication factor C subunit 4 OS=Homo sapiens GN=RFC4 PE=1 SV=2 | 3,37 | 3,51 | 2 | 5,234 |
| 2279 | sp|Q9P0R6|GSKIP_HUMAN | HUMAN | GSK3-beta interaction protein OS=Homo sapiens GN=GSKIP PE=1 SV=2 | 3,37 | 3,43 | 2 | 31,65 |
| 2280 | sp|Q9H5X1|FA96A_HUMAN | HUMAN | MIP18 family protein FAM96A OS=Homo sapiens GN=FAM96A PE=1 SV=1 | 3,36 | 3,4 | 2 | 12,5 |
| 2281 | sp|Q96S97|MYADM_HUMAN | HUMAN | Myeloid-associated differentiation marker OS=Homo sapiens GN=MYADM PE=1 SV=2 | 3,35 | 3,62 | 2 | 10,25 |
| 2282 | sp|O60613|SEP15_HUMAN | HUMAN | 15 kDa selenoprotein OS=Homo sapiens GN=SEP15 PE=1 SV=3 | 3,35 | 3,46 | 3 | 17,9 |
| 2283 | sp|Q8N6T3|ARFG1_HUMAN | HUMAN | ADP-ribosylation factor GTPase-activating protein 1 OS=Homo sapiens GN=ARFGAP1 PE=1 SV=2 | 3,35 | 3,4 | 2 | 7,143 |
| 2284 | sp|O75475|PSIP1_HUMAN | HUMAN | PC4 and SFRS1-interacting protein OS=Homo sapiens GN=PSIP1 PE=1 SV=1 | 3,34 | 4,25 | 5 | 10,57 |
| 2285 | sp|Q6NYC8|PPR18_HUMAN | HUMAN | Phostensin OS=Homo sapiens GN=PPP1R18 PE=1 SV=1 | 3,33 | 3,55 | 2 | 4,568 |
| 2286 | sp|Q96JJ3|ELMO2_HUMAN | HUMAN | Engulfment and cell motility protein 2 OS=Homo sapiens GN=ELMO2 PE=1 SV=2 | 3,33 | 3,54 | 2 | 3,889 |
| 2287 | sp|O15042|SR140_HUMAN | HUMAN | U2 snRNP-associated SURP motif-containing protein OS=Homo sapiens GN=U2SURP PE=1 SV=2 | 3,33 | 3,52 | 2 | 2,43 |
| 2288 | sp|Q9NS86|LANC2_HUMAN | HUMAN | LanC-like protein 2 OS=Homo sapiens GN=LANCL2 PE=1 SV=1 | 3,33 | 3,5 | 2 | 6,222 |
| 2289 | sp|Q05397|FAK1_HUMAN | HUMAN | Focal adhesion kinase 1 OS=Homo sapiens GN=PTK2 PE=1 SV=2 | 3,33 | 3,42 | 2 | 2,757 |
| 2290 | sp|Q96DB5|RMD1_HUMAN | HUMAN | Regulator of microtubule dynamics protein 1 OS=Homo sapiens GN=RMDN1 PE=1 SV=1 | 3,32 | 3,43 | 3 | 11,78 |
| 2291 | sp|Q5EBL8|PDZ11_HUMAN | HUMAN | PDZ domain-containing protein 11 OS=Homo sapiens GN=PDZD11 PE=1 SV=2 | 3,32 | 3,38 | 2 | 25,71 |
| 2292 | sp|Q93034|CUL5_HUMAN | HUMAN | Cullin-5 OS=Homo sapiens GN=CUL5 PE=1 SV=4 | 3,3 | 4,22 | 3 | 3,077 |
| 2293 | sp|P47985|UCRI_HUMAN | HUMAN | Cytochrome b-c1 complex subunit Rieske, mitochondrial OS=Homo sapiens GN=UQCRFS1 PE=1 SV=2 | 3,3 | 3,47 | 3 | 15,33 |
| 2294 | sp|Q9NYJ1|COA4_HUMAN | HUMAN | Cytochrome c oxidase assembly factor 4 homolog, mitochondrial OS=Homo sapiens GN=COA4 PE=1 SV=2 | 3,3 | 3,43 | 3 | 34,48 |
| 2295 | sp|P10109|ADX_HUMAN | HUMAN | Adrenodoxin, mitochondrial OS=Homo sapiens GN=FDX1 PE=1 SV=1 | 3,3 | 3,38 | 2 | 9,783 |
| 2296 | sp|O75884|RBBP9_HUMAN | HUMAN | Putative hydrolase RBBP9 OS=Homo sapiens GN=RBBP9 PE=1 SV=2 | 3,3 | 3,37 | 2 | 19,89 |
| 2297 | sp|P62861|RS30_HUMAN | HUMAN | 40S ribosomal protein S30 OS=Homo sapiens GN=FAU PE=1 SV=1 | 3,3 | 3,37 | 6 | 44,07 |
| 2298 | sp|P60033|CD81_HUMAN | HUMAN | CD81 antigen OS=Homo sapiens GN=CD81 PE=1 SV=1 | 3,29 | 3,36 | 2 | 18,22 |
| 2299 | sp|Q9UDW1|QCR9_HUMAN | HUMAN | Cytochrome b-c1 complex subunit 9 OS=Homo sapiens GN=UQCR10 PE=1 SV=3 | 3,28 | 3,4 | 2 | 38,1 |
| 2300 | sp|Q96S44|PRPK_HUMAN | HUMAN | TP53-regulating kinase OS=Homo sapiens GN=TP53RK PE=1 SV=2 | 3,28 | 3,37 | 2 | 10,28 |
| 2301 | sp|P62834|RAP1A_HUMAN | HUMAN | Ras-related protein Rap-1A OS=Homo sapiens GN=RAP1A PE=1 SV=1 | 3,27 | 14,98 | 9 | 57,61 |
| 2302 | sp|Q9Y5K8|VATD_HUMAN | HUMAN | V-type proton ATPase subunit D OS=Homo sapiens GN=ATP6V1D PE=1 SV=1 | 3,27 | 3,45 | 2 | 9,312 |
| 2303 | sp|P07477|TRY1_HUMAN | HUMAN | Trypsin-1 OS=Homo sapiens GN=PRSS1 PE=1 SV=1 | 3,26 | 3,31 | 15 | 12,15 |
| 2304 | sp|O75531|BAF_HUMAN | HUMAN | Barrier-to-autointegration factor OS=Homo sapiens GN=BANF1 PE=1 SV=1 | 3,25 | 3,37 | 4 | 48,31 |
| 2305 | sp|P11215|ITAM_HUMAN | HUMAN | Integrin alpha-M OS=Homo sapiens GN=ITGAM PE=1 SV=2 | 3,24 | 3,42 | 2 | 1,997 |
| 2306 | sp|Q9BXS5|AP1M1_HUMAN | HUMAN | AP-1 complex subunit mu-1 OS=Homo sapiens GN=AP1M1 PE=1 SV=3 | 3,23 | 7,92 | 5 | 13,95 |
| 2307 | sp|Q96IY4|CBPB2_HUMAN | HUMAN | Carboxypeptidase B2 OS=Homo sapiens GN=CPB2 PE=1 SV=2 | 3,21 | 3,32 | 2 | 4,492 |
| 2308 | sp|O60575|ISK4_HUMAN | HUMAN | Serine protease inhibitor Kazal-type 4 OS=Homo sapiens GN=SPINK4 PE=3 SV=1 | 3,19 | 3,25 | 3 | 10,47 |
| 2309 | sp|Q9UEW8|STK39_HUMAN | HUMAN | STE20/SPS1-related proline-alanine-rich protein kinase OS=Homo sapiens GN=STK39 PE=1 SV=3 | 3,17 | 4,58 | 4 | 8,624 |
| 2310 | sp|Q9UI10|EI2BD_HUMAN | HUMAN | Translation initiation factor eIF-2B subunit delta OS=Homo sapiens GN=EIF2B4 PE=1 SV=2 | 3,17 | 3,41 | 2 | 7,648 |
| 2311 | sp|O14907|TX1B3_HUMAN | HUMAN | Tax1-binding protein 3 OS=Homo sapiens GN=TAX1BP3 PE=1 SV=2 | 3,17 | 3,26 | 3 | 42,74 |
| 2312 | sp|Q9P289|STK26_HUMAN | HUMAN | Serine/threonine-protein kinase 26 OS=Homo sapiens GN=STK26 PE=1 SV=2 | 3,16 | 10,64 | 5 | 14,66 |
| 2313 | sp|Q6FHJ7|SFRP4_HUMAN | HUMAN | Secreted frizzled-related protein 4 OS=Homo sapiens GN=SFRP4 PE=1 SV=2 | 3,16 | 3,22 | 2 | 8,96 |
| 2314 | sp|O14880|MGST3_HUMAN | HUMAN | Microsomal glutathione S-transferase 3 OS=Homo sapiens GN=MGST3 PE=1 SV=1 | 3,15 | 3,22 | 2 | 13,82 |
| 2315 | sp|P03952|KLKB1_HUMAN | HUMAN | Plasma kallikrein OS=Homo sapiens GN=KLKB1 PE=1 SV=1 | 3,14 | 5 | 3 | 4,859 |
| 2316 | sp|P61964|WDR5_HUMAN | HUMAN | WD repeat-containing protein 5 OS=Homo sapiens GN=WDR5 PE=1 SV=1 | 3,14 | 3,2 | 2 | 7,784 |
| 2317 | sp|P07305|H10_HUMAN | HUMAN | Histone H1.0 OS=Homo sapiens GN=H1F0 PE=1 SV=3 | 3,13 | 3,2 | 2 | 11,34 |
| 2318 | sp|Q96PZ0|PUS7_HUMAN | HUMAN | Pseudouridylate synthase 7 homolog OS=Homo sapiens GN=PUS7 PE=1 SV=2 | 3,12 | 3,43 | 2 | 6,051 |
| 2319 | sp|P11387|TOP1_HUMAN | HUMAN | DNA topoisomerase 1 OS=Homo sapiens GN=TOP1 PE=1 SV=2 | 3,12 | 3,27 | 2 | 3,137 |
| 2320 | sp|Q9Y3B8|ORN_HUMAN | HUMAN | Oligoribonuclease, mitochondrial OS=Homo sapiens GN=REXO2 PE=1 SV=3 | 3,12 | 3,22 | 3 | 15,19 |
| 2321 | sp|Q567U6|CCD93_HUMAN | HUMAN | Coiled-coil domain-containing protein 93 OS=Homo sapiens GN=CCDC93 PE=1 SV=2 | 3,11 | 3,27 | 2 | 3,803 |
| 2322 | sp|Q9UHY1|NRBP_HUMAN | HUMAN | Nuclear receptor-binding protein OS=Homo sapiens GN=NRBP1 PE=1 SV=1 | 3,11 | 3,22 | 2 | 4,86 |
| 2323 | sp|O00204|ST2B1_HUMAN | HUMAN | Sulfotransferase family cytosolic 2B member 1 OS=Homo sapiens GN=SULT2B1 PE=1 SV=2 | 3,11 | 3,2 | 2 | 9,589 |
| 2324 | sp|P04155|TFF1_HUMAN | HUMAN | Trefoil factor 1 OS=Homo sapiens GN=TFF1 PE=1 SV=1 | 3,11 | 3,18 | 4 | 44,05 |
| 2325 | sp|P13984|T2FB_HUMAN | HUMAN | General transcription factor IIF subunit 2 OS=Homo sapiens GN=GTF2F2 PE=1 SV=2 | 3,1 | 3,17 | 2 | 12,05 |
| 2326 | sp|O60828|PQBP1_HUMAN | HUMAN | Polyglutamine-binding protein 1 OS=Homo sapiens GN=PQBP1 PE=1 SV=1 | 3,09 | 3,16 | 2 | 15,47 |
| 2327 | sp|P06331|HV209_HUMAN | HUMAN | Ig heavy chain V-II region ARH-77 OS=Homo sapiens PE=4 SV=1 | 3,09 | 3,14 | 6 | 17,12 |
| 2328 | sp|Q92522|H1X_HUMAN | HUMAN | Histone H1x OS=Homo sapiens GN=H1FX PE=1 SV=1 | 3,08 | 3,16 | 2 | 9,859 |
| 2329 | sp|Q7Z4H8|KDEL2_HUMAN | HUMAN | KDEL motif-containing protein 2 OS=Homo sapiens GN=KDELC2 PE=1 SV=2 | 3,08 | 3,14 | 2 | 4,931 |
| 2330 | sp|Q16625|OCLN_HUMAN | HUMAN | Occludin OS=Homo sapiens GN=OCLN PE=1 SV=1 | 3,07 | 3,17 | 3 | 8,238 |
| 2331 | sp|Q92733|PRCC_HUMAN | HUMAN | Proline-rich protein PRCC OS=Homo sapiens GN=PRCC PE=1 SV=1 | 3,07 | 3,14 | 3 | 14,05 |
| 2332 | sp|P62877|RBX1_HUMAN | HUMAN | E3 ubiquitin-protein ligase RBX1 OS=Homo sapiens GN=RBX1 PE=1 SV=1 | 3,07 | 3,12 | 2 | 24,07 |
| 2333 | sp|P07358|CO8B_HUMAN | HUMAN | Complement component C8 beta chain OS=Homo sapiens GN=C8B PE=1 SV=3 | 3,07 | 3,12 | 2 | 4,399 |
| 2334 | sp|P49023|PAXI_HUMAN | HUMAN | Paxillin OS=Homo sapiens GN=PXN PE=1 SV=3 | 3,06 | 3,14 | 2 | 4,399 |
| 2335 | sp|P35080|PROF2_HUMAN | HUMAN | Profilin-2 OS=Homo sapiens GN=PFN2 PE=1 SV=3 | 3,06 | 3,1 | 2 | 20,71 |
| 2336 | sp|P43405|KSYK_HUMAN | HUMAN | Tyrosine-protein kinase SYK OS=Homo sapiens GN=SYK PE=1 SV=1 | 3,04 | 3,21 | 3 | 5,827 |
| 2337 | sp|P10720|PF4V_HUMAN | HUMAN | Platelet factor 4 variant OS=Homo sapiens GN=PF4V1 PE=1 SV=1 | 3,04 | 3,14 | 2 | 23,08 |
| 2338 | sp|Q9H3R2|MUC13_HUMAN | HUMAN | Mucin-13 OS=Homo sapiens GN=MUC13 PE=1 SV=3 | 3,04 | 3,13 | 3 | 6,25 |
| 2339 | sp|P42025|ACTY_HUMAN | HUMAN | Beta-centractin OS=Homo sapiens GN=ACTR1B PE=1 SV=1 | 3,03 | 13,74 | 9 | 31,91 |
| 2340 | sp|O75356|ENTP5_HUMAN | HUMAN | Ectonucleoside triphosphate diphosphohydrolase 5 OS=Homo sapiens GN=ENTPD5 PE=1 SV=1 | 3,03 | 3,14 | 2 | 8,178 |
| 2341 | sp|Q92572|AP3S1_HUMAN | HUMAN | AP-3 complex subunit sigma-1 OS=Homo sapiens GN=AP3S1 PE=1 SV=1 | 3,03 | 3,08 | 2 | 13,47 |
| 2342 | sp|P0DJI8|SAA1_HUMAN | HUMAN | Serum amyloid A-1 protein OS=Homo sapiens GN=SAA1 PE=1 SV=1 | 3,02 | 6,29 | 4 | 47,54 |
| 2343 | sp|O43169|CYB5B_HUMAN | HUMAN | Cytochrome b5 type B OS=Homo sapiens GN=CYB5B PE=1 SV=2 | 3,02 | 3,27 | 2 | 31,51 |
| 2344 | sp|Q9NUJ1|ABHDA_HUMAN | HUMAN | Mycophenolic acid acyl-glucuronide esterase, mitochondrial OS=Homo sapiens GN=ABHD10 PE=1 SV=1 | 3,02 | 3,16 | 3 | 10,13 |
| 2345 | sp|Q5RKV6|EXOS6_HUMAN | HUMAN | Exosome complex component MTR3 OS=Homo sapiens GN=EXOSC6 PE=1 SV=1 | 3,02 | 3,14 | 2 | 9,191 |
| 2346 | sp|Q9Y6C2|EMIL1_HUMAN | HUMAN | EMILIN-1 OS=Homo sapiens GN=EMILIN1 PE=1 SV=2 | 3,01 | 3,17 | 2 | 2,756 |
| 2347 | sp|O43633|CHM2A_HUMAN | HUMAN | Charged multivesicular body protein 2a OS=Homo sapiens GN=CHMP2A PE=1 SV=1 | 3,01 | 3,06 | 2 | 7,207 |
| 2348 | sp|Q13043|STK4_HUMAN | HUMAN | Serine/threonine-protein kinase 4 OS=Homo sapiens GN=STK4 PE=1 SV=2 | 3 | 3,07 | 2 | 5,544 |
| 2349 | sp|Q9P0V9|SEP10_HUMAN | HUMAN | Septin-10 OS=Homo sapiens GN=SEPT10 PE=1 SV=2 | 2,99 | 5,66 | 5 | 12,33 |
| 2350 | sp|P35613|BASI_HUMAN | HUMAN | Basigin OS=Homo sapiens GN=BSG PE=1 SV=2 | 2,99 | 3,13 | 3 | 8,571 |
| 2351 | sp|Q04941|PLP2_HUMAN | HUMAN | Proteolipid protein 2 OS=Homo sapiens GN=PLP2 PE=1 SV=1 | 2,99 | 3,04 | 2 | 18,42 |
| 2352 | sp|Q99543|DNJC2_HUMAN | HUMAN | DnaJ homolog subfamily C member 2 OS=Homo sapiens GN=DNAJC2 PE=1 SV=4 | 2,97 | 3,16 | 3 | 5,153 |
| 2353 | sp|P19388|RPAB1_HUMAN | HUMAN | DNA-directed RNA polymerases I, II, and III subunit RPABC1 OS=Homo sapiens GN=POLR2E PE=1 SV=4 | 2,97 | 3,12 | 2 | 14,76 |
| 2354 | sp|Q9BY42|RTF2_HUMAN | HUMAN | Protein RTF2 homolog OS=Homo sapiens GN=RTFDC1 PE=1 SV=3 | 2,97 | 3,11 | 2 | 10,13 |
| 2355 | sp|P35558|PCKGC_HUMAN | HUMAN | Phosphoenolpyruvate carboxykinase, cytosolic [GTP] OS=Homo sapiens GN=PCK1 PE=1 SV=3 | 2,96 | 5,19 | 4 | 7,878 |
| 2356 | sp|O76071|CIAO1_HUMAN | HUMAN | Probable cytosolic iron-sulfur protein assembly protein CIAO1 OS=Homo sapiens GN=CIAO1 PE=1 SV=1 | 2,96 | 3,11 | 4 | 16,81 |
| 2357 | sp|Q92835|SHIP1_HUMAN | HUMAN | Phosphatidylinositol 3,4,5-trisphosphate 5-phosphatase 1 OS=Homo sapiens GN=INPP5D PE=1 SV=2 | 2,95 | 3,23 | 2 | 2,103 |
| 2358 | sp|Q9NPF4|OSGEP_HUMAN | HUMAN | Probable tRNA N6-adenosine threonylcarbamoyltransferase OS=Homo sapiens GN=OSGEP PE=1 SV=1 | 2,94 | 3,02 | 2 | 8,657 |
| 2359 | sp|Q71UI9|H2AV_HUMAN | HUMAN | Histone H2A.V OS=Homo sapiens GN=H2AFV PE=1 SV=3 | 2,93 | 7,14 | 6 | 31,25 |
| 2360 | sp|O75323|NIPS2_HUMAN | HUMAN | Protein NipSnap homolog 2 OS=Homo sapiens GN=GBAS PE=1 SV=1 | 2,93 | 3,27 | 3 | 8,741 |
| 2361 | sp|Q14160|SCRIB_HUMAN | HUMAN | Protein scribble homolog OS=Homo sapiens GN=SCRIB PE=1 SV=4 | 2,93 | 3,18 | 3 | 1,963 |
| 2362 | sp|P46734|MP2K3_HUMAN | HUMAN | Dual specificity mitogen-activated protein kinase kinase 3 OS=Homo sapiens GN=MAP2K3 PE=1 SV=2 | 2,93 | 3,07 | 3 | 9,798 |
| 2363 | sp|Q8TBX8|PI42C_HUMAN | HUMAN | Phosphatidylinositol 5-phosphate 4-kinase type-2 gamma OS=Homo sapiens GN=PIP4K2C PE=1 SV=3 | 2,93 | 3,06 | 4 | 15,2 |
| 2364 | sp|Q96SI9|STRBP_HUMAN | HUMAN | Spermatid perinuclear RNA-binding protein OS=Homo sapiens GN=STRBP PE=1 SV=1 | 2,92 | 15,44 | 8 | 10,27 |
| 2365 | sp|Q9Y2J2|E41L3_HUMAN | HUMAN | Band 4.1-like protein 3 OS=Homo sapiens GN=EPB41L3 PE=1 SV=2 | 2,92 | 5,88 | 5 | 5,244 |
| 2366 | sp|Q15833|STXB2_HUMAN | HUMAN | Syntaxin-binding protein 2 OS=Homo sapiens GN=STXBP2 PE=1 SV=2 | 2,92 | 3,37 | 2 | 5,059 |
| 2367 | sp|P27144|KAD4_HUMAN | HUMAN | Adenylate kinase 4, mitochondrial OS=Homo sapiens GN=AK4 PE=1 SV=1 | 2,92 | 3,06 | 3 | 14,35 |
| 2368 | sp|Q969E4|TCAL3_HUMAN | HUMAN | Transcription elongation factor A protein-like 3 OS=Homo sapiens GN=TCEAL3 PE=1 SV=1 | 2,92 | 3,01 | 2 | 16,5 |
| 2369 | sp|Q9Y508|RN114_HUMAN | HUMAN | E3 ubiquitin-protein ligase RNF114 OS=Homo sapiens GN=RNF114 PE=1 SV=1 | 2,92 | 3 | 2 | 14,04 |
| 2370 | sp|Q9NXR1|NDE1_HUMAN | HUMAN | Nuclear distribution protein nudE homolog 1 OS=Homo sapiens GN=NDE1 PE=1 SV=2 | 2,92 | 2,99 | 2 | 6,647 |
| 2371 | sp|O00469|PLOD2_HUMAN | HUMAN | Procollagen-lysine,2-oxoglutarate 5-dioxygenase 2 OS=Homo sapiens GN=PLOD2 PE=1 SV=2 | 2,91 | 3 | 2 | 3,664 |
| 2372 | sp|O75792|RNH2A_HUMAN | HUMAN | Ribonuclease H2 subunit A OS=Homo sapiens GN=RNASEH2A PE=1 SV=2 | 2,9 | 3,27 | 2 | 10,03 |
| 2373 | sp|O43837|IDH3B_HUMAN | HUMAN | Isocitrate dehydrogenase [NAD] subunit beta, mitochondrial OS=Homo sapiens GN=IDH3B PE=1 SV=2 | 2,9 | 2,96 | 2 | 8,571 |
| 2374 | sp|O00483|NDUA4_HUMAN | HUMAN | Cytochrome c oxidase subunit NDUFA4 OS=Homo sapiens GN=NDUFA4 PE=1 SV=1 | 2,9 | 2,95 | 2 | 27,16 |
| 2375 | sp|Q9UBI1|COMD3_HUMAN | HUMAN | COMM domain-containing protein 3 OS=Homo sapiens GN=COMMD3 PE=1 SV=1 | 2,89 | 2,97 | 2 | 11,79 |
| 2376 | sp|P43897|EFTS_HUMAN | HUMAN | Elongation factor Ts, mitochondrial OS=Homo sapiens GN=TSFM PE=1 SV=2 | 2,88 | 3,03 | 4 | 18,15 |
| 2377 | sp|Q92797|SYMPK_HUMAN | HUMAN | Symplekin OS=Homo sapiens GN=SYMPK PE=1 SV=2 | 2,88 | 2,94 | 2 | 2,198 |
| 2378 | sp|P05204|HMGN2_HUMAN | HUMAN | Non-histone chromosomal protein HMG-17 OS=Homo sapiens GN=HMGN2 PE=1 SV=3 | 2,88 | 2,92 | 2 | 21,11 |
| 2379 | sp|Q14116|IL18_HUMAN | HUMAN | Interleukin-18 OS=Homo sapiens GN=IL18 PE=1 SV=1 | 2,86 | 2,95 | 2 | 8,808 |
| 2380 | sp|P45973|CBX5_HUMAN | HUMAN | Chromobox protein homolog 5 OS=Homo sapiens GN=CBX5 PE=1 SV=1 | 2,86 | 2,94 | 2 | 13,61 |
| 2381 | sp|Q8WZ42|TITIN_HUMAN | HUMAN | Titin OS=Homo sapiens GN=TTN PE=1 SV=4 | 2,85 | 5,12 | 3 | 0,06405 |
| 2382 | sp|P36915|GNL1_HUMAN | HUMAN | Guanine nucleotide-binding protein-like 1 OS=Homo sapiens GN=GNL1 PE=1 SV=2 | 2,84 | 2,89 | 2 | 4,119 |
| 2383 | sp|O75891|AL1L1_HUMAN | HUMAN | Cytosolic 10-formyltetrahydrofolate dehydrogenase OS=Homo sapiens GN=ALDH1L1 PE=1 SV=2 | 2,83 | 4,34 | 2 | 2,55 |
| 2384 | sp|Q9Y2X7|GIT1_HUMAN | HUMAN | ARF GTPase-activating protein GIT1 OS=Homo sapiens GN=GIT1 PE=1 SV=2 | 2,83 | 2,98 | 2 | 2,497 |
| 2385 | sp|Q14061|COX17_HUMAN | HUMAN | Cytochrome c oxidase copper chaperone OS=Homo sapiens GN=COX17 PE=1 SV=2 | 2,83 | 2,87 | 4 | 36,51 |
| 2386 | sp|P36507|MP2K2_HUMAN | HUMAN | Dual specificity mitogen-activated protein kinase kinase 2 OS=Homo sapiens GN=MAP2K2 PE=1 SV=1 | 2,82 | 9,64 | 6 | 22 |
| 2387 | sp|P67812|SC11A_HUMAN | HUMAN | Signal peptidase complex catalytic subunit SEC11A OS=Homo sapiens GN=SEC11A PE=1 SV=1 | 2,81 | 2,86 | 2 | 11,17 |
| 2388 | sp|P04220|MUCB_HUMAN | HUMAN | Ig mu heavy chain disease protein OS=Homo sapiens PE=1 SV=1 | 2,8 | 23,85 | 18 | 43,99 |
| 2389 | sp|P24298|ALAT1_HUMAN | HUMAN | Alanine aminotransferase 1 OS=Homo sapiens GN=GPT PE=1 SV=3 | 2,8 | 2,98 | 4 | 20,36 |
| 2390 | sp|O95319|CELF2_HUMAN | HUMAN | CUGBP Elav-like family member 2 OS=Homo sapiens GN=CELF2 PE=1 SV=1 | 2,8 | 2,91 | 2 | 5,118 |
| 2391 | sp|P05156|CFAI_HUMAN | HUMAN | Complement factor I OS=Homo sapiens GN=CFI PE=1 SV=2 | 2,8 | 2,87 | 1 | 2,23 |
| 2392 | sp|Q9UQE7|SMC3_HUMAN | HUMAN | Structural maintenance of chromosomes protein 3 OS=Homo sapiens GN=SMC3 PE=1 SV=2 | 2,79 | 3,39 | 2 | 2,054 |
| 2393 | sp|Q96BW5|PTER_HUMAN | HUMAN | Phosphotriesterase-related protein OS=Homo sapiens GN=PTER PE=1 SV=1 | 2,79 | 2,88 | 2 | 6,59 |
| 2394 | sp|O60237|MYPT2_HUMAN | HUMAN | Protein phosphatase 1 regulatory subunit 12B OS=Homo sapiens GN=PPP1R12B PE=1 SV=2 | 2,78 | 4,57 | 3 | 3,36 |
| 2395 | sp|Q14919|NC2A_HUMAN | HUMAN | Dr1-associated corepressor OS=Homo sapiens GN=DRAP1 PE=1 SV=3 | 2,77 | 2,91 | 2 | 9,268 |
| 2396 | sp|P51161|FABP6_HUMAN | HUMAN | Gastrotropin OS=Homo sapiens GN=FABP6 PE=1 SV=2 | 2,76 | 2,88 | 4 | 22,66 |
| 2397 | sp|Q86YP4|P66A_HUMAN | HUMAN | Transcriptional repressor p66-alpha OS=Homo sapiens GN=GATAD2A PE=1 SV=1 | 2,76 | 2,82 | 1 | 1,738 |
| 2398 | sp|Q9UNP9|PPIE_HUMAN | HUMAN | Peptidyl-prolyl cis-trans isomerase E OS=Homo sapiens GN=PPIE PE=1 SV=1 | 2,74 | 3,14 | 4 | 16,61 |
| 2399 | sp|P16157|ANK1_HUMAN | HUMAN | Ankyrin-1 OS=Homo sapiens GN=ANK1 PE=1 SV=3 | 2,74 | 2,86 | 2 | 1,17 |
| 2400 | sp|Q9H832|UBE2Z_HUMAN | HUMAN | Ubiquitin-conjugating enzyme E2 Z OS=Homo sapiens GN=UBE2Z PE=1 SV=2 | 2,73 | 2,84 | 2 | 7,062 |
| 2401 | sp|Q9Y2Q5|LTOR2_HUMAN | HUMAN | Ragulator complex protein LAMTOR2 OS=Homo sapiens GN=LAMTOR2 PE=1 SV=1 | 2,73 | 2,82 | 2 | 21,6 |
| 2402 | sp|Q92917|GPKOW_HUMAN | HUMAN | G patch domain and KOW motifs-containing protein OS=Homo sapiens GN=GPKOW PE=1 SV=2 | 2,72 | 2,86 | 2 | 5,252 |
| 2403 | sp|Q9H3S7|PTN23_HUMAN | HUMAN | Tyrosine-protein phosphatase non-receptor type 23 OS=Homo sapiens GN=PTPN23 PE=1 SV=1 | 2,71 | 2,84 | 2 | 1,773 |
| 2404 | sp|Q96JB2|COG3_HUMAN | HUMAN | Conserved oligomeric Golgi complex subunit 3 OS=Homo sapiens GN=COG3 PE=1 SV=3 | 2,7 | 2,84 | 2 | 4,348 |
| 2405 | sp|A5YKK6|CNOT1_HUMAN | HUMAN | CCR4-NOT transcription complex subunit 1 OS=Homo sapiens GN=CNOT1 PE=1 SV=2 | 2,69 | 2,77 | 2 | 0,7997 |
| 2406 | sp|Q8IYI6|EXOC8_HUMAN | HUMAN | Exocyst complex component 8 OS=Homo sapiens GN=EXOC8 PE=1 SV=2 | 2,68 | 3,39 | 3 | 8,138 |
| 2407 | sp|O00468|AGRIN_HUMAN | HUMAN | Agrin OS=Homo sapiens GN=AGRN PE=1 SV=5 | 2,68 | 2,77 | 2 | 1,016 |
| 2408 | sp|Q9BVJ7|DUS23_HUMAN | HUMAN | Dual specificity protein phosphatase 23 OS=Homo sapiens GN=DUSP23 PE=1 SV=1 | 2,68 | 2,72 | 2 | 15,33 |
| 2409 | sp|P50148|GNAQ_HUMAN | HUMAN | Guanine nucleotide-binding protein G(q) subunit alpha OS=Homo sapiens GN=GNAQ PE=1 SV=4 | 2,68 | 2,71 | 2 | 8,357 |
| 2410 | sp|Q9BSE5|SPEB_HUMAN | HUMAN | Agmatinase, mitochondrial OS=Homo sapiens GN=AGMAT PE=1 SV=2 | 2,67 | 2,73 | 2 | 11,65 |
| 2411 | sp|P26358|DNMT1_HUMAN | HUMAN | DNA (cytosine-5)-methyltransferase 1 OS=Homo sapiens GN=DNMT1 PE=1 SV=2 | 2,66 | 2,83 | 2 | 1,671 |
| 2412 | sp|Q9UPT5|EXOC7_HUMAN | HUMAN | Exocyst complex component 7 OS=Homo sapiens GN=EXOC7 PE=1 SV=3 | 2,66 | 2,71 | 2 | 2,993 |
| 2413 | sp|Q9BY77|PDIP3_HUMAN | HUMAN | Polymerase delta-interacting protein 3 OS=Homo sapiens GN=POLDIP3 PE=1 SV=2 | 2,65 | 2,7 | 2 | 5,463 |
| 2414 | sp|Q8N3U4|STAG2_HUMAN | HUMAN | Cohesin subunit SA-2 OS=Homo sapiens GN=STAG2 PE=1 SV=3 | 2,64 | 2,7 | 1 | 2,031 |
| 2415 | sp|Q9BV68|RN126_HUMAN | HUMAN | E3 ubiquitin-protein ligase RNF126 OS=Homo sapiens GN=RNF126 PE=1 SV=1 | 2,64 | 2,67 | 2 | 11,66 |
| 2416 | sp|P31483|TIA1_HUMAN | HUMAN | Nucleolysin TIA-1 isoform p40 OS=Homo sapiens GN=TIA1 PE=1 SV=3 | 2,63 | 5,17 | 4 | 9,067 |
| 2417 | sp|Q96T76|MMS19_HUMAN | HUMAN | MMS19 nucleotide excision repair protein homolog OS=Homo sapiens GN=MMS19 PE=1 SV=2 | 2,63 | 2,75 | 2 | 3,301 |
| 2418 | sp|O75940|SPF30_HUMAN | HUMAN | Survival of motor neuron-related-splicing factor 30 OS=Homo sapiens GN=SMNDC1 PE=1 SV=1 | 2,63 | 2,67 | 2 | 15,55 |
| 2419 | sp|Q9BPW8|NIPS1_HUMAN | HUMAN | Protein NipSnap homolog 1 OS=Homo sapiens GN=NIPSNAP1 PE=1 SV=1 | 2,62 | 2,77 | 2 | 4,93 |
| 2420 | sp|O75795|UDB17_HUMAN | HUMAN | UDP-glucuronosyltransferase 2B17 OS=Homo sapiens GN=UGT2B17 PE=1 SV=1 | 2,62 | 2,75 | 3 | 5,849 |
| 2421 | sp|O00161|SNP23_HUMAN | HUMAN | Synaptosomal-associated protein 23 OS=Homo sapiens GN=SNAP23 PE=1 SV=1 | 2,62 | 2,73 | 3 | 19,91 |
| 2422 | sp|Q99747|SNAG_HUMAN | HUMAN | Gamma-soluble NSF attachment protein OS=Homo sapiens GN=NAPG PE=1 SV=1 | 2,62 | 2,72 | 2 | 7,372 |
| 2423 | sp|O95487|SC24B_HUMAN | HUMAN | Protein transport protein Sec24B OS=Homo sapiens GN=SEC24B PE=1 SV=2 | 2,61 | 2,7 | 2 | 2,603 |
| 2424 | sp|Q8NBJ4|GOLM1_HUMAN | HUMAN | Golgi membrane protein 1 OS=Homo sapiens GN=GOLM1 PE=1 SV=1 | 2,6 | 2,72 | 3 | 9,726 |
| 2425 | sp|Q7LBC6|KDM3B_HUMAN | HUMAN | Lysine-specific demethylase 3B OS=Homo sapiens GN=KDM3B PE=1 SV=2 | 2,6 | 2,64 | 2 | 1,42 |
| 2426 | sp|P37840|SYUA_HUMAN | HUMAN | Alpha-synuclein OS=Homo sapiens GN=SNCA PE=1 SV=1 | 2,6 | 2,64 | 2 | 25,71 |
| 2427 | sp|Q9NRF9|DPOE3_HUMAN | HUMAN | DNA polymerase epsilon subunit 3 OS=Homo sapiens GN=POLE3 PE=1 SV=1 | 2,59 | 2,67 | 2 | 23,13 |
| 2428 | sp|O14908|GIPC1_HUMAN | HUMAN | PDZ domain-containing protein GIPC1 OS=Homo sapiens GN=GIPC1 PE=1 SV=2 | 2,58 | 3,38 | 2 | 8,108 |
| 2429 | sp|P23743|DGKA_HUMAN | HUMAN | Diacylglycerol kinase alpha OS=Homo sapiens GN=DGKA PE=1 SV=3 | 2,57 | 2,61 | 2 | 4,354 |
| 2430 | sp|O15514|RPB4_HUMAN | HUMAN | DNA-directed RNA polymerase II subunit RPB4 OS=Homo sapiens GN=POLR2D PE=1 SV=1 | 2,57 | 2,61 | 2 | 23,94 |
| 2431 | sp|Q4G0J3|LARP7_HUMAN | HUMAN | La-related protein 7 OS=Homo sapiens GN=LARP7 PE=1 SV=1 | 2,56 | 2,66 | 2 | 4,467 |
| 2432 | sp|P48507|GSH0_HUMAN | HUMAN | Glutamate--cysteine ligase regulatory subunit OS=Homo sapiens GN=GCLM PE=1 SV=1 | 2,56 | 2,66 | 1 | 4,745 |
| 2433 | sp|Q9BTE6|AASD1_HUMAN | HUMAN | Alanyl-tRNA editing protein Aarsd1 OS=Homo sapiens GN=AARSD1 PE=1 SV=2 | 2,56 | 2,59 | 2 | 7,282 |
| 2434 | sp|P61619|S61A1_HUMAN | HUMAN | Protein transport protein Sec61 subunit alpha isoform 1 OS=Homo sapiens GN=SEC61A1 PE=1 SV=2 | 2,55 | 2,65 | 2 | 3,571 |
| 2435 | sp|O43264|ZW10_HUMAN | HUMAN | Centromere/kinetochore protein zw10 homolog OS=Homo sapiens GN=ZW10 PE=1 SV=3 | 2,55 | 2,63 | 2 | 3,466 |
| 2436 | sp|Q13094|LCP2_HUMAN | HUMAN | Lymphocyte cytosolic protein 2 OS=Homo sapiens GN=LCP2 PE=1 SV=1 | 2,55 | 2,58 | 2 | 5,253 |
| 2437 | sp|Q05048|CSTF1_HUMAN | HUMAN | Cleavage stimulation factor subunit 1 OS=Homo sapiens GN=CSTF1 PE=1 SV=1 | 2,55 | 2,58 | 2 | 9,049 |
| 2438 | sp|P36980|FHR2_HUMAN | HUMAN | Complement factor H-related protein 2 OS=Homo sapiens GN=CFHR2 PE=1 SV=1 | 2,54 | 7,15 | 4 | 17,41 |
| 2439 | sp|P07711|CATL1_HUMAN | HUMAN | Cathepsin L1 OS=Homo sapiens GN=CTSL PE=1 SV=2 | 2,54 | 3,32 | 4 | 12,31 |
| 2440 | sp|Q9BU89|DOHH_HUMAN | HUMAN | Deoxyhypusine hydroxylase OS=Homo sapiens GN=DOHH PE=1 SV=1 | 2,54 | 2,61 | 2 | 11,92 |
| 2441 | sp|Q9UII2|ATIF1_HUMAN | HUMAN | ATPase inhibitor, mitochondrial OS=Homo sapiens GN=ATPIF1 PE=1 SV=1 | 2,53 | 2,6 | 3 | 25,47 |
| 2442 | sp|Q92804|RBP56_HUMAN | HUMAN | TATA-binding protein-associated factor 2N OS=Homo sapiens GN=TAF15 PE=1 SV=1 | 2,52 | 6,66 | 5 | 10,98 |
| 2443 | sp|Q9UBS4|DJB11_HUMAN | HUMAN | DnaJ homolog subfamily B member 11 OS=Homo sapiens GN=DNAJB11 PE=1 SV=1 | 2,52 | 2,55 | 2 | 8,38 |
| 2444 | sp|P01772|HV311_HUMAN | HUMAN | Ig heavy chain V-III region KOL OS=Homo sapiens PE=1 SV=1 | 2,52 | 2,55 | 4 | 12,7 |
| 2445 | sp|Q9Y624|JAM1_HUMAN | HUMAN | Junctional adhesion molecule A OS=Homo sapiens GN=F11R PE=1 SV=1 | 2,52 | 2,55 | 2 | 6,355 |
| 2446 | sp|Q86WA6|BPHL_HUMAN | HUMAN | Valacyclovir hydrolase OS=Homo sapiens GN=BPHL PE=1 SV=1 | 2,52 | 2,55 | 2 | 9,622 |
| 2447 | sp|Q96DE0|NUD16_HUMAN | HUMAN | U8 snoRNA-decapping enzyme OS=Homo sapiens GN=NUDT16 PE=1 SV=2 | 2,51 | 2,61 | 2 | 16,92 |
| 2448 | sp|Q86U38|NOP9_HUMAN | HUMAN | Nucleolar protein 9 OS=Homo sapiens GN=NOP9 PE=1 SV=1 | 2,5 | 3,5 | 4 | 5,189 |
| 2449 | sp|Q9UID3|VPS51_HUMAN | HUMAN | Vacuolar protein sorting-associated protein 51 homolog OS=Homo sapiens GN=VPS51 PE=1 SV=2 | 2,5 | 2,58 | 2 | 4,476 |
| 2450 | sp|Q9HD45|TM9S3_HUMAN | HUMAN | Transmembrane 9 superfamily member 3 OS=Homo sapiens GN=TM9SF3 PE=1 SV=2 | 2,5 | 2,55 | 1 | 4,414 |
| 2451 | sp|P29120|NEC1_HUMAN | HUMAN | Neuroendocrine convertase 1 OS=Homo sapiens GN=PCSK1 PE=1 SV=2 | 2,49 | 2,57 | 2 | 2,656 |
| 2452 | sp|Q14CX7|NAA25_HUMAN | HUMAN | N-alpha-acetyltransferase 25, NatB auxiliary subunit OS=Homo sapiens GN=NAA25 PE=1 SV=1 | 2,49 | 2,54 | 1 | 1,852 |
| 2453 | sp|O43493|TGON2_HUMAN | HUMAN | Trans-Golgi network integral membrane protein 2 OS=Homo sapiens GN=TGOLN2 PE=1 SV=2 | 2,48 | 2,76 | 3 | 5,208 |
| 2454 | sp|Q9Y316|MEMO1_HUMAN | HUMAN | Protein MEMO1 OS=Homo sapiens GN=MEMO1 PE=1 SV=1 | 2,48 | 2,52 | 2 | 9,428 |
| 2455 | sp|Q9BV20|MTNA_HUMAN | HUMAN | Methylthioribose-1-phosphate isomerase OS=Homo sapiens GN=MRI1 PE=1 SV=1 | 2,47 | 2,56 | 2 | 5,691 |
| 2456 | sp|Q92620|PRP16_HUMAN | HUMAN | Pre-mRNA-splicing factor ATP-dependent RNA helicase PRP16 OS=Homo sapiens GN=DHX38 PE=1 SV=2 | 2,47 | 2,53 | 2 | 2,2 |
| 2457 | sp|Q9H0E2|TOLIP_HUMAN | HUMAN | Toll-interacting protein OS=Homo sapiens GN=TOLLIP PE=1 SV=1 | 2,47 | 2,5 | 1 | 4,745 |
| 2458 | sp|Q9UJZ1|STML2_HUMAN | HUMAN | Stomatin-like protein 2, mitochondrial OS=Homo sapiens GN=STOML2 PE=1 SV=1 | 2,46 | 2,6 | 3 | 10,67 |
| 2459 | sp|Q96A65|EXOC4_HUMAN | HUMAN | Exocyst complex component 4 OS=Homo sapiens GN=EXOC4 PE=1 SV=1 | 2,46 | 2,53 | 1 | 1,54 |
| 2460 | sp|Q9UBP6|TRMB_HUMAN | HUMAN | tRNA (guanine-N(7)-)-methyltransferase OS=Homo sapiens GN=METTL1 PE=1 SV=1 | 2,45 | 2,52 | 1 | 8,696 |
| 2461 | sp|O94906|PRP6_HUMAN | HUMAN | Pre-mRNA-processing factor 6 OS=Homo sapiens GN=PRPF6 PE=1 SV=1 | 2,44 | 2,67 | 4 | 4,888 |
| 2462 | sp|O75995|SASH3_HUMAN | HUMAN | SAM and SH3 domain-containing protein 3 OS=Homo sapiens GN=SASH3 PE=1 SV=2 | 2,44 | 2,48 | 1 | 4,211 |
| 2463 | sp|Q92538|GBF1_HUMAN | HUMAN | Golgi-specific brefeldin A-resistance guanine nucleotide exchange factor 1 OS=Homo sapiens GN=GBF1 PE=1 SV=2 | 2,42 | 3,11 | 4 | 1,937 |
| 2464 | sp|Q15005|SPCS2_HUMAN | HUMAN | Signal peptidase complex subunit 2 OS=Homo sapiens GN=SPCS2 PE=1 SV=3 | 2,42 | 2,52 | 3 | 11,95 |
| 2465 | sp|Q99611|SPS2_HUMAN | HUMAN | Selenide, water dikinase 2 OS=Homo sapiens GN=SEPHS2 PE=1 SV=3 | 2,41 | 2,46 | 2 | 4,911 |
| 2466 | sp|Q9H098|F107B_HUMAN | HUMAN | Protein FAM107B OS=Homo sapiens GN=FAM107B PE=1 SV=1 | 2,41 | 2,44 | 2 | 16,03 |
| 2467 | sp|Q9Y2Y8|PRG3_HUMAN | HUMAN | Proteoglycan 3 OS=Homo sapiens GN=PRG3 PE=1 SV=2 | 2,41 | 2,44 | 1 | 4,889 |
| 2468 | sp|P16083|NQO2_HUMAN | HUMAN | Ribosyldihydronicotinamide dehydrogenase [quinone] OS=Homo sapiens GN=NQO2 PE=1 SV=5 | 2,41 | 2,43 | 1 | 7,792 |
| 2469 | sp|Q9UBU9|NXF1_HUMAN | HUMAN | Nuclear RNA export factor 1 OS=Homo sapiens GN=NXF1 PE=1 SV=1 | 2,4 | 2,49 | 2 | 5,493 |
| 2470 | sp|Q8IUD2|RB6I2_HUMAN | HUMAN | ELKS/Rab6-interacting/CAST family member 1 OS=Homo sapiens GN=ERC1 PE=1 SV=1 | 2,39 | 2,5 | 1 | 0,5376 |
| 2471 | sp|Q15287|RNPS1_HUMAN | HUMAN | RNA-binding protein with serine-rich domain 1 OS=Homo sapiens GN=RNPS1 PE=1 SV=1 | 2,39 | 2,42 | 1 | 4,918 |
| 2472 | sp|Q2M389|WASH7_HUMAN | HUMAN | WASH complex subunit 7 OS=Homo sapiens GN=KIAA1033 PE=1 SV=2 | 2,39 | 2,41 | 3 | 3,581 |
| 2473 | sp|Q8WUA2|PPIL4_HUMAN | HUMAN | Peptidyl-prolyl cis-trans isomerase-like 4 OS=Homo sapiens GN=PPIL4 PE=1 SV=1 | 2,38 | 2,46 | 2 | 6,098 |
| 2474 | sp|P02745|C1QA_HUMAN | HUMAN | Complement C1q subcomponent subunit A OS=Homo sapiens GN=C1QA PE=1 SV=2 | 2,38 | 2,41 | 2 | 9,388 |
| 2475 | sp|Q92930|RAB8B_HUMAN | HUMAN | Ras-related protein Rab-8B OS=Homo sapiens GN=RAB8B PE=1 SV=2 | 2,37 | 8,42 | 9 | 25,6 |
| 2476 | sp|Q9BYT8|NEUL_HUMAN | HUMAN | Neurolysin, mitochondrial OS=Homo sapiens GN=NLN PE=1 SV=1 | 2,37 | 3,68 | 3 | 4,972 |
| 2477 | sp|P06316|LV107_HUMAN | HUMAN | Ig lambda chain V-I region BL2 OS=Homo sapiens PE=2 SV=1 | 2,37 | 3,38 | 3 | 25,38 |
| 2478 | sp|P24390|ERD21_HUMAN | HUMAN | ER lumen protein-retaining receptor 1 OS=Homo sapiens GN=KDELR1 PE=1 SV=1 | 2,37 | 2,45 | 2 | 8,962 |
| 2479 | sp|Q15050|RRS1_HUMAN | HUMAN | Ribosome biogenesis regulatory protein homolog OS=Homo sapiens GN=RRS1 PE=1 SV=2 | 2,37 | 2,4 | 2 | 9,863 |
| 2480 | sp|Q9NRW7|VPS45_HUMAN | HUMAN | Vacuolar protein sorting-associated protein 45 OS=Homo sapiens GN=VPS45 PE=1 SV=1 | 2,37 | 2,4 | 1 | 2,456 |
| 2481 | sp|Q15382|RHEB_HUMAN | HUMAN | GTP-binding protein Rheb OS=Homo sapiens GN=RHEB PE=1 SV=1 | 2,36 | 4,2 | 2 | 12,5 |
| 2482 | sp|A0FGR8|ESYT2_HUMAN | HUMAN | Extended synaptotagmin-2 OS=Homo sapiens GN=ESYT2 PE=1 SV=1 | 2,36 | 2,47 | 2 | 2,932 |
| 2483 | sp|P51808|DYLT3_HUMAN | HUMAN | Dynein light chain Tctex-type 3 OS=Homo sapiens GN=DYNLT3 PE=1 SV=1 | 2,35 | 2,38 | 1 | 14,66 |
| 2484 | sp|Q16595|FRDA_HUMAN | HUMAN | Frataxin, mitochondrial OS=Homo sapiens GN=FXN PE=1 SV=2 | 2,35 | 2,37 | 2 | 10,95 |
| 2485 | sp|O75688|PPM1B_HUMAN | HUMAN | Protein phosphatase 1B OS=Homo sapiens GN=PPM1B PE=1 SV=1 | 2,34 | 5,12 | 4 | 9,186 |
| 2486 | sp|P06132|DCUP_HUMAN | HUMAN | Uroporphyrinogen decarboxylase OS=Homo sapiens GN=UROD PE=1 SV=2 | 2,34 | 2,49 | 1 | 6,812 |
| 2487 | sp|O94826|TOM70_HUMAN | HUMAN | Mitochondrial import receptor subunit TOM70 OS=Homo sapiens GN=TOMM70A PE=1 SV=1 | 2,34 | 2,43 | 1 | 2,961 |
| 2488 | sp|P82970|HMGN5_HUMAN | HUMAN | High mobility group nucleosome-binding domain-containing protein 5 OS=Homo sapiens GN=HMGN5 PE=1 SV=1 | 2,34 | 2,42 | 3 | 13,12 |
| 2489 | sp|O75694|NU155_HUMAN | HUMAN | Nuclear pore complex protein Nup155 OS=Homo sapiens GN=NUP155 PE=1 SV=1 | 2,34 | 2,4 | 1 | 1,15 |
| 2490 | sp|P10451|OSTP_HUMAN | HUMAN | Osteopontin OS=Homo sapiens GN=SPP1 PE=1 SV=1 | 2,34 | 2,35 | 1 | 4,14 |
| 2491 | sp|P60510|PP4C_HUMAN | HUMAN | Serine/threonine-protein phosphatase 4 catalytic subunit OS=Homo sapiens GN=PPP4C PE=1 SV=1 | 2,33 | 4,41 | 3 | 8,795 |
| 2492 | sp|Q9UNF1|MAGD2_HUMAN | HUMAN | Melanoma-associated antigen D2 OS=Homo sapiens GN=MAGED2 PE=1 SV=2 | 2,33 | 2,6 | 2 | 3,63 |
| 2493 | sp|Q8WUM0|NU133_HUMAN | HUMAN | Nuclear pore complex protein Nup133 OS=Homo sapiens GN=NUP133 PE=1 SV=2 | 2,33 | 2,55 | 1 | 1,384 |
| 2494 | sp|Q96CM8|ACSF2_HUMAN | HUMAN | Acyl-CoA synthetase family member 2, mitochondrial OS=Homo sapiens GN=ACSF2 PE=1 SV=2 | 2,33 | 2,47 | 3 | 6,179 |
| 2495 | sp|P23258|TBG1_HUMAN | HUMAN | Tubulin gamma-1 chain OS=Homo sapiens GN=TUBG1 PE=1 SV=2 | 2,33 | 2,42 | 2 | 3,769 |
| 2496 | sp|Q96EL3|RM53_HUMAN | HUMAN | 39S ribosomal protein L53, mitochondrial OS=Homo sapiens GN=MRPL53 PE=1 SV=1 | 2,33 | 2,36 | 1 | 11,61 |
| 2497 | sp|Q9BV86|NTM1A_HUMAN | HUMAN | N-terminal Xaa-Pro-Lys N-methyltransferase 1 OS=Homo sapiens GN=NTMT1 PE=1 SV=3 | 2,33 | 2,35 | 1 | 6,726 |
| 2498 | sp|P04440|DPB1_HUMAN | HUMAN | HLA class II histocompatibility antigen, DP beta 1 chain OS=Homo sapiens GN=HLA-DPB1 PE=1 SV=1 | 2,33 | 2,34 | 1 | 3,488 |
| 2499 | sp|Q9NUG6|PDRG1_HUMAN | HUMAN | p53 and DNA damage-regulated protein 1 OS=Homo sapiens GN=PDRG1 PE=1 SV=2 | 2,32 | 2,45 | 2 | 22,56 |
| 2500 | sp|Q9UI12|VATH_HUMAN | HUMAN | V-type proton ATPase subunit H OS=Homo sapiens GN=ATP6V1H PE=1 SV=1 | 2,32 | 2,44 | 2 | 8,696 |
| 2501 | sp|Q9P016|THYN1_HUMAN | HUMAN | Thymocyte nuclear protein 1 OS=Homo sapiens GN=THYN1 PE=1 SV=1 | 2,32 | 2,41 | 1 | 3,111 |
| 2502 | sp|Q15813|TBCE_HUMAN | HUMAN | Tubulin-specific chaperone E OS=Homo sapiens GN=TBCE PE=1 SV=1 | 2,32 | 2,38 | 1 | 4,175 |
| 2503 | sp|Q9GZN8|CT027_HUMAN | HUMAN | UPF0687 protein C20orf27 OS=Homo sapiens GN=C20orf27 PE=1 SV=3 | 2,32 | 2,34 | 1 | 8,046 |
| 2504 | sp|Q9UBW5|BIN2_HUMAN | HUMAN | Bridging integrator 2 OS=Homo sapiens GN=BIN2 PE=1 SV=3 | 2,31 | 3,71 | 3 | 6,549 |
| 2505 | sp|P11532|DMD_HUMAN | HUMAN | Dystrophin OS=Homo sapiens GN=DMD PE=1 SV=3 | 2,31 | 2,54 | 1 | 0,3256 |
| 2506 | sp|Q96KC8|DNJC1_HUMAN | HUMAN | DnaJ homolog subfamily C member 1 OS=Homo sapiens GN=DNAJC1 PE=1 SV=1 | 2,31 | 2,45 | 2 | 4,332 |
| 2507 | sp|Q9NRY5|F1142_HUMAN | HUMAN | Protein FAM114A2 OS=Homo sapiens GN=FAM114A2 PE=1 SV=4 | 2,31 | 2,38 | 2 | 7,525 |
| 2508 | sp|O43660|PLRG1_HUMAN | HUMAN | Pleiotropic regulator 1 OS=Homo sapiens GN=PLRG1 PE=1 SV=1 | 2,31 | 2,33 | 1 | 5,253 |
| 2509 | sp|P41219|PERI_HUMAN | HUMAN | Peripherin OS=Homo sapiens GN=PRPH PE=1 SV=2 | 2,3 | 10,59 | 11 | 8,511 |
| 2510 | sp|Q86SX6|GLRX5_HUMAN | HUMAN | Glutaredoxin-related protein 5, mitochondrial OS=Homo sapiens GN=GLRX5 PE=1 SV=2 | 2,3 | 2,34 | 2 | 21,02 |
| 2511 | sp|O15127|SCAM2_HUMAN | HUMAN | Secretory carrier-associated membrane protein 2 OS=Homo sapiens GN=SCAMP2 PE=1 SV=2 | 2,3 | 2,32 | 1 | 6,079 |
| 2512 | sp|A1X283|SPD2B_HUMAN | HUMAN | SH3 and PX domain-containing protein 2B OS=Homo sapiens GN=SH3PXD2B PE=1 SV=3 | 2,29 | 2,57 | 1 | 1,427 |
| 2513 | sp|Q9Y3C4|TPRKB_HUMAN | HUMAN | EKC/KEOPS complex subunit TPRKB OS=Homo sapiens GN=TPRKB PE=1 SV=1 | 2,29 | 2,35 | 2 | 10,86 |
| 2514 | sp|O00186|STXB3_HUMAN | HUMAN | Syntaxin-binding protein 3 OS=Homo sapiens GN=STXBP3 PE=1 SV=2 | 2,29 | 2,33 | 1 | 1,52 |
| 2515 | sp|Q12849|GRSF1_HUMAN | HUMAN | G-rich sequence factor 1 OS=Homo sapiens GN=GRSF1 PE=1 SV=3 | 2,28 | 2,62 | 3 | 3,958 |
| 2516 | sp|Q8IW45|NNRD_HUMAN | HUMAN | ATP-dependent (S)-NAD(P)H-hydrate dehydratase OS=Homo sapiens GN=CARKD PE=1 SV=1 | 2,28 | 2,35 | 1 | 5,476 |
| 2517 | sp|Q9UJY5|GGA1_HUMAN | HUMAN | ADP-ribosylation factor-binding protein GGA1 OS=Homo sapiens GN=GGA1 PE=1 SV=1 | 2,28 | 2,31 | 1 | 5,008 |
| 2518 | sp|Q92615|LAR4B_HUMAN | HUMAN | La-related protein 4B OS=Homo sapiens GN=LARP4B PE=1 SV=3 | 2,28 | 2,3 | 1 | 1,491 |
| 2519 | sp|P46977|STT3A_HUMAN | HUMAN | Dolichyl-diphosphooligosaccharide--protein glycosyltransferase subunit STT3A OS=Homo sapiens GN=STT3A PE=1 SV=2 | 2,27 | 2,59 | 1 | 0,9929 |
| 2520 | sp|P49441|INPP_HUMAN | HUMAN | Inositol polyphosphate 1-phosphatase OS=Homo sapiens GN=INPP1 PE=1 SV=1 | 2,27 | 2,39 | 1 | 3,008 |
| 2521 | sp|Q8IXM3|RM41_HUMAN | HUMAN | 39S ribosomal protein L41, mitochondrial OS=Homo sapiens GN=MRPL41 PE=1 SV=1 | 2,27 | 2,34 | 2 | 16,06 |
| 2522 | sp|P61803|DAD1_HUMAN | HUMAN | Dolichyl-diphosphooligosaccharide--protein glycosyltransferase subunit DAD1 OS=Homo sapiens GN=DAD1 PE=1 SV=3 | 2,27 | 2,29 | 1 | 10,62 |
| 2523 | sp|Q3MHD2|LSM12_HUMAN | HUMAN | Protein LSM12 homolog OS=Homo sapiens GN=LSM12 PE=1 SV=2 | 2,26 | 2,36 | 2 | 13,85 |
| 2524 | sp|Q8NEZ5|FBX22_HUMAN | HUMAN | F-box only protein 22 OS=Homo sapiens GN=FBXO22 PE=1 SV=1 | 2,25 | 2,27 | 1 | 2,481 |
| 2525 | sp|P78346|RPP30_HUMAN | HUMAN | Ribonuclease P protein subunit p30 OS=Homo sapiens GN=RPP30 PE=1 SV=1 | 2,25 | 2,27 | 2 | 7,09 |
| 2526 | sp|P11171|41_HUMAN | HUMAN | Protein 4.1 OS=Homo sapiens GN=EPB41 PE=1 SV=4 | 2,24 | 4,27 | 3 | 4,514 |
| 2527 | sp|P60903|S10AA_HUMAN | HUMAN | Protein S100-A10 OS=Homo sapiens GN=S100A10 PE=1 SV=2 | 2,24 | 2,38 | 2 | 17,53 |
| 2528 | sp|P49757|NUMB_HUMAN | HUMAN | Protein numb homolog OS=Homo sapiens GN=NUMB PE=1 SV=2 | 2,24 | 2,28 | 1 | 3,687 |
| 2529 | sp|O95758|PTBP3_HUMAN | HUMAN | Polypyrimidine tract-binding protein 3 OS=Homo sapiens GN=PTBP3 PE=1 SV=2 | 2,23 | 9,47 | 6 | 15,94 |
| 2530 | sp|P51159|RB27A_HUMAN | HUMAN | Ras-related protein Rab-27A OS=Homo sapiens GN=RAB27A PE=1 SV=3 | 2,23 | 4,55 | 7 | 17,65 |
| 2531 | sp|Q8N573|OXR1_HUMAN | HUMAN | Oxidation resistance protein 1 OS=Homo sapiens GN=OXR1 PE=1 SV=2 | 2,23 | 3,06 | 2 | 1,945 |
| 2532 | sp|Q15654|TRIP6_HUMAN | HUMAN | Thyroid receptor-interacting protein 6 OS=Homo sapiens GN=TRIP6 PE=1 SV=3 | 2,23 | 2,3 | 2 | 8,193 |
| 2533 | sp|Q13287|NMI_HUMAN | HUMAN | N-myc-interactor OS=Homo sapiens GN=NMI PE=1 SV=2 | 2,23 | 2,26 | 1 | 3,257 |
| 2534 | sp|O15533|TPSN_HUMAN | HUMAN | Tapasin OS=Homo sapiens GN=TAPBP PE=1 SV=1 | 2,23 | 2,25 | 1 | 2,902 |
| 2535 | sp|Q9Y6A4|CFA20_HUMAN | HUMAN | Cilia- and flagella-associated protein 20 OS=Homo sapiens GN=CFAP20 PE=1 SV=1 | 2,22 | 2,24 | 1 | 4,663 |
| 2536 | sp|Q9NUL5|RYDEN_HUMAN | HUMAN | Repressor of yield of DENV protein OS=Homo sapiens GN=RYDEN PE=1 SV=2 | 2,22 | 2,24 | 1 | 6,186 |
| 2537 | sp|O95379|TFIP8_HUMAN | HUMAN | Tumor necrosis factor alpha-induced protein 8 OS=Homo sapiens GN=TNFAIP8 PE=1 SV=1 | 2,22 | 2,24 | 1 | 10,1 |
| 2538 | sp|O00461|GOLI4_HUMAN | HUMAN | Golgi integral membrane protein 4 OS=Homo sapiens GN=GOLIM4 PE=1 SV=1 | 2,22 | 2,23 | 1 | 1,58 |
| 2539 | sp|Q9C0E2|XPO4_HUMAN | HUMAN | Exportin-4 OS=Homo sapiens GN=XPO4 PE=1 SV=2 | 2,21 | 2,48 | 1 | 1,477 |
| 2540 | sp|Q9H307|PININ_HUMAN | HUMAN | Pinin OS=Homo sapiens GN=PNN PE=1 SV=4 | 2,21 | 2,31 | 1 | 2,371 |
| 2541 | sp|Q03169|TNAP2_HUMAN | HUMAN | Tumor necrosis factor alpha-induced protein 2 OS=Homo sapiens GN=TNFAIP2 PE=2 SV=2 | 2,21 | 2,23 | 2 | 3,517 |
| 2542 | sp|Q96GD0|PLPP_HUMAN | HUMAN | Pyridoxal phosphate phosphatase OS=Homo sapiens GN=PDXP PE=1 SV=2 | 2,21 | 2,22 | 1 | 4,73 |
| 2543 | sp|P48426|PI42A_HUMAN | HUMAN | Phosphatidylinositol 5-phosphate 4-kinase type-2 alpha OS=Homo sapiens GN=PIP4K2A PE=1 SV=2 | 2,2 | 6,14 | 6 | 14,78 |
| 2544 | sp|O15258|RER1_HUMAN | HUMAN | Protein RER1 OS=Homo sapiens GN=RER1 PE=1 SV=1 | 2,2 | 2,25 | 3 | 21,43 |
| 2545 | sp|Q8TD19|NEK9_HUMAN | HUMAN | Serine/threonine-protein kinase Nek9 OS=Homo sapiens GN=NEK9 PE=1 SV=2 | 2,2 | 2,24 | 1 | 1,328 |
| 2546 | sp|P20338|RAB4A_HUMAN | HUMAN | Ras-related protein Rab-4A OS=Homo sapiens GN=RAB4A PE=1 SV=3 | 2,19 | 4,5 | 6 | 17,43 |
| 2547 | sp|Q9Y4X5|ARI1_HUMAN | HUMAN | E3 ubiquitin-protein ligase ARIH1 OS=Homo sapiens GN=ARIH1 PE=1 SV=2 | 2,19 | 2,28 | 2 | 7,361 |
| 2548 | sp|P40123|CAP2_HUMAN | HUMAN | Adenylyl cyclase-associated protein 2 OS=Homo sapiens GN=CAP2 PE=1 SV=1 | 2,18 | 6,21 | 3 | 5,031 |
| 2549 | sp|P37235|HPCL1_HUMAN | HUMAN | Hippocalcin-like protein 1 OS=Homo sapiens GN=HPCAL1 PE=1 SV=3 | 2,18 | 2,37 | 2 | 7,772 |
| 2550 | sp|Q2TAY7|SMU1_HUMAN | HUMAN | WD40 repeat-containing protein SMU1 OS=Homo sapiens GN=SMU1 PE=1 SV=2 | 2,18 | 2,25 | 1 | 1,949 |
| 2551 | sp|O95573|ACSL3_HUMAN | HUMAN | Long-chain-fatty-acid--CoA ligase 3 OS=Homo sapiens GN=ACSL3 PE=1 SV=3 | 2,18 | 2,24 | 1 | 2,5 |
| 2552 | sp|P05362|ICAM1_HUMAN | HUMAN | Intercellular adhesion molecule 1 OS=Homo sapiens GN=ICAM1 PE=1 SV=2 | 2,18 | 2,2 | 1 | 2,632 |
| 2553 | sp|P48163|MAOX_HUMAN | HUMAN | NADP-dependent malic enzyme OS=Homo sapiens GN=ME1 PE=1 SV=1 | 2,17 | 2,24 | 1 | 1,923 |
| 2554 | sp|Q8IXQ4|GPAM1_HUMAN | HUMAN | GPALPP motifs-containing protein 1 OS=Homo sapiens GN=GPALPP1 PE=1 SV=1 | 2,17 | 2,19 | 1 | 3,824 |
| 2555 | sp|P80365|DHI2_HUMAN | HUMAN | Corticosteroid 11-beta-dehydrogenase isozyme 2 OS=Homo sapiens GN=HSD11B2 PE=1 SV=2 | 2,16 | 2,56 | 2 | 5,185 |
| 2556 | sp|Q5T1M5|FKB15_HUMAN | HUMAN | FK506-binding protein 15 OS=Homo sapiens GN=FKBP15 PE=1 SV=2 | 2,16 | 2,2 | 1 | 1,148 |
| 2557 | sp|Q86VM9|ZCH18_HUMAN | HUMAN | Zinc finger CCCH domain-containing protein 18 OS=Homo sapiens GN=ZC3H18 PE=1 SV=2 | 2,16 | 2,18 | 1 | 1,259 |
| 2558 | sp|Q93062|RBPMS_HUMAN | HUMAN | RNA-binding protein with multiple splicing OS=Homo sapiens GN=RBPMS PE=1 SV=1 | 2,16 | 2,18 | 1 | 7,143 |
| 2559 | sp|Q6WKZ4|RFIP1_HUMAN | HUMAN | Rab11 family-interacting protein 1 OS=Homo sapiens GN=RAB11FIP1 PE=1 SV=3 | 2,15 | 2,25 | 3 | 2,572 |
| 2560 | sp|Q9UKF6|CPSF3_HUMAN | HUMAN | Cleavage and polyadenylation specificity factor subunit 3 OS=Homo sapiens GN=CPSF3 PE=1 SV=1 | 2,14 | 2,24 | 2 | 2,924 |
| 2561 | sp|P34096|RNAS4_HUMAN | HUMAN | Ribonuclease 4 OS=Homo sapiens GN=RNASE4 PE=1 SV=3 | 2,14 | 2,22 | 1 | 7,483 |
| 2562 | sp|Q9Y237|PIN4_HUMAN | HUMAN | Peptidyl-prolyl cis-trans isomerase NIMA-interacting 4 OS=Homo sapiens GN=PIN4 PE=1 SV=1 | 2,14 | 2,19 | 1 | 9,16 |
| 2563 | sp|P24941|CDK2_HUMAN | HUMAN | Cyclin-dependent kinase 2 OS=Homo sapiens GN=CDK2 PE=1 SV=2 | 2,13 | 3,6 | 2 | 7,383 |
| 2564 | sp|Q15080|NCF4_HUMAN | HUMAN | Neutrophil cytosol factor 4 OS=Homo sapiens GN=NCF4 PE=1 SV=2 | 2,13 | 2,44 | 1 | 2,95 |
| 2565 | sp|P40818|UBP8_HUMAN | HUMAN | Ubiquitin carboxyl-terminal hydrolase 8 OS=Homo sapiens GN=USP8 PE=1 SV=1 | 2,13 | 2,19 | 1 | 1,968 |
| 2566 | sp|P49770|EI2BB_HUMAN | HUMAN | Translation initiation factor eIF-2B subunit beta OS=Homo sapiens GN=EIF2B2 PE=1 SV=3 | 2,13 | 2,15 | 1 | 5,128 |
| 2567 | sp|Q7L2J0|MEPCE_HUMAN | HUMAN | 7SK snRNA methylphosphate capping enzyme OS=Homo sapiens GN=MEPCE PE=1 SV=1 | 2,13 | 2,14 | 1 | 1,597 |
| 2568 | sp|P04233|HG2A_HUMAN | HUMAN | HLA class II histocompatibility antigen gamma chain OS=Homo sapiens GN=CD74 PE=1 SV=3 | 2,13 | 2,14 | 1 | 4,73 |
| 2569 | sp|P01920|DQB1_HUMAN | HUMAN | HLA class II histocompatibility antigen, DQ beta 1 chain OS=Homo sapiens GN=HLA-DQB1 PE=1 SV=2 | 2,13 | 2,14 | 1 | 4,598 |
| 2570 | sp|Q9C002|NMES1_HUMAN | HUMAN | Normal mucosa of esophagus-specific gene 1 protein OS=Homo sapiens GN=NMES1 PE=2 SV=1 | 2,13 | 2,14 | 1 | 22,89 |
| 2571 | sp|Q01523|DEF5_HUMAN | HUMAN | Defensin-5 OS=Homo sapiens GN=DEFA5 PE=1 SV=1 | 2,13 | 2,14 | 2 | 36,17 |
| 2572 | sp|Q13620|CUL4B_HUMAN | HUMAN | Cullin-4B OS=Homo sapiens GN=CUL4B PE=1 SV=4 | 2,12 | 10,4 | 5 | 5,915 |
| 2573 | sp|Q9P013|CWC15_HUMAN | HUMAN | Spliceosome-associated protein CWC15 homolog OS=Homo sapiens GN=CWC15 PE=1 SV=2 | 2,12 | 2,14 | 1 | 4,803 |
| 2574 | sp|P85037|FOXK1_HUMAN | HUMAN | Forkhead box protein K1 OS=Homo sapiens GN=FOXK1 PE=1 SV=1 | 2,12 | 2,14 | 1 | 4,911 |
| 2575 | sp|Q9BZH6|WDR11_HUMAN | HUMAN | WD repeat-containing protein 11 OS=Homo sapiens GN=WDR11 PE=1 SV=1 | 2,11 | 2,23 | 2 | 2,042 |
| 2576 | sp|Q4G0N4|NAKD2_HUMAN | HUMAN | NAD kinase 2, mitochondrial OS=Homo sapiens GN=NADK2 PE=1 SV=2 | 2,1 | 2,2 | 2 | 6,109 |
| 2577 | sp|Q8NFH3|NUP43_HUMAN | HUMAN | Nucleoporin Nup43 OS=Homo sapiens GN=NUP43 PE=1 SV=1 | 2,1 | 2,19 | 2 | 8,158 |
| 2578 | sp|P08253|MMP2_HUMAN | HUMAN | 72 kDa type IV collagenase OS=Homo sapiens GN=MMP2 PE=1 SV=2 | 2,1 | 2,18 | 2 | 5,758 |
| 2579 | sp|O75122|CLAP2_HUMAN | HUMAN | CLIP-associating protein 2 OS=Homo sapiens GN=CLASP2 PE=1 SV=2 | 2,1 | 2,14 | 1 | 1,468 |
| 2580 | sp|O95295|SNAPN_HUMAN | HUMAN | SNARE-associated protein Snapin OS=Homo sapiens GN=SNAPIN PE=1 SV=1 | 2,1 | 2,12 | 1 | 8,824 |
| 2581 | sp|P14927|QCR7_HUMAN | HUMAN | Cytochrome b-c1 complex subunit 7 OS=Homo sapiens GN=UQCRB PE=1 SV=2 | 2,1 | 2,12 | 1 | 11,71 |
| 2582 | sp|Q9Y3B3|TMED7_HUMAN | HUMAN | Transmembrane emp24 domain-containing protein 7 OS=Homo sapiens GN=TMED7 PE=1 SV=2 | 2,1 | 2,11 | 1 | 5,804 |
| 2583 | sp|P98095|FBLN2_HUMAN | HUMAN | Fibulin-2 OS=Homo sapiens GN=FBLN2 PE=1 SV=2 | 2,1 | 2,1 | 1 | 1,52 |
| 2584 | sp|P42574|CASP3_HUMAN | HUMAN | Caspase-3 OS=Homo sapiens GN=CASP3 PE=1 SV=2 | 2,09 | 4,13 | 3 | 10,83 |
| 2585 | sp|P04433|KV309_HUMAN | HUMAN | Ig kappa chain V-III region VG (Fragment) OS=Homo sapiens PE=1 SV=1 | 2,09 | 2,46 | 2 | 23,48 |
| 2586 | sp|Q9UBB5|MBD2_HUMAN | HUMAN | Methyl-CpG-binding domain protein 2 OS=Homo sapiens GN=MBD2 PE=1 SV=1 | 2,09 | 2,16 | 1 | 2,433 |
| 2587 | sp|O95466|FMNL1_HUMAN | HUMAN | Formin-like protein 1 OS=Homo sapiens GN=FMNL1 PE=1 SV=3 | 2,09 | 2,11 | 1 | 1,182 |
| 2588 | sp|Q96EY8|MMAB_HUMAN | HUMAN | Cob(I)yrinic acid a,c-diamide adenosyltransferase, mitochondrial OS=Homo sapiens GN=MMAB PE=1 SV=1 | 2,09 | 2,1 | 1 | 4 |
| 2589 | sp|Q8TCS8|PNPT1_HUMAN | HUMAN | Polyribonucleotide nucleotidyltransferase 1, mitochondrial OS=Homo sapiens GN=PNPT1 PE=1 SV=2 | 2,08 | 2,28 | 2 | 1,916 |
| 2590 | sp|O43148|MCES_HUMAN | HUMAN | mRNA cap guanine-N7 methyltransferase OS=Homo sapiens GN=RNMT PE=1 SV=1 | 2,08 | 2,14 | 1 | 2,521 |
| 2591 | sp|P02741|CRP_HUMAN | HUMAN | C-reactive protein OS=Homo sapiens GN=CRP PE=1 SV=1 | 2,08 | 2,13 | 1 | 4,464 |
| 2592 | sp|O95400|CD2B2_HUMAN | HUMAN | CD2 antigen cytoplasmic tail-binding protein 2 OS=Homo sapiens GN=CD2BP2 PE=1 SV=1 | 2,08 | 2,1 | 1 | 4,106 |
| 2593 | sp|Q6NUM9|RETST_HUMAN | HUMAN | All-trans-retinol 13,14-reductase OS=Homo sapiens GN=RETSAT PE=1 SV=2 | 2,08 | 2,09 | 1 | 1,967 |
| 2594 | sp|Q9BXV9|CN142_HUMAN | HUMAN | Uncharacterized protein C14orf142 OS=Homo sapiens GN=C14orf142 PE=1 SV=2 | 2,08 | 2,08 | 2 | 34 |
| 2595 | sp|P62873|GBB1_HUMAN | HUMAN | Guanine nucleotide-binding protein G(I)/G(S)/G(T) subunit beta-1 OS=Homo sapiens GN=GNB1 PE=1 SV=3 | 2,07 | 12,65 | 7 | 27,94 |
| 2596 | sp|Q8TDB6|DTX3L_HUMAN | HUMAN | E3 ubiquitin-protein ligase DTX3L OS=Homo sapiens GN=DTX3L PE=1 SV=1 | 2,07 | 2,23 | 1 | 2,027 |
| 2597 | sp|P04430|KV122_HUMAN | HUMAN | Ig kappa chain V-I region BAN OS=Homo sapiens PE=1 SV=1 | 2,07 | 2,22 | 4 | 23,15 |
| 2598 | sp|P78330|SERB_HUMAN | HUMAN | Phosphoserine phosphatase OS=Homo sapiens GN=PSPH PE=1 SV=2 | 2,07 | 2,16 | 2 | 9,778 |
| 2599 | sp|Q9UPN6|SCAF8_HUMAN | HUMAN | Protein SCAF8 OS=Homo sapiens GN=SCAF8 PE=1 SV=1 | 2,07 | 2,14 | 1 | 0,9441 |
| 2600 | sp|Q4G0F5|VP26B_HUMAN | HUMAN | Vacuolar protein sorting-associated protein 26B OS=Homo sapiens GN=VPS26B PE=1 SV=2 | 2,07 | 2,1 | 1 | 3,274 |
| 2601 | sp|Q8IVM0|CCD50_HUMAN | HUMAN | Coiled-coil domain-containing protein 50 OS=Homo sapiens GN=CCDC50 PE=1 SV=1 | 2,07 | 2,09 | 2 | 3,922 |
| 2602 | sp|P36639|8ODP_HUMAN | HUMAN | 7,8-dihydro-8-oxoguanine triphosphatase OS=Homo sapiens GN=NUDT1 PE=1 SV=3 | 2,07 | 2,09 | 1 | 6,091 |
| 2603 | sp|P40855|PEX19_HUMAN | HUMAN | Peroxisomal biogenesis factor 19 OS=Homo sapiens GN=PEX19 PE=1 SV=1 | 2,07 | 2,08 | 1 | 7,023 |
| 2604 | sp|Q99895|CTRC_HUMAN | HUMAN | Chymotrypsin-C OS=Homo sapiens GN=CTRC PE=1 SV=2 | 2,07 | 2,08 | 1 | 5,97 |
| 2605 | sp|O15260|SURF4_HUMAN | HUMAN | Surfeit locus protein 4 OS=Homo sapiens GN=SURF4 PE=1 SV=3 | 2,07 | 2,08 | 1 | 4,833 |
| 2606 | sp|P22033|MUTA_HUMAN | HUMAN | Methylmalonyl-CoA mutase, mitochondrial OS=Homo sapiens GN=MUT PE=1 SV=4 | 2,07 | 2,07 | 1 | 1,467 |
| 2607 | sp|Q9NWH9|SLTM_HUMAN | HUMAN | SAFB-like transcription modulator OS=Homo sapiens GN=SLTM PE=1 SV=2 | 2,06 | 2,26 | 2 | 2,998 |
| 2608 | sp|Q99797|MIPEP_HUMAN | HUMAN | Mitochondrial intermediate peptidase OS=Homo sapiens GN=MIPEP PE=1 SV=2 | 2,06 | 2,07 | 1 | 1,403 |
| 2609 | sp|Q9NPA8|ENY2_HUMAN | HUMAN | Transcription and mRNA export factor ENY2 OS=Homo sapiens GN=ENY2 PE=1 SV=1 | 2,06 | 2,07 | 1 | 16,83 |
| 2610 | sp|P07738|PMGE_HUMAN | HUMAN | Bisphosphoglycerate mutase OS=Homo sapiens GN=BPGM PE=1 SV=2 | 2,06 | 2,07 | 1 | 7,336 |
| 2611 | sp|O43709|WBS22_HUMAN | HUMAN | Probable 18S rRNA (guanine-N(7))-methyltransferase OS=Homo sapiens GN=WBSCR22 PE=1 SV=2 | 2,06 | 2,07 | 1 | 7,829 |
| 2612 | sp|O95208|EPN2_HUMAN | HUMAN | Epsin-2 OS=Homo sapiens GN=EPN2 PE=1 SV=3 | 2,06 | 2,06 | 1 | 1,56 |
| 2613 | sp|P22694|KAPCB_HUMAN | HUMAN | cAMP-dependent protein kinase catalytic subunit beta OS=Homo sapiens GN=PRKACB PE=1 SV=2 | 2,05 | 7,44 | 6 | 19,37 |
| 2614 | sp|P55210|CASP7_HUMAN | HUMAN | Caspase-7 OS=Homo sapiens GN=CASP7 PE=1 SV=1 | 2,05 | 2,11 | 1 | 2,64 |
| 2615 | sp|Q13427|PPIG_HUMAN | HUMAN | Peptidyl-prolyl cis-trans isomerase G OS=Homo sapiens GN=PPIG PE=1 SV=2 | 2,05 | 2,08 | 1 | 1,592 |
| 2616 | sp|O95486|SC24A_HUMAN | HUMAN | Protein transport protein Sec24A OS=Homo sapiens GN=SEC24A PE=1 SV=2 | 2,05 | 2,07 | 1 | 1,372 |
| 2617 | sp|P42768|WASP_HUMAN | HUMAN | Wiskott-Aldrich syndrome protein OS=Homo sapiens GN=WAS PE=1 SV=4 | 2,05 | 2,07 | 1 | 2,988 |
| 2618 | sp|P60709|ACTB_HUMAN | HUMAN | Actin, cytoplasmic 1 OS=Homo sapiens GN=ACTB PE=1 SV=1 | 2,04 | 77,88 | 357 | 80,27 |
| 2619 | sp|O95292|VAPB_HUMAN | HUMAN | Vesicle-associated membrane protein-associated protein B/C OS=Homo sapiens GN=VAPB PE=1 SV=3 | 2,04 | 4,12 | 2 | 10,7 |
| 2620 | sp|P06732|KCRM_HUMAN | HUMAN | Creatine kinase M-type OS=Homo sapiens GN=CKM PE=1 SV=2 | 2,04 | 3,3 | 9 | 15,22 |
| 2621 | sp|P28715|ERCC5_HUMAN | HUMAN | DNA repair protein complementing XP-G cells OS=Homo sapiens GN=ERCC5 PE=1 SV=3 | 2,04 | 2,06 | 1 | 1,265 |
| 2622 | sp|Q8NBN7|RDH13_HUMAN | HUMAN | Retinol dehydrogenase 13 OS=Homo sapiens GN=RDH13 PE=1 SV=2 | 2,04 | 2,06 | 1 | 7,251 |
| 2623 | sp|O00178|GTPB1_HUMAN | HUMAN | GTP-binding protein 1 OS=Homo sapiens GN=GTPBP1 PE=1 SV=3 | 2,04 | 2,04 | 1 | 2,392 |
| 2624 | sp|Q8IYS1|P20D2_HUMAN | HUMAN | Peptidase M20 domain-containing protein 2 OS=Homo sapiens GN=PM20D2 PE=1 SV=2 | 2,04 | 2,04 | 1 | 3,44 |
| 2625 | sp|P01613|KV121_HUMAN | HUMAN | Ig kappa chain V-I region Ni OS=Homo sapiens PE=1 SV=1 | 2,04 | 2,04 | 1 | 14,29 |
| 2626 | sp|Q15349|KS6A2_HUMAN | HUMAN | Ribosomal protein S6 kinase alpha-2 OS=Homo sapiens GN=RPS6KA2 PE=1 SV=2 | 2,03 | 11,27 | 6 | 9,413 |
| 2627 | sp|O95219|SNX4_HUMAN | HUMAN | Sorting nexin-4 OS=Homo sapiens GN=SNX4 PE=1 SV=1 | 2,03 | 2,09 | 1 | 2 |
| 2628 | sp|P15428|PGDH_HUMAN | HUMAN | 15-hydroxyprostaglandin dehydrogenase [NAD(+)] OS=Homo sapiens GN=HPGD PE=1 SV=1 | 2,03 | 2,08 | 1 | 4,887 |
| 2629 | sp|Q6T4P5|PLPR3_HUMAN | HUMAN | Phospholipid phosphatase-related protein type 3 OS=Homo sapiens GN=PLPPR3 PE=2 SV=1 | 2,03 | 2,06 | 1 | 1,253 |
| 2630 | sp|Q9NX63|MIC19_HUMAN | HUMAN | MICOS complex subunit MIC19 OS=Homo sapiens GN=CHCHD3 PE=1 SV=1 | 2,03 | 2,05 | 2 | 7,048 |
| 2631 | sp|Q9Y6C9|MTCH2_HUMAN | HUMAN | Mitochondrial carrier homolog 2 OS=Homo sapiens GN=MTCH2 PE=1 SV=1 | 2,03 | 2,05 | 1 | 4,29 |
| 2632 | sp|Q96GE6|CALL4_HUMAN | HUMAN | Calmodulin-like protein 4 OS=Homo sapiens GN=CALML4 PE=2 SV=3 | 2,03 | 2,04 | 1 | 6,633 |
| 2633 | sp|Q8N2S1|LTBP4_HUMAN | HUMAN | Latent-transforming growth factor beta-binding protein 4 OS=Homo sapiens GN=LTBP4 PE=1 SV=2 | 2,03 | 2,04 | 1 | 0,8621 |
| 2634 | sp|P51608|MECP2_HUMAN | HUMAN | Methyl-CpG-binding protein 2 OS=Homo sapiens GN=MECP2 PE=1 SV=1 | 2,03 | 2,03 | 1 | 2,881 |
| 2635 | sp|Q96EE3|SEH1_HUMAN | HUMAN | Nucleoporin SEH1 OS=Homo sapiens GN=SEH1L PE=1 SV=3 | 2,03 | 2,03 | 1 | 5,278 |
| 2636 | sp|Q15363|TMED2_HUMAN | HUMAN | Transmembrane emp24 domain-containing protein 2 OS=Homo sapiens GN=TMED2 PE=1 SV=1 | 2,03 | 2,03 | 1 | 4,478 |
| 2637 | sp|Q01658|NC2B_HUMAN | HUMAN | Protein Dr1 OS=Homo sapiens GN=DR1 PE=1 SV=1 | 2,03 | 2,03 | 1 | 7,386 |
| 2638 | sp|Q969T9|WBP2_HUMAN | HUMAN | WW domain-binding protein 2 OS=Homo sapiens GN=WBP2 PE=1 SV=1 | 2,03 | 2,03 | 1 | 3,831 |
| 2639 | sp|P0CG39|POTEJ_HUMAN | HUMAN | POTE ankyrin domain family member J OS=Homo sapiens GN=POTEJ PE=3 SV=1 | 2,02 | 13,37 | 46 | 12,52 |
| 2640 | sp|P01621|KV303_HUMAN | HUMAN | Ig kappa chain V-III region NG9 (Fragment) OS=Homo sapiens PE=2 SV=1 | 2,02 | 13,01 | 11 | 73 |
| 2641 | sp|Q5JWF2|GNAS1_HUMAN | HUMAN | Guanine nucleotide-binding protein G(s) subunit alpha isoforms XLas OS=Homo sapiens GN=GNAS PE=1 SV=2 | 2,02 | 4,16 | 3 | 3,086 |
| 2642 | sp|Q8NF91|SYNE1_HUMAN | HUMAN | Nesprin-1 OS=Homo sapiens GN=SYNE1 PE=1 SV=4 | 2,02 | 3,79 | 1 | 0,1364 |
| 2643 | sp|Q9UL45|BL1S6_HUMAN | HUMAN | Biogenesis of lysosome-related organelles complex 1 subunit 6 OS=Homo sapiens GN=BLOC1S6 PE=1 SV=1 | 2,02 | 2,35 | 2 | 11,05 |
| 2644 | sp|P55083|MFAP4_HUMAN | HUMAN | Microfibril-associated glycoprotein 4 OS=Homo sapiens GN=MFAP4 PE=1 SV=2 | 2,02 | 2,33 | 1 | 7,059 |
| 2645 | sp|P54687|BCAT1_HUMAN | HUMAN | Branched-chain-amino-acid aminotransferase, cytosolic OS=Homo sapiens GN=BCAT1 PE=1 SV=3 | 2,02 | 2,2 | 2 | 5,181 |
| 2646 | sp|O00757|F16P2_HUMAN | HUMAN | Fructose-1,6-bisphosphatase isozyme 2 OS=Homo sapiens GN=FBP2 PE=1 SV=2 | 2,02 | 2,1 | 1 | 2,95 |
| 2647 | sp|Q92544|TM9S4_HUMAN | HUMAN | Transmembrane 9 superfamily member 4 OS=Homo sapiens GN=TM9SF4 PE=1 SV=2 | 2,02 | 2,09 | 1 | 2,96 |
| 2648 | sp|O60524|NEMF_HUMAN | HUMAN | Nuclear export mediator factor NEMF OS=Homo sapiens GN=NEMF PE=1 SV=4 | 2,02 | 2,08 | 1 | 1,115 |
| 2649 | sp|Q9ULR0|ISY1_HUMAN | HUMAN | Pre-mRNA-splicing factor ISY1 homolog OS=Homo sapiens GN=ISY1 PE=1 SV=3 | 2,02 | 2,06 | 1 | 6,667 |
| 2650 | sp|O75844|FACE1_HUMAN | HUMAN | CAAX prenyl protease 1 homolog OS=Homo sapiens GN=ZMPSTE24 PE=1 SV=2 | 2,02 | 2,05 | 1 | 2,316 |
| 2651 | sp|Q9P260|K1468_HUMAN | HUMAN | LisH domain and HEAT repeat-containing protein KIAA1468 OS=Homo sapiens GN=KIAA1468 PE=1 SV=2 | 2,02 | 2,03 | 1 | 0,9868 |
| 2652 | sp|Q9BZE4|NOG1_HUMAN | HUMAN | Nucleolar GTP-binding protein 1 OS=Homo sapiens GN=GTPBP4 PE=1 SV=3 | 2,02 | 2,03 | 1 | 3,943 |
| 2653 | sp|P42785|PCP_HUMAN | HUMAN | Lysosomal Pro-X carboxypeptidase OS=Homo sapiens GN=PRCP PE=1 SV=1 | 2,02 | 2,03 | 1 | 3,831 |
| 2654 | sp|Q5ZPR3|CD276_HUMAN | HUMAN | CD276 antigen OS=Homo sapiens GN=CD276 PE=1 SV=1 | 2,02 | 2,03 | 1 | 4,869 |
| 2655 | sp|Q9Y221|NIP7_HUMAN | HUMAN | 60S ribosome subunit biogenesis protein NIP7 homolog OS=Homo sapiens GN=NIP7 PE=1 SV=1 | 2,02 | 2,02 | 1 | 7,778 |
| 2656 | sp|Q96MM6|HS12B_HUMAN | HUMAN | Heat shock 70 kDa protein 12B OS=Homo sapiens GN=HSPA12B PE=1 SV=2 | 2,02 | 2,02 | 1 | 1,458 |
| 2657 | sp|O75208|COQ9_HUMAN | HUMAN | Ubiquinone biosynthesis protein COQ9, mitochondrial OS=Homo sapiens GN=COQ9 PE=1 SV=1 | 2,02 | 2,02 | 1 | 2,83 |
| 2658 | sp|Q8TEA8|DTD1_HUMAN | HUMAN | D-tyrosyl-tRNA(Tyr) deacylase 1 OS=Homo sapiens GN=DTD1 PE=1 SV=2 | 2,02 | 2,02 | 1 | 7,177 |
| 2659 | sp|Q12974|TP4A2_HUMAN | HUMAN | Protein tyrosine phosphatase type IVA 2 OS=Homo sapiens GN=PTP4A2 PE=1 SV=1 | 2,02 | 2,02 | 1 | 8,383 |
| 2660 | sp|Q9BV40|VAMP8_HUMAN | HUMAN | Vesicle-associated membrane protein 8 OS=Homo sapiens GN=VAMP8 PE=1 SV=1 | 2,02 | 2,02 | 2 | 10 |
| 2661 | sp|Q15041|AR6P1_HUMAN | HUMAN | ADP-ribosylation factor-like protein 6-interacting protein 1 OS=Homo sapiens GN=ARL6IP1 PE=1 SV=2 | 2,02 | 2,02 | 1 | 4,926 |
| 2662 | sp|Q13637|RAB32_HUMAN | HUMAN | Ras-related protein Rab-32 OS=Homo sapiens GN=RAB32 PE=1 SV=3 | 2,02 | 2,02 | 1 | 4,889 |
| 2663 | sp|P43251|BTD_HUMAN | HUMAN | Biotinidase OS=Homo sapiens GN=BTD PE=1 SV=2 | 2,02 | 2,02 | 1 | 2,394 |
| 2664 | sp|P83916|CBX1_HUMAN | HUMAN | Chromobox protein homolog 1 OS=Homo sapiens GN=CBX1 PE=1 SV=1 | 2,01 | 4,03 | 2 | 14,59 |
| 2665 | sp|P06493|CDK1_HUMAN | HUMAN | Cyclin-dependent kinase 1 OS=Homo sapiens GN=CDK1 PE=1 SV=3 | 2,01 | 3,44 | 2 | 6,061 |
| 2666 | sp|Q86Y46|K2C73_HUMAN | HUMAN | Keratin, type II cytoskeletal 73 OS=Homo sapiens GN=KRT73 PE=1 SV=1 | 2,01 | 3,29 | 3 | 3,333 |
| 2667 | sp|Q86W42|THOC6_HUMAN | HUMAN | THO complex subunit 6 homolog OS=Homo sapiens GN=THOC6 PE=1 SV=1 | 2,01 | 2,83 | 2 | 7,038 |
| 2668 | sp|Q8N668|COMD1_HUMAN | HUMAN | COMM domain-containing protein 1 OS=Homo sapiens GN=COMMD1 PE=1 SV=1 | 2,01 | 2,31 | 1 | 11,05 |
| 2669 | sp|Q13595|TRA2A_HUMAN | HUMAN | Transformer-2 protein homolog alpha OS=Homo sapiens GN=TRA2A PE=1 SV=1 | 2,01 | 2,27 | 1 | 4,965 |
| 2670 | RRRRRsp|Q8WWW0|RASF5_HUMAN ^(b)^ | HUMAN | REVERSED Ras association domain-containing protein 5 OS=Homo sapiens GN=RASSF5 PE=1 SV=1 | 2,01 | 2,11 | 1 | 2,392 |
| 2671 | sp|Q9BX40|LS14B_HUMAN | HUMAN | Protein LSM14 homolog B OS=Homo sapiens GN=LSM14B PE=1 SV=1 | 2,01 | 2,09 | 2 | 4,675 |
| 2672 | sp|P59768|GBG2_HUMAN | HUMAN | Guanine nucleotide-binding protein G(I)/G(S)/G(O) subunit gamma-2 OS=Homo sapiens GN=GNG2 PE=1 SV=2 | 2,01 | 2,08 | 1 | 22,54 |
| 2673 | sp|Q5VZ89|DEN4C_HUMAN | HUMAN | DENN domain-containing protein 4C OS=Homo sapiens GN=DENND4C PE=1 SV=2 | 2,01 | 2,04 | 1 | 0,7173 |
| 2674 | sp|Q9NWS0|PIHD1_HUMAN | HUMAN | PIH1 domain-containing protein 1 OS=Homo sapiens GN=PIH1D1 PE=1 SV=1 | 2,01 | 2,04 | 1 | 4,828 |
| 2675 | sp|Q13895|BYST_HUMAN | HUMAN | Bystin OS=Homo sapiens GN=BYSL PE=1 SV=3 | 2,01 | 2,02 | 1 | 2,059 |
| 2676 | sp|Q13572|ITPK1_HUMAN | HUMAN | Inositol-tetrakisphosphate 1-kinase OS=Homo sapiens GN=ITPK1 PE=1 SV=2 | 2,01 | 2,02 | 1 | 3,14 |
| 2677 | sp|P05089|ARGI1_HUMAN | HUMAN | Arginase-1 OS=Homo sapiens GN=ARG1 PE=1 SV=2 | 2,01 | 2,02 | 1 | 6,211 |
| 2678 | sp|Q9NRN5|OLFL3_HUMAN | HUMAN | Olfactomedin-like protein 3 OS=Homo sapiens GN=OLFML3 PE=2 SV=1 | 2,01 | 2,02 | 1 | 2,463 |
| 2679 | sp|Q9Y3C1|NOP16_HUMAN | HUMAN | Nucleolar protein 16 OS=Homo sapiens GN=NOP16 PE=1 SV=2 | 2,01 | 2,01 | 1 | 8,427 |
| 2680 | sp|P01781|HV320_HUMAN | HUMAN | Ig heavy chain V-III region GAL OS=Homo sapiens PE=1 SV=1 | 2,01 | 2,01 | 1 | 7,759 |
| 2681 | sp|Q96BQ3|TRI43_HUMAN | HUMAN | Tripartite motif-containing protein 43 OS=Homo sapiens GN=TRIM43 PE=1 SV=1 | 2,01 | 2,01 | 1 | 1,57 |
| 2682 | sp|P28799|GRN_HUMAN | HUMAN | Granulins OS=Homo sapiens GN=GRN PE=1 SV=2 | 2,01 | 2,01 | 1 | 2,361 |
| 2683 | sp|Q92851|CASPA_HUMAN | HUMAN | Caspase-10 OS=Homo sapiens GN=CASP10 PE=1 SV=3 | 2,01 | 2,01 | 1 | 2,687 |
| 2684 | sp|Q8WW33|GTSF1_HUMAN | HUMAN | Gametocyte-specific factor 1 OS=Homo sapiens GN=GTSF1 PE=1 SV=2 | 2,01 | 2,01 | 1 | 7,186 |
| 2685 | sp|Q02083|NAAA_HUMAN | HUMAN | N-acylethanolamine-hydrolyzing acid amidase OS=Homo sapiens GN=NAAA PE=1 SV=3 | 2,01 | 2,01 | 1 | 4,735 |
| 2686 | sp|P63172|DYLT1_HUMAN | HUMAN | Dynein light chain Tctex-type 1 OS=Homo sapiens GN=DYNLT1 PE=1 SV=1 | 2,01 | 2,01 | 1 | 15,93 |
| 2687 | sp|P48539|PCP4_HUMAN | HUMAN | Purkinje cell protein 4 OS=Homo sapiens GN=PCP4 PE=1 SV=3 | 2,01 | 2,01 | 1 | 25,81 |
| 2688 | sp|P32519|ELF1_HUMAN | HUMAN | ETS-related transcription factor Elf-1 OS=Homo sapiens GN=ELF1 PE=1 SV=2 | 2,01 | 2,01 | 1 | 2,908 |
| 2689 | sp|Q13885|TBB2A_HUMAN | HUMAN | Tubulin beta-2A chain OS=Homo sapiens GN=TUBB2A PE=1 SV=1 | 2 | 53,52 | 92 | 75,73 |
| 2690 | sp|P08134|RHOC_HUMAN | HUMAN | Rho-related GTP-binding protein RhoC OS=Homo sapiens GN=RHOC PE=1 SV=1 | 2 | 23,29 | 21 | 56,48 |
| 2691 | sp|P69891|HBG1_HUMAN | HUMAN | Hemoglobin subunit gamma-1 OS=Homo sapiens GN=HBG1 PE=1 SV=2 | 2 | 22,36 | 40 | 76,87 |
| 2692 | sp|P12236|ADT3_HUMAN | HUMAN | ADP/ATP translocase 3 OS=Homo sapiens GN=SLC25A6 PE=1 SV=4 | 2 | 15,91 | 8 | 32,89 |
| 2693 | sp|P57721|PCBP3_HUMAN | HUMAN | Poly(rC)-binding protein 3 OS=Homo sapiens GN=PCBP3 PE=2 SV=2 | 2 | 9,66 | 8 | 17,79 |
| 2694 | sp|Q8WWU7|ITLN2_HUMAN | HUMAN | Intelectin-2 OS=Homo sapiens GN=ITLN2 PE=2 SV=1 | 2 | 7,73 | 7 | 21,85 |
| 2695 | sp|P01766|HV305_HUMAN | HUMAN | Ig heavy chain V-III region BRO OS=Homo sapiens PE=1 SV=1 | 2 | 5,02 | 9 | 25 |
| 2696 | sp|P01593|KV101_HUMAN | HUMAN | Ig kappa chain V-I region AG OS=Homo sapiens PE=1 SV=1 | 2 | 4,89 | 9 | 37,96 |
| 2697 | sp|Q8WUP2|FBLI1_HUMAN | HUMAN | Filamin-binding LIM protein 1 OS=Homo sapiens GN=FBLIM1 PE=1 SV=2 | 2 | 2,18 | 2 | 5,898 |
| 2698 | sp|Q8NHV1|GIMA7_HUMAN | HUMAN | GTPase IMAP family member 7 OS=Homo sapiens GN=GIMAP7 PE=1 SV=1 | 2 | 2,15 | 2 | 6,667 |
| 2699 | sp|P01604|KV112_HUMAN | HUMAN | Ig kappa chain V-I region Kue OS=Homo sapiens PE=1 SV=1 | 2 | 2,14 | 2 | 16,67 |
| 2700 | sp|Q8NFF5|FAD1_HUMAN | HUMAN | FAD synthase OS=Homo sapiens GN=FLAD1 PE=1 SV=1 | 2 | 2,11 | 1 | 3,578 |
| 2701 | sp|Q13421|MSLN_HUMAN | HUMAN | Mesothelin OS=Homo sapiens GN=MSLN PE=1 SV=2 | 2 | 2,1 | 2 | 4,444 |
| 2702 | sp|P01611|KV119_HUMAN | HUMAN | Ig kappa chain V-I region Wes OS=Homo sapiens PE=1 SV=1 | 2 | 2,09 | 4 | 16,67 |
| 2703 | sp|Q9UL15|BAG5_HUMAN | HUMAN | BAG family molecular chaperone regulator 5 OS=Homo sapiens GN=BAG5 PE=1 SV=1 | 2 | 2,07 | 1 | 3,356 |
| 2704 | sp|O43286|B4GT5_HUMAN | HUMAN | Beta-1,4-galactosyltransferase 5 OS=Homo sapiens GN=B4GALT5 PE=2 SV=1 | 2 | 2,07 | 1 | 2,062 |
| 2705 | sp|Q9BXD5|NPL_HUMAN | HUMAN | N-acetylneuraminate lyase OS=Homo sapiens GN=NPL PE=1 SV=1 | 2 | 2,05 | 1 | 3,438 |
| 2706 | sp|Q9GZR7|DDX24_HUMAN | HUMAN | ATP-dependent RNA helicase DDX24 OS=Homo sapiens GN=DDX24 PE=1 SV=1 | 2 | 2,04 | 1 | 1,281 |
| 2707 | sp|Q6P6C2|ALKB5_HUMAN | HUMAN | RNA demethylase ALKBH5 OS=Homo sapiens GN=ALKBH5 PE=1 SV=2 | 2 | 2,04 | 1 | 7,36 |
| 2708 | sp|P49821|NDUV1_HUMAN | HUMAN | NADH dehydrogenase [ubiquinone] flavoprotein 1, mitochondrial OS=Homo sapiens GN=NDUFV1 PE=1 SV=4 | 2 | 2,02 | 2 | 6,897 |
| 2709 | sp|Q7Z6K5|ARPIN_HUMAN | HUMAN | Arpin OS=Homo sapiens GN=ARPIN PE=1 SV=1 | 2 | 2,02 | 1 | 5,31 |
| 2710 | sp|Q9UNW1|MINP1_HUMAN | HUMAN | Multiple inositol polyphosphate phosphatase 1 OS=Homo sapiens GN=MINPP1 PE=1 SV=1 | 2 | 2,02 | 1 | 2,259 |
| 2711 | sp|Q9H4A5|GLP3L_HUMAN | HUMAN | Golgi phosphoprotein 3-like OS=Homo sapiens GN=GOLPH3L PE=1 SV=1 | 2 | 2,01 | 2 | 10,53 |
| 2712 | sp|Q92785|REQU_HUMAN | HUMAN | Zinc finger protein ubi-d4 OS=Homo sapiens GN=DPF2 PE=1 SV=2 | 2 | 2,01 | 1 | 4,092 |
| 2713 | sp|P52655|TF2AA_HUMAN | HUMAN | Transcription initiation factor IIA subunit 1 OS=Homo sapiens GN=GTF2A1 PE=1 SV=1 | 2 | 2,01 | 1 | 2,926 |
| 2714 | sp|Q66K66|TM198_HUMAN | HUMAN | Transmembrane protein 198 OS=Homo sapiens GN=TMEM198 PE=1 SV=1 | 2 | 2,01 | 1 | 2,5 |
| 2715 | sp|A6NK58|LIPT2_HUMAN | HUMAN | Putative lipoyltransferase 2, mitochondrial OS=Homo sapiens GN=LIPT2 PE=3 SV=1 | 2 | 2,01 | 1 | 4,762 |
| 2716 | sp|Q96EY5|MB12A_HUMAN | HUMAN | Multivesicular body subunit 12A OS=Homo sapiens GN=MVB12A PE=1 SV=1 | 2 | 2,01 | 1 | 5,495 |
| 2717 | sp|Q5BKZ1|ZN326_HUMAN | HUMAN | DBIRD complex subunit ZNF326 OS=Homo sapiens GN=ZNF326 PE=1 SV=2 | 2 | 2,01 | 1 | 2,749 |
| 2718 | sp|P30536|TSPOA_HUMAN | HUMAN | Translocator protein OS=Homo sapiens GN=TSPO PE=1 SV=3 | 2 | 2,01 | 1 | 13,61 |
| 2719 | sp|P06889|LV405_HUMAN | HUMAN | Ig lambda chain V-IV region MOL OS=Homo sapiens PE=1 SV=1 | 2 | 2,01 | 1 | 23,58 |
| 2720 | sp|Q9Y4P1|ATG4B_HUMAN | HUMAN | Cysteine protease ATG4B OS=Homo sapiens GN=ATG4B PE=1 SV=2 | 2 | 2 | 1 | 4,835 |
| 2721 | sp|Q9UNL2|SSRG_HUMAN | HUMAN | Translocon-associated protein subunit gamma OS=Homo sapiens GN=SSR3 PE=1 SV=1 | 2 | 2 | 1 | 7,568 |
| 2722 | sp|Q9H3S4|TPK1_HUMAN | HUMAN | Thiamin pyrophosphokinase 1 OS=Homo sapiens GN=TPK1 PE=1 SV=1 | 2 | 2 | 1 | 3,704 |
| 2723 | sp|Q8TBF2|PGFS_HUMAN | HUMAN | Prostamide/prostaglandin F synthase OS=Homo sapiens GN=FAM213B PE=2 SV=1 | 2 | 2 | 2 | 9,596 |
| 2724 | sp|Q8NFJ5|RAI3_HUMAN | HUMAN | Retinoic acid-induced protein 3 OS=Homo sapiens GN=GPRC5A PE=1 SV=2 | 2 | 2 | 1 | 3,641 |
| 2725 | sp|Q9Y6A9|SPCS1_HUMAN | HUMAN | Signal peptidase complex subunit 1 OS=Homo sapiens GN=SPCS1 PE=1 SV=4 | 2 | 2 | 1 | 16,67 |
| 2726 | sp|Q9Y547|IFT25_HUMAN | HUMAN | Intraflagellar transport protein 25 homolog OS=Homo sapiens GN=HSPB11 PE=1 SV=1 | 2 | 2 | 1 | 17,36 |
| 2727 | sp|Q9Y3A6|TMED5_HUMAN | HUMAN | Transmembrane emp24 domain-containing protein 5 OS=Homo sapiens GN=TMED5 PE=1 SV=1 | 2 | 2 | 1 | 5,24 |
| 2728 | sp|Q9UIL1|SCOC_HUMAN | HUMAN | Short coiled-coil protein OS=Homo sapiens GN=SCOC PE=1 SV=2 | 2 | 2 | 1 | 11,32 |
| 2729 | sp|Q9NRP0|OSTC_HUMAN | HUMAN | Oligosaccharyltransferase complex subunit OSTC OS=Homo sapiens GN=OSTC PE=1 SV=1 | 2 | 2 | 1 | 8,054 |
| 2730 | sp|Q9NPG8|ZDHC4_HUMAN | HUMAN | Probable palmitoyltransferase ZDHHC4 OS=Homo sapiens GN=ZDHHC4 PE=2 SV=1 | 2 | 2 | 1 | 2,616 |
| 2731 | sp|Q9BUK0|CHCH7_HUMAN | HUMAN | Coiled-coil-helix-coiled-coil-helix domain-containing protein 7 OS=Homo sapiens GN=CHCHD7 PE=1 SV=1 | 2 | 2 | 2 | 21,18 |
| 2732 | sp|Q9BTL3|RAM_HUMAN | HUMAN | RNMT-activating mini protein OS=Homo sapiens GN=FAM103A1 PE=1 SV=1 | 2 | 2 | 1 | 12,71 |
| 2733 | sp|Q99735|MGST2_HUMAN | HUMAN | Microsomal glutathione S-transferase 2 OS=Homo sapiens GN=MGST2 PE=1 SV=1 | 2 | 2 | 1 | 9,524 |
| 2734 | sp|Q96IX5|USMG5_HUMAN | HUMAN | Up-regulated during skeletal muscle growth protein 5 OS=Homo sapiens GN=USMG5 PE=1 SV=1 | 2 | 2 | 1 | 25,86 |
| 2735 | sp|Q96B54|ZN428_HUMAN | HUMAN | Zinc finger protein 428 OS=Homo sapiens GN=ZNF428 PE=1 SV=2 | 2 | 2 | 1 | 7,447 |
| 2736 | sp|Q8N0U8|VKORL_HUMAN | HUMAN | Vitamin K epoxide reductase complex subunit 1-like protein 1 OS=Homo sapiens GN=VKORC1L1 PE=1 SV=2 | 2 | 2 | 1 | 6,25 |
| 2737 | sp|Q6QNY0|BL1S3_HUMAN | HUMAN | Biogenesis of lysosome-related organelles complex 1 subunit 3 OS=Homo sapiens GN=BLOC1S3 PE=1 SV=1 | 2 | 2 | 1 | 7,921 |
| 2738 | sp|Q6ICB0|DESI1_HUMAN | HUMAN | Desumoylating isopeptidase 1 OS=Homo sapiens GN=DESI1 PE=1 SV=1 | 2 | 2 | 1 | 4,762 |
| 2739 | sp|Q59GN2|R39L5_HUMAN | HUMAN | Putative 60S ribosomal protein L39-like 5 OS=Homo sapiens GN=RPL39P5 PE=5 SV=2 | 2 | 2 | 1 | 19,61 |
| 2740 | sp|Q53QV2|LBH_HUMAN | HUMAN | Protein LBH OS=Homo sapiens GN=LBH PE=1 SV=1 | 2 | 2 | 1 | 18,1 |
| 2741 | sp|Q16831|UPP1_HUMAN | HUMAN | Uridine phosphorylase 1 OS=Homo sapiens GN=UPP1 PE=1 SV=1 | 2 | 2 | 1 | 3,226 |
| 2742 | sp|Q14802|FXYD3_HUMAN | HUMAN | FXYD domain-containing ion transport regulator 3 OS=Homo sapiens GN=FXYD3 PE=2 SV=1 | 2 | 2 | 1 | 21,84 |
| 2743 | sp|Q01629|IFM2_HUMAN | HUMAN | Interferon-induced transmembrane protein 2 OS=Homo sapiens GN=IFITM2 PE=1 SV=2 | 2 | 2 | 1 | 12,12 |
| 2744 | sp|P82933|RT09_HUMAN | HUMAN | 28S ribosomal protein S9, mitochondrial OS=Homo sapiens GN=MRPS9 PE=1 SV=2 | 2 | 2 | 1 | 3,283 |
| 2745 | sp|P19957|ELAF_HUMAN | HUMAN | Elafin OS=Homo sapiens GN=PI3 PE=1 SV=3 | 2 | 2 | 1 | 13,68 |
| 2746 | sp|P18827|SDC1_HUMAN | HUMAN | Syndecan-1 OS=Homo sapiens GN=SDC1 PE=1 SV=3 | 2 | 2 | 1 | 5,484 |
| 2747 | sp|P06870|KLK1_HUMAN | HUMAN | Kallikrein-1 OS=Homo sapiens GN=KLK1 PE=1 SV=2 | 2 | 2 | 1 | 4,962 |
| 2748 | sp|P01717|LV403_HUMAN | HUMAN | Ig lambda chain V-IV region Hil OS=Homo sapiens PE=1 SV=1 | 2 | 2 | 1 | 17,76 |
| 2749 | sp|P01703|LV105_HUMAN | HUMAN | Ig lambda chain V-I region NEWM OS=Homo sapiens PE=1 SV=1 | 2 | 2 | 1 | 16,5 |
| 2750 | sp|O95190|OAZ2_HUMAN | HUMAN | Ornithine decarboxylase antizyme 2 OS=Homo sapiens GN=OAZ2 PE=1 SV=1 | 2 | 2 | 1 | 6,878 |
| 2751 | sp|P45954|ACDSB_HUMAN | HUMAN | Short/branched chain specific acyl-CoA dehydrogenase, mitochondrial OS=Homo sapiens GN=ACADSB PE=1 SV=1 | 1,98 | 2,92 | 7 | 12,04 |
| 2752 | sp|Q9Y2S6|TMA7_HUMAN | HUMAN | Translation machinery-associated protein 7 OS=Homo sapiens GN=TMA7 PE=1 SV=1 | 1,98 | 2,3 | 2 | 26,56 |
| 2753 | sp|Q16537|2A5E_HUMAN | HUMAN | Serine/threonine-protein phosphatase 2A 56 kDa regulatory subunit epsilon isoform OS=Homo sapiens GN=PPP2R5E PE=1 SV=1 | 1,98 | 2,2 | 2 | 5,996 |
| 2754 | sp|P11217|PYGM_HUMAN | HUMAN | Glycogen phosphorylase, muscle form OS=Homo sapiens GN=PYGM PE=1 SV=6 | 1,97 | 18,91 | 14 | 14,96 |
| 2755 | sp|Q9NPQ8|RIC8A_HUMAN | HUMAN | Synembryn-A OS=Homo sapiens GN=RIC8A PE=1 SV=3 | 1,97 | 2,41 | 5 | 8,098 |
| 2756 | sp|Q9NVP1|DDX18_HUMAN | HUMAN | ATP-dependent RNA helicase DDX18 OS=Homo sapiens GN=DDX18 PE=1 SV=2 | 1,97 | 2,13 | 1 | 1,791 |
| 2757 | sp|O75175|CNOT3_HUMAN | HUMAN | CCR4-NOT transcription complex subunit 3 OS=Homo sapiens GN=CNOT3 PE=1 SV=1 | 1,97 | 2,11 | 2 | 2,656 |
| 2758 | sp|Q9BQC3|DPH2_HUMAN | HUMAN | Diphthamide biosynthesis protein 2 OS=Homo sapiens GN=DPH2 PE=1 SV=1 | 1,97 | 2,01 | 1 | 3,885 |
| 2759 | sp|Q6NXG1|ESRP1_HUMAN | HUMAN | Epithelial splicing regulatory protein 1 OS=Homo sapiens GN=ESRP1 PE=1 SV=2 | 1,96 | 4,2 | 2 | 3,084 |
| 2760 | sp|P14091|CATE_HUMAN | HUMAN | Cathepsin E OS=Homo sapiens GN=CTSE PE=1 SV=2 | 1,96 | 2,04 | 1 | 1,995 |
| 2761 | sp|P06319|LV605_HUMAN | HUMAN | Ig lambda chain V-VI region EB4 OS=Homo sapiens PE=2 SV=1 | 1,96 | 2 | 1 | 15,27 |
| 2762 | sp|Q0JRZ9|FCHO2_HUMAN | HUMAN | F-BAR domain only protein 2 OS=Homo sapiens GN=FCHO2 PE=1 SV=2 | 1,95 | 2,67 | 2 | 2,469 |
| 2763 | sp|O00471|EXOC5_HUMAN | HUMAN | Exocyst complex component 5 OS=Homo sapiens GN=EXOC5 PE=1 SV=1 | 1,95 | 2,07 | 2 | 2,966 |
| 2764 | sp|P49459|UBE2A_HUMAN | HUMAN | Ubiquitin-conjugating enzyme E2 A OS=Homo sapiens GN=UBE2A PE=1 SV=2 | 1,95 | 2,07 | 3 | 28,95 |
| 2765 | sp|Q9NZ01|TECR_HUMAN | HUMAN | Very-long-chain enoyl-CoA reductase OS=Homo sapiens GN=TECR PE=1 SV=1 | 1,95 | 2,01 | 1 | 3,896 |
| 2766 | sp|Q13423|NNTM_HUMAN | HUMAN | NAD(P) transhydrogenase, mitochondrial OS=Homo sapiens GN=NNT PE=1 SV=3 | 1,95 | 1,99 | 1 | 1,197 |
| 2767 | sp|O95983|MBD3_HUMAN | HUMAN | Methyl-CpG-binding domain protein 3 OS=Homo sapiens GN=MBD3 PE=1 SV=1 | 1,93 | 1,97 | 1 | 5,155 |
| 2768 | sp|P23083|HV103_HUMAN | HUMAN | Ig heavy chain V-I region V35 OS=Homo sapiens PE=1 SV=1 | 1,92 | 4 | 4 | 20,51 |
| 2769 | sp|Q6UX04|CWC27_HUMAN | HUMAN | Peptidyl-prolyl cis-trans isomerase CWC27 homolog OS=Homo sapiens GN=CWC27 PE=1 SV=1 | 1,92 | 2,1 | 1 | 3,178 |
| 2770 | sp|P08637|FCG3A_HUMAN | HUMAN | Low affinity immunoglobulin gamma Fc region receptor III-A OS=Homo sapiens GN=FCGR3A PE=1 SV=2 | 1,92 | 1,96 | 1 | 4,331 |
| 2771 | sp|Q9BW83|IFT27_HUMAN | HUMAN | Intraflagellar transport protein 27 homolog OS=Homo sapiens GN=IFT27 PE=1 SV=1 | 1,9 | 2 | 1 | 4,301 |
| 2772 | sp|O43815|STRN_HUMAN | HUMAN | Striatin OS=Homo sapiens GN=STRN PE=1 SV=4 | 1,9 | 1,99 | 1 | 2,692 |
| 2773 | sp|Q93084|AT2A3_HUMAN | HUMAN | Sarcoplasmic/endoplasmic reticulum calcium ATPase 3 OS=Homo sapiens GN=ATP2A3 PE=1 SV=2 | 1,89 | 4,25 | 3 | 4,602 |
| 2774 | sp|Q6P3W7|SCYL2_HUMAN | HUMAN | SCY1-like protein 2 OS=Homo sapiens GN=SCYL2 PE=1 SV=1 | 1,89 | 1,98 | 1 | 1,507 |
| 2775 | sp|Q12965|MYO1E_HUMAN | HUMAN | Unconventional myosin-Ie OS=Homo sapiens GN=MYO1E PE=1 SV=2 | 1,88 | 6,11 | 3 | 3,159 |
| 2776 | sp|Q96AZ6|ISG20_HUMAN | HUMAN | Interferon-stimulated gene 20 kDa protein OS=Homo sapiens GN=ISG20 PE=1 SV=2 | 1,88 | 3,66 | 2 | 11,05 |
| 2777 | sp|Q4L180|FIL1L_HUMAN | HUMAN | Filamin A-interacting protein 1-like OS=Homo sapiens GN=FILIP1L PE=1 SV=2 | 1,86 | 2,03 | 1 | 1,233 |
| 2778 | sp|Q9H2H8|PPIL3_HUMAN | HUMAN | Peptidyl-prolyl cis-trans isomerase-like 3 OS=Homo sapiens GN=PPIL3 PE=1 SV=1 | 1,86 | 2 | 2 | 15,53 |
| 2779 | sp|O43924|PDE6D_HUMAN | HUMAN | Retinal rod rhodopsin-sensitive cGMP 3',5'-cyclic phosphodiesterase subunit delta OS=Homo sapiens GN=PDE6D PE=1 SV=1 | 1,86 | 1,92 | 1 | 8 |
| 2780 | sp|Q06265|EXOS9_HUMAN | HUMAN | Exosome complex component RRP45 OS=Homo sapiens GN=EXOSC9 PE=1 SV=3 | 1,85 | 1,97 | 2 | 5,239 |
| 2781 | sp|Q712K3|UB2R2_HUMAN | HUMAN | Ubiquitin-conjugating enzyme E2 R2 OS=Homo sapiens GN=UBE2R2 PE=1 SV=1 | 1,85 | 1,96 | 3 | 13,45 |
| 2782 | sp|O75594|PGRP1_HUMAN | HUMAN | Peptidoglycan recognition protein 1 OS=Homo sapiens GN=PGLYRP1 PE=1 SV=1 | 1,85 | 1,92 | 1 | 8,163 |
| 2783 | sp|Q92974|ARHG2_HUMAN | HUMAN | Rho guanine nucleotide exchange factor 2 OS=Homo sapiens GN=ARHGEF2 PE=1 SV=4 | 1,84 | 1,99 | 2 | 2,535 |
| 2784 | sp|O95817|BAG3_HUMAN | HUMAN | BAG family molecular chaperone regulator 3 OS=Homo sapiens GN=BAG3 PE=1 SV=3 | 1,84 | 1,92 | 1 | 2,261 |
| 2785 | sp|P52701|MSH6_HUMAN | HUMAN | DNA mismatch repair protein Msh6 OS=Homo sapiens GN=MSH6 PE=1 SV=2 | 1,83 | 1,93 | 2 | 1,25 |
| 2786 | sp|Q9H190|SDCB2_HUMAN | HUMAN | Syntenin-2 OS=Homo sapiens GN=SDCBP2 PE=1 SV=2 | 1,83 | 1,9 | 1 | 3,425 |
| 2787 | sp|Q10589|BST2_HUMAN | HUMAN | Bone marrow stromal antigen 2 OS=Homo sapiens GN=BST2 PE=1 SV=1 | 1,82 | 1,9 | 1 | 5,556 |
| 2788 | sp|P46952|3HAO_HUMAN | HUMAN | 3-hydroxyanthranilate 3,4-dioxygenase OS=Homo sapiens GN=HAAO PE=1 SV=2 | 1,82 | 1,89 | 1 | 3,497 |
| 2789 | sp|Q96JH7|VCIP1_HUMAN | HUMAN | Deubiquitinating protein VCIP135 OS=Homo sapiens GN=VCPIP1 PE=1 SV=2 | 1,82 | 1,88 | 1 | 1,146 |
| 2790 | sp|Q9UNE7|CHIP_HUMAN | HUMAN | E3 ubiquitin-protein ligase CHIP OS=Homo sapiens GN=STUB1 PE=1 SV=2 | 1,81 | 2,08 | 1 | 3,96 |
| 2791 | sp|Q96GQ7|DDX27_HUMAN | HUMAN | Probable ATP-dependent RNA helicase DDX27 OS=Homo sapiens GN=DDX27 PE=1 SV=2 | 1,81 | 1,94 | 2 | 3,392 |
| 2792 | sp|P35228|NOS2_HUMAN | HUMAN | Nitric oxide synthase, inducible OS=Homo sapiens GN=NOS2 PE=1 SV=2 | 1,81 | 1,89 | 1 | 0,954 |
| 2793 | sp|Q6PJG6|BRAT1_HUMAN | HUMAN | BRCA1-associated ATM activator 1 OS=Homo sapiens GN=BRAT1 PE=1 SV=2 | 1,81 | 1,88 | 1 | 1,462 |
| 2794 | sp|P10412|H14_HUMAN | HUMAN | Histone H1.4 OS=Homo sapiens GN=HIST1H1E PE=1 SV=2 | 1,8 | 14,75 | 9 | 26,94 |
| 2795 | sp|O75695|XRP2_HUMAN | HUMAN | Protein XRP2 OS=Homo sapiens GN=RP2 PE=1 SV=4 | 1,8 | 1,9 | 2 | 5,143 |
| 2796 | sp|Q9NRX2|RM17_HUMAN | HUMAN | 39S ribosomal protein L17, mitochondrial OS=Homo sapiens GN=MRPL17 PE=1 SV=1 | 1,8 | 1,89 | 1 | 4,571 |
| 2797 | sp|Q9BW91|NUDT9_HUMAN | HUMAN | ADP-ribose pyrophosphatase, mitochondrial OS=Homo sapiens GN=NUDT9 PE=1 SV=1 | 1,79 | 1,91 | 1 | 4 |
| 2798 | sp|Q92621|NU205_HUMAN | HUMAN | Nuclear pore complex protein Nup205 OS=Homo sapiens GN=NUP205 PE=1 SV=3 | 1,78 | 1,92 | 1 | 0,4473 |
| 2799 | sp|P04156|PRIO_HUMAN | HUMAN | Major prion protein OS=Homo sapiens GN=PRNP PE=1 SV=1 | 1,78 | 1,84 | 2 | 3,557 |
| 2800 | sp|P62760|VISL1_HUMAN | HUMAN | Visinin-like protein 1 OS=Homo sapiens GN=VSNL1 PE=1 SV=2 | 1,78 | 1,83 | 1 | 6,283 |
| 2801 | sp|P25098|ARBK1_HUMAN | HUMAN | Beta-adrenergic receptor kinase 1 OS=Homo sapiens GN=ADRBK1 PE=1 SV=2 | 1,77 | 2,05 | 2 | 2,467 |
| 2802 | sp|Q96SU4|OSBL9_HUMAN | HUMAN | Oxysterol-binding protein-related protein 9 OS=Homo sapiens GN=OSBPL9 PE=1 SV=2 | 1,77 | 1,93 | 3 | 3,804 |
| 2803 | sp|P01891|1A68_HUMAN | HUMAN | HLA class I histocompatibility antigen, A-68 alpha chain OS=Homo sapiens GN=HLA-A PE=1 SV=4 | 1,76 | 17,29 | 13 | 30,41 |
| 2804 | sp|Q14151|SAFB2_HUMAN | HUMAN | Scaffold attachment factor B2 OS=Homo sapiens GN=SAFB2 PE=1 SV=1 | 1,76 | 11,42 | 6 | 13,96 |
| 2805 | sp|Q8WWI5|CTL1_HUMAN | HUMAN | Choline transporter-like protein 1 OS=Homo sapiens GN=SLC44A1 PE=1 SV=1 | 1,76 | 1,81 | 1 | 1,674 |
| 2806 | sp|P51398|RT29_HUMAN | HUMAN | 28S ribosomal protein S29, mitochondrial OS=Homo sapiens GN=DAP3 PE=1 SV=1 | 1,75 | 2,18 | 1 | 3,769 |
| 2807 | sp|Q9P035|HACD3_HUMAN | HUMAN | Very-long-chain (3R)-3-hydroxyacyl-CoA dehydratase 3 OS=Homo sapiens GN=HACD3 PE=1 SV=2 | 1,75 | 1,86 | 2 | 6,906 |
| 2808 | sp|Q96P16|RPR1A_HUMAN | HUMAN | Regulation of nuclear pre-mRNA domain-containing protein 1A OS=Homo sapiens GN=RPRD1A PE=1 SV=1 | 1,75 | 1,85 | 1 | 3,526 |
| 2809 | sp|O95671|ASML_HUMAN | HUMAN | N-acetylserotonin O-methyltransferase-like protein OS=Homo sapiens GN=ASMTL PE=1 SV=3 | 1,75 | 1,84 | 1 | 2,738 |
| 2810 | sp|O43715|TRIA1_HUMAN | HUMAN | TP53-regulated inhibitor of apoptosis 1 OS=Homo sapiens GN=TRIAP1 PE=1 SV=1 | 1,75 | 1,83 | 1 | 17,11 |
| 2811 | sp|Q05086|UBE3A_HUMAN | HUMAN | Ubiquitin-protein ligase E3A OS=Homo sapiens GN=UBE3A PE=1 SV=4 | 1,74 | 1,92 | 1 | 1,486 |
| 2812 | sp|Q14790|CASP8_HUMAN | HUMAN | Caspase-8 OS=Homo sapiens GN=CASP8 PE=1 SV=1 | 1,74 | 1,86 | 2 | 7,098 |
| 2813 | sp|Q8NDH3|PEPL1_HUMAN | HUMAN | Probable aminopeptidase NPEPL1 OS=Homo sapiens GN=NPEPL1 PE=1 SV=3 | 1,74 | 1,85 | 2 | 7,266 |
| 2814 | RRRRRsp|Q9BTE7|DCNL5_HUMAN ^(b)^ | HUMAN | REVERSED DCN1-like protein 5 OS=Homo sapiens GN=DCUN1D5 PE=1 SV=1 | 1,74 | 1,8 | 1 | 3,797 |
| 2815 | sp|Q92608|DOCK2_HUMAN | HUMAN | Dedicator of cytokinesis protein 2 OS=Homo sapiens GN=DOCK2 PE=1 SV=2 | 1,73 | 1,82 | 1 | 0,4918 |
| 2816 | sp|P07311|ACYP1_HUMAN | HUMAN | Acylphosphatase-1 OS=Homo sapiens GN=ACYP1 PE=1 SV=2 | 1,73 | 1,81 | 1 | 13,13 |
| 2817 | sp|Q7L5D6|GET4_HUMAN | HUMAN | Golgi to ER traffic protein 4 homolog OS=Homo sapiens GN=GET4 PE=1 SV=1 | 1,73 | 1,78 | 1 | 3,67 |
| 2818 | sp|Q06828|FMOD_HUMAN | HUMAN | Fibromodulin OS=Homo sapiens GN=FMOD PE=1 SV=2 | 1,73 | 1,78 | 1 | 4,521 |
| 2819 | sp|Q53HC9|TSSC1_HUMAN | HUMAN | Protein TSSC1 OS=Homo sapiens GN=TSSC1 PE=1 SV=2 | 1,72 | 1,8 | 1 | 3,359 |
| 2820 | sp|Q13868|EXOS2_HUMAN | HUMAN | Exosome complex component RRP4 OS=Homo sapiens GN=EXOSC2 PE=1 SV=2 | 1,71 | 1,82 | 2 | 7,167 |
| 2821 | sp|Q86V21|AACS_HUMAN | HUMAN | Acetoacetyl-CoA synthetase OS=Homo sapiens GN=AACS PE=1 SV=1 | 1,71 | 1,81 | 1 | 1,488 |
| 2822 | sp|P16455|MGMT_HUMAN | HUMAN | Methylated-DNA--protein-cysteine methyltransferase OS=Homo sapiens GN=MGMT PE=1 SV=1 | 1,71 | 1,81 | 2 | 15,46 |
| 2823 | sp|Q14699|RFTN1_HUMAN | HUMAN | Raftlin OS=Homo sapiens GN=RFTN1 PE=1 SV=4 | 1,71 | 1,78 | 2 | 4,325 |
| 2824 | sp|Q8N392|RHG18_HUMAN | HUMAN | Rho GTPase-activating protein 18 OS=Homo sapiens GN=ARHGAP18 PE=1 SV=3 | 1,7 | 2,04 | 3 | 4,676 |
| 2825 | sp|O94992|HEXI1_HUMAN | HUMAN | Protein HEXIM1 OS=Homo sapiens GN=HEXIM1 PE=1 SV=1 | 1,7 | 1,83 | 2 | 6,128 |
| 2826 | sp|Q86SG5|S1A7A_HUMAN | HUMAN | Protein S100-A7A OS=Homo sapiens GN=S100A7A PE=1 SV=3 | 1,7 | 1,77 | 1 | 11,88 |
| 2827 | sp|O95218|ZRAB2_HUMAN | HUMAN | Zinc finger Ran-binding domain-containing protein 2 OS=Homo sapiens GN=ZRANB2 PE=1 SV=2 | 1,69 | 1,84 | 3 | 11,52 |
| 2828 | sp|P07902|GALT_HUMAN | HUMAN | Galactose-1-phosphate uridylyltransferase OS=Homo sapiens GN=GALT PE=1 SV=3 | 1,69 | 1,81 | 2 | 7,124 |
| 2829 | sp|P61009|SPCS3_HUMAN | HUMAN | Signal peptidase complex subunit 3 OS=Homo sapiens GN=SPCS3 PE=1 SV=1 | 1,69 | 1,76 | 1 | 6,667 |
| 2830 | sp|Q8TE77|SSH3_HUMAN | HUMAN | Protein phosphatase Slingshot homolog 3 OS=Homo sapiens GN=SSH3 PE=1 SV=2 | 1,68 | 1,8 | 2 | 3,642 |
| 2831 | sp|Q9H329|E41LB_HUMAN | HUMAN | Band 4.1-like protein 4B OS=Homo sapiens GN=EPB41L4B PE=2 SV=2 | 1,68 | 1,8 | 1 | 0,8889 |
| 2832 | sp|P48436|SOX9_HUMAN | HUMAN | Transcription factor SOX-9 OS=Homo sapiens GN=SOX9 PE=1 SV=1 | 1,68 | 1,78 | 2 | 3,34 |
| 2833 | sp|Q14508|WFDC2_HUMAN | HUMAN | WAP four-disulfide core domain protein 2 OS=Homo sapiens GN=WFDC2 PE=1 SV=2 | 1,67 | 1,74 | 1 | 11,29 |
| 2834 | sp|Q15172|2A5A_HUMAN | HUMAN | Serine/threonine-protein phosphatase 2A 56 kDa regulatory subunit alpha isoform OS=Homo sapiens GN=PPP2R5A PE=1 SV=1 | 1,66 | 2,15 | 1 | 2,675 |
| 2835 | sp|Q05655|KPCD_HUMAN | HUMAN | Protein kinase C delta type OS=Homo sapiens GN=PRKCD PE=1 SV=2 | 1,66 | 1,83 | 2 | 3,254 |
| 2836 | sp|Q8TF72|SHRM3_HUMAN | HUMAN | Protein Shroom3 OS=Homo sapiens GN=SHROOM3 PE=1 SV=2 | 1,66 | 1,8 | 1 | 0,4509 |
| 2837 | sp|Q92667|AKAP1_HUMAN | HUMAN | A-kinase anchor protein 1, mitochondrial OS=Homo sapiens GN=AKAP1 PE=1 SV=1 | 1,66 | 1,71 | 1 | 1,772 |
| 2838 | sp|Q9Y2B9|IPKG_HUMAN | HUMAN | cAMP-dependent protein kinase inhibitor gamma OS=Homo sapiens GN=PKIG PE=2 SV=1 | 1,66 | 1,7 | 1 | 21,05 |
| 2839 | sp|Q00534|CDK6_HUMAN | HUMAN | Cyclin-dependent kinase 6 OS=Homo sapiens GN=CDK6 PE=1 SV=1 | 1,65 | 3,19 | 2 | 7,055 |
| 2840 | sp|Q9BQE5|APOL2_HUMAN | HUMAN | Apolipoprotein L2 OS=Homo sapiens GN=APOL2 PE=1 SV=1 | 1,65 | 1,77 | 1 | 3,264 |
| 2841 | sp|Q8IUR0|TPPC5_HUMAN | HUMAN | Trafficking protein particle complex subunit 5 OS=Homo sapiens GN=TRAPPC5 PE=1 SV=1 | 1,65 | 1,74 | 1 | 5,319 |
| 2842 | sp|Q9UK23|NAGPA_HUMAN | HUMAN | N-acetylglucosamine-1-phosphodiester alpha-N-acetylglucosaminidase OS=Homo sapiens GN=NAGPA PE=1 SV=2 | 1,65 | 1,71 | 2 | 3,107 |
| 2843 | RRRRRsp|Q8WZ42|TITIN_HUMAN ^(b)^ | HUMAN | REVERSED Titin OS=Homo sapiens GN=TTN PE=1 SV=4 | 1,64 | 4,19 | 3 | 0,0524 |
| 2844 | sp|Q08378|GOGA3_HUMAN | HUMAN | Golgin subfamily A member 3 OS=Homo sapiens GN=GOLGA3 PE=1 SV=2 | 1,64 | 1,78 | 2 | 1,602 |
| 2845 | sp|P36551|HEM6_HUMAN | HUMAN | Oxygen-dependent coproporphyrinogen-III oxidase, mitochondrial OS=Homo sapiens GN=CPOX PE=1 SV=3 | 1,64 | 1,77 | 2 | 7,269 |
| 2846 | sp|P01707|LV204_HUMAN | HUMAN | Ig lambda chain V-II region TRO OS=Homo sapiens PE=1 SV=1 | 1,64 | 1,74 | 1 | 7,207 |
| 2847 | sp|Q15024|EXOS7_HUMAN | HUMAN | Exosome complex component RRP42 OS=Homo sapiens GN=EXOSC7 PE=1 SV=3 | 1,64 | 1,7 | 1 | 3,78 |
| 2848 | sp|Q14002|CEAM7_HUMAN | HUMAN | Carcinoembryonic antigen-related cell adhesion molecule 7 OS=Homo sapiens GN=CEACAM7 PE=1 SV=1 | 1,64 | 1,7 | 1 | 3,774 |
| 2849 | sp|Q3KQV9|UAP1L_HUMAN | HUMAN | UDP-N-acetylhexosamine pyrophosphorylase-like protein 1 OS=Homo sapiens GN=UAP1L1 PE=1 SV=2 | 1,63 | 3,77 | 2 | 3,945 |
| 2850 | sp|P63218|GBG5_HUMAN | HUMAN | Guanine nucleotide-binding protein G(I)/G(S)/G(O) subunit gamma-5 OS=Homo sapiens GN=GNG5 PE=1 SV=3 | 1,63 | 1,75 | 2 | 23,53 |
| 2851 | sp|Q96ST2|IWS1_HUMAN | HUMAN | Protein IWS1 homolog OS=Homo sapiens GN=IWS1 PE=1 SV=2 | 1,63 | 1,74 | 1 | 1,709 |
| 2852 | sp|Q9UQN3|CHM2B_HUMAN | HUMAN | Charged multivesicular body protein 2b OS=Homo sapiens GN=CHMP2B PE=1 SV=1 | 1,63 | 1,71 | 1 | 4,695 |
| 2853 | sp|Q15054|DPOD3_HUMAN | HUMAN | DNA polymerase delta subunit 3 OS=Homo sapiens GN=POLD3 PE=1 SV=2 | 1,63 | 1,7 | 1 | 2,79 |
| 2854 | sp|Q92922|SMRC1_HUMAN | HUMAN | SWI/SNF complex subunit SMARCC1 OS=Homo sapiens GN=SMARCC1 PE=1 SV=3 | 1,62 | 6,15 | 4 | 4,072 |
| 2855 | sp|P56385|ATP5I_HUMAN | HUMAN | ATP synthase subunit e, mitochondrial OS=Homo sapiens GN=ATP5I PE=1 SV=2 | 1,62 | 1,91 | 1 | 15,94 |
| 2856 | sp|Q9NZI8|IF2B1_HUMAN | HUMAN | Insulin-like growth factor 2 mRNA-binding protein 1 OS=Homo sapiens GN=IGF2BP1 PE=1 SV=2 | 1,62 | 1,8 | 1 | 2,773 |
| 2857 | sp|P35858|ALS_HUMAN | HUMAN | Insulin-like growth factor-binding protein complex acid labile subunit OS=Homo sapiens GN=IGFALS PE=1 SV=1 | 1,62 | 1,71 | 1 | 2,645 |
| 2858 | sp|Q15477|SKIV2_HUMAN | HUMAN | Helicase SKI2W OS=Homo sapiens GN=SKIV2L PE=1 SV=3 | 1,61 | 1,76 | 1 | 1,685 |
| 2859 | sp|O43516|WIPF1_HUMAN | HUMAN | WAS/WASL-interacting protein family member 1 OS=Homo sapiens GN=WIPF1 PE=1 SV=3 | 1,61 | 1,74 | 1 | 5,169 |
| 2860 | sp|Q9HD42|CHM1A_HUMAN | HUMAN | Charged multivesicular body protein 1a OS=Homo sapiens GN=CHMP1A PE=1 SV=1 | 1,61 | 1,73 | 2 | 8,673 |
| 2861 | sp|P57772|SELB_HUMAN | HUMAN | Selenocysteine-specific elongation factor OS=Homo sapiens GN=EEFSEC PE=1 SV=4 | 1,61 | 1,72 | 2 | 3,188 |
| 2862 | sp|Q8IWB7|WDFY1_HUMAN | HUMAN | WD repeat and FYVE domain-containing protein 1 OS=Homo sapiens GN=WDFY1 PE=1 SV=1 | 1,61 | 1,67 | 1 | 2,683 |
| 2863 | sp|P02775|CXCL7_HUMAN | HUMAN | Platelet basic protein OS=Homo sapiens GN=PPBP PE=1 SV=3 | 1,61 | 1,67 | 1 | 7,031 |
| 2864 | sp|P42167|LAP2B_HUMAN | HUMAN | Lamina-associated polypeptide 2, isoforms beta/gamma OS=Homo sapiens GN=TMPO PE=1 SV=2 | 1,6 | 20,2 | 18 | 31,72 |
| 2865 | sp|P29965|CD40L_HUMAN | HUMAN | CD40 ligand OS=Homo sapiens GN=CD40LG PE=1 SV=1 | 1,6 | 1,66 | 1 | 3,831 |
| 2866 | sp|Q9H2P9|DPH5_HUMAN | HUMAN | Diphthine methyl ester synthase OS=Homo sapiens GN=DPH5 PE=1 SV=2 | 1,6 | 1,65 | 1 | 6,667 |
| 2867 | sp|P08861|CEL3B_HUMAN | HUMAN | Chymotrypsin-like elastase family member 3B OS=Homo sapiens GN=CELA3B PE=1 SV=3 | 1,59 | 1,64 | 1 | 5,926 |
| 2868 | sp|Q14C86|GAPD1_HUMAN | HUMAN | GTPase-activating protein and VPS9 domain-containing protein 1 OS=Homo sapiens GN=GAPVD1 PE=1 SV=2 | 1,58 | 1,97 | 2 | 2,097 |
| 2869 | sp|Q9NV70|EXOC1_HUMAN | HUMAN | Exocyst complex component 1 OS=Homo sapiens GN=EXOC1 PE=1 SV=4 | 1,58 | 1,72 | 2 | 2,461 |
| 2870 | sp|Q92626|PXDN_HUMAN | HUMAN | Peroxidasin homolog OS=Homo sapiens GN=PXDN PE=1 SV=2 | 1,58 | 1,7 | 2 | 1,42 |
| 2871 | sp|Q92823|NRCAM_HUMAN | HUMAN | Neuronal cell adhesion molecule OS=Homo sapiens GN=NRCAM PE=1 SV=3 | 1,58 | 1,69 | 2 | 2,684 |
| 2872 | sp|Q15042|RB3GP_HUMAN | HUMAN | Rab3 GTPase-activating protein catalytic subunit OS=Homo sapiens GN=RAB3GAP1 PE=1 SV=3 | 1,58 | 1,65 | 1 | 1,325 |
| 2873 | sp|Q96F85|CNRP1_HUMAN | HUMAN | CB1 cannabinoid receptor-interacting protein 1 OS=Homo sapiens GN=CNRIP1 PE=1 SV=1 | 1,58 | 1,65 | 2 | 17,68 |
| 2874 | sp|Q00978|IRF9_HUMAN | HUMAN | Interferon regulatory factor 9 OS=Homo sapiens GN=IRF9 PE=1 SV=1 | 1,58 | 1,63 | 1 | 3,308 |
| 2875 | sp|P11166|GTR1_HUMAN | HUMAN | Solute carrier family 2, facilitated glucose transporter member 1 OS=Homo sapiens GN=SLC2A1 PE=1 SV=2 | 1,57 | 1,97 | 1 | 2,033 |
| 2876 | sp|Q9Y2H5|PKHA6_HUMAN | HUMAN | Pleckstrin homology domain-containing family A member 6 OS=Homo sapiens GN=PLEKHA6 PE=1 SV=4 | 1,56 | 1,67 | 1 | 1,145 |
| 2877 | sp|Q9Y5V0|ZN706_HUMAN | HUMAN | Zinc finger protein 706 OS=Homo sapiens GN=ZNF706 PE=1 SV=1 | 1,56 | 1,66 | 2 | 30,26 |
| 2878 | sp|Q9HCD5|NCOA5_HUMAN | HUMAN | Nuclear receptor coactivator 5 OS=Homo sapiens GN=NCOA5 PE=1 SV=2 | 1,55 | 2,05 | 1 | 1,554 |
| 2879 | sp|O60343|TBCD4_HUMAN | HUMAN | TBC1 domain family member 4 OS=Homo sapiens GN=TBC1D4 PE=1 SV=2 | 1,55 | 1,78 | 1 | 0,8475 |
| 2880 | sp|O43639|NCK2_HUMAN | HUMAN | Cytoplasmic protein NCK2 OS=Homo sapiens GN=NCK2 PE=1 SV=2 | 1,55 | 1,62 | 1 | 2,105 |
| 2881 | sp|Q5NUL3|FFAR4_HUMAN | HUMAN | Free fatty acid receptor 4 OS=Homo sapiens GN=FFAR4 PE=1 SV=2 | 1,55 | 1,61 | 1 | 3,714 |
| 2882 | sp|Q9HCN8|SDF2L_HUMAN | HUMAN | Stromal cell-derived factor 2-like protein 1 OS=Homo sapiens GN=SDF2L1 PE=1 SV=2 | 1,55 | 1,6 | 1 | 5,882 |
| 2883 | sp|Q92769|HDAC2_HUMAN | HUMAN | Histone deacetylase 2 OS=Homo sapiens GN=HDAC2 PE=1 SV=2 | 1,54 | 8,23 | 4 | 11,27 |
| 2884 | sp|Q14344|GNA13_HUMAN | HUMAN | Guanine nucleotide-binding protein subunit alpha-13 OS=Homo sapiens GN=GNA13 PE=1 SV=2 | 1,54 | 3,7 | 2 | 5,57 |
| 2885 | sp|Q9NTJ4|MA2C1_HUMAN | HUMAN | Alpha-mannosidase 2C1 OS=Homo sapiens GN=MAN2C1 PE=1 SV=1 | 1,54 | 1,64 | 1 | 1,827 |
| 2886 | sp|Q14118|DAG1_HUMAN | HUMAN | Dystroglycan OS=Homo sapiens GN=DAG1 PE=1 SV=2 | 1,53 | 1,63 | 1 | 1,229 |
| 2887 | sp|O75689|ADAP1_HUMAN | HUMAN | Arf-GAP with dual PH domain-containing protein 1 OS=Homo sapiens GN=ADAP1 PE=1 SV=2 | 1,53 | 1,6 | 1 | 2,674 |
| 2888 | sp|Q9NQL2|RRAGD_HUMAN | HUMAN | Ras-related GTP-binding protein D OS=Homo sapiens GN=RRAGD PE=1 SV=1 | 1,53 | 1,59 | 1 | 2,25 |
| 2889 | sp|Q9UJA5|TRM6_HUMAN | HUMAN | tRNA (adenine(58)-N(1))-methyltransferase non-catalytic subunit TRM6 OS=Homo sapiens GN=TRMT6 PE=1 SV=1 | 1,52 | 1,6 | 1 | 2,616 |
| 2890 | sp|Q9BZJ0|CRNL1_HUMAN | HUMAN | Crooked neck-like protein 1 OS=Homo sapiens GN=CRNKL1 PE=1 SV=4 | 1,51 | 1,62 | 2 | 2,948 |
| 2891 | RRRRRsp|Q16875|F263_HUMAN ^(b)^ | HUMAN | REVERSED 6-phosphofructo-2-kinase/fructose-2,6-bisphosphatase 3 OS=Homo sapiens GN=PFKFB3 PE=1 SV=1 | 1,51 | 1,59 | 1 | 1,538 |
| 2892 | sp|Q9Y244|POMP_HUMAN | HUMAN | Proteasome maturation protein OS=Homo sapiens GN=POMP PE=1 SV=1 | 1,51 | 1,57 | 1 | 7,092 |
| 2893 | sp|P48729|KC1A_HUMAN | HUMAN | Casein kinase I isoform alpha OS=Homo sapiens GN=CSNK1A1 PE=1 SV=2 | 1,51 | 1,57 | 1 | 2,671 |
| 2894 | sp|Q96K76|UBP47_HUMAN | HUMAN | Ubiquitin carboxyl-terminal hydrolase 47 OS=Homo sapiens GN=USP47 PE=1 SV=3 | 1,5 | 1,59 | 1 | 0,6545 |
| 2895 | sp|Q92565|RPGF5_HUMAN | HUMAN | Rap guanine nucleotide exchange factor 5 OS=Homo sapiens GN=RAPGEF5 PE=1 SV=1 | 1,5 | 1,56 | 1 | 1,552 |
| 2896 | sp|Q9BWG4|SSBP4_HUMAN | HUMAN | Single-stranded DNA-binding protein 4 OS=Homo sapiens GN=SSBP4 PE=1 SV=1 | 1,5 | 1,56 | 1 | 2,597 |
| 2897 | sp|O43865|SAHH2_HUMAN | HUMAN | Adenosylhomocysteinase 2 OS=Homo sapiens GN=AHCYL1 PE=1 SV=2 | 1,49 | 24,45 | 13 | 24,15 |
| 2898 | sp|P05162|LEG2_HUMAN | HUMAN | Galectin-2 OS=Homo sapiens GN=LGALS2 PE=1 SV=3 | 1,49 | 1,57 | 1 | 9,091 |
| 2899 | sp|O75629|CREG1_HUMAN | HUMAN | Protein CREG1 OS=Homo sapiens GN=CREG1 PE=1 SV=1 | 1,49 | 1,56 | 1 | 9,545 |
| 2900 | sp|P14621|ACYP2_HUMAN | HUMAN | Acylphosphatase-2 OS=Homo sapiens GN=ACYP2 PE=1 SV=2 | 1,49 | 1,55 | 1 | 13,13 |
| 2901 | sp|Q8IYB5|SMAP1_HUMAN | HUMAN | Stromal membrane-associated protein 1 OS=Homo sapiens GN=SMAP1 PE=1 SV=2 | 1,48 | 1,63 | 3 | 6,424 |
| 2902 | sp|Q9HD26|GOPC_HUMAN | HUMAN | Golgi-associated PDZ and coiled-coil motif-containing protein OS=Homo sapiens GN=GOPC PE=1 SV=1 | 1,48 | 1,59 | 2 | 4,329 |
| 2903 | sp|P52799|EFNB2_HUMAN | HUMAN | Ephrin-B2 OS=Homo sapiens GN=EFNB2 PE=1 SV=1 | 1,48 | 1,54 | 1 | 3,604 |
| 2904 | sp|Q9H1B7|I2BPL_HUMAN | HUMAN | Interferon regulatory factor 2-binding protein-like OS=Homo sapiens GN=IRF2BPL PE=1 SV=1 | 1,47 | 3,22 | 2 | 3,392 |
| 2905 | sp|O94886|CSCL1_HUMAN | HUMAN | CSC1-like protein 1 OS=Homo sapiens GN=TMEM63A PE=1 SV=3 | 1,47 | 1,55 | 1 | 1,487 |
| 2906 | sp|P40199|CEAM6_HUMAN | HUMAN | Carcinoembryonic antigen-related cell adhesion molecule 6 OS=Homo sapiens GN=CEACAM6 PE=1 SV=3 | 1,46 | 1,69 | 3 | 15,12 |
| 2907 | sp|Q0VF49|K2012_HUMAN | HUMAN | Uncharacterized protein KIAA2012 OS=Homo sapiens GN=KIAA2012 PE=2 SV=2 | 1,46 | 1,62 | 1 | 1,186 |
| 2908 | sp|Q8NEV8|EXPH5_HUMAN | HUMAN | Exophilin-5 OS=Homo sapiens GN=EXPH5 PE=1 SV=3 | 1,46 | 1,57 | 1 | 0,4525 |
| 2909 | sp|Q14728|MFS10_HUMAN | HUMAN | Major facilitator superfamily domain-containing protein 10 OS=Homo sapiens GN=MFSD10 PE=1 SV=1 | 1,46 | 1,53 | 1 | 2,857 |
| 2910 | sp|P15144|AMPN_HUMAN | HUMAN | Aminopeptidase N OS=Homo sapiens GN=ANPEP PE=1 SV=4 | 1,46 | 1,52 | 1 | 1,241 |
| 2911 | sp|Q16650|TBR1_HUMAN | HUMAN | T-box brain protein 1 OS=Homo sapiens GN=TBR1 PE=2 SV=1 | 1,46 | 1,51 | 1 | 1,76 |
| 2912 | sp|Q9Y3Q8|T22D4_HUMAN | HUMAN | TSC22 domain family protein 4 OS=Homo sapiens GN=TSC22D4 PE=1 SV=2 | 1,45 | 2,21 | 2 | 4,557 |
| 2913 | sp|Q14789|GOGB1_HUMAN | HUMAN | Golgin subfamily B member 1 OS=Homo sapiens GN=GOLGB1 PE=1 SV=2 | 1,45 | 1,75 | 2 | 0,7057 |
| 2914 | sp|Q9BQ13|KCD14_HUMAN | HUMAN | BTB/POZ domain-containing protein KCTD14 OS=Homo sapiens GN=KCTD14 PE=1 SV=2 | 1,45 | 1,54 | 1 | 5,882 |
| 2915 | sp|P38571|LICH_HUMAN | HUMAN | Lysosomal acid lipase/cholesteryl ester hydrolase OS=Homo sapiens GN=LIPA PE=1 SV=2 | 1,45 | 1,51 | 1 | 2,757 |
| 2916 | sp|Q9UM07|PADI4_HUMAN | HUMAN | Protein-arginine deiminase type-4 OS=Homo sapiens GN=PADI4 PE=1 SV=2 | 1,44 | 1,55 | 2 | 4,072 |
[truncated: 24,721 more chars]
